# Identification of pharmacodynamic biomarker hypotheses through literature analysis with IBM Watson

**DOI:** 10.1371/journal.pone.0214619

**Published:** 2019-04-08

**Authors:** Sonja Hatz, Scott Spangler, Andrew Bender, Matthew Studham, Philipp Haselmayer, Alix M. B. Lacoste, Van C. Willis, Richard L. Martin, Harsha Gurulingappa, Ulrich Betz

**Affiliations:** 1 Merck KGaA, Frankfurter Straße, Darmstadt, Germany; 2 IBM Watson Health, Almaden, California, United States of America; 3 EMD Serono, Middlesex Turnpike, Billerica, United States of America; 4 IBM Watson Health, New York, New York, United States of America; 5 IBM Watson Health, Cambridge, Massachusetts, United States of America; University of South Carolina, UNITED STATES

## Abstract

**Background:**

Pharmacodynamic biomarkers are becoming increasingly valuable for assessing drug activity and target modulation in clinical trials. However, identifying quality biomarkers is challenging due to the increasing volume and heterogeneity of relevant data describing the biological networks that underlie disease mechanisms. A biological pathway network typically includes entities (e.g. genes, proteins and chemicals/drugs) as well as the relationships between these and is typically curated or mined from structured databases and textual co-occurrence data. We propose a hybrid Natural Language Processing and directed relationships-based network analysis approach using IBM Watson for Drug Discovery to rank all human genes and identify potential candidate biomarkers, requiring only an initial determination of a specific target-disease relationship.

**Methods:**

Through natural language processing of scientific literature, Watson for Drug Discovery creates a network of semantic relationships between biological concepts such as genes, drugs, and diseases. Using Bruton’s tyrosine kinase as a case study, Watson for Drug Discovery’s automatically extracted relationship network was compared with a prominent manually curated physical interaction network. Additionally, potential biomarkers for Bruton’s tyrosine kinase inhibition were predicted using a matrix factorization approach and subsequently compared with expert-generated biomarkers.

**Results:**

Watson’s natural language processing generated a relationship network matching 55 (86%) genes upstream of BTK and 98 (95%) genes downstream of Bruton’s tyrosine kinase in a prominent manually curated physical interaction network. Matrix factorization analysis predicted 11 of 13 genes identified by Merck subject matter experts in the top 20% of Watson for Drug Discovery’s 13,595 ranked genes, with 7 in the top 5%.

**Conclusion:**

Taken together, these results suggest that Watson for Drug Discovery’s automatic relationship network identifies the majority of upstream and downstream genes in biological pathway networks and can be used to help with the identification and prioritization of pharmacodynamic biomarker evaluation, accelerating the early phases of disease hypothesis generation.

## Introduction

The usefulness of biomarkers can be seen in many contexts such as guiding treatment decisions in the form of FDA approved safety and efficacy biomarkers, as well as in the use of pharmacodynamic biomarkers which guide dose selection and demonstrate drug mechanism of action. The use of biomarkers in the early phases of clinical research has significantly enhanced the probability of success from phase I to approval from an average of 8.4% to 25.9% [1].

Traditionally, biomarker discovery has been dominated by proteomic and transcriptomic analysis methods resulting in the nomination of biomarkers that meet stringent regulatory requirements for robust validation and qualification, for example by the FDA Biomarker qualification program [2]. In contrast, pharmacodynamic and other biomarkers are identified using biological pathway knowledge and systems pharmacology approaches that are developed to monitor molecular responses to a therapy [3]. Once a biological hypothesis has been generated, it can be tested for disease context using different experimental systems. In the case of immunological disease and systemic lupus erythematosus (SLE) in particular, these range from human blood PBMCs, serum, and mouse lupus models, to validation in patients, using gene expression data or epigenetic markers from ongoing clinical trials [4, 5]. In this way, pharmacodynamic biomarkers in the research setting are used to provide mechanistic insights to confirm or reject the hypothesis that inhibition of a certain biological pathway will provide a therapeutic effect. This means that the search strategies are complex, iteratively adapted, and hypothesis generation and testing adhere to more loosely defined requirements than traditional biomarker discovery. Consequently, biomarkers used in this context are often discovered using information from multiple data sources, including unstructured data (e.g. scientific literature reports).

The events that follow Bruton’s Tyrosine Kinase (BTK) inhibition by small molecule drugs are excellent examples of the types of therapy responses that form the scientific basis for pharmacodynamic biomarker discovery:

1. Transient changes directly downstream of BTK changing on the order of seconds to minutes such as phospholipase C gamma 2 (PLCγ2) phosphorylation or other signaling events linked very closely to BTK activity. These biomarkers are generally validated in target engagement assays, such as protein phosphorylation assays or immunoprecipitation assays [6].
2. Proximal gene or protein changes that occur as a consequence of BTK pathway disruption. These are changes most likely seen on the order of hours to days in cells expressing BTK and can be validated by gene expression analysis or protein expression analysis methods.
3. Distal changes further downstream of BTK such as gene signatures, cell populations or antibody levels which can be correlated with disease. Changes may be due to effects resulting from BTK inhibition in not only the cells expressing BTK, but also in cells not directly affected by compound treatment.

Analyzing events that follow BTK inhibition illustrates that targeted pharmacodynamic biomarker discovery requires access to biological pathway information including the type of effect (e.g. inhibition, activation) in relation to the target of interest. A primary resource for this information is curated content and meta-databases. However, databases contain partly overlapping information on only selected protein sets, as discussed in a recent review [7], and in the case of BTK may contain conflicting information from studies more relevant to the field of oncology rather than immunology as BTK is a target for lymphoma treatment.

Unstructured data sources can serve as important alternatives to curated databases. Recently, automated text mining systems that leverage machine learning and natural language processing capabilities have experienced accelerated development to address this. Examples of resources that obtain pathway information from unstructured text are given in Table 1. Existing methods are currently based on the assumption that biomarker genes or proteins can be found by virtue of co-occurring in a window of text. DisGeNet is a prominent early example with fairly good retrieval (11%) of disease biomarker associations identified as compared to a curated database [8]. Our recent review of the literature has not provided any examples of therapy response biomarker discovery and the challenge for these pharmacodynamic biomarkers lies in automatically extracting information on relationships with direction and activity, since the existence of co-occurrence in text does not necessarily imply the existence of a relationship or indicate its directionality.

**Table 1:** References to biomedical relation extraction from text.

Reference	Data set	Methods /Dictionaries	Benchmarking datasets	Biological question
van Haagen et al. 2009 [9]	12 Million Medline Abstracts to July 2007	ER, CO, Compared performance of co-occurrence of keywords vs. entities, Context profiles and STRING	Biogrid, DIP, HPRD, IntAct, MINT, Reactome, and UniProt/Swiss-Prot were used to establish a set of 61,807 known human PPIs. Concept profiles improved sensitivity to 43% at 99% specificity with ROC 0.9.	Protein-Protein interactions Performance evaluation of different text mining scoring systems.
Bravo et al. 2014 [8]	MEDLINE abstracts annotated with MeSH term “pharmacological biomarkers” and “biological markers”: 164,300 abstracts	ER (bioNER) CO (tf/idf variant) Proteins: NCBI, Uniprot, HGNC Disease: UMLS	686172 cooccurrences between 2803 biomarkers and 2751 diseases	Biomarker-disease associations
Mihăilă, C. and Ananiadou, S. 2014 [10]	BioCause corpus, a collection of 19 open-access full-text journal articles pertaining to the subdomain of infectious diseases	RE, CS Using linguistic discourse “trigger” and “argument” models (cause-effect), semi-supervised learning classifier	BioCause corpus (infectious disease).	Infectious disease
Ahlers et. al. 2007 [11]	Semantic medline	ER, CO, RE	Gold standard annotation of 300 sentences (selected by co-occurring drugs and genes) with 850 predictions. 55% recall and 73% precision	Pharmacodynamic effects of drugs
Ahmed et. al. 2018 [12]	AIMed and BioInfer benchmark datasets which are subsets of PubMed articles annotated with Protein-Protein interactions	RE applied to train and evaluate tree recurrent neural network architecture.	Validation was done by 10-fold cross validation over AIMed and Bioinfer datasets which yielded F1-scores of 81% and 89% respectively	Protein-Protein interactions
Vlietstra et. al. 2017 [13]	Not defined (“triples from text and databases”)	ER Peregrine indexer, knowledge graph mapping UMLS Metathesaurus and semantic medline (and other resources: Uniprot, LODD)	Systematic literature review of 234 studies: 163 of 222 compounds ranked in top 2000 of 51 000 extracted compounds	Diagnostic biomarkers for migrane in blood and CSF Using a subgraph of migrane-related concepts, team ranked substances identified as pharmaceuticals
Chang et. al. 2017 [14]	PubMed queries (Keyword in title and abstract) 12052 articles	ER, CO Vocabularies defined by experts and including comparative toxicogenomics database, MeSH, Discovery Services, IGRhCellID and HyperCLDB. Sentence classifier trained on LOiverCancerMarkerRIF using word vectors released in 2013	Extracted 2128 gene/protein biomarker candidates and compared with several online resources (incl. liverome, MarkerRIF, GeneCards, Malacards, COSMIC). Comparison with HCC-related databases showed retrieval of between 20% (Liverome, omics data) and 50% (MarkerRIF, manually curated from literature)	Diagnostic biomarkers for hepatocellular carcinoma. Ranked biomarker citations based on journal impact factor, co-occurrence of biomarker with statistical terms together with a biomarker score (co-occurrence with species, source, disease, etc.)
Jurca 2016 et al. [15]	MEDLINE abstracts, API search for “breast cancer”, used those abstracts that contained genes 117,339 abstracts	ER (BeCAS), CO Gene-disease relationships from DisGeNet (CTD, UniProt) GEO DB	Non systematic: Compared co-expressed genes (experimental data) in GeneMania (PPI from BioGRID and pathway commons) compared to a community formed by network analysis	Diagnostic biomarkers for breast cancer Using gene-disease relationships, clustering and network analysis.

ER, entity recognition; CO, co-occurence analysis; RE, relationship extraction; CS, cross-sentence references; PPI, protein-protein interaction; ROC, area under receiver operating characteristic curve; CSF, cerebrospinal fluid.

In spite of a good fit with text mining capabilities, Table 1 therefore contains few examples of capabilities and performance characteristics suitable for extracting biological entities (such as genes and proteins) together with specific relationships between them (such as upregulation, phosphorylation or other specific interactions). Text mining technologies are currently being used directly in several applications around biomarker discovery, e.g. to annotate modules discovered from gene expression in the search for clinical biomarker signatures [16], but applications applying tools to real research questions with biological context while also providing levels of uncertainty and advanced analytics (clustering or ranking) are still emerging [17, 18].

Therefore, in the field of immunology today, the discovery of proximal and distal BTK biomarkers typically involves hypothesis generation that is driven by non-systematic literature analysis in combination with gene expression studies and pathway mapping resources to generate prioritized lists. As there is already a wide acceptance of higher levels of uncertainty in predictive tools routinely used in this space (such as GSEA or GWA), an unbiased quantitative literature analysis could be employed to significantly accelerate the process by focusing gene expression analysis and other systems biology tools towards higher quality hypotheses. Speed and adaptability of a search is particularly an issue because of the iterative nature of generating and rejecting hypotheses in the context of pharmacodynamic biomarkers, the presence of complex (text) data sources, and the need to consider timelines of events and other acknowledged limitations in current tools, such as the ability to address polypharmacology and variability.

This study was motivated by Merck’s R&D strategy to advance into digitally enhanced R&D and to explore the capabilities of cognitive computing in general and IBM Watson in particular to assist in that task. This study aimed to test the hypothesis that automated, high-throughput semantic relationship extraction from scientific literature, followed by a machine learning algorithm for novel inferences, applied herein using IBM Watson, has the potential to address the shortcomings of standard, co-occurrence-based text analysis in the context of pharmacodynamic biomarker discovery.

## Methods

### Generation of relationship networks and biomarker predictions

Watson for Drug Discovery (WDD) biological relationship network extraction has been described previously [19, 20]. Briefly, interaction networks of biological entities are extracted as guided by a catalog of known biological interaction types and relying on natural language parsing of sentences to establish explicit, directional relationships between known entities, including genes and proteins (hereafter, “genes” for brevity), drugs, chemicals, and diseases. Importantly, WDD identifies not only direct relations between two entities, but also indirect relationships; indirect relationship extraction allows for a greater degree of variation in the semantic positioning of the agent, verb and theme of the relationship, including the appearance of other arbitrary words in key roles, for example “bcl-2 is able to modulate transmembrane trafficking of p53” would be considered an indirect relationship between bcl-2 and p53. WDD relies on a rule-based approach for learning syntactic relationships that connect known entities through verbs and other trigger-phrases from a curated dictionary, each of which also has a canonical, or normalized, form. For example, “inhibit” and “inhibits” are trigger words used to identify events associating a drug to a gene, and alongside similar phrases, map to a canonical form “Regulation Negative”. Learned rules are then applied to a MEDLINE corpus (https://www.ncbi.nlm.nih.gov/pubmed/) for extraction of relationships.

The WDD extraction engine is built using IBM InfoSphere BigInsights SystemT [21]–a powerful information extraction system for extracting structured information from unstructured and semi-structured text. SystemT provides basic text analytic capabilities such as sentence splitting, token detection, natural language parse of sentences, etc. Deep-parsing components of SystemT are derived from an English Slot Grammar (ESG) parser [22]. that provides core linguistic analysis. In the following sections, we describe the individual components that are central to WDD relationship extraction: (1) entity identification and normalization and (2) relation recognition and normalization.

### Entity identification and normalization

In order to understand mentions of genes according to their context and map them to standardized identifiers, both rule-based [23] and machine-learning-based [24] approaches were integrated [25] for gene and protein extraction and normalization. To ignore mentions of generic protein families, such as “Histone”, and instead focus on mentions of specific genes and proteins such as “Histone H3” can be very challenging. For example, given a mention such as “GRK-1,3 and 5 have strong impact”, WDD needs to extract not only GRK-1, but also GRK-3 and GRK-5. Moreover, WDD needs to normalize genes to their canonical forms (their standard name); a key challenge here is that genes often share synonyms or abbreviations. Thus, WDD must understand mentions of genes according to their context and map them to standardized identifiers. To this end, WDD uses a hybrid model for gene extraction and normalization. The gene annotation process involves three steps: candidate generation, candidate selection, and entity normalization. For purposes of text inference, and specifically for biomarker discovery, distinguishing the gene from its protein product in text is not essential, therefore, WDD does not explicitly differentiate between genes and proteins.

### Gene candidate generation

To ensure high recall for the gene extraction task, WDD relies upon a comprehensive dictionary of human genes compiled from NCBI [26], the UniProt KnowledgeBase [27], HUGO [28], and CTD [29]. This dictionary contains approximately 461,000 entries, where each entry is mapped to one of approximately 37,500 gene canonical forms. When generating candidates, WDD employs a combination of dictionary-matching, pattern-matching, and abbreviation-matching rules to maximize recall while maintaining precision; these are summarized below and described in greater detail in a recent technical publication [20].

Dictionary-matching rules: Because it is infeasible for a dictionary to capture all possible gene synonyms used in the literature, WDD supports fuzzy, or proximal matching of dictionary terms, i.e. the identification of phrases in text that are similar to, but not exactly matching, phrases in the annotation dictionary, which for example supports extraction of names with variations in the use of space and hyphen characters. WDD also accounts for different levels of ambiguity in the dictionary terms. During dictionary compilation, each dictionary term is semi-automatically categorized according to length and character complexity into one of three categories: unambiguous terms like “G protein-coupled receptor kinase 2”, ambiguous terms like “ATM” (which could conceivably be used as an abbreviation to refer to any number of non-gene concepts), and very risky terms like “C1” and “C2” (which could additionally stand for table and figure numbers, etc.). The dictionary at time of writing contains approximately 429,000, 17,000, and 500 terms in each category respectively. Each mention that matches some dictionary term is assigned a confidence score of “high”, “medium”, or “low”, depending on the category of the term. Mentions with a confidence score of medium or low are further verified using the context-based classifier described in the Candidate Selection section.

Pattern- and abbreviation-matching rules: Pattern matches concern examples like “ERK1/2”, whereupon detection of “ERK1” as a gene annotation, the immediate local context is inspected and the character pattern “/2” is identified as shorthand for “ERK2” and normalized accordingly. Abbreviation matching concerns dynamic detection of context-specific abbreviations in text, such that in a context like “angiotensin-converting enzyme (ACE)”, the abbreviation phrase “ACE” can be confidently extracted and normalized in the same manner as its definition “angiotensin-converting enzyme”, independent of the confidence level that “ACE” is assigned in any dictionary, or indeed whether it is a dictionary term at all. Dynamic abbreviation phrases identified in this way are then extracted across the entire text of the document so that subsequent mentions of e.g. “ACE” can be understood correctly. Since the meaning of each abbreviation can only be said to be constant within the particular document in question, phrases identified as abbreviations do not carry over into other documents.

### Candidate selection

The context surrounding terms provides important information for resolving ambiguities resulting from abbreviation and term overlap. In order to exploit the cues in the surrounding context, we trained a naïve Bayes classifier that uses features derived from the neighboring context of a putative protein mention. WDD uses the sentence containing the discovered mention as the context span and considers all words present to the left and right of the mention, the number of gene mentions in the sentence, and whether or not the two immediate words to the left and right of the discovered mention are gene names. In addition to this model, the classification step involves application of a series of manually curated rules that filter medium and low confidence gene candidates based on key phrases and text patterns that denote a non-gene context.

To evaluate the classifier quantitatively, we compare its output to a sample of documents manually curated with the expected or ground truth annotations. Two ground truth corpora are utilized for this purpose, comprised of 100 MEDLINE abstracts prepared in collaboration with subject matter experts [19] (826 gene annotations) and all 347 MEDLINE abstracts from the BioNLP 2011 gene extraction challenge “GENIA” (https://sites.google.com/site/bionlpst/home/genia-event-extraction-genia) (5,301 gene annotations) respectively. As WDD development operates on agile model of continuous development and deployment [30], evaluation is performed within a testing framework which automatically executes upon every software build. This evaluation is performed after all document pre-processing, annotation, normalization and other post-processing steps have been performed ensuring that the evaluation metrics reflect the final, user-facing result. At the time of writing, gene classification in the collaboratively curated ground truth achieves 91.3% precision, 74.0% recall, and 81.7% F1 score and 77.0% precision, 67.8% recall, and 72.1% F1 score in the BioNLP 2013 challenge corpus. The difference in performance between these two datasets is primarily due to the BioNLP challenge dataset including ambiguous or otherwise non-specific annotations such as “these genes” or “a variety of cytokines”, which are not generally desired in WDD output, but nevertheless affect the annotator’s quantitative performance results in this corpus.

To assess annotation performance at full-corpus scale (28,278,250 MEDLINE abstracts at the time of writing), the classifier is also evaluated qualitatively. This is done using full-corpus scale annotation histograms that relay the most frequently annotated phrases and canonical names which result, in addition to numerous other aggregated statistics calculated across the total MEDLINE annotation count of 33,767,950 gene mentions (averaging 1.19 gene mentions per abstract, including document titles). User-provided annotation feedback is also used as a means of identifying and resolving annotation issues.

### Entity normalization

To map a mention using a synonym or alias back to the correct canonical gene, WDD adopts the idea of unigram language models from computational linguistics [31] and builds “context models” for genes. For example, synonym “D1” maps back to both the Dopamine Receptor D1 (DRD1) and Leiomoden 1 (LMOD1). Whenever WDD identifies D1 as a gene mention, it analyzes the surrounding words for clues to identify the correct canonical gene, allowing disambiguation. For example, a mention of “dopamine” or the family name “GPCR” in the abstract would indicate that the abstract was discussing Dopamine Receptor D1 (DRD1); these patterns are identified through training a machine learning model which utilizes data about the other occurrences of each candidate gene across the literature.

In addition to the use of unstructured context information for gene disambiguation, WDD also leverages structured data associated with each of the candidate genes, and the document in which the annotation occurs. The structured data used for candidate genes is sourced from the Gene Ontology (GO) [32, 33] and used to increase the context model score for each candidate gene if any of the GO labels also occur in the document text. The structured data used for documents depends on the corpus in question, for example, MEDLINE abstracts include curated Medical Subject Headers (MeSH); this document metadata is used in the machine learning model in an equivalent manner to the context words extracted from the document text.

A prior technical publication describes the gene disambiguation algorithm in greater detail through a worked example of disambiguating a mention of “Drp1”, which could mean one of many genes depending on the context; the other processes mentioned here are also described in greater detail [20].

### Event extraction and normalization

To extract relationships, WDD uses a catalog of gene-gene relation types and other biologically relevant verbs, together with natural language parsing of sentences. The catalog is maintained as a list structure similar to that used in the BioNLP 2013 pathway extraction task. The goal of extraction is to obtain triples, each comprising:

1. A relation: an element appearing in the catalog of relationships
2. An agent: a gene that is causally active in an event.
3. A theme: a gene that undergoes the effects of an interaction.

Entities (agents and themes) and the relationship between them are collectively referred to as events.

Depending on the relation, extracted triples may or may not possess directionality. Binding relationships, for instance, do not represent directional relationships, whereas phosphorylation is an example of a relation that has a clear causal agent and affected theme.

WDD extracts two kinds of triples that capture relationships between genes–direct and indirect. The first is a simple triple where there is a direct relationship between known entities. The following two sentences express direct relationships between known entities Plk1 and RSK1, and between p53 and Notch1: “In addition, studies on HeLa cells using Plk1 siRNA interference and overexpression showed that phosphorylation of RSK1 increased upon interference and decreased after overexpression, suggesting that Plk1 inhibits RSK1.” “These data indicate that p53 negatively regulates Notch1 activation during T cell development.” The second is a relationship where the genes are involved in more complex and indirect relationships. In the following examples we see that WDD extracts relationships between bcl-2 and p53 that are indirect, since different processes are involved in their interaction: “Silencing of Bcl-2 induced massive p53-dependent apoptosis.” “Our data suggests that bcl-2 is able to modulate transmembrane trafficking of p53.”

### Learning patterns for event extraction

WDD applies rules over natural language parse trees for relation detection. Each sentence is run through the IBM ESG Parser and the dependency parse structure is examined for syntactic connections between the labeled relationships, agents, and themes. WDD applies complex rules where indirect interactions are involved. For example, a sentence such as “ATM-mediated phosphorylation of p53 at serine 15.” contains an indirect interaction, “mediated,” between the agent and the main relation, phosphorylation, i.e., “ATM-mediated” is an adjective modifying “phosphorylation”:

amod(phosphorylation, ATM-mediated) prep_of(phosphorylation, p53)

A rule is applied to allow for any indirect relationship (from the relationship catalog) to act as a connector between an agent and the relationship.

WDD applies rules where agents and themes are not simply genes but rather processes or events involving a gene. For example, in the sentence “Our data suggests that bcl-2 is able to modulate transmembrane trafficking of p53.” the relation “modulate” is connected to an entity “p53” via a process that is captured in the phrase “transmembrane trafficking of p53”. Parse structures for every gene-gene relation in the labeled training data are examined in the above manner to collect a set of rules that describe how relationships and entities might relate to one another. Although the set of rules is not exhaustive and will not identify every possible relationship, the high quality of the rules yields precision and recall sufficient to create a network well suited to relationship prediction [20].

### Inference from networks

Collaborative Filtering [34] is the process of using known connections in a network to predict possible new connections. While this approach has been applied extensively to predict human preferences, the application of this method for predicting new biological connections is less well studied. Our approach to doing prediction over the generated network of gene relationships uses matrix factorization (MF) [35]. This technique can use the discovered triples directly to simultaneously predict many kinds of relationships. The process of evaluating the consistency of a relationship between entities, or between entities and a property, starts with extracting the known relationships from publications, as described in earlier sections. These relationships are then represented as a binary matrix. The score in this matrix for the relationship to be evaluated is set to zero. This matrix is then factored into H*W, where H and W represent smaller, dense matrices of a fixed, lesser dimensionality. The score of the relationship is its value in the product matrix of H*W. This score is compared to a set of comparable existing relationships evaluated in a similar manner in order to obtain a ranking of the relationship.

Given a set of agents (M) and a set of Targets/Properties (N), and directional relationships extracted from publications of the form m → n, where *m* ∈ *M* & *n* ∈ *N*, calculate the relative consistency of any particular m,n pair as follows:

1. Build a binary matrix, X, of dimension M x N containing a 1 in the mth row, nth column iff m→n exists in the scientific literature.
2. Set X[a,b] = 0, for the relationship a→b, whose consistency is to be evaluated.
3. Create a factorization of X, such that:
  - *L*_*ij*_(**W**_i*_,H_*j_): loss at (*i*, *j*)
  - We assume the input is non-negative.
  - Find best model:
    $$\underset{W,H}{\min}\sum_{(i,j)\in Z}L_{ij}(W_{i*},H_{*j})$$
  - In our implementation we use Spark ALS (Alternating Least Squares Matrix Factorization) for implementation of matrix factorization. More details about the ALS document in Spark:
    – https://spark.apache.org/docs/latest/api/java/org/apache/spark/mllib/recommendation/ALS.html
    – http://spark.apache.org/docs/latest/mllib-collaborative-filtering.html#collaborative-filtering
  - Here are some brief explanations about the parameters along with default values.
    – *numBlocks* is the number of blocks used to parallelize computation (set to -1 to auto-configure). (-1)
    – *rank* is the number of latent factors in the model. (20)
    – *Iterations* (20)
    – *lambda* specifies the regularization parameter in ALS. (0.1)
    – *implicitPrefs* specifies whether to use the *explicit feedback* ALS variant or one adapted for *implicit feedback* data. (implicit)
    – *alpha* is a parameter applicable to the implicit feedback variant of ALS that governs the *baseline* consistency in preference observations. (0.1)
4. The resulting H,W matrices can be multiplied to produce a new matrix X_2_. The value of X_2_[a,b] is the relative consistency score for the relationship a→b.
5. Repeat this process for a set of comparable relationships to use as a background population. This can be some representative sample of all existing relationships in the literature.

### Biological networks and performance evaluation

The WDD BTK network consisted of 64 gene upstream of BTK and 103 genes downstream of BTK. This network was compared to a BTK network from Clarivate Analytics MetaBase (https://clarivate.com/products/metacore/). MetaBase provides manually curated high quality systems biology content with nearly 1.7 million molecular interactions from over 1,600 pathway maps, and more than 230,000 disease-gene associations. A comparative study made by Shmelkov et. al. for transcriptional regulatory pathways in humans for seven well-studied transcription factors indicated that MetaBase had significantly high overlap with 10 other commonly used pathway databases [36].

The BTK network from MetaBase consisted of genes that were up to four links from BTK, upstream or downstream, with each link representing a directional physical interaction such as phosphorylation, receptor binding, ubiquitination, etc. As described, WDD extracts information on direct as well as indirect relationships. Since direct relationships are more likely to be transient changes upon BTK inhibition which were not in the focus of this study, we removed directly interacting proteins after ranking.

### Data and code availability

All data generated or analyzed during this study are included in the published article and its supplementary information files (S1 and S2 Tables). A free 30-day trial of WDD is available at https://content.mkt.watson-health.ibm.com/whls-2018-wdd-free-trial.html.

## Results

**Fig 1:**
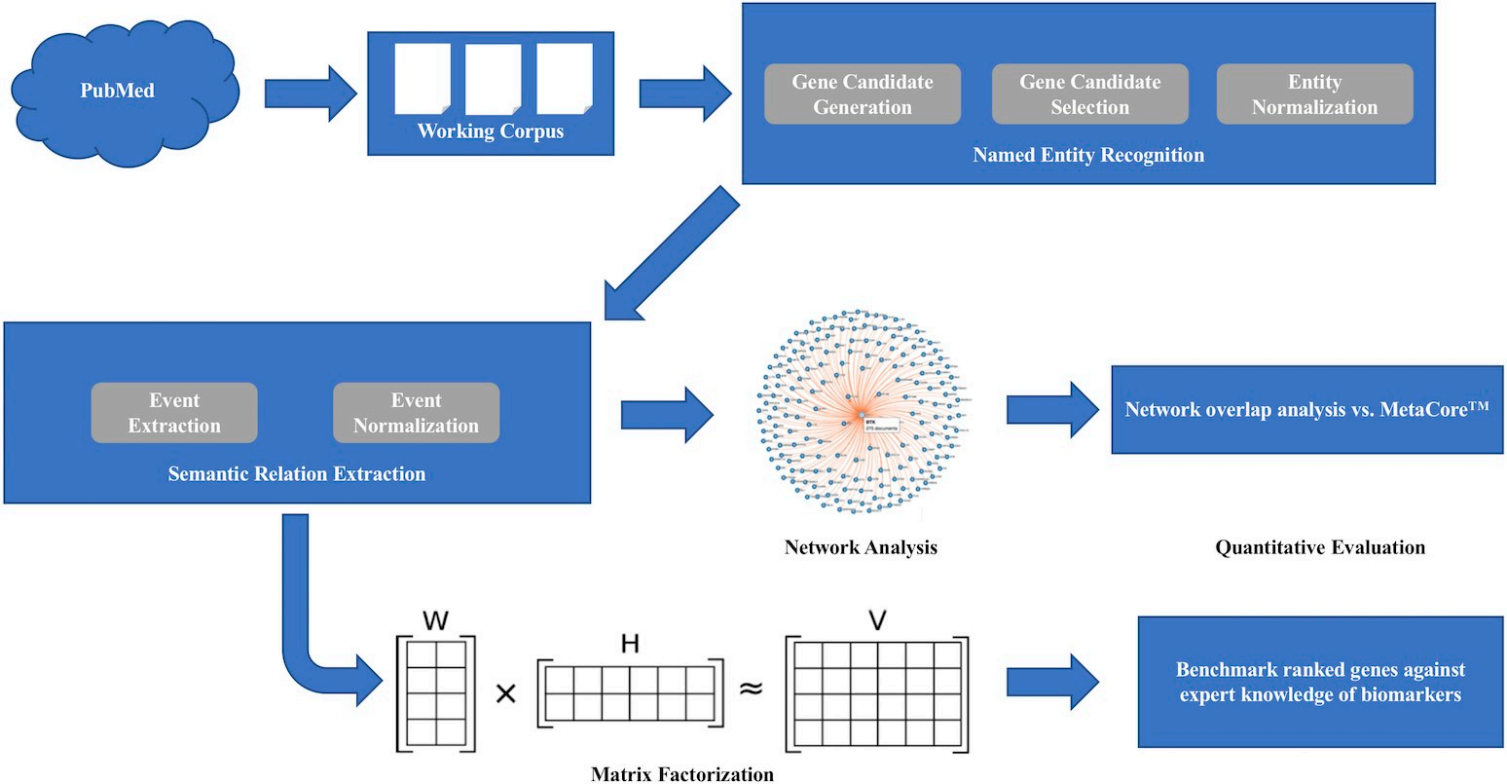
Overview of pipeline used for identification of putative BTK inhibition biomarkers. Pubmed abstracts were used as an input for generating a working corpus from which gene entities were identified, selected, and normalized for generation of a relationship network. This relationship network was used for comparison to the MetaCore curated database and further analysis using matrix factorization for the prediction of potential BTK biomarkers and compared to a list of potential BTK biomarkers created by subject matter experts.

Fig 1 shows the end-to-end approach used for identification of putative biomarkers using literature mining and network analysis. Our method begins with extracting all of the biological relationships between genes and representing them as a network. A small part of this network for BTK is depicted in S1 Fig. Evaluation of each gene-gene connection in detail reveals sentence-level extractions (S2 Fig), which in aggregate create a list of directional connections between genes.

To assess the accuracy of our approach for pharmacodynamic biomarker identification, we compared the networks generated by WDD and Metabase for our gene of interest, BTK. Of the 64 genes upstream of BTK in the WDD network, 55 of them (86%) were also found upstream of BTK in Metabase. Of the 103 genes downstream of BTK in the WDD network, 98 of them (95%) were also found downstream of BTK in the Metabase network. These results show that WDD was able to accurately identify genes up and downstream of BTK.

Overlap may be good in well-established canonical pathways and the quality of data may differ depending on the biological pathway studied. Differences in methods for determining relevant interactions (such as high throughput or others) and variability arising from different tissue or cell line origins are known challenges for direct quantitative comparisons [37].

**Fig 2:**
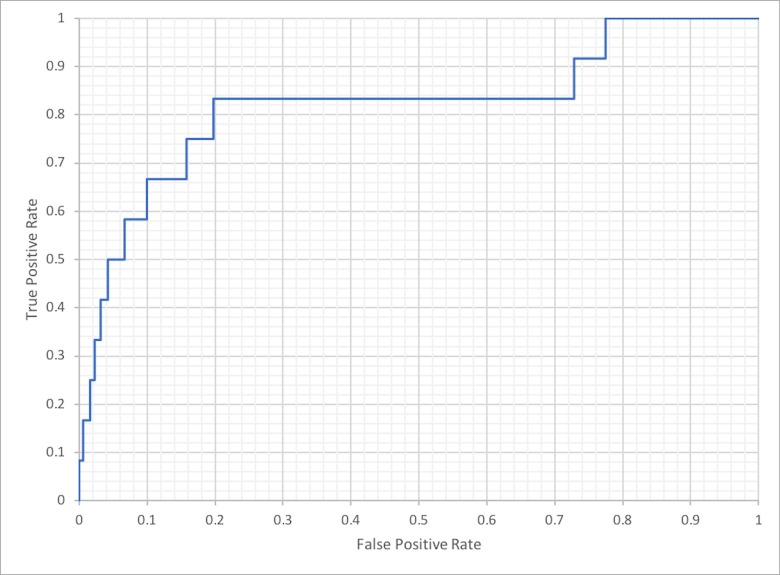
Receiver operating characteristic curve of WDD-predicted versus known BTK interactions. Resulting receiver operating characteristic curve from analysis comparing WDD-predicted BTK biomarker ranking to a subject matter expert-derived list of potential BTK biomarkers. Area under the curve = 0.82.

Next, we tested the ability of the WDD gene network to predict new indirect interactions between downstream genes impacted by BTK, based on what is already known in the network about these genes. In total there were 103 downstream genes found in the WDD network extracted from sentences in MEDLINE abstracts. We then used matrix factorization to rank all remaining genes based on strength of downstream association to BTK. This is done by representing the sentence-level gene-gene connections identified earlier as a matrix (S3 Fig and S1 Table), with a number in each cell indicating how many articles there are with a sentence describing a relationship of gene(row) -> gene(column). The top 50 results of this ranking are shown in Table 2 and the full 13,595 results shown in Supplemental Table 2. This ranking was then compared to a known set of BTK biomarkers identified by Merck subject matter experts, only one of which was in the set of 103 genes originally known to WDD to be one step downstream of BTK. As shown in Table 3, matrix factorization analysis predicted 11 of 13 genes identified by Merck subject matter experts in the top 20% of the 13,595 ranked genes, including 7 in the top 5%. The area under the receiver operating characteristics (ROC) curve in Fig 2 demonstrates that gene ordering compared to these 13 genes was significantly more accurate than chance, with an area under the curve of 0.82. This result demonstrates that the Watson gene-gene network generation pipeline can identify new hypotheses that match expert analyses, i.e., predicting unknown associations based on known associations observed in literature.

**Table 2:** Top 50 genes predicted by WDD to be downstream of BTK.

Gene	Score	Known Downstream to BTK (1 = yes, 0 = no)	Rank (including known)
*AKT1*	0.54458815	1	1
*NFKB1*	0.5154992	1	2
*TNF*	0.50323606	0	3
*IFNA1*	0.4434148	1	4
*TP53*	0.4385939	1	5
*EPHB2*	0.4313362	1	6
*IL6*	0.42688292	1	7
*INS*	0.41607705	1	8
*MAPK8*	0.41537482	1	9
*STAT3*	0.38802144	0	10
*IL2*	0.3744676	1	11
*MAPK3*	0.36648414	0	12
*BCR*	0.35648954	1	13
*VEGFA*	0.3489667	0	14
*IL10*	0.34443948	1	15
*IL4*	0.3414984	0	16
*IFNG*	0.33224836	0	17
*JUN*	0.3214404	0	18
*CRK*	0.31818953	0	19
*TGFB1*	0.3179956	0	20
*EGFR*	0.31714404	1	21
*CD4*	0.3144715	0	22
*TLR4*	0.31039178	1	23
*IL8*	0.303452	0	24
*STAT5A*	0.30185032	0	25
*EGF*	0.30117995	0	26
*IGHV1-2*	0.30093542	0	27
*CBL*	0.29676566	1	28
*STAT1*	0.2944623	1	29
*RAC1*	0.28429946	1	30
*MTOR*	0.28102204	1	31
*SYK*	0.28000763	1	32
*ABL1*	0.27899128	1	33
*IL17A*	0.27572146	0	34
*MYC*	0.27440986	1	35
*CD34*	0.27148065	1	36
*CSF2*	0.2702503	0	37
*CD40*	0.2699324	1	38
*CD8A*	0.2696973	0	39
*JAK2*	0.26840165	0	40
*VAV1*	0.26694292	0	41
*FAS*	0.26671404	1	42
*BCL2*	0.2651292	1	43
*AIMP2*	0.26320416	0	44
*LYN*	0.2610977	1	45
*GRB2*	0.26066443	0	46
*PI3*	0.25447765	1	47
*CASP3*	0.25388432	0	48
*SRC*	0.25309193	0	49
*CDKN1A*	0.25100806	0	50

Includes known genes (those already in WDD’s network). Full table of 13,595 ranked genes available in supplemental Table 1.

**Table 3:** WDD matrix factorization ranking of known BTK targets.

Gene	WDD Rank	Percentile
*BCL2A1*	2240	16%
*CCL3*	506	4%
*EBI3*	124	1%
*EGR1*	384	3%
*EGR3*	1448	11%
*IKZF1*	379	3%
*IL4I1*	2775	20%
*IRF4*	284	2%
*RASGRP1*	997	7%
*TNF*	3	0.02%
*IGKC*	10558	78%
*IGJ*	9933	73%
*SDC1*	654	5%

To evaluate our method’s performance against alternative applications, we repeated our analysis using co-occurrence of genes with BTK in Medline abstracts. The assumption is that such an occurrence gives indirect evidence of a biological relationship between the gene and BTK, and roughly speaking, the more co-occurrence we see the stronger the relationship. If we were to observe the same (or a better) level of accuracy at identifying biomarkers with co-occurrence, we would generally prefer it, since it is simpler to compute and more general in its application.

Table 4 shows the validation gene set, along with the number of Medline articles where each gene co-occurs with BTK and a resulting area under the ROC curve of 0.72.

**Table 4:** Co-occurrence ranking of known BTK targets.

Gene	Abstract Co-occurrence count	Rank
*TNF*	49	10
*CCL3*	9	84
*SDC1*	4	186
*IKZF1*	3	241
*IRF4*	3	241
*EGR1*	1	684
*RASGRP1*	1	684
*BCL2A1*	0	7272
*EBI3*	0	7272
*EGR3*	0	7272
*IGJ*	0	7272
*IGKC*	0	7272
*IL4I1*	0	7272

In order to be trustworthy, matrix factorization results need to be explainable in a way that scientists can understand. Our method for doing this relies on finding similar matrix rows containing the connection predicted for the input element. That is, if a connection is predicted to exist downstream of BTK for gene X, then we find another gene whose pattern of connections is most similar to X that also has a connection downstream of BTK. Table 5 depicts the results of this type of analysis for STAT3, a gene predicted to be downstream of BTK. Here we see that STAT1 is very similar to STAT3 in its overall connections and therefore can assess the likelihood that STAT3 will also behave in a similar manner with BTK. The direct evidence for the BTK->STAT3 connection can be displayed to the user based on the sentence(s) that generated that connection in the network.

**Table 5:** Analysis of similar STAT3 matrix rows.

Similar Gene	# of shared connections to STAT3	Total Connections	P value^a^
*STAT1*	273	446	6.22E-17
*JAK1*	102	145	1.70E-11
*IL3*	154	269	1.36E-06
*BCL2L1*	218	409	1.12E-05
*IL7*	100	173	8.04E-05
*NFATC1*	99	173	1.54E-04
*PTPN6*	88	156	7.59E-04
*CD40*	160	311	0.00240641
*PI3*	181	358	0.00343664
*RELA*	231	468	0.00430228
*FLT3*	107	202	0.00449299
*HAVCR2*	36	59	0.00594752
*LYN*	85	159	0.00865486

^a^ *p* values were calculated using a Chi Squared test comparing the number of expected shared connections and (based on individual frequencies) the actual number of shared connections.

## Discussion

We have demonstrated how a directed network of gene-gene relationships extracted from literature can be used to identify novel biomarkers that are not explicitly stated in any of the ingested literature. Sentence parsing of biological relationships enables much richer representation and reasoning than simpler text mining methods based on co-occurrence, which cannot provide crucial information on direct or indirect relationships required to answer most biological questions. Further, as shown in our comparison, co-occurrence among genes of infrequent publication in literature is largely silent when it comes to giving early signals of a potential connection. Using matrix factorization, in contrast, allows us to make the most of what little we know to make our best educated guess at where connections might lie. As we have outlined in the introduction, the effects of BTK inhibition can be direct as well as indirect and this has important implications for the usefulness of experimental data for discovery of proximal or distal pharmacodynamic biomarkers. Here we have shown an example of a WDD workflow that uses direct and indirect relationships and therefore has superior predictive power to other automated literature-based discovery approaches. Compared to an analysis based on direct biological relationships, we also removed the limitations towards proximal biomarker discovery that would exist if we only used direct relationships.

Other computational approaches in network-based discovery, as well as experimental gene expression analysis (GEA) take relationships into account by design but are still suffering from low precision. WDD as used in this work implicitly introduces a tunable compound confidence parameter since the confidence of any resulting predictions will depend on the quality of the underlying data used in the similarity analysis. Whether the experimental result was in vitro or in vivo, whether the result is in the context of a specified disease or in the context of gene mutations are examples of filters that can be used to select only those parts of WDD that are relevant to a specific use case. Additionally, it is important to be able to explain predictions to the scientist and to bring them back to the relevant literature whenever possible, and our method provides a compelling explanation of each prediction based on analogous gene function.

The biggest shortcoming of our method to date is that it lacks a validation mechanism to verify that the hypothesis that is generated is actually likely to be true. Therefore, in the future we hope to incorporate more non-literature information about gene phenotypes into this type of analysis. We will also work to find better methods for filtering the generic network extracted from text based on factors such as species, confidence in the network connection, whether the experimental result was in vitro or in vivo, and whether the network is relevant to a specified disease. We will continue to investigate ways to incorporate real world evidence and supplementary data from publications and to utilize manually curated pathway information (e.g. Kyoto Encyclopedia of Genes and Genomes (KEGG)) to supplement what we extract from text.

This will allow users to develop different biomarker discovery pipelines providing a side-by-side comparison of biomarker rankings with confidence ratings as based on underlying data but also the quality of the prediction. This direct comparison provides users with a technique to prioritize the underlying disease hypotheses based on likelihood, value and risk. To aid this comparison, WDD should be supplemented with known manually curated pathway information (e.g. KEGG) and Gene Expression Omnibus (GEO) data. Such analyses will yield insights that go beyond the literal text of publications, in order to accelerate the pace of discovery, enhance our understanding of disease, and give hope to patients.

## Supporting information

S1 Table Network connections used to generate analysis matrix. (CSV)

S2 Table All genes predicted by WDD to be downstream of BTK. (PDF)

S1 Fig Visual representation of WDD gene network for BTK. Searched entities (inputs) are represented by white circles and connected gene entities by blue circles. Curvature of the connecting arrows indicates reciprocal (curved) or non-reciprocal (straight) relationships. Distance from the searched entity is associated with the number of documents supporting the connection: nearer circles are connected by relationships in more documents than farther circles.
(PDF)

S2 Fig Example sentence-level extractions from WDD BTK gene-gene network. On the left is a summary of gene-gene network connections which can be selected to show sentence-level evidence (on the right) for the relationship connection.
(PDF)

S3 Fig Matrix of WDD sentence-level gene-gene relationships for BTK. BTK is the input gene, and on the left-hand side we see the score for each gene in the matrix factorization result. On the right-hand side are selected columns from the original matrix with yellow dots indicating non-zero values in the original input matrix and darker shading indicating higher floating-point values in the resulting matrix after matrix factorization is applied. This gives the scientist both a value for likelihood of being downstream of BTK, as well as both direct and indirect evidence that generated that value. The direct evidence is sentences in publications that say BTK effects the gene. The indirect evidence is genes that are similar to BTK in their behavior, that show a downstream effect on the gene.
(PDF)

## Abbreviations

ACE: angiotensin-converting enzyme
ALS: Alternating Least Squares Matrix Factorization
ATM: Ataxia-Telangiesctasia mutated
bcl-2: B-cell lymphoma 2
BTK: Bruton’s Tyrosine Kinase
CO: co-occurrence analysis
CS: cross-sentence references
CSF: cerebrospinal fluid
DRD1: Dopamine Receptor D1
ER: entity recognition
ERK: Extracellular receptor kinase
ESG: English slot grammar
FDA: Food and Drug Administration
GEA: gene expression analysis
GEO: Gene Expression Omnibus
GO: Gene Ontology
GPCR: G protein-coupled receptor
GRK: G protein-coupled receptor kinase
GSEA: gene set enrichment analysis
GWA: genome-wide association
KEGG: Kyoto Encyclopedia of Genes and Genomes
LMOD1: Leiomoden 1
MeSH: Medical Subject Headers
MF: matrix factorization
p53: tumor protein 53
PBMC: peripheral blood mononuclear cell
PLCγ2: phospholipase C gamma 2
Plk1: Polo Like Kinase 1
PPI: protein-protein interaction
R&D: research & development
RE: relationship extraction
ROC: area under receiver operating characteristic curve
RSK1: ribosomal S6 kinase 1
siRNA: small interfering Ribonucleic Acid
SLE: systemic lupus erythematosus
STAT: signal transducer and activator of transcription 1
WDD: Watson for Drug Discovery
