# Supplementary material for: Identification of pharmacodynamic biomarker hypotheses through literature analysis with IBM Watson
Source: PLoS One. 2019 Apr 8;14(4):e0214619. doi: 10.1371/journal.pone.0214619 (PMC6453528; doi:10.1371/journal.pone.0214619)
Supplement: S2 Table — (PDF) [file pone.0214619.s002.pdf]

Supplementary Table 1. All genes predicted by WDD to be downstream of BTK.

Gene	Score	Known Downstream to BTK (1 = yes, 0 = no)	Rank
AKT1	0.54458815	1	1
NFKB1	0.5154992	1	2
TNF	0.50323606	0	3
IFNA1	0.4434148	1	4
TP53	0.4385939	1	5
EPHB2	0.4313362	1	6
IL6	0.42688292	1	7
INS	0.41607705	1	8
MAPK8	0.41537482	1	9
STAT3	0.38802144	0	10
IL2	0.3744676	1	11
MAPK3	0.36648414	0	12
BCR	0.35648954	1	13
VEGFA	0.3489667	0	14
IL10	0.34443948	1	15
IL4	0.3414984	0	16
IFNG	0.33224836	0	17
JUN	0.3214404	0	18
CRK	0.31818953	0	19
TGFB1	0.3179956	0	20
EGFR	0.31714404	1	21
CD4	0.3144715	0	22
TLR4	0.31039178	1	23
IL8	0.303452	0	24
STAT5A	0.30185032	0	25
EGF	0.30117995	0	26
IGHV1-2	0.30093542	0	27
CBL	0.29676566	1	28
STAT1	0.2944623	1	29
RAC1	0.28429946	1	30
MTOR	0.28102204	1	31
SYK	0.28000763	1	32
ABL1	0.27899128	1	33
IL17A	0.27572146	0	34
MYC	0.27440986	1	35
CD34	0.27148065	1	36
CSF2	0.2702503	0	37
CD40	0.2699324	1	38

CD8A	0.2696973	0	39
JAK2	0.26840165	0	40
VAV1	0.26694292	0	41
FAS	0.26671404	1	42
BCL2	0.2651292	1	43
AIMP2	0.26320416	0	44
LYN	0.2610977	1	45
GRB2	0.26066443	0	46
PI3	0.25447765	1	47
CASP3	0.25388432	0	48
SRC	0.25309193	0	49
CDKN1A	0.25100806	0	50
CD19	0.2502738	1	51
HLA-E	0.24962842	0	52
FLT3	0.24822447	1	53
MAP2K7	0.24805494	0	54
PTPN11	0.24717925	0	55
CTNNB1	0.24611801	0	56
RAF1	0.24444664	0	57
TRAF6	0.24265029	0	58
IL2RA	0.24227504	0	59
IRF3	0.2422209	0	60
IL1B	0.24073777	0	61
PTPRC	0.2391716	0	62
CD28	0.238719	0	63
IL1	0.23850015	1	64
IL3	0.23684445	1	65
CCL2	0.23642315	0	66
FOXP3	0.23453143	0	67
MMP9	0.23179427	0	68
PTGS2	0.23168208	0	69
ERBB2	0.22457442	0	70
KIT	0.22433446	0	71
ICAM1	0.22395681	0	72
MYD88	0.22302127	1	73
PTK2	0.22269592	1	74
PIK3R2	0.22266148	0	75
PTK2B	0.2222466	0	76
ITGAM	0.21991624	0	77
LCK	0.21970645	0	78
RELA	0.21888448	1	79
TLR2	0.21364053	1	80
CXCL12	0.21334949	1	81

CCND1	0.21065463	0	82
SHC1	0.21063924	0	83
CDC42	0.20803843	0	84
CD40LG	0.20455934	0	85
IGF1	0.20246181	0	86
PTPN6	0.20205273	1	87
ROS1	0.199689	0	88
IL13	0.19910493	0	89
CD14	0.19827025	0	90
ZAP70	0.19809094	0	91
FUT1	0.19794428	0	92
RHOA	0.1964468	0	93
EPO	0.19607337	0	94
TOLLIP	0.19528013	1	95
HGF	0.19252048	0	96
FASLG	0.19051701	0	97
CD44	0.19000398	1	98
AGT	0.18968885	0	99
PXN	0.18957709	0	100
TLR3	0.18887964	1	101
CD86	0.18728264	0	102
BCL2L1	0.18588752	1	103
IL5	0.18559676	1	104
CDH1	0.18501683	0	105
INSR	0.18460935	0	106
GRAP2	0.18396585	0	107
IRS1	0.18396044	0	108
CD38	0.18371755	1	109
JAK1	0.1835913	1	110
ITGAL	0.18219687	0	111
WG	0.18147683	0	112
MAPK1	0.18129331	0	113
FYN	0.1802847	0	114
KDR	0.17939405	0	115
ITGAX	0.17795187	0	116
MAPK9	0.17625216	0	117
IGHM	0.17554535	0	118
CD80	0.17550473	0	119
CSF3	0.17513879	0	120
NR2C2	0.17501968	0	121
PRRT2	0.17496769	0	122
IFNB1	0.17480958	0	123
EBI3	0.17447309	0	124

PLCG1	0.17446895	1	125
CXCR4	0.1732867	1	126
TLR9	0.17306511	1	127
ESR1	0.17293447	0	128
HSPG2	0.1711953	0	129
RASA1	0.17059256	0	130
IGHE	0.1701545	0	131
TBK1	0.17007922	0	132
NOS2	0.16965817	0	133
DDX58	0.16907282	0	134
SPI1	0.1690132	0	135
GORASP1	0.16842063	0	136
CD79A	0.16772267	0	137
IL7	0.16742833	1	138
TRAF2	0.16717038	0	139
KITLG	0.16640957	0	140
DLD	0.16581176	0	141
TRAF3	0.16376314	0	142
BTK	0.16253898	1	143
AR	0.16143122	0	144
STAT6	0.16120383	0	145
IL15	0.16031998	0	146
IRF7	0.16019732	0	147
EPHA8	0.15946506	0	148
TNFSF11	0.15896146	1	149
CD2	0.15854444	0	150
IGHG1	0.15802231	0	151
TNFRSF1A	0.15764478	0	152
PTEN	0.15753315	0	153
CCL5	0.15717553	0	154
STAT5B	0.15669487	0	155
MDM2	0.1554432	0	156
CSF1	0.15339997	0	157
EP300	0.15305163	1	158
ALK	0.15256715	0	159
PML	0.15253812	0	160
RAP1A	0.15193146	0	161
EPOR	0.15139632	0	162
IGF1R	0.15134346	0	163
SNORA62	0.15093958	0	164
INPP5D	0.15077607	0	165
CHUK	0.14978065	0	166
MMP2	0.14925869	0	167

KRAS	0.1486262	0	168
CCR7	0.14726707	0	169
LAT	0.14715032	0	170
SOS1	0.14548004	0	171
CREB1	0.14507158	1	172
HSP90AA1	0.1445843	0	173
IL7R	0.14451976	0	174
CTLA4	0.14371371	0	175
BAX	0.14360239	0	176
SOCS1	0.1433335	0	177
FGF2	0.14304444	1	178
FOS	0.14284849	0	179
CD5	0.1417611	0	180
PLCG2	0.14078629	1	181
ALB	0.14077114	0	182
CAV1	0.14063811	1	183
IL23A	0.14060469	0	184
CSF1R	0.14034283	1	185
KRT20	0.14019628	0	186
CSK	0.13982432	0	187
NFKBIA	0.13913496	0	188
BLNK	0.13882582	1	189
NCAM1	0.13792525	0	190
RUNX1	0.13755767	0	191
BCL6	0.13724433	0	192
REL	0.13699926	0	193
DECR1	0.13686065	0	194
PDCD1	0.13620238	0	195
CXCL10	0.13610427	0	196
ETV6	0.1354229	0	197
CRKL	0.13528852	0	198
LCP2	0.13484478	0	199
RIPK1	0.13447839	0	200
IL18	0.13434123	0	201
CDKN2A	0.13420904	0	202
IL9	0.13382117	0	203
CASP8	0.13375604	0	204
TLR7	0.1333411	1	205
IL6ST	0.13311896	0	206
NLRP3	0.13224909	0	207
NGF	0.13222055	0	208
THPO	0.13019541	0	209
PDGFRB	0.12985067	0	210

CD274	0.1297981	0	211
F3	0.1294971	0	212
SELL	0.12928803	0	213
MSC	0.12907237	0	214
FCGR3A	0.1286662	0	215
PDC	0.12854859	0	216
IL22	0.12845513	0	217
GZMB	0.1275128	0	218
TNFRSF8	0.12694107	0	219
CD69	0.12686634	0	220
ITGB2	0.12685503	0	221
NTRK1	0.126749	0	222
NCK1	0.12649442	0	223
SMAD3	0.1262175	1	224
LEP	0.1261904	0	225
VIM	0.12613457	0	226
SOCS3	0.12573245	0	227
GAB2	0.12553151	0	228
IRAK1	0.12514487	1	229
ITGA2B	0.12480426	0	230
JAK3	0.12472375	0	231
GAB1	0.12463217	0	232
CD27	0.12461409	0	233
CRP	0.12460591	0	234
ARHGEF2	0.12329735	0	235
FCGR1A	0.12279585	0	236
CD33	0.12186863	0	237
NOTCH1	0.12156736	0	238
RB1	0.12115002	0	239
PAX5	0.12108929	0	240
MET	0.12047224	1	241
VCAM1	0.12002453	0	242
GSTK1	0.11921489	0	243
BCL2L11	0.11871125	0	244
TBX21	0.11841855	0	245
RASGRF1	0.11815614	0	246
SPP1	0.11731648	0	247
SP1	0.11716127	0	248
IRF1	0.11695287	0	249
NOD2	0.11694186	0	250
IRF8	0.11656204	0	251
NOS3	0.11619969	0	252
FCER2	0.11617961	0	253

TFRC	0.11536595	0	254
TNFSF13B	0.11532255	0	255
FOXO1	0.1151569	0	256
PRKAA2	0.11479474	0	257
MCL1	0.11464883	0	258
MUC1	0.11456057	0	259
CD22	0.11451316	0	260
MAVS	0.1144932	0	261
PPP2R4	0.1141956	0	262
HRAS	0.11407167	0	263
NFATC1	0.11388299	1	264
TYR	0.11370127	0	265
RPS6KB1	0.11320974	0	266
BIRC5	0.11298367	0	267
CSF3R	0.11203193	0	268
PECAM1	0.11144906	0	269
WAS	0.11133077	1	270
IKBKG	0.11115594	0	271
CTNND1	0.11096997	0	272
RLN3	0.11078851	0	273
IL21	0.11069239	0	274
CD36	0.11038809	0	275
IFIH1	0.11037515	0	276
FCGR2B	0.10952666	1	277
MAPK14	0.10912466	0	278
SOD1	0.10881761	0	279
HSPA4	0.10857546	0	280
APCS	0.10856585	0	281
HCK	0.10839995	0	282
PRL	0.10821551	0	283
IRF4	0.10727329	0	284
POLDIP2	0.10706557	0	285
BMP2	0.10686481	0	286
CASP1	0.10605595	0	287
TPO	0.10537594	0	288
EZR	0.10536481	0	289
STAT4	0.1052753	0	290
TYK2	0.10501675	0	291
ABCB1	0.10490487	1	292
TICAM1	0.10478853	0	293
MAP3K5	0.10425807	0	294
MLL	0.10377791	0	295
CDKN1B	0.10287464	0	296

PSMD9	0.10266794	0	297
MAP3K14	0.10257404	0	298
PTPN1	0.10254838	0	299
FAM49B	0.1019937	0	300
ITK	0.10199186	1	301
GATA1	0.10198014	0	302
ISYNA1	0.10189252	0	303
MYB	0.10185169	0	304
MPO	0.10178772	0	305
MBP	0.10109842	0	306
CTTN	0.10094791	0	307
RUNX2	0.10042723	0	308
PLEK	0.10037734	1	309
TEC	0.10018311	1	310
PPARG	0.09995874	0	311
TNFAIP3	0.09974206	0	312
MAP2K1	0.09955397	0	313
APOE	0.09927501	0	314
PAK1	0.09914032	0	315
NFKB2	0.0989966	0	316
NFE2L2	0.09882832	0	317
NPM1	0.0984012	0	318
PDLIM7	0.09804539	0	319
TH1L	0.09795225	0	320
CISH	0.09779207	0	321
FLT3LG	0.09765891	0	322
HDAC	0.09760127	0	323
IGH	0.09748204	0	324
TRIM63	0.0974322	1	325
MPL	0.09728344	0	326
EDN1	0.09680755	0	327
TERT	0.09651176	0	328
ICOS	0.09619811	0	329
KLRK1	0.09619228	0	330
GATA3	0.09615743	0	331
HMGB1	0.09588342	0	332
CYBB	0.09488519	0	333
ISG20	0.09469623	0	334
AHR	0.09435068	0	335
PRDM1	0.09412389	0	336
HPGDS	0.09404156	0	337
CD7	0.09354919	0	338
CD244	0.09328464	0	339

SND1	0.09288479	1	340
CD276	0.09234878	0	341
RAG2	0.09229694	0	342
OPN1SW	0.09174199	0	343
CDH5	0.0914538	0	344
AICDA	0.09115531	0	345
SMAD2	0.09092972	0	346
TET2	0.09077006	0	347
CLEC7A	0.09033206	0	348
DOK1	0.08957174	0	349
EIF2AK2	0.08880292	0	350
NR3C1	0.08873069	0	351
IRF5	0.08845416	0	352
ITGAE	0.08823121	0	353
PYCARD	0.08764651	0	354
SLC2A4	0.08743361	0	355
UBASH3B	0.0873485	0	356
BCL10	0.08692619	0	357
BCAR1	0.08641321	0	358
MIR155	0.08627002	0	359
DDX41	0.08563491	1	360
BECN1	0.08516987	0	361
PRKCD	0.08515456	0	362
RELB	0.08514959	0	363
CARD11	0.08507566	1	364
CREBBP	0.08491711	0	365
IL27	0.08381063	0	366
WDTC1	0.08378379	0	367
CYCS	0.08340251	0	368
LYZ	0.08318842	0	369
DHX58	0.08311306	0	370
CNTN2	0.08310894	0	371
MAP4K1	0.08297931	0	372
HIF1A	0.0825908	0	373
FGFR1	0.08227874	0	374
BIRC2	0.08188752	0	375
GSK3B	0.08178402	0	376
JUND	0.0817457	0	377
PAG1	0.08148518	0	378
IKZF1	0.08136331	1	379
GLI2	0.08082863	0	380
PMEL	0.08064893	0	381
BRAF	0.08060235	0	382

TNFRSF9	0.08050341	0	383
EGR1	0.08038781	0	384
TAT	0.08009603	0	385
MMP1	0.08001456	0	386
CEBPA	0.07951484	0	387
CD1A	0.07920189	0	388
ALDH9A1	0.07917011	0	389
ACTA2	0.07914447	0	390
ELK1	0.07910233	0	391
GH1	0.07904661	0	392
SMAD4	0.0789137	0	393
IKBKB	0.07861196	0	394
ANPEP	0.07783859	0	395
IARS	0.07768926	0	396
TYROBP	0.07759761	0	397
IL33	0.07759442	0	398
HNF4A	0.07741937	0	399
TSLP	0.07726087	0	400
BIRC3	0.07690778	0	401
RIPK2	0.07664932	0	402
FCGRT	0.07609064	0	403
JUNB	0.07603148	0	404
CXCL1	0.07555895	0	405
FANCB	0.07544189	0	406
CR2	0.07524768	0	407
SNORD12C	0.07509243	0	408
PARP1	0.07501233	0	409
RTEL1	0.07479762	0	410
EVPL	0.07475095	0	411
TCF3	0.0745292	0	412
JAG1	0.07440444	0	413
PDK1	0.07439016	0	414
TAM	0.07420589	0	415
ADIPOQ	0.07387074	0	416
IGHD	0.07350098	0	417
POU5F1	0.07338609	0	418
LAG3	0.0733472	0	419
SPN	0.07292277	0	420
CCL22	0.07288049	0	421
MAP2K4	0.07274049	0	422
IL4R	0.0726101	0	423
RAC2	0.07258748	0	424
SIRPA	0.07257308	0	425

IL3RA	0.07253343	0	426
WT1	0.07252823	0	427
PLD2	0.0725091	0	428
PPA1	0.07245307	0	429
RBL2	0.07237442	0	430
LBR	0.0723162	0	431
CXCR5	0.07226152	0	432
IL11	0.0716054	0	433
EZH2	0.07119566	0	434
FOXO3	0.07107612	0	435
LAMP1	0.07091213	0	436
CALR	0.07066313	1	437
MBTPS1	0.07049929	0	438
HTL	0.07042182	0	439
ASXL1	0.07033905	0	440
SERPINE1	0.07026155	0	441
STAT2	0.07001629	0	442
TLR1	0.06997858	0	443
HDAC1	0.06991316	0	444
CEBPB	0.06989902	0	445
CD68	0.06983625	0	446
BDNF	0.06977465	0	447
VAV2	0.06975426	0	448
MAP3K1	0.06959723	0	449
CBLB	0.06953983	0	450
DCTN4	0.06938981	0	451
NRAS	0.06936494	0	452
CR1	0.06919055	0	453
DYM	0.06916831	0	454
ERBB3	0.06914055	0	455
ANXA2	0.06906501	0	456
RARA	0.06885932	0	457
LIF	0.06866344	0	458
HAVCR2	0.06854224	1	459
NANOG	0.06838957	0	460
CD83	0.06819395	0	461
PDGFRA	0.06778958	0	462
FPR1	0.06773836	0	463
CD1C	0.0674618	0	464
SERPINB3	0.06745414	0	465
PSMA7	0.06743185	0	466
PIM1	0.06685821	0	467
STK11	0.06664847	0	468

CRLF2	0.06637629	0	469
SMAD1	0.06625412	0	470
HAMP	0.06595016	0	471
RAB5A	0.06589858	0	472
INHBE	0.06583617	0	473
CD79B	0.0655871	0	474
CNTNAP1	0.06555276	0	475
PROM1	0.06550144	0	476
BMP4	0.06546763	0	477
RUNX1T1	0.06530224	0	478
THM	0.06511968	0	479
NR4A1	0.06509146	0	480
ERBB4	0.06499659	0	481
CXCR3	0.06495385	0	482
MAF	0.06493721	0	483
HRH4	0.06481667	0	484
TGFBR2	0.06467466	0	485
GJA1	0.06467449	0	486
THY1	0.06445184	0	487
LANCL1	0.064017	0	488
TLR5	0.06386808	0	489
KIAA0101	0.06382578	0	490
VTLG	0.06377555	0	491
IL1A	0.06363565	0	492
TEK	0.06359179	0	493
C5AR1	0.06358794	0	494
CD24	0.06336969	0	495
FUT4	0.0633197	0	496
PREB	0.06284493	0	497
MUC5AC	0.06280753	0	498
SELE	0.0627929	0	499
CCL4	0.06252965	0	500
PTH	0.06229422	0	501
MAP1LC3A	0.06213516	0	502
ARHGEF1	0.06171858	0	503
IFNAR1	0.06111366	0	504
MITF	0.06104334	0	505
CCL3	0.06086525	0	506
PLAU	0.0608402	0	507
TNFRSF4	0.06074132	0	508
SUMO1	0.06060288	0	509
NR3C2	0.06029001	0	510
RAPGEF1	0.06027836	0	511

PPARA	0.06016952	0	512
NM	0.06012209	0	513
FOXM1	0.060047	0	514
TNK2	0.0597467	0	515
CD81	0.05971959	0	516
HLA-DRB4	0.05963961	0	517
AXL	0.05963154	0	518
FES	0.05959513	0	519
LPL	0.05957531	0	520
TNFRSF13C	0.05929933	0	521
IGHG3	0.05907955	0	522
BRD2	0.05907446	0	523
NR0B2	0.0590267	0	524
ELANE	0.05902483	0	525
PTK6	0.05890045	0	526
ITGA4	0.05887443	0	527
MCS	0.05886562	0	528
CCR5	0.05883726	0	529
TNFSF10	0.05871616	0	530
LGALS1	0.05869626	0	531
PELP1	0.05858394	0	532
ARF1	0.05852293	0	533
TF	0.05850461	0	534
RAG1	0.05847704	0	535
S100A8	0.05845285	0	536
MALT1	0.05844462	0	537
EIF3A	0.05842827	0	538
TNFRSF1B	0.05833099	0	539
HOXA9	0.05817146	0	540
VWF	0.05811067	0	541
FYB	0.05806434	0	542
CSF2RA	0.0578699	0	543
TNFRSF11B	0.05781313	0	544
ARF6	0.05749848	0	545
SF3B1	0.05733888	0	546
CCL17	0.05730423	0	547
ETS1	0.05725237	1	548
XBP1	0.05704748	1	549
FADD	0.05690381	0	550
CCL21	0.05685424	0	551
NCF1	0.05682374	0	552
ARHGAP1	0.05658241	0	553
ID2	0.05634878	0	554

IRS2	0.05630069	0	555
S100B	0.05622045	0	556
RIPK3	0.05618733	0	557
CFTR	0.05614621	0	558
RRAS	0.05603962	0	559
ID1	0.05584666	0	560
FCGR2A	0.05578852	0	561
MAP3K3	0.05577747	0	562
IL6R	0.05569122	0	563
GIF	0.05568481	0	564
CYLD	0.05567918	0	565
ACAD8	0.05549493	0	566
PRKCA	0.05527759	0	567
SOX9	0.05524892	0	568
OSM	0.05523653	0	569
TLR8	0.05488419	1	570
NGFR	0.05467207	0	571
CD1D	0.05463364	0	572
WNK1	0.05463107	0	573
NPHS1	0.05459476	0	574
NCL	0.05456649	0	575
CD55	0.05441628	0	576
SRF	0.05437363	0	577
ANXA5	0.05431419	0	578
MMP14	0.05408052	0	579
NPY6R	0.05407015	0	580
BRCA1	0.05404267	0	581
CD59	0.05395612	0	582
CCR2	0.05395556	0	583
SELP	0.05389216	0	584
ARHGEF7	0.05378194	0	585
ATXN1	0.05374469	0	586
SOS2	0.05369117	0	587
GHR	0.05366918	0	588
SOCS2	0.05366778	0	589
CD63	0.05356064	0	590
CUX1	0.05355895	0	591
POMC	0.05349191	0	592
CD48	0.05345861	0	593
CCL20	0.05319229	0	594
FLT1	0.05315686	0	595
CCL11	0.05309851	0	596
CASP9	0.05307865	0	597

PRKD1	0.05304235	0	598
TIRAP	0.05303868	1	599
DOCK1	0.05284304	0	600
RUNX3	0.05273415	0	601
EPHA3	0.05256379	0	602
CD53	0.05253822	0	603
RET	0.0524932	0	604
WASL	0.05235669	0	605
MYOD1	0.05234635	0	606
DNTT	0.05231753	0	607
LDLR	0.05197618	0	608
LRP1	0.05197002	0	609
PRSS27	0.05178596	0	610
HSPB3	0.05174864	0	611
IL2RB	0.05170089	0	612
EOMES	0.05155768	0	613
ACTR2	0.05153345	0	614
SIRT1	0.05152487	0	615
TRAF1	0.0514374	0	616
IRF9	0.05140604	0	617
CXCL13	0.0513902	0	618
TWIST1	0.05133088	0	619
CHRDL1	0.05128941	0	620
HSPB1	0.05127223	0	621
SMAD7	0.05122246	0	622
PDCD1LG2	0.05110248	0	623
PGR	0.05099607	0	624
ABI1	0.05096042	0	625
YAP1	0.05094289	0	626
SLC2A1	0.05092507	0	627
TRADD	0.05084294	0	628
ANXA6	0.05071418	0	629
CTGF	0.0506517	0	630
MST1	0.05061238	0	631
SREBF1	0.05058296	0	632
KHDRBS1	0.0504307	0	633
SLAMF1	0.05019054	0	634
SQSTM1	0.05017234	0	635
TAL1	0.05008945	0	636
ILK	0.05000247	0	637
TNFSF4	0.04994926	0	638
NR2E3	0.04987775	0	639
CXCL9	0.04986784	0	640

SETD2	0.04985059	0	641
CAT	0.04983402	0	642
HCCS	0.04983159	0	643
AHSA1	0.049705	0	644
APP	0.04964163	0	645
HCLS1	0.04962508	0	646
TGFBR1	0.04961952	0	647
ST2	0.04919424	0	648
MSN	0.04917648	0	649
CCR6	0.04906806	0	650
F2RL1	0.04902528	0	651
SNRPE	0.04880796	0	652
CFLAR	0.04868009	0	653
SDC1	0.04865464	0	654
MMP3	0.04858523	0	655
TBP	0.04851817	0	656
EPHA2	0.04823963	0	657
ITGA2	0.04815902	0	658
ITIH4	0.04814423	0	659
PLAT	0.04805426	0	660
PIK3CG	0.04801115	0	661
LAP	0.04800441	0	662
HSP90B1	0.04793783	0	663
PBX1	0.04782153	0	664
ENG	0.0476272	0	665
SH2B3	0.04752505	0	666
IFIT1	0.04741747	0	667
FLVCR1	0.04731228	0	668
WDR77	0.04711965	0	669
UGCG	0.04711559	1	670
AKAP13	0.04709613	0	671
THBS1	0.04707782	0	672
IL12RB1	0.04706839	0	673
CD72	0.0469964	0	674
ROCK1	0.04697854	0	675
NCR1	0.04685467	0	676
GLI1	0.04674391	0	677
MAP2K6	0.04665037	0	678
NEDD9	0.04661224	0	679
CSE1L	0.04650261	0	680
UMOD	0.04649084	0	681
NCF2	0.04648766	0	682
FGFR2	0.04643761	0	683

TSPO	0.04640612	0	684
SOX2	0.04629143	0	685
GATA4	0.04618526	0	686
KLRG1	0.0460172	0	687
PSMC6	0.04581879	0	688
HMOX1	0.04577462	0	689
VCL	0.04576104	0	690
CD247	0.04552367	0	691
SLC9A1	0.04540941	0	692
CARD9	0.04540506	0	693
ALOX5	0.04516915	0	694
EBF1	0.04513938	0	695
CEBPZ	0.0450621	0	696
IRAK4	0.04498551	0	697
ATM	0.04497464	0	698
IV	0.04495072	0	699
IL2RG	0.04490807	0	700
LGALS3	0.04479694	0	701
RIMS2	0.0447459	0	702
CXCL2	0.0447288	0	703
PTPRU	0.04470374	0	704
APC	0.044671	0	705
PGC	0.04459652	0	706
E2F1	0.04451338	0	707
IL1RN	0.0445117	0	708
MME	0.04447879	0	709
GATA2	0.04424109	0	710
LMO2	0.044194	0	711
VEGFC	0.04399165	0	712
RGS6	0.04397796	0	713
HSPA5	0.04394	0	714
CD1B	0.04393866	0	715
ELF4	0.04380482	0	716
EIF2S1	0.04377228	0	717
KLRB1	0.04362937	0	718
FGR	0.04357445	0	719
CDH2	0.04352994	0	720
CD9	0.0435134	0	721
CDK5R1	0.04346973	0	722
WNT3A	0.04346203	0	723
NOX4	0.04345731	0	724
IGAN	0.04331459	0	725
TXN	0.04324419	0	726

IGKV1D-39	0.04324069	0	727
SELPLG	0.04321713	0	728
LCN2	0.04312921	0	729
CD58	0.04308725	0	730
IL24	0.04304415	0	731
FLI1	0.04299453	0	732
PCNA	0.04296472	0	733
INPPL1	0.04293345	0	734
BGLAP	0.04286401	0	735
MMP13	0.04281501	0	736
FER	0.04277746	0	737
CD226	0.04269217	0	738
MCF2	0.04264941	0	739
ATP6AP2	0.04256715	0	740
CD47	0.04253081	0	741
SH2B1	0.04251732	0	742
ASAP1	0.04246954	0	743
DPP4	0.04238696	0	744
GAPDH	0.04237813	0	745
AFF1	0.04237764	0	746
HIST2H2BE	0.04223213	0	747
XIAP	0.0421664	0	748
MX1	0.04199877	0	749
CLEC4C	0.04194749	0	750
TIMP1	0.04191167	0	751
LTA	0.041866	0	752
CANX	0.04180849	0	753
EFS	0.04171091	0	754
TCF4	0.04157063	0	755
GP6	0.04156894	0	756
STAP1	0.04155741	1	757
PCS	0.04147581	0	758
TP73	0.04147032	0	759
LY96	0.04146119	0	760
APOA1	0.04144177	0	761
EPCAM	0.04138111	0	762
BLK	0.04132035	0	763
ITGA5	0.0412324	0	764
CTSB	0.04115233	0	765
CDC25C	0.04107207	0	766
ADAM11	0.04102955	0	767
IL17F	0.04098183	0	768
BCHE	0.04097943	0	769

HSF1	0.04093973	0	770
NEDD4	0.04093149	0	771
ZFP36	0.04089104	0	772
PIK3R1	0.04086525	0	773
MATN1	0.04065381	0	774
CCND3	0.04064643	0	775
DUSP1	0.04062499	0	776
IL5RA	0.04057752	0	777
GFAP	0.04056263	0	778
GP1BA	0.04051674	0	779
SH2D1A	0.04048054	1	780
RAB11A	0.04039953	0	781
IFNA2	0.04025653	0	782
LEF1	0.04021466	0	783
FIP1L1	0.03991894	0	784
GPI	0.03984276	0	785
EBP	0.03984244	0	786
NFATC2	0.03981118	0	787
SLAMF6	0.03957823	0	788
EMD	0.03952454	0	789
GRB10	0.0394914	0	790
QRSL1	0.03944939	0	791
PTPN2	0.03936786	0	792
F2	0.03936118	0	793
KLRD1	0.03930626	0	794
CEACAM6	0.03928654	0	795
LGALS4	0.03925457	0	796
KIAA0319	0.03910823	0	797
CKAP4	0.0390571	0	798
FRS2	0.03905232	0	799
MLC1	0.03896608	0	800
SPRY2	0.03892851	0	801
ABCA1	0.03889358	0	802
RDX	0.03888578	0	803
PARD3	0.03885362	0	804
SKAP1	0.03875661	0	805
A4GALT	0.03866857	0	806
B2M	0.0385722	0	807
GNB2L1	0.03854255	0	808
HTT	0.038495	0	809
NOX1	0.03840716	0	810
PDIK1L	0.0383357	0	811
CYP2B6	0.03827361	0	812

GRB7	0.03824702	0	813
OXA1L	0.03819187	0	814
HDAC6	0.03813054	0	815
HCST	0.0381145	0	816
MVD	0.03806817	0	817
AXIN1	0.0380449	0	818
ITGB3	0.03802535	0	819
TNFRSF10B	0.03788567	0	820
PAK2	0.03783537	0	821
WIPF1	0.03783092	1	822
PLG	0.03783039	0	823
KIR3DL1	0.03766622	0	824
CDKL1	0.03763178	0	825
NCOR1	0.03742671	0	826
CD3E	0.03741317	0	827
TRIM33	0.03732079	0	828
MEIS1	0.0372843	0	829
SHH	0.03721462	1	830
RSAD2	0.0370834	0	831
DEFB4A	0.03705769	0	832
LILRB3	0.03702825	0	833
DNM2	0.03695259	0	834
GSN	0.03682615	0	835
PLD1	0.03675169	0	836
CEACAM1	0.03675159	0	837
NCOR2	0.03667237	0	838
TNFRSF18	0.03651143	0	839
MOG	0.0365095	0	840
CD99	0.03650364	0	841
TMEM173	0.03639708	0	842
SH3KBP1	0.03636994	0	843
RIN2	0.036349	0	844
PSIP1	0.03629665	0	845
PIAS1	0.03608492	0	846
DOK2	0.03607545	0	847
CCL19	0.03607147	0	848
AGFG1	0.03602286	0	849
TAB1	0.03572515	0	850
BAK1	0.03563241	0	851
HSPA8	0.03559876	0	852
PRLR	0.03559783	0	853
BCL3	0.03557615	0	854
HBB	0.03553383	0	855

IL37	0.03535681	0	856
DNMT3A	0.03534757	0	857
CCND2	0.03532739	0	858
MIF	0.0353158	0	859
SF1	0.03530993	0	860
ERVW-1	0.0352945	0	861
MMRN1	0.03524823	0	862
TRIM28	0.03522781	0	863
COL2A1	0.03520936	0	864
NF1	0.03517271	0	865
ISG15	0.03514203	0	866
SLC9A3R1	0.03510677	0	867
MN1	0.03496498	0	868
WNT5A	0.03495409	0	869
CNBP	0.03492068	0	870
DIABLO	0.03489006	0	871
AFP	0.03487831	0	872
SERPINA5	0.03485706	1	873
EIF4EBP1	0.03483846	0	874
TNFSF9	0.03466508	0	875
RABGEF1	0.03436859	0	876
RHOB	0.0343498	0	877
MLLT3	0.0343156	0	878
B3GAT1	0.03430149	0	879
SNCA	0.03423799	0	880
NTN1	0.03420878	0	881
TJP1	0.03416761	0	882
TRIM21	0.03413866	0	883
CD70	0.03393146	0	884
NRP1	0.03387978	0	885
BMX	0.03381173	0	886
ARHGAP35	0.03377854	0	887
RAP1B	0.03375294	0	888
SLC4A1	0.03367429	0	889
IL1R1	0.03359618	0	890
CHAF1A	0.03346142	0	891
TNFRSF13B	0.03343654	0	892
GTF2I	0.03341335	1	893
ENPP3	0.03338158	0	894
F2R	0.03333482	0	895
LY9	0.03332496	0	896
IL25	0.03330267	0	897
VASP	0.03329328	0	898

CLEC6A	0.03318599	0	899
CIITA	0.03315097	0	900
SLAMF7	0.03311755	0	901
IRF2	0.03308567	0	902
SLURP1	0.03308415	0	903
DEF6	0.03306944	0	904
IQGAP1	0.03299607	0	905
TAB2	0.032926	0	906
ADAM17	0.03291087	0	907
MT2A	0.03287333	0	908
MLLT4	0.03286561	0	909
KLF1	0.03282652	0	910
CCL16	0.0328227	0	911
C3	0.0327747	0	912
CXCR1	0.03269221	0	913
INPP5K	0.032599	1	914
TNFRSF10A	0.03241235	0	915
DAPP1	0.0322799	0	916
TRIM25	0.0321415	0	917
HSPD1	0.03213626	0	918
CDC123	0.03212917	0	919
PTS	0.03212776	0	920
ABCG2	0.03211928	0	921
GCG	0.03202563	0	922
IGKV4-1	0.03199191	0	923
CD180	0.03190888	0	924
MYOG	0.03177957	0	925
GIT1	0.03176096	0	926
KLK3	0.03175837	0	927
MAP3K4	0.03165425	0	928
TRIM69	0.031638	0	929
NOTCH2	0.03162805	1	930
TRAF3IP2	0.03160389	0	931
MAG	0.03153118	0	932
BLZF1	0.03152169	0	933
EGR2	0.03147659	0	934
HIST2H2AA3	0.03143009	0	935
ZBTB16	0.03141617	0	936
HES1	0.03138676	0	937
NOLC1	0.03130491	0	938
PLSCR1	0.03124327	0	939
NCOA1	0.03124128	0	940
ATF2	0.03118188	0	941

GTF3A	0.03111869	0	942
PDLIM4	0.03105419	0	943
EPHA1	0.03101861	0	944
HT	0.03101684	0	945
IL32	0.03098234	0	946
AKT2	0.03086104	0	947
U2AF1	0.0308557	0	948
WASF2	0.03084785	0	949
RAB1B	0.03079608	0	950
KLRC1	0.03072775	0	951
UBE2I	0.0306485	0	952
MAPK7	0.03062441	0	953
MAPK10	0.03046016	0	954
STAP2	0.03044168	0	955
FCGR3B	0.03043458	0	956
MCAM	0.03029905	0	957
SKP1	0.03024001	0	958
ARAF	0.03023893	0	959
IL21R	0.03023271	0	960
MYH11	0.03015894	0	961
ACTB	0.03011805	0	962
NPC1	0.03005735	0	963
IL23R	0.03002235	0	964
EPHB1	0.03002131	0	965
PSEN1	0.03001979	0	966
NAT9	0.02997443	0	967
PRKCQ	0.02992526	0	968
SEMA4D	0.02984354	0	969
CTNNA1	0.02977225	0	970
VPREB1	0.02971355	1	971
VAV3	0.02969835	0	972
NKX2-5	0.02967942	0	973
CD163	0.02966789	0	974
CD2AP	0.02966254	0	975
LMNA	0.0296498	0	976
SMPD1	0.02962858	0	977
FGF1	0.02961904	0	978
PIN1	0.02948345	0	979
PIK3CD	0.02947439	0	980
LRP6	0.02944833	0	981
NES	0.02940225	0	982
CBFB	0.02939388	0	983
SH3BP2	0.02936181	0	984

FLT4	0.02931973	0	985
CLPS	0.02931146	0	986
TFF1	0.02930744	0	987
RALGDS	0.02928282	0	988
NFAT5	0.0292378	0	989
UBE2N	0.02923267	0	990
HGS	0.02921864	0	991
KMT2A	0.02920274	0	992
BMP7	0.02919437	0	993
HDC	0.02912715	0	994
ACVRL1	0.02910773	0	995
PPARGC1A	0.02908035	0	996
RASGRP1	0.02897279	0	997
MKKS	0.0288413	0	998
PTGS1	0.02883133	0	999
MUM1	0.02878141	0	1000
AREG	0.02875986	0	1001
TREM2	0.02874785	0	1002
PF4	0.02874478	0	1003
FGFR3	0.02873556	0	1004
NTRK2	0.02872896	0	1005
TGFB1I1	0.0287213	0	1006
ICOSLG	0.02870902	0	1007
CYP1A1	0.02863229	0	1008
MAP3K8	0.02861843	0	1009
GPLD1	0.02856328	0	1010
ARHGAP4	0.02854787	0	1011
PTPN4	0.02848257	0	1012
ATN1	0.02843776	0	1013
MIP	0.0284324	0	1014
NOD1	0.02843183	0	1015
BSG	0.02840313	0	1016
LTB4R	0.02839265	0	1017
RAB7B	0.02836417	0	1018
GDNF	0.02832283	0	1019
TCL1B	0.02815476	0	1020
AIRE	0.0281335	0	1021
KAT5	0.02811485	0	1022
SGK1	0.02802668	0	1023
HOXA10	0.02795221	0	1024
IL17C	0.02792722	0	1025
DPEP1	0.02783296	0	1026
FASN	0.02782446	0	1027

EIF2AK3	0.02781664	0	1028
EIF4E	0.02780663	0	1029
SIGLEC1	0.02768644	0	1030
SRSF2	0.02765267	0	1031
CYBA	0.02761571	0	1032
FKBP4	0.02755122	0	1033
PRKDC	0.02753588	0	1034
CD82	0.0275131	0	1035
SCT	0.02744458	0	1036
EPHB4	0.02743698	0	1037
SMN1	0.02742327	0	1038
LCS1	0.02734882	0	1039
CDK4	0.02717528	0	1040
POU2F1	0.02711143	0	1041
RPS6	0.0271047	0	1042
ETV5	0.02710366	0	1043
TBX1	0.02708256	0	1044
CX3CR1	0.02706925	0	1045
HLCS	0.02703438	0	1046
RBFOX2	0.0270224	0	1047
IFI27	0.02699751	0	1048
RPS6KA2	0.02696756	0	1049
SKP2	0.02695888	0	1050
EXOSC10	0.02691407	1	1051
TRAF5	0.02688606	0	1052
MATK	0.02681572	0	1053
ERN1	0.02676291	0	1054
MLN	0.02671855	0	1055
KLF4	0.02663669	0	1056
TP63	0.02660413	0	1057
DOK3	0.02658961	0	1058
NT5E	0.02658914	0	1059
KRT8	0.02653307	0	1060
TARP	0.02649292	0	1061
ULK1	0.0264763	0	1062
GLUL	0.02646568	0	1063
HLA-G	0.02644508	0	1064
LILRB4	0.02636194	0	1065
DCLK3	0.0263398	0	1066
RHOG	0.0262978	0	1067
EFNA5	0.02629629	0	1068
ANGPTL2	0.02620065	0	1069
VHL	0.02619932	0	1070

DDX53	0.02616452	0	1071
ING1	0.02609123	0	1072
AGXT	0.02607849	0	1073
IKBKE	0.0260657	0	1074
OCLN	0.0260648	0	1075
IRS4	0.02605956	0	1076
BTRC	0.02603527	0	1077
IL31RA	0.02603454	0	1078
FCER1A	0.02603105	0	1079
CDK9	0.02598307	0	1080
PDPN	0.02585364	0	1081
PRDM10	0.02582653	0	1082
CEACAM5	0.02578039	0	1083
LPA	0.02576134	0	1084
GFI1B	0.02568163	0	1085
MECOM	0.02562907	0	1086
GBA	0.02560598	0	1087
MAP3K11	0.02555198	0	1088
CXCR2	0.02553123	0	1089
SETBP1	0.02553071	0	1090
DLG4	0.02550419	0	1091
DAB1	0.02547643	0	1092
LRP2	0.02541518	0	1093
DVL2	0.02536882	0	1094
CAPNS1	0.02536	0	1095
PRH2	0.02534114	0	1096
SLC17A5	0.02503894	0	1097
CX3CL1	0.02503893	0	1098
TICAM2	0.02503614	0	1099
SAFB	0.02503009	0	1100
MERTK	0.02493842	0	1101
FCER1G	0.02486645	0	1102
TGFA	0.02486645	0	1103
PIKFYVE	0.02484927	1	1104
DAPK1	0.024847	0	1105
MAP2K3	0.02476731	0	1106
CBLC	0.02471672	0	1107
COPS5	0.02470774	0	1108
EFNB1	0.02470367	0	1109
IFI44	0.02464105	0	1110
FAT4	0.02463388	0	1111
IFNGR1	0.02461799	0	1112
UBE2D1	0.02455476	0	1113

DAP	0.02451632	0	1114
CTAG1B	0.02448633	0	1115
TNFRSF12A	0.02439126	0	1116
PRTN3	0.02436811	0	1117
ELMO1	0.02435993	0	1118
NR1I2	0.02432289	0	1119
SMARCA1	0.02431575	1	1120
GNLY	0.02430181	0	1121
TSC2	0.02427868	0	1122
BST2	0.02427216	0	1123
RHOH	0.02426428	0	1124
SUMO3	0.02424798	0	1125
NFE2	0.0242413	0	1126
SPHK1	0.02422738	0	1127
SORBS1	0.02422195	0	1128
PLXNB1	0.02421253	0	1129
CCR4	0.02414779	0	1130
S100A9	0.0241298	0	1131
PMP22	0.02412255	0	1132
VTN	0.02411281	0	1133
BANF1	0.02410964	0	1134
ENTPD1	0.02409858	0	1135
CD300A	0.02404297	0	1136
LAT2	0.02401837	0	1137
SDC4	0.02400379	0	1138
PGF	0.02399321	0	1139
LAD1	0.02398607	0	1140
ILF3	0.02398552	0	1141
NCOA3	0.02396277	0	1142
CLEC1B	0.0239381	0	1143
NOP2	0.02392904	0	1144
TIMM8A	0.0239265	0	1145
FRTS	0.02386686	0	1146
ACTN4	0.02384717	0	1147
WTS	0.02383352	0	1148
MT1JP	0.02382993	0	1149
STK3	0.0238006	0	1150
TCHP	0.02378459	0	1151
KRT14	0.023782	0	1152
IKZF3	0.0237445	0	1153
ANGPT1	0.02371638	0	1154
SMO	0.02364012	0	1155
MYCN	0.02362804	0	1156

NBN	0.02359412	0	1157
MEFV	0.02350928	0	1158
PODXL	0.02344449	0	1159
MAP3K2	0.02343107	0	1160
SLC6A8	0.02337463	1	1161
PDGFB	0.02333012	0	1162
DAB2IP	0.02330098	0	1163
DLL4	0.02326869	0	1164
PDCD6IP	0.0232373	0	1165
POR	0.02320478	0	1166
ANC	0.02317319	0	1167
GREM1	0.02316421	0	1168
FKBP1A	0.02316409	0	1169
DUSP6	0.02313726	0	1170
DSG3	0.02306577	0	1171
FLOT1	0.02305344	0	1172
IL17D	0.0230331	0	1173
DNMT1	0.02301651	0	1174
FOXP1	0.02301476	0	1175
IFIT3	0.022949	0	1176
AIM2	0.0229481	0	1177
PIK3CA	0.02292804	0	1178
PMAIP1	0.0228985	0	1179
APOB	0.02289331	0	1180
PLA2G4A	0.02286603	0	1181
GCKR	0.02285943	0	1182
SRA1	0.02283103	0	1183
BACH2	0.02281964	0	1184
MEF2A	0.02281521	0	1185
SWAP70	0.02272631	0	1186
EVA1C	0.0227197	0	1187
RAB4A	0.02271591	0	1188
SGSM3	0.02267196	0	1189
PLAUR	0.02259164	0	1190
ERCC3	0.02255954	0	1191
MAPK8IP3	0.02254182	0	1192
FH	0.02253118	0	1193
CCDC88A	0.02251805	0	1194
NLRX1	0.02250904	0	1195
PIM2	0.02238732	0	1196
PPP5C	0.0223762	0	1197
RAP2A	0.02237546	0	1198
IGHV@	0.02235441	0	1199

DAXX	0.02232246	0	1200
LILRB1	0.0222518	0	1201
CIB1	0.02223979	0	1202
WNK4	0.02223541	0	1203
RRBP1	0.02218214	0	1204
ARHGEF4	0.02217026	0	1205
KIR2DL1	0.0221492	0	1206
CTRL	0.02213755	0	1207
LILRB2	0.02211669	0	1208
TDGF1	0.02207763	0	1209
EIF4G2	0.02205865	0	1210
DMD	0.02205181	0	1211
NLRC5	0.0220515	0	1212
SPIB	0.02204794	0	1213
RAD51	0.02203681	0	1214
RARS	0.02201692	0	1215
PRKCZ	0.02200248	0	1216
CDK2	0.02199346	0	1217
PLA2G1B	0.02191107	0	1218
NCR3	0.02188255	0	1219
F8	0.02186919	0	1220
DHFR	0.02185126	0	1221
MYOCD	0.02182249	0	1222
PKM	0.02182072	0	1223
IDO1	0.02181904	0	1224
CSNK1A1	0.02180188	0	1225
TSC1	0.0217919	0	1226
IL17RA	0.02176801	0	1227
SPRED2	0.02171753	0	1228
PAX3	0.02168189	0	1229
NOV	0.02165385	0	1230
TAS2R38	0.02164714	0	1231
EPHA4	0.02160466	0	1232
NSUN5	0.02160271	0	1233
IL9R	0.02153438	0	1234
HDAC3	0.02152943	0	1235
GNPTAB	0.02151434	0	1236
MMD	0.02149991	0	1237
WDR48	0.0214924	0	1238
TRAC	0.02145284	0	1239
UBR5	0.02140059	0	1240
PTGES3	0.02139537	0	1241
IL29	0.02134821	0	1242

TCF7	0.02132709	0	1243
IGHA1	0.02130891	0	1244
NTRK3	0.02129059	0	1245
HRASLS	0.0212196	1	1246
ARHGEF28	0.02117858	0	1247
FLNA	0.02114843	0	1248
TRIP6	0.02113045	0	1249
PIP4K2C	0.02109807	1	1250
RHOQ	0.02107105	0	1251
LGR5	0.02106381	0	1252
UBE2T	0.02105484	0	1253
TRIM27	0.02103312	0	1254
FARP2	0.02100736	0	1255
LIFR	0.0210031	0	1256
CD46	0.02100183	0	1257
HLA-A	0.02096424	0	1258
SMOC1	0.02096319	0	1259
LPXN	0.02089354	0	1260
DVL1	0.02088286	0	1261
BAP1	0.02084549	0	1262
GAS6	0.02082348	0	1263
ATG5	0.02081151	0	1264
ZHX2	0.02079855	0	1265
HSPA1A	0.02076149	0	1266
HDAC2	0.02075217	0	1267
KRT7	0.02072298	0	1268
P4HB	0.02070827	0	1269
MAP2K2	0.0207034	0	1270
APOBEC3B	0.02067713	0	1271
RNPC3	0.02066163	0	1272
ANLN	0.02064079	0	1273
IL12A	0.02062886	0	1274
ARHGAP17	0.02059241	0	1275
HP	0.02055644	0	1276
ITGAV	0.02049191	0	1277
ZEB1	0.02048337	0	1278
PCM1	0.02042525	0	1279
ACCS	0.02034773	0	1280
TBCE	0.02030226	0	1281
IL27RA	0.02029887	0	1282
CD300C	0.02029437	0	1283
THBD	0.02028281	0	1284
P2RX7	0.02027069	0	1285

ENAH	0.02026496	0	1286
H2AFX	0.02024588	0	1287
KLF2	0.02021839	0	1288
KRT5	0.02017425	0	1289
PKD1	0.02012307	0	1290
PITX1	0.02012237	0	1291
WNT2	0.02011624	0	1292
RPS6KA3	0.02007341	0	1293
PPIA	0.01999602	0	1294
CCR9	0.01994652	0	1295
PSMC1	0.01994539	0	1296
TNFRSF14	0.01989349	0	1297
GBP1	0.01985226	0	1298
SORBS2	0.01984518	0	1299
ACP1	0.0198316	0	1300
NME1	0.0198224	0	1301
SGCB	0.01982158	0	1302
OAS3	0.01980396	0	1303
ID3	0.01979294	0	1304
IL19	0.01978766	0	1305
PDLIM5	0.01978441	0	1306
GPNMB	0.01976227	0	1307
PRB1	0.01974406	0	1308
DHX16	0.01973797	0	1309
IGK	0.01973236	0	1310
S100P	0.01972838	0	1311
PTPN22	0.01970714	0	1312
KRT18	0.01970377	0	1313
MLLT1	0.01969922	0	1314
HLA-DOA	0.01969484	0	1315
RANGAP1	0.01966249	0	1316
IL31	0.01964269	0	1317
F10	0.01961445	0	1318
MAPKAPK2	0.01953507	0	1319
BAIAP2	0.01952718	0	1320
TBCC	0.01952547	0	1321
CD207	0.01952228	0	1322
FXYD5	0.0195068	0	1323
CLEC4D	0.01946458	0	1324
CD200	0.01941061	0	1325
SUMO2	0.01938349	0	1326
EIF2S3	0.01936221	0	1327
CTNND2	0.01935494	0	1328

CDC37	0.01934707	0	1329
KLRAP1	0.01927363	0	1330
RACGAP1	0.01927027	0	1331
FRS3	0.01925492	0	1332
MLKL	0.01923288	0	1333
GRB14	0.01923022	0	1334
SULT1E1	0.01921271	0	1335
CDKN2B	0.01921118	0	1336
SRSF5	0.01919308	0	1337
SLC35B2	0.01919118	0	1338
SEMA3A	0.01914499	0	1339
MYF5	0.01912287	0	1340
CAMP	0.01907497	0	1341
UBE2L3	0.01907121	0	1342
CD52	0.01906884	0	1343
PLK1	0.01906605	0	1344
EML4	0.0190442	0	1345
NLRC4	0.01903275	0	1346
EEA1	0.01900945	0	1347
LTF	0.01899585	0	1348
RNF19A	0.01898677	0	1349
RALA	0.01893217	0	1350
RIPK4	0.0189214	0	1351
ECD	0.01889443	0	1352
MLLT10	0.01888415	0	1353
ACO1	0.01887508	0	1354
CEACAM3	0.01886222	0	1355
NRSN1	0.01886128	0	1356
UBE2B	0.01884531	0	1357
TNFSF13	0.01882553	0	1358
ARHGDIB	0.01880215	0	1359
CEACAM7	0.01878828	0	1360
RPGR	0.01878521	0	1361
EFNA2	0.01877795	0	1362
MAP2K5	0.01873487	0	1363
TAB3	0.01871829	0	1364
KLF5	0.01871528	0	1365
DNM1L	0.01869028	0	1366
MAP3K13	0.01868453	0	1367
HLA-DQB1	0.01866496	0	1368
APOBEC3G	0.01865009	0	1369
ASCC1	0.01863333	0	1370
SLIT2	0.01862169	0	1371

TAP1	0.01860675	0	1372
BANK1	0.01858518	0	1373
KRT19	0.01858063	0	1374
TNNT2	0.01856702	0	1375
PEA15	0.01856464	0	1376
YWHAQ	0.01856114	0	1377
CDK1	0.01855637	0	1378
TNFSF8	0.01855297	0	1379
FGF8	0.01855111	0	1380
SHB	0.01854693	0	1381
SHC3	0.01851892	0	1382
PIK3CB	0.01847624	0	1383
TRIM22	0.01845875	0	1384
CSRP3	0.0184575	0	1385
CYTH1	0.01844545	0	1386
KIRREL	0.01843877	0	1387
CD200R1	0.01839657	0	1388
C6ORF25	0.01838384	0	1389
AIF1	0.01838066	0	1390
EFNA1	0.01837676	0	1391
TYMP	0.01836728	0	1392
VDR	0.01835774	0	1393
NCOA2	0.01831031	0	1394
PARD6A	0.01830606	0	1395
LTB	0.01829852	0	1396
DAG1	0.01828384	0	1397
TIA1	0.01825545	0	1398
CADM1	0.01823168	0	1399
LCP1	0.01821505	0	1400
SIGLEC7	0.01819689	0	1401
MEF2C	0.0181932	0	1402
NRG1	0.01818569	0	1403
KSR1	0.01818046	0	1404
THOC5	0.01817224	0	1405
ANP32A	0.01813704	0	1406
CYMD	0.01812682	0	1407
LEPREL2	0.01812084	0	1408
MIR15B	0.01811353	0	1409
CGB5	0.01811021	0	1410
PTPRE	0.01808224	0	1411
RAB40B	0.01807626	0	1412
SCD	0.01806824	0	1413
KCNMA1	0.01803493	0	1414

AIMP1	0.01803141	0	1415
TIAM1	0.01802028	0	1416
RGS4	0.01801889	0	1417
GRDX	0.01800362	0	1418
VPS45	0.01796302	0	1419
IFIT2	0.0179566	0	1420
ICAM3	0.0179532	0	1421
PIK3AP1	0.01795308	1	1422
PAX7	0.01792427	0	1423
IFT122	0.01792159	0	1424
CXCL11	0.01791147	0	1425
PHC1	0.01786645	0	1426
BHLHE23	0.01785103	0	1427
GDF15	0.01784889	0	1428
PGP	0.01783486	0	1429
PPFIBP1	0.01783185	0	1430
WASF3	0.01782425	0	1431
PPP2CA	0.01777543	0	1432
SDC2	0.01776608	0	1433
PSMB11	0.01775039	0	1434
SGPL1	0.01768802	0	1435
BTLA	0.01766748	0	1436
DDX17	0.01766062	0	1437
MGAT5	0.0176519	0	1438
RNASE3	0.01760897	0	1439
RBX1	0.01760024	0	1440
RASGRP3	0.0175398	0	1441
GZMA	0.01753337	0	1442
RMDN3	0.01750204	0	1443
EPS8	0.01749886	0	1444
IGF2	0.0174579	0	1445
CD177	0.01744763	0	1446
BMF	0.01744379	0	1447
EGR3	0.017414	0	1448
PHB2	0.0174064	1	1449
RNF135	0.01740135	0	1450
IFNAR2	0.01735968	0	1451
CLTC	0.01734093	0	1452
TG	0.01732003	0	1453
SUCLG2	0.01730916	0	1454
GCLC	0.0173041	0	1455
ABCD1	0.01730263	0	1456
CYP27A1	0.01725893	0	1457

CDCP1	0.01721939	0	1458
AGRN	0.01721462	0	1459
RXRA	0.01718528	0	1460
HDAC8	0.01718423	0	1461
PRIM2	0.01718213	0	1462
ADAR	0.01715565	0	1463
KNG1	0.01709489	0	1464
DLL1	0.01709484	0	1465
EDA	0.01708503	0	1466
RAB3GAP1	0.01699635	0	1467
IL10RA	0.01697058	0	1468
HSP90B2P	0.01694432	0	1469
NR5A1	0.01694026	0	1470
GOLGB1	0.01693589	0	1471
SUCLG1	0.01691822	0	1472
SLC25A3	0.01682727	0	1473
DNM1	0.01682437	0	1474
SECTM1	0.01678344	0	1475
MSR1	0.0167617	0	1476
ATR	0.01673298	0	1477
NRIP1	0.01672037	0	1478
SMAD5	0.01670476	0	1479
MVP	0.01669745	0	1480
TCF12	0.01669316	0	1481
RORC	0.01667035	0	1482
ARHGDIA	0.01666245	0	1483
FGFR4	0.01665519	0	1484
KRIT1	0.01663897	0	1485
AURKA	0.01663029	0	1486
NOL3	0.01662185	0	1487
KCNJ1	0.01661182	0	1488
MAGEC2	0.01660282	0	1489
CPB1	0.01658635	0	1490
IFNL3	0.01658316	0	1491
USP7	0.01656462	0	1492
PSMA5	0.0164864	0	1493
PTP4A3	0.01648337	0	1494
SHARPIN	0.01645135	0	1495
CH25H	0.01644663	0	1496
IL15RA	0.01644596	0	1497
PTPRO	0.01644221	0	1498
CHDM	0.01643825	0	1499
FCAR	0.0164127	0	1500

CTSG	0.01641044	0	1501
ATG7	0.01637819	0	1502
APPL1	0.01636809	0	1503
MIR125A	0.0163315	0	1504
BATF	0.0163059	0	1505
KIF5B	0.01628451	0	1506
PTPRA	0.01628073	0	1507
TREM1	0.01626109	0	1508
DDX3X	0.01625994	0	1509
TLR6	0.01625593	0	1510
CPSF4	0.01622823	0	1511
OCA2	0.01621	0	1512
OSCAR	0.01620949	0	1513
DOCK8	0.01620605	0	1514
ROR1	0.01615424	0	1515
BATF3	0.01615217	0	1516
RPS3	0.01615121	0	1517
TOM1L1	0.01614968	0	1518
HAVCR1	0.01614947	0	1519
RCAN1	0.0161328	0	1520
ITSN1	0.01610452	0	1521
GRAP	0.01609634	0	1522
IL1RL2	0.01607792	0	1523
KIR3DL2	0.01607376	0	1524
TNC	0.01601754	0	1525
RARRES3	0.01601112	0	1526
MIR31	0.01598991	0	1527
CALCOCO2	0.01598623	0	1528
EREG	0.01598027	0	1529
OPTN	0.01592832	0	1530
SLA2	0.01592236	0	1531
PRKD2	0.01591469	0	1532
XBP1P1	0.01588513	0	1533
GOLGA4	0.01587617	0	1534
HSH2D	0.01587593	0	1535
NMT1	0.01587505	0	1536
APOH	0.01586767	0	1537
PRKCB	0.01582736	1	1538
ASPG	0.01579096	0	1539
IGLL1	0.01575705	1	1540
KCNA3	0.01574231	0	1541
NEDD4L	0.01572381	0	1542
HNRNPA1	0.01572099	0	1543

PELI1	0.01570949	0	1544
ABP1	0.0156985	0	1545
MMP7	0.01568183	0	1546
NIN	0.01564395	0	1547
NT5C2	0.01563833	0	1548
IFNA17	0.01562727	0	1549
GRIP1	0.01562692	0	1550
TFR2	0.01558077	0	1551
RETN	0.01557146	0	1552
NKX2-1	0.01555623	0	1553
ARHGEF12	0.01555067	0	1554
HNRNPC	0.01552679	0	1555
ESR2	0.01552275	0	1556
DDX21	0.01551272	0	1557
ORAI1	0.01550745	0	1558
FUT7	0.0154906	0	1559
CEBPE	0.01546207	0	1560
TCRB	0.01546019	0	1561
EIF4B	0.01545615	0	1562
TANK	0.01544912	0	1563
CTSL1	0.0154469	0	1564
PTGDR	0.01541733	0	1565
SPG21	0.01540405	0	1566
CYP3A4	0.01539179	0	1567
RBPJ	0.01538977	0	1568
ADRB2	0.01538885	0	1569
AGO2	0.01537963	0	1570
SKAP2	0.01536958	0	1571
FSTL1	0.01535784	0	1572
NDRG1	0.01533948	0	1573
NFIL3	0.01532383	0	1574
CFH	0.01530361	0	1575
CRBN	0.01525635	0	1576
ITGB4	0.01523427	0	1577
ADAM22	0.01522806	0	1578
HNMT	0.01521135	0	1579
CNR1	0.01520644	0	1580
TLR10	0.01519983	0	1581
MAFD2	0.01515872	0	1582
LPAR2	0.01513429	0	1583
PRAM1	0.01513368	0	1584
FLG	0.01512956	0	1585
TNFRSF25	0.01512514	0	1586

RPTOR	0.01509713	0	1587
ARHGEF26	0.01508423	0	1588
SM1	0.01505718	0	1589
GSTP1	0.0150548	0	1590
PRKAA1	0.01504896	0	1591
STUB1	0.0150483	0	1592
RHO	0.01504822	0	1593
EIF2B1	0.01504815	0	1594
PEBP1	0.01504355	0	1595
FCGR2C	0.01503096	0	1596
UBTF	0.01502756	0	1597
GNA12	0.01499513	0	1598
LSP1	0.01497898	0	1599
SARS	0.01497826	0	1600
TAGLN	0.01494341	0	1601
PCK2	0.01492395	0	1602
GAB3	0.01491846	0	1603
OAS1	0.01491103	0	1604
RANP1	0.0149106	0	1605
HPP1	0.01489446	0	1606
TMPRSS6	0.01488767	0	1607
CBFA2T3	0.0148709	0	1608
NXF1	0.01482513	0	1609
PRDX5	0.01482178	0	1610
ALDH1A1	0.01479111	0	1611
DCSTAMP	0.01477298	0	1612
EPX	0.01477217	0	1613
NCR2	0.01475111	0	1614
VCP	0.01474149	0	1615
PPP1R9B	0.01472111	0	1616
EIF3K	0.01471854	0	1617
S1PR1	0.0147138	0	1618
RPAIN	0.01468908	0	1619
ITCH	0.01467868	0	1620
BMI1	0.01466215	0	1621
HLA-DRB5	0.01464744	0	1622
TYRO3	0.01464659	0	1623
EVI5	0.01462396	0	1624
NSG1	0.01462327	0	1625
PPP1R12A	0.01462061	0	1626
CCL27	0.01461295	0	1627
CLEC9A	0.01458817	0	1628
IL16	0.01458742	0	1629

TOM1	0.0145855	0	1630
LTBR	0.01455618	0	1631
NR1H4	0.01455506	0	1632
SORBS3	0.01454769	0	1633
ESAM	0.01454201	0	1634
CYP3A	0.01452942	0	1635
SOCS7	0.01452176	0	1636
KEAP1	0.01449597	0	1637
PRMT5	0.01448788	0	1638
NFATC3	0.01448082	0	1639
XPO1	0.01448058	0	1640
DPYSL2	0.01447486	0	1641
PTPRK	0.01446485	0	1642
SKIL	0.01445056	0	1643
TAX1BP1	0.01445043	0	1644
RPL26	0.0144466	0	1645
DUSP4	0.0144389	0	1646
GFI1	0.01443245	0	1647
CASP2	0.01443171	0	1648
TRIB2	0.01442708	0	1649
PRF1	0.01441921	0	1650
IBSP	0.01441625	0	1651
RRAS2	0.01441101	0	1652
DNAJB7	0.01440682	0	1653
TSG101	0.01440246	0	1654
HOXA7	0.01439044	0	1655
PIAS3	0.01437918	0	1656
MPZL1	0.01433867	0	1657
MIB2	0.01432031	0	1658
NEU3	0.01429982	0	1659
OSTC	0.01428112	0	1660
HBM	0.01425922	0	1661
RAE1	0.014256	0	1662
FOSB	0.01424698	0	1663
MSTN	0.01423892	0	1664
TNNI3	0.01423775	0	1665
RAN	0.01421069	0	1666
SLC5A7	0.01419544	0	1667
FGD1	0.01417292	0	1668
TIE1	0.01417008	0	1669
HK1	0.0141527	0	1670
SIGLEC9	0.01414251	0	1671
ECSIT	0.01410012	0	1672

IFNR	0.01408008	0	1673
ABCC2	0.01403805	0	1674
ULBP2	0.01401458	0	1675
OSMR	0.01400919	0	1676
SLC9A3	0.01400629	0	1677
ELF3	0.01399459	0	1678
PBRM1	0.01397009	0	1679
UNC93B1	0.01396022	0	1680
PVR	0.01393509	0	1681
SCARF1	0.01393108	0	1682
RANBP2	0.01393099	0	1683
TRPC6	0.01392179	0	1684
FOLH1	0.01392107	0	1685
CYP7A1	0.0139183	0	1686
HIPK2	0.01386711	0	1687
PLN	0.01384733	0	1688
YBX1	0.01383635	0	1689
PTPN12	0.01383492	0	1690
TCN2	0.01382071	0	1691
MIR21	0.01381729	0	1692
CASP7	0.01376232	0	1693
GAL	0.01376058	0	1694
SLC3A2	0.01375988	0	1695
GATA6	0.01375058	0	1696
TCN1	0.0137352	0	1697
SPG7	0.01373309	0	1698
SHC4	0.01372764	0	1699
CDR3	0.01369731	0	1700
EXOC2	0.01364075	0	1701
SAV1	0.01361092	0	1702
CLIC4	0.01360992	0	1703
COL9A3	0.01359585	0	1704
ATXN3	0.01357646	0	1705
SMAD6	0.01356744	0	1706
MYO6	0.01355982	0	1707
RORA	0.01354444	0	1708
TRIM31	0.0135185	0	1709
USP18	0.01350653	0	1710
IFNA4	0.01349801	0	1711
ARHGEF25	0.01347207	0	1712
CYP2D6	0.01345409	0	1713
NF2	0.01345218	0	1714
SFPQ	0.01343549	0	1715

CD209	0.01341897	0	1716
ATF6	0.01340575	0	1717
MAP1LC3B	0.01340361	0	1718
CTHRC1	0.01340352	0	1719
HDAC4	0.0133989	0	1720
SLCO6A1	0.01339721	0	1721
PDXP	0.01337056	0	1722
UBE3A	0.01332502	0	1723
CD6	0.01331286	0	1724
KAT2B	0.01329199	0	1725
RPN1	0.01328185	0	1726
TBCA	0.01327223	0	1727
YY1	0.01326568	0	1728
CUL2	0.01326217	0	1729
MIR590	0.01325601	0	1730
HAP1	0.01324938	0	1731
VAMP8	0.01322861	0	1732
BCAR3	0.01322447	0	1733
CAMK2G	0.01322109	0	1734
PTGER2	0.01321222	0	1735
NPHS2	0.01320421	0	1736
BAALC	0.01319474	0	1737
PLCB1	0.01316779	0	1738
RGS14	0.0131616	0	1739
MIR628	0.01316031	0	1740
CSN1S1	0.01314728	0	1741
FHL2	0.01310562	0	1742
PRX	0.01308063	0	1743
TBC1D4	0.01306067	0	1744
CCR1	0.01305246	0	1745
PIEZO2	0.01305194	0	1746
PAWR	0.0130225	0	1747
SSBP3	0.01301067	0	1748
BNIP1	0.0130076	0	1749
HBE1	0.01300372	0	1750
IL18R1	0.01297271	0	1751
ZFPM1	0.01295094	0	1752
MAFG	0.0129498	0	1753
TMED7	0.0129466	0	1754
FGF7	0.01293287	0	1755
MBD2	0.01292657	0	1756
DXS435E	0.01291743	0	1757
RNF11	0.01290805	0	1758

GNAZ	0.01289639	0	1759
FGF10	0.01288765	0	1760
PKLR	0.01288712	0	1761
DLAT	0.01287582	0	1762
AVP	0.01287489	0	1763
IRAK3	0.01287373	0	1764
ROR2	0.01286836	0	1765
SMURF2	0.01285264	0	1766
IFI35	0.0128482	0	1767
ALCAM	0.01284615	0	1768
MIR27A	0.01282854	0	1769
BACE1	0.01282784	0	1770
PPARGC1B	0.01282237	0	1771
PLCB2	0.0128161	0	1772
CDK20	0.01281298	0	1773
IGFBP7	0.01281077	0	1774
TNFRSF11A	0.01280832	0	1775
IFI16	0.01280453	0	1776
TGM2	0.01280183	0	1777
RASSF2	0.01279346	0	1778
AKTIP	0.0127818	0	1779
FAP	0.01278013	0	1780
SLC22A1	0.01274671	0	1781
IDH2	0.01274419	0	1782
ATF1	0.01273345	0	1783
GADD45A	0.01271223	0	1784
ARHGAP24	0.01270106	0	1785
PSTPIP1	0.01269476	0	1786
RNF2	0.01267902	0	1787
ZMYND11	0.01266829	0	1788
RBCK1	0.01264767	0	1789
CAMK2B	0.01264346	0	1790
CDKL2	0.01263723	0	1791
2-Sep	0.01261567	0	1792
STARD13	0.01260132	0	1793
CAND1	0.01256129	0	1794
RNF41	0.01253283	0	1795
ZMYM2	0.01252371	0	1796
CIC	0.01250608	0	1797
MIR24-1	0.01250052	0	1798
DUOX1	0.01249995	0	1799
FURIN	0.01249515	0	1800
NACA	0.0124921	0	1801

KRT12	0.01249209	0	1802
NLK	0.01242281	0	1803
PSC	0.01241216	0	1804
GCM1	0.01239969	0	1805
NBR1	0.01238208	0	1806
SLC20A1	0.01236972	0	1807
SMARCA4	0.01236464	0	1808
C1QBP	0.01236168	0	1809
CMA1	0.01235843	0	1810
IL22RA1	0.01235385	0	1811
CSN2	0.01235292	0	1812
DAB2	0.01234193	0	1813
F7	0.01233101	0	1814
PPP1R42	0.01232296	0	1815
G3BP1	0.01228665	0	1816
DUSP3	0.01226424	0	1817
BTG2	0.01225234	0	1818
MIR223	0.01224836	0	1819
RAC3	0.01224771	0	1820
MAEA	0.01224673	0	1821
TSC22D3	0.01223597	0	1822
BRD4	0.01222218	0	1823
SENP8	0.01222039	0	1824
NOXA1	0.01221628	0	1825
MMACHC	0.01221106	0	1826
HTRA2	0.0122106	0	1827
NOX3	0.01220736	0	1828
PTPRN2	0.01220542	0	1829
ATP2A2	0.0122043	0	1830
CALD1	0.01220303	0	1831
LPP	0.01220264	0	1832
LBP	0.01218063	0	1833
PADI3	0.01218007	0	1834
PLXND1	0.01217508	0	1835
AIFM1	0.01217055	0	1836
BRF1	0.01216525	0	1837
APOBEC3A	0.01216044	0	1838
PIK3C2B	0.01215497	0	1839
COX7A2L	0.01215102	0	1840
SH3PXD2A	0.01211849	0	1841
EIF2S2	0.01211738	0	1842
HDAC7	0.01211654	0	1843
ATL1	0.01206699	0	1844

IGKV1D-35	0.01206665	0	1845
ABL2	0.01204487	0	1846
NS2	0.01204433	0	1847
LRP5	0.01203574	0	1848
IGKV6D-21	0.01202448	0	1849
PPCS	0.01201008	0	1850
ANXA1	0.01200834	0	1851
RHOU	0.01200832	0	1852
ACAT1	0.01199685	0	1853
FGF5	0.01196784	0	1854
JBS	0.01196173	0	1855
UBE2D2	0.01195479	0	1856
TRB	0.01195146	0	1857
PRNP	0.01195129	0	1858
USP36	0.01193998	0	1859
HUNK	0.01191872	0	1860
NLRP1	0.01191805	0	1861
LYST	0.01189793	0	1862
PSTPIP2	0.01188725	0	1863
TACSTD2	0.01188008	0	1864
FOSL2	0.01186645	0	1865
FCRL3	0.01186065	0	1866
PDPK1	0.01186011	0	1867
TIAM2	0.0118593	0	1868
SOX6	0.0118593	0	1869
MRC1	0.01185718	0	1870
IFITM1	0.01184501	0	1871
PLCB3	0.01183002	0	1872
PSG5	0.01182068	0	1873
TM4SF5	0.01180177	0	1874
HNF1A	0.01176179	0	1875
MAPK11	0.01175499	0	1876
MON2	0.01174734	0	1877
ART1	0.01174438	0	1878
MYOC	0.01174035	0	1879
CPAT1	0.01171923	1	1880
CA1	0.01170849	0	1881
SFRP1	0.01170794	0	1882
KIR2DL4	0.01169637	0	1883
ARHGEF6	0.01168652	0	1884
ETV2	0.01168486	0	1885
KRT85	0.01167423	0	1886
LPHN2	0.01167313	0	1887

BCRP2	0.01165202	0	1888
FGF4	0.01164538	0	1889
PMVK	0.01164405	0	1890
DBNL	0.01164284	0	1891
PRAME	0.01162477	0	1892
ZBTB7B	0.01160489	0	1893
MCF2L	0.01160158	0	1894
HBG2	0.01160037	0	1895
DUSP5	0.01159798	0	1896
TSHR	0.01157248	0	1897
ADRBK1	0.01157118	0	1898
MANEA	0.01155909	0	1899
AKR1B1	0.01155737	0	1900
RIEG2	0.011538	0	1901
MRAS	0.01153596	0	1902
IFITM3	0.01152366	0	1903
GP9	0.01150874	0	1904
HEY1	0.01150123	0	1905
PDX1	0.01149777	0	1906
PKN1	0.01148896	0	1907
OLFM4	0.01146312	0	1908
UROD	0.01144271	0	1909
CUL3	0.01142727	0	1910
HUWE1	0.01142343	0	1911
MX2	0.01141951	0	1912
APBB1	0.01140213	0	1913
DSPP	0.01139563	0	1914
NR1I3	0.01139557	0	1915
AKAP8	0.01138837	0	1916
COG2	0.0113827	0	1917
ATF3	0.01138074	0	1918
H3F3AP4	0.01138039	0	1919
STXBP1	0.01137866	0	1920
TXNDC15	0.01137784	0	1921
SYNPO	0.01135359	0	1922
RALBP1	0.01135143	0	1923
PANX1	0.01134068	0	1924
NCKAP1	0.0113364	0	1925
PKN2	0.01132796	0	1926
FRK	0.01132509	0	1927
ZBTB17	0.01131632	0	1928
RPS19	0.01131256	0	1929
CCL26	0.01130626	0	1930

UGT1A6	0.01130626	0	1931
EGLN3	0.01128272	0	1932
CYP4F3	0.01128171	0	1933
GMPS	0.01127471	0	1934
PTPRCAP	0.01125518	0	1935
MAP3K7	0.01124757	0	1936
RND1	0.01124564	0	1937
NUP62	0.01124188	0	1938
PTPN13	0.01123993	0	1939
HACE1	0.01123968	0	1940
SEC23IP	0.01122972	0	1941
DNMBP	0.01122317	0	1942
BAG1	0.01121908	0	1943
ERC1	0.01120478	0	1944
CLU	0.01120344	0	1945
TBX5	0.01120117	0	1946
CSNK2A1	0.01119361	0	1947
TPX2	0.01118518	0	1948
USP25	0.01116724	0	1949
SERPINA1	0.01115382	0	1950
APBB1IP	0.01114805	0	1951
HNRNPUL1	0.01113964	0	1952
IER3	0.01112932	0	1953
DDX6	0.01112354	0	1954
AKAP1	0.0111137	0	1955
BMP6	0.01111308	0	1956
TYRP1	0.01110976	0	1957
MED1	0.01110248	0	1958
MIR219-1	0.0110865	0	1959
CD93	0.01106028	0	1960
KAT6A	0.01104061	0	1961
SMURF1	0.01103065	0	1962
DDX5	0.01101535	0	1963
NR5A2	0.01100977	0	1964
TIMD4	0.01100466	0	1965
MAFB	0.0109951	0	1966
RAP1GDS1	0.01099281	0	1967
CLCF1	0.01099068	0	1968
4-Sep	0.01098867	0	1969
CSPG4	0.01098514	0	1970
CYP27B1	0.01097857	0	1971
CCL25	0.0109521	0	1972
KLF6	0.01095125	0	1973

LY75	0.01093359	0	1974
ETFA	0.0109284	0	1975
ALS2	0.01088474	0	1976
RASA2	0.01087354	0	1977
APAF1	0.0108554	0	1978
JAG2	0.01084784	0	1979
LAMP2	0.01083082	0	1980
HPSE	0.0108229	0	1981
BP1	0.01080632	0	1982
FOSL1	0.01077341	0	1983
RAPH1	0.01076738	0	1984
LYL1	0.01075048	0	1985
KLF13	0.01072376	0	1986
TTF1	0.01072318	0	1987
CBFA2T2	0.01071894	0	1988
IGL	0.01071392	0	1989
TXK	0.01069934	0	1990
SDHB	0.01066975	0	1991
SREBF2	0.01066546	0	1992
AVIL	0.01066213	0	1993
PGGT1B	0.01066202	0	1994
ARHGAP10	0.01065686	0	1995
PLEKHO1	0.01065383	0	1996
RBBP7	0.01064844	0	1997
DYSF	0.01063356	0	1998
PPP4C	0.01062401	0	1999
USP8	0.01062198	0	2000
HMBS	0.01061826	0	2001
IL17RB	0.01059578	0	2002
HLN2	0.01058171	0	2003
CLEC5A	0.01057654	0	2004
PRR9	0.01057625	0	2005
ABCG5	0.01057607	0	2006
MB21D1	0.0105681	0	2007
SLC22A6	0.01056808	0	2008
NFIB	0.01056539	0	2009
OLR1	0.01056351	0	2010
HEMGN	0.01054714	0	2011
UBE2V1	0.01054547	0	2012
PIP5K1C	0.01052571	0	2013
PRDM16	0.01051572	0	2014
DAZAP2	0.01050411	0	2015
SLK	0.01047378	0	2016

SLI1	0.01046906	0	2017
ABCG1	0.01045117	0	2018
TPM3	0.01044613	0	2019
ABCB11	0.01044434	0	2020
ASAP3	0.01043101	0	2021
PTPRN	0.010425	0	2022
KRT16	0.0104235	0	2023
LRPPRC	0.01041675	0	2024
TNFRSF17	0.01040968	0	2025
AHCYL1	0.01040843	0	2026
PGAM5	0.01039004	0	2027
MKNK1	0.01038425	0	2028
IL1RAP	0.01038246	0	2029
VIL1	0.01038157	0	2030
CTSK	0.01037268	0	2031
RAB1A	0.01036447	0	2032
CXCR7	0.01035692	0	2033
ITGAD	0.01032954	0	2034
TMF1	0.01032688	0	2035
MYLK2	0.01032126	0	2036
ARHGEF11	0.01031542	0	2037
MED15	0.01030746	0	2038
MGMT	0.01030034	0	2039
ANGPT2	0.01028684	0	2040
POF1B	0.01027805	0	2041
OAT	0.01027351	0	2042
NCK2	0.01026048	0	2043
GABPA	0.01025875	0	2044
EDARADD	0.01024742	0	2045
GTF2B	0.01023634	0	2046
CEACAM8	0.01021926	0	2047
CALM1	0.0102171	0	2048
EEF1A1	0.01020594	0	2049
EFNB2	0.01019429	0	2050
MAL	0.01017589	1	2051
SH2D1B	0.01017114	0	2052
SKI	0.01016855	0	2053
FAF1	0.01016192	0	2054
WSN	0.01015703	0	2055
YWHAE	0.01014875	0	2056
MUC16	0.0101461	0	2057
G6PC2	0.01014522	0	2058
SIRT2	0.0101424	0	2059

GPR183	0.0101312	0	2060
HSD17B6	0.01010303	0	2061
IGSF9	0.01009524	0	2062
CYTH2	0.01009124	0	2063
PRG2	0.0100857	0	2064
MED28	0.01007912	0	2065
APOA2	0.01007146	0	2066
RIN1	0.01007105	0	2067
RAB32	0.01006607	0	2068
MAP4K2	0.01006569	0	2069
NR2F1	0.01006478	0	2070
ADM	0.01004672	0	2071
PRAP1	0.01001851	0	2072
SCARB1	0.0100139	0	2073
MIR196B	0.01000968	0	2074
MST1R	0.0100037	0	2075
CDX2	0.01000253	0	2076
EWSR1	0.00998866	0	2077
TFAP2A	0.00998046	0	2078
GABARAPL2	0.00996847	0	2079
PPP6C	0.0099678	0	2080
GUCY2D	0.00996524	0	2081
HDAC5	0.00996032	0	2082
SDHD	0.00995856	0	2083
PREX2	0.00994186	0	2084
IDH1	0.009938	0	2085
GRN	0.00993429	0	2086
DHDDS	0.00992779	0	2087
NUMA1	0.00991906	0	2088
NAAA	0.00991869	0	2089
CTPS1	0.00991527	0	2090
DUOX2	0.00991377	0	2091
MBTPS2	0.00990867	0	2092
GADD45G	0.00990519	0	2093
STAMBP	0.00990341	0	2094
SLC25A1	0.00989865	0	2095
PARVA	0.00988972	0	2096
WISP3	0.00987096	0	2097
GNAQ	0.00986514	0	2098
SP100	0.00985474	0	2099
POU1F1	0.00983247	0	2100
NOG	0.00982525	0	2101
SP7	0.00982403	0	2102

FPGS	0.00981179	0	2103
SPRED1	0.00980096	0	2104
NAB2	0.00980035	0	2105
SFTPC	0.00979609	0	2106
ARNT	0.00978749	0	2107
PTPRF	0.00978402	0	2108
HOXB4	0.00977982	0	2109
NLRP12	0.00977961	0	2110
PROK2	0.00976233	0	2111
PIWIL4	0.00976039	0	2112
PLEC	0.00975181	0	2113
PCBP4	0.00974144	0	2114
BCOR	0.0097314	0	2115
PSMD7	0.00971985	0	2116
PTN	0.00971571	0	2117
MLL2	0.00970419	0	2118
SH3BP5	0.00969137	0	2119
TKTL1	0.00967902	0	2120
DSG2	0.0096751	0	2121
PHLDA1	0.00967301	0	2122
ITM2C	0.00967296	0	2123
ARID2	0.00967047	0	2124
PTPRT	0.00964443	0	2125
DLK1	0.00963681	0	2126
GP5	0.00963148	0	2127
SRSF1	0.00960886	0	2128
DMP1	0.00960192	0	2129
CP	0.0095742	0	2130
PPR1	0.00957158	0	2131
UGT1A1	0.00956731	0	2132
DHRS2	0.00956432	0	2133
EPHA5	0.00956203	0	2134
P2RX4	0.00955552	0	2135
RGS1	0.00955033	0	2136
PACS2	0.00954705	0	2137
CD248	0.00954692	0	2138
CBX5	0.00953261	0	2139
LASP1	0.00952895	0	2140
ADAM10	0.00952845	0	2141
1-Mar	0.0095197	0	2142
PPP1R14A	0.00951749	0	2143
CUBN	0.00951192	0	2144
IL34	0.00950649	0	2145

IL28A	0.00949683	0	2146
CLEC4E	0.00949299	0	2147
RBM15	0.00949277	0	2148
FBXW7	0.00948037	0	2149
ARHGAP5	0.00946847	0	2150
SART3	0.00946732	0	2151
ESRRA	0.00946511	0	2152
NLRP10	0.00946465	0	2153
CSF2RB	0.00946166	0	2154
DUSP14	0.0094603	0	2155
RNASE2	0.00945246	0	2156
SRPX	0.00942679	0	2157
RC3H1	0.0094244	0	2158
RHOD	0.00941762	0	2159
MIR188	0.00941425	0	2160
REG1A	0.00938886	0	2161
SIPA1	0.00938768	0	2162
TCEB1	0.00938631	0	2163
TARDBP	0.00938148	0	2164
YTHDC1	0.00937943	0	2165
FBLIM1	0.00937595	0	2166
WBP7	0.00936131	0	2167
ND1	0.00935113	0	2168
LRRC23	0.00933953	0	2169
TRIM17	0.00932832	0	2170
FEZF2	0.00931356	0	2171
TM9SF2	0.00930826	0	2172
DOCK3	0.00930296	0	2173
THOC1	0.00930032	0	2174
PDCD6	0.00929577	0	2175
DSG1	0.00929241	0	2176
MLANA	0.00929111	0	2177
TUSC3	0.00927021	0	2178
PIAS4	0.00923969	0	2179
LGALS9	0.00923807	0	2180
NUP98	0.00921501	0	2181
DOCK2	0.0092104	0	2182
ADSL	0.00919016	0	2183
MLH1	0.00918503	0	2184
GADD45GIP1	0.0091831	0	2185
SART1	0.00917266	0	2186
YARS	0.00916682	0	2187
DUSP26	0.0091579	0	2188

CDK5	0.0091544	0	2189
AMELX	0.00915136	0	2190
LGR4	0.0091503	0	2191
C1QB	0.0091459	0	2192
RPLP2	0.00913816	0	2193
PLCD1	0.00913184	0	2194
NR0B1	0.00912682	0	2195
RNF20	0.00912435	0	2196
GNAS	0.00910076	0	2197
AFAP1	0.0090993	0	2198
HBEGF	0.00909619	0	2199
SERPINA4	0.0090946	0	2200
VAC14	0.00909417	0	2201
CD37	0.00909172	0	2202
MYH9	0.00908652	0	2203
TGOLN2	0.00908096	0	2204
TLX1	0.00907042	0	2205
DOK7	0.0090646	0	2206
ADI1	0.00906146	0	2207
GUSB	0.00906129	0	2208
LRRN2	0.00905168	0	2209
TCF19	0.00904793	0	2210
KCNH8	0.00904745	0	2211
RNF34	0.00904127	0	2212
BAG3	0.00903807	0	2213
DCC	0.00903459	0	2214
FL1	0.00903404	0	2215
HLA-C	0.00902716	0	2216
LRRK2	0.00902457	0	2217
SSBP2	0.00901354	0	2218
HRG	0.00899928	0	2219
POU2F2	0.00899854	0	2220
DOK4	0.00896117	0	2221
CDON	0.00895273	0	2222
VDAC1	0.0089522	0	2223
PINX1	0.00894674	0	2224
TRIP10	0.00894634	0	2225
DPCR1	0.00894094	0	2226
MNDA	0.00893764	0	2227
NFIC	0.00893231	0	2228
NEDD8	0.00891918	0	2229
ABCC1	0.00889716	0	2230
RAB10	0.00889572	0	2231

ARAP3	0.00888604	0	2232
ALOX15	0.00887463	0	2233
RAB11FIP3	0.00887434	0	2234
PHGDH	0.00885683	0	2235
IRAK2	0.00884573	0	2236
DCTN1	0.00884481	0	2237
SMAD9	0.00883777	0	2238
BIRC8	0.00883586	0	2239
BCL2A1	0.00883508	0	2240
HLA-DQA1	0.008833	0	2241
ETV4	0.00882645	0	2242
MIR146A	0.0088181	0	2243
RAB3IP	0.00881453	0	2244
CHL1	0.0088142	0	2245
LAMA1	0.00880238	0	2246
CCL28	0.00880104	0	2247
PIK3C3	0.0087989	0	2248
LALBA	0.0087932	0	2249
MBL3P	0.00878637	0	2250
CMD1B	0.00877819	0	2251
A1BG	0.00877729	0	2252
PHB	0.00876879	0	2253
PLXNA1	0.00876713	0	2254
LEPR	0.00873658	0	2255
CYTL1	0.00873559	0	2256
LRRC32	0.00873454	0	2257
EEF1D	0.0087282	0	2258
POLR2A	0.00872759	0	2259
TGIF1	0.00872727	0	2260
CDC34	0.00872527	0	2261
BCL11A	0.0087248	0	2262
TGFB2	0.00872217	0	2263
GYPA	0.00872032	0	2264
TRPC1	0.00871227	0	2265
ACVR1B	0.00871024	0	2266
MYO9B	0.00870854	0	2267
MTX1	0.00870383	0	2268
GSTT1	0.00870025	0	2269
PDHX	0.00867599	0	2270
UBA7	0.00866984	0	2271
DEK	0.00864852	0	2272
DCLK1	0.00864618	0	2273
SENP3	0.00864157	0	2274

CHRNA1	0.00863747	0	2275
ARL3	0.00862179	0	2276
USO1	0.00861547	0	2277
C5	0.00861108	0	2278
PITX2	0.00858651	0	2279
NPC1L1	0.00858003	0	2280
RNF7	0.00857858	0	2281
ITGA8	0.00857383	0	2282
BMP15	0.00857203	0	2283
AMFR	0.00857182	0	2284
INF2	0.00856774	0	2285
TRIB3	0.00856275	0	2286
ZCCHC6	0.00854207	0	2287
COL17A1	0.00852796	0	2288
TCIRG1	0.00851665	0	2289
LIPC	0.00851615	0	2290
PTGDR2	0.00851378	0	2291
UBR1	0.00851082	0	2292
PEAR1	0.00850823	0	2293
FOXP2	0.00850556	0	2294
UBE2L1	0.00850449	0	2295
MIER1	0.0084942	0	2296
MISP	0.00848961	0	2297
PTBP1	0.00848747	0	2298
NET1	0.00848383	0	2299
UBASH3A	0.00847813	0	2300
KDM6B	0.00847029	0	2301
MAD2L1	0.00846707	0	2302
DYNLT1	0.00846131	0	2303
RAB27B	0.00846122	0	2304
CCS	0.00845289	0	2305
CD200R1L	0.00843519	0	2306
KRT4	0.00842721	0	2307
RGS18	0.00841592	0	2308
CLC	0.00841545	0	2309
OGN	0.00840557	0	2310
TRPV6	0.00840224	0	2311
SFTPD	0.00836622	0	2312
MYO5B	0.00835022	0	2313
HIVEP1	0.00834674	0	2314
TIGIT	0.00834559	0	2315
DIAPH1	0.00833497	0	2316
RNF125	0.00833433	0	2317

ARHGAP31	0.00833155	0	2318
EPB41	0.00831078	0	2319
KLRC3	0.00830269	0	2320
PICALM	0.00830208	0	2321
RASSF5	0.00829737	0	2322
KPNA1	0.00827669	0	2323
ACVR1	0.00827392	0	2324
IGKV1D-22	0.00826812	0	2325
DKK1	0.00826091	0	2326
TAZ	0.00826038	0	2327
PRS	0.00824605	0	2328
TJP2	0.00824312	0	2329
RNF8	0.00823639	0	2330
TERF2IP	0.00823557	0	2331
ROCK2	0.0082325	0	2332
GPR107	0.00820642	0	2333
MTR	0.00820026	0	2334
SLC30A2	0.00819285	0	2335
TNIP1	0.00818685	0	2336
VAPA	0.00818219	0	2337
PPIG	0.00817246	0	2338
TRPV5	0.00817073	0	2339
ENPP2	0.00817062	0	2340
NUDT15	0.00815784	0	2341
STT3A	0.00814668	0	2342
PTPRJ	0.00814659	0	2343
NDRG2	0.00813974	0	2344
MSI2	0.00812991	0	2345
ATF4	0.0081199	0	2346
PSMB8	0.00811795	0	2347
SIGIRR	0.00811398	0	2348
CAST	0.00811154	0	2349
RHEB	0.00809197	0	2350
PTH1R	0.00808036	0	2351
DLX2	0.00808005	0	2352
GLIS2	0.00807501	0	2353
CCZ1	0.00807288	0	2354
ASF1A	0.0080641	0	2355
CLEC16A	0.00806114	0	2356
CYP26A1	0.0080523	0	2357
FANCC	0.00805107	0	2358
CORO1C	0.00804958	0	2359
STAM2	0.00803123	0	2360

ATG14	0.00802891	0	2361
DDIT3	0.00798447	0	2362
CD96	0.00798342	0	2363
GATA5	0.0079811	0	2364
TBCD	0.00797292	0	2365
ENDOG	0.00796877	0	2366
ORMDL3	0.00796833	0	2367
DR1	0.0079683	0	2368
YWHAZ	0.00795341	0	2369
YME1L1	0.00793868	0	2370
XCR1	0.00793589	0	2371
SOCS5	0.00793134	0	2372
EIF5B	0.00792828	0	2373
ANGPTL1	0.00792762	0	2374
SACS	0.00792553	0	2375
PLIN2	0.00792529	0	2376
ARL1	0.00792461	0	2377
HNRNPK	0.0079068	0	2378
PTGIR	0.00790636	0	2379
MROS	0.00790473	0	2380
ALPK1	0.00789979	0	2381
SAR1A	0.00789857	0	2382
HMMR	0.00789769	0	2383
CEL	0.00789766	0	2384
IL1RL1	0.00789758	0	2385
RPS6KA1	0.00789725	0	2386
PLA2G7	0.00787573	0	2387
PAX2	0.00786544	0	2388
STIM1	0.00785603	0	2389
SAA@	0.00785552	0	2390
PARK7	0.00785274	0	2391
TIMELESS	0.00785191	0	2392
PIBF1	0.00785126	0	2393
CAV2	0.00784935	0	2394
RAPSN	0.00784313	0	2395
CDR1	0.00783665	0	2396
RBP1	0.0078363	0	2397
CEACAM20	0.00782874	0	2398
STK33	0.00780679	0	2399
NCF4	0.00779384	0	2400
TDG	0.00779354	0	2401
OBSCN	0.00779336	0	2402
PTGES	0.00779206	0	2403

RALB	0.00778972	0	2404
NDUFA13	0.00778938	0	2405
PKD2L1	0.00778651	0	2406
FOXRED1	0.00777782	0	2407
DPM1	0.00777635	0	2408
MED14	0.00776592	0	2409
HBA2	0.00776052	0	2410
MIR532	0.00775763	0	2411
OSBP	0.00775628	0	2412
NGEF	0.00774852	0	2413
ETS2	0.00774842	0	2414
CLEC4A	0.00774628	0	2415
RNF31	0.00773462	0	2416
RABEP1	0.00773197	0	2417
ACSL3	0.00772551	0	2418
PACSIN2	0.00772468	0	2419
MIR142	0.00771725	0	2420
PABPN1	0.00771383	0	2421
SCLC1	0.00771365	0	2422
PCSK7	0.00771238	0	2423
FERMT3	0.00771018	0	2424
DLC1	0.00770964	0	2425
ACTR3	0.00770584	0	2426
HSD17B3	0.0076928	0	2427
ATIC	0.00769141	0	2428
TMEFF2	0.00768825	0	2429
SPATA2	0.007686	0	2430
KCNU1	0.0076844	0	2431
C4BPB	0.00767587	0	2432
CD3D	0.00767236	0	2433
CLEC10A	0.00767113	0	2434
SPG16	0.00767088	0	2435
TRAIP	0.00766194	0	2436
IL10RB	0.00765206	0	2437
MB	0.00765197	0	2438
RGS9	0.00764779	0	2439
DCT	0.00763898	0	2440
TNS3	0.00761939	0	2441
BNIP3	0.00761649	0	2442
EPS15	0.00761159	0	2443
AMACR	0.00761063	0	2444
ATG16L2	0.00761023	0	2445
AZI2	0.00760187	0	2446

BAMBI	0.00760074	0	2447
TGM6	0.00759762	0	2448
MAPK12	0.00758965	0	2449
TPR	0.00758724	0	2450
LGALS3BP	0.00758615	0	2451
GOLGA2	0.00758267	0	2452
RRAGA	0.00757388	0	2453
PRRX1	0.00757112	0	2454
AAVS1	0.00756334	0	2455
EPN1	0.00756115	0	2456
PSD	0.00753614	0	2457
KRT31	0.00753571	0	2458
PDZK1	0.00753317	0	2459
RASGRP2	0.00752894	0	2460
TK2	0.00751912	0	2461
ADAM28	0.00751707	0	2462
CCL1	0.00751619	0	2463
CYP19A1	0.00751483	0	2464
MEGF10	0.0075097	0	2465
SLC27A4	0.00750956	0	2466
LNX1	0.00750183	0	2467
SAMD4A	0.00749848	0	2468
CDC73	0.00749698	0	2469
CECR	0.00749631	0	2470
SF	0.00749295	0	2471
PKP3	0.0074872	0	2472
SOX17	0.0074848	0	2473
HARS	0.00747686	0	2474
KL	0.00745361	0	2475
LAPTM5	0.00744893	0	2476
DNLZ	0.00743942	0	2477
WWP1	0.00743472	0	2478
AMPH	0.00741929	0	2479
KPNA3	0.00741878	0	2480
CPVL	0.00741682	0	2481
GTF2A1L	0.00741642	0	2482
HNRNPH1	0.00740503	0	2483
AMER1	0.0074031	0	2484
ACR	0.0073615	0	2485
WRNIP1	0.00735904	0	2486
BMPR2	0.00735443	0	2487
BTN3A1	0.007341	0	2488
SOX7	0.00734033	0	2489

PRKG1	0.00733649	0	2490
UPF1	0.00733398	0	2491
FLOT2	0.0073309	0	2492
ERRFI1	0.00731852	0	2493
PPID	0.00731708	0	2494
ADA	0.00731261	0	2495
CMPK2	0.00730733	0	2496
EML1	0.00730701	0	2497
ROBO4	0.00729683	0	2498
HEATR3	0.00728991	0	2499
HDAC9	0.00727441	0	2500
MUT	0.00726959	0	2501
RAPGEF3	0.00726674	0	2502
RARG	0.00726408	0	2503
AGAP1	0.00725685	0	2504
CUL1	0.00725267	0	2505
MIR216A	0.00724357	0	2506
AES	0.00724024	0	2507
NCOA5	0.00723946	0	2508
TAS2R63P	0.00723711	0	2509
TXNRD1	0.00722817	0	2510
KLRC2	0.00722355	0	2511
DIAPH3	0.00722214	0	2512
LTB4R2	0.00722135	0	2513
NTAN1	0.0072197	0	2514
PHF20	0.00721113	0	2515
CPSF6	0.0071996	0	2516
SLC22A17	0.00719706	0	2517
NPHP1	0.00718263	0	2518
EIF2AK1	0.00717641	0	2519
SH2B2	0.00717127	0	2520
NMI	0.00715695	0	2521
ABCG8	0.00714454	0	2522
MIR424	0.00713839	0	2523
STX7	0.00713685	0	2524
MIR489	0.00712832	0	2525
CYP1A2	0.00712663	0	2526
MIR302B	0.00712108	0	2527
TACC3	0.00711958	0	2528
ZNF76	0.00711374	0	2529
KRT10	0.00710707	0	2530
UBE2E1	0.00710493	0	2531
PPRC1	0.00710146	0	2532

SLPI	0.0070993	0	2533
TK1	0.00708726	0	2534
ACSM1	0.00708632	0	2535
USP15	0.00708008	0	2536
ZMIZ1	0.00707441	0	2537
RBM39	0.007073	0	2538
EFNB3	0.00706755	0	2539
AURKB	0.00705993	0	2540
MAPK13	0.0070593	0	2541
SUPT5H	0.00704836	0	2542
ENPEP	0.00704653	0	2543
HYAL2	0.00704623	0	2544
NOX5	0.0070449	0	2545
NPTN	0.00704486	0	2546
MAGI3	0.00704401	0	2547
UGT2B17	0.00703432	0	2548
SH2D2A	0.0070328	0	2549
IVL	0.00703135	0	2550
HBZ	0.00702947	0	2551
NCOA6	0.00702543	0	2552
ELF2	0.0070208	0	2553
SCRIB	0.00701729	0	2554
ABCB4	0.00701322	0	2555
PTHLH	0.0070103	0	2556
ALDH3A1	0.00700866	0	2557
MMP23B	0.00700679	0	2558
PIR	0.00700359	0	2559
VANGL2	0.00699845	0	2560
PPP1CA	0.00699839	0	2561
REPS2	0.00699026	0	2562
FSHR	0.00698811	0	2563
CCR10	0.00698795	0	2564
MIR486	0.00698137	0	2565
EHMT2	0.00696116	0	2566
F2RL2	0.00696086	0	2567
ITGB1	0.00696008	0	2568
NQO1	0.00695928	0	2569
TRERF1	0.00695571	0	2570
PIGP	0.00695537	0	2571
CA9	0.00695395	0	2572
IL26	0.00695214	0	2573
SMAL	0.00693614	0	2574
SLC16A1	0.00692915	0	2575

AHNAK	0.00692779	0	2576
SOD2	0.00691728	0	2577
FZD4	0.00690437	0	2578
ROBO2	0.00690043	0	2579
IGHJ2	0.00689988	0	2580
PREX1	0.00689191	0	2581
EIF4A1	0.00686952	0	2582
SGCA	0.00686905	0	2583
MAST2	0.00686756	0	2584
NAMPT	0.0068634	0	2585
HBS1L	0.00686004	0	2586
ST13	0.00685781	0	2587
ACTA1	0.00685664	0	2588
SNX1	0.00684199	0	2589
PLD3	0.00683597	0	2590
SMUG1	0.00682214	0	2591
RNF168	0.00680565	0	2592
MIR181A1	0.00680519	0	2593
MBD4	0.00680081	0	2594
ZNF569	0.00679967	0	2595
FXN	0.00679209	0	2596
ADARB1	0.00676306	0	2597
ACSL4	0.00675954	0	2598
WNT1	0.00675731	0	2599
SIM2	0.00675728	0	2600
CYTB	0.0067566	0	2601
AHRR	0.00675603	0	2602
GEM	0.00675492	0	2603
FAIM	0.00675101	0	2604
EZH1	0.00674997	0	2605
ALG1	0.00674543	0	2606
SH3BP1	0.0067451	0	2607
MOBP	0.0067383	0	2608
DLEU2	0.00673333	0	2609
CACNA1E	0.00672421	0	2610
LAMP3	0.00672414	0	2611
C4B	0.0067218	0	2612
S1PR4	0.00671348	0	2613
RAB8B	0.00671274	0	2614
MIR631	0.00669305	0	2615
HK3	0.00669026	0	2616
KLF8	0.00668957	0	2617
RNASEL	0.00667037	0	2618

ZC3HAV1	0.00666804	0	2619
SERPINA9	0.00666418	0	2620
GIPC1	0.00666314	0	2621
GCSH	0.00666232	0	2622
PCDH8	0.00665681	0	2623
PELI3	0.00665575	0	2624
ITGA6	0.00665168	0	2625
SNCAIP	0.00664579	0	2626
FGF3	0.00663403	0	2627
PTPN23	0.00663211	0	2628
SLC4A3	0.00662846	0	2629
NAIP	0.00662754	0	2630
NHLH1	0.0066252	0	2631
C1GALT1	0.00660748	0	2632
OGG1	0.00660253	0	2633
TPPP	0.00659959	0	2634
RETNLB	0.00659288	0	2635
NR2F6	0.00658387	0	2636
SIGLEC5	0.00657848	0	2637
PCGF2	0.00657462	0	2638
ACSL6	0.00655668	0	2639
NRG2	0.00655025	0	2640
C6ORF47	0.0065475	0	2641
FSCN1	0.00653521	0	2642
ARF5	0.00653442	0	2643
TOMM70A	0.00652941	0	2644
RGS16	0.00652648	0	2645
FGF21	0.00652504	0	2646
TMED10	0.00652417	0	2647
BHLHE40	0.00651764	0	2648
PIGR	0.00651709	0	2649
ABI2	0.00651385	0	2650
SGMS1	0.00651339	0	2651
CAMKK1	0.00651283	0	2652
SLC30A8	0.00650852	0	2653
PRPF3	0.00650778	0	2654
ELK4	0.00650539	0	2655
MIR222	0.00649785	0	2656
RP9	0.00649631	0	2657
TMED1	0.00648423	0	2658
ANXA4	0.00647306	0	2659
SAMSN1	0.00646935	0	2660
PRPH2	0.00646334	0	2661

TRIM13	0.00646019	0	2662
NMRAL1	0.00644856	0	2663
AMH	0.00644721	0	2664
ARFGEF1	0.00644631	0	2665
HBP1	0.006445	0	2666
HEXIM1	0.00644035	0	2667
SPTA1	0.00643735	0	2668
RGS2	0.00642781	0	2669
MMAB	0.00642352	0	2670
MR1	0.00642106	0	2671
PTCRA	0.00640862	0	2672
GYPC	0.00640292	0	2673
MTTP	0.00639997	0	2674
TBX4	0.00639346	0	2675
MIR130A	0.00639138	0	2676
CNC	0.00638463	0	2677
GRK5	0.00637663	0	2678
RNF5	0.00636928	0	2679
UBD	0.0063682	0	2680
TRIM32	0.00636445	0	2681
PKD2	0.00636195	0	2682
MIR146B	0.00634747	0	2683
PHYH	0.00634217	0	2684
CYP2C19	0.00633101	0	2685
TRIP11	0.0063288	0	2686
TNIK	0.0063283	0	2687
NCAM2	0.0063238	0	2688
PAM	0.00632255	0	2689
SMAP	0.00631913	0	2690
FIZ1	0.00631764	0	2691
GAK	0.00631735	0	2692
CREB3L3	0.00631643	0	2693
HLA-DPA1	0.0063136	0	2694
STOML2	0.00631203	0	2695
SRCIN1	0.00630103	0	2696
NELFCD	0.00630013	0	2697
SH3PXD2B	0.00630013	0	2698
REM1	0.00629836	0	2699
KAT6B	0.00629445	0	2700
ACPP	0.0062843	0	2701
PKDCC	0.00628144	0	2702
ADCY9	0.00627917	0	2703
AGAP2	0.00627349	0	2704

CPD	0.00627258	0	2705
5-Mar	0.00627174	0	2706
ALL1	0.00626872	0	2707
SGMS2	0.00625222	0	2708
ITSN2	0.00624123	0	2709
TINF2	0.00623301	0	2710
SAMHD1	0.0062272	0	2711
DCP1A	0.00622707	0	2712
ST14	0.0062259	0	2713
ATF7IP	0.00621646	0	2714
PLEKHG2	0.00621637	0	2715
USP21	0.00620885	0	2716
ECT2	0.00620798	0	2717
IFI6	0.00619973	0	2718
SIGLEC8	0.00619334	0	2719
CTCF	0.00618474	0	2720
ALDH1A2	0.00618266	0	2721
IDUA	0.00618158	0	2722
NBPF10	0.00617454	0	2723
UBE2K	0.00617093	0	2724
RAB35	0.00617035	0	2725
SORL1	0.00616617	0	2726
ATP8B1	0.00616454	0	2727
XPR1	0.00615448	0	2728
CD8B	0.00614853	0	2729
TSSK1B	0.00614424	0	2730
HOXC4	0.00614302	0	2731
KDM4A	0.00613487	0	2732
SUGT1	0.00613096	0	2733
FGF9	0.00612923	0	2734
MRAP	0.00612781	0	2735
TNK1	0.00612521	0	2736
APOL1	0.00612116	0	2737
MUC4	0.00611961	0	2738
ST3	0.00611378	0	2739
NAE1	0.00611226	0	2740
PLTP	0.00611167	0	2741
MORF4	0.00610563	0	2742
NDFIP1	0.0061032	0	2743
DNAJB1	0.00609301	0	2744
DERL1	0.00609278	0	2745
DOCK10	0.00609234	0	2746
FOXA1	0.00609163	0	2747

CD84	0.0060891	0	2748
IGLJ2	0.00608456	0	2749
FZD2	0.00607918	0	2750
PHLPP1	0.0060754	0	2751
XAF1	0.00607063	0	2752
PLEKHG6	0.00606865	0	2753
ZIC5	0.00606848	0	2754
AIP	0.00606144	0	2755
MIR182	0.00605767	0	2756
BHLHA15	0.00605052	0	2757
CSN3	0.00604827	0	2758
KLHL2	0.00603322	0	2759
BUB1B	0.0060324	0	2760
PRKACA	0.00603148	0	2761
MED12	0.00602295	0	2762
U2AF2	0.0060225	0	2763
CRYZ	0.00601972	0	2764
UTP3	0.00601502	0	2765
POP1	0.00601445	0	2766
IL12B	0.00600489	0	2767
FBXO5	0.00600024	0	2768
FBXO7	0.00599768	0	2769
NLRP4	0.0059974	0	2770
IMMT	0.0059971	0	2771
COX5A	0.00599287	0	2772
EXOSC8	0.00599013	0	2773
FUS	0.00598292	0	2774
IL4I1	0.00598159	0	2775
KRT17	0.00598089	0	2776
MYEF2	0.00597841	0	2777
DEGS1	0.0059701	0	2778
EEC1	0.00596609	0	2779
TRIM38	0.00596037	0	2780
BMP5	0.00595684	0	2781
CTAG1A	0.00594827	0	2782
STK38	0.00594547	0	2783
SPTBN1	0.00594545	0	2784
LONP1	0.00593992	0	2785
TRIM23	0.00593306	0	2786
CERK	0.00593132	0	2787
HSP90AA2	0.0059283	0	2788
VIT	0.00592562	0	2789
TLE1	0.0059163	0	2790

BCL2L14	0.00591538	0	2791
MIR155HG	0.00590601	0	2792
PARK2	0.00590511	0	2793
CRYAB	0.00590142	0	2794
HMI	0.00590003	0	2795
ING2	0.00589944	0	2796
HOXB8	0.00589745	0	2797
FAM123B	0.00589107	0	2798
COPB1	0.00588988	0	2799
HSP90AB1	0.00588624	0	2800
SIAH1	0.00588457	0	2801
COASY	0.00588381	0	2802
TBL1XR1	0.00587894	0	2803
CASP4	0.00587458	0	2804
BIN1	0.00587181	0	2805
MCOLN1	0.00587005	0	2806
CPOX	0.00586351	0	2807
MAL2	0.00586291	0	2808
PTPN3	0.00586035	0	2809
MPP5	0.00584667	0	2810
IQSEC1	0.00584223	0	2811
UFD1L	0.00584028	0	2812
MYL12B	0.00583601	0	2813
PIM3	0.00583146	0	2814
PROKR2	0.00582435	0	2815
NLRP6	0.0058205	0	2816
EPHB6	0.0058143	0	2817
7-Mar	0.00581208	0	2818
DDX3Y	0.00581082	0	2819
MIR203	0.00580843	0	2820
TOP1	0.00580436	0	2821
BEND5	0.00580198	0	2822
BBOX1	0.00579432	0	2823
SMARCA2	0.00579138	0	2824
MYH10	0.00578454	0	2825
AFF4	0.00578215	0	2826
MAX	0.0057748	0	2827
CYP17A1	0.00577029	0	2828
GZMH	0.00576175	0	2829
CCNH	0.00576086	0	2830
SLC38A5	0.00576006	0	2831
ESRRB	0.0057593	0	2832
IBD5	0.00575851	0	2833

CSE	0.00575311	0	2834
STXBP3	0.0057481	0	2835
LSS	0.00574344	0	2836
DYNLRB1	0.00574207	0	2837
RNF19B	0.00574135	0	2838
EDEM1	0.00574082	0	2839
RAP1GAP	0.00573821	0	2840
ARL2BP	0.00573123	0	2841
PDE2A	0.0057286	0	2842
RHOJ	0.00572088	0	2843
PPP1CB	0.00571666	0	2844
IGKV2-23	0.00571223	0	2845
RIT1	0.00571212	0	2846
GSC	0.00570892	0	2847
MYH6	0.00569968	0	2848
HSR	0.00569623	0	2849
LYVE1	0.00569461	0	2850
KCNA2	0.00569303	0	2851
HOXB6	0.00569268	0	2852
TAS2R6P	0.0056852	0	2853
SKIV2L	0.00567552	0	2854
KAT2A	0.00567279	0	2855
STAM	0.00566965	0	2856
CD300LF	0.00566418	0	2857
CACNB4	0.00566316	0	2858
PYGO2	0.00566117	0	2859
INTS6	0.00565705	0	2860
SAA3P	0.00565627	0	2861
POTEF	0.00564492	0	2862
GREB1	0.00564376	0	2863
PGK1	0.00564355	0	2864
PALLD	0.00564119	0	2865
S100A4	0.00563874	0	2866
RBM4	0.00563667	0	2867
ZHX1	0.00563253	0	2868
PSG1	0.00562412	0	2869
DUSP15	0.00562371	0	2870
MARVELD2	0.00560918	0	2871
NR1H3	0.00560239	0	2872
MMAA	0.00559852	0	2873
HPN	0.00559615	0	2874
CSH1	0.00559526	0	2875
RAB6A	0.00559159	0	2876

RPL12	0.0055818	0	2877
NPTX1	0.00557299	0	2878
TTC3	0.00557235	0	2879
CYP2C9	0.00557223	0	2880
RTL1	0.00556971	0	2881
UGT1A	0.00556902	0	2882
PRDX2	0.00555741	0	2883
NFASC	0.00554865	0	2884
FCRL6	0.00554058	0	2885
PPP1R1A	0.00553807	0	2886
IRGM	0.00553806	0	2887
APOBEC3F	0.00553615	0	2888
NEO1	0.00553482	0	2889
SHANK1	0.00553395	0	2890
SCARNA5	0.00553302	0	2891
PAK4	0.00553105	0	2892
ROBO1	0.00551457	0	2893
LSM2	0.00551359	0	2894
LATS1	0.00550759	0	2895
SSNA1	0.00549744	0	2896
ASPSCR1	0.00549602	0	2897
HLA-DPB1	0.00549401	0	2898
PPBP	0.00549191	0	2899
UBE2E2	0.00549186	0	2900
RFX1	0.00549125	0	2901
RGS20	0.00549088	0	2902
TMOD4	0.00548974	0	2903
JUP	0.00548956	0	2904
SLC24A2	0.00548641	0	2905
ITLN1	0.00548158	0	2906
B4GALNT2	0.00546077	0	2907
XRCC1	0.00545891	0	2908
PARVB	0.00545273	0	2909
SLC1A6	0.00544945	0	2910
KTWS	0.00544828	0	2911
RAB27A	0.00544823	0	2912
KCNQ4	0.00544792	0	2913
MYO1A	0.00544477	0	2914
CETP	0.00544358	0	2915
OCSTAMP	0.00544085	0	2916
SPDEF	0.00544085	0	2917
BRE	0.00544021	0	2918
STAB2	0.00543522	0	2919

KIAA1549	0.00543442	0	2920
LGMN	0.00543199	0	2921
HFE	0.00543004	0	2922
CYFIP2	0.00542893	0	2923
NRK	0.00542774	0	2924
USP3	0.00542543	0	2925
SRY	0.00542228	0	2926
RPS2	0.00542183	0	2927
SIGLEC10	0.00542066	0	2928
P2RY8	0.00541553	0	2929
ELF5	0.00540827	0	2930
RBM5	0.0054064	0	2931
PPEF1	0.00540178	0	2932
PRD	0.00540101	0	2933
BUD31	0.00539634	0	2934
CARD10	0.00539617	0	2935
REG3G	0.00539101	0	2936
RHOF	0.00538992	0	2937
ATP6AP1	0.0053822	0	2938
FGD4	0.00538025	0	2939
MUC2	0.00537285	0	2940
UVRAG	0.00536205	0	2941
SCN2A	0.00536201	0	2942
PSMB10	0.00535744	0	2943
RPE	0.00535141	0	2944
ELL	0.00534232	0	2945
HLF	0.00534194	0	2946
SLC26A6	0.00533894	0	2947
TFAP2C	0.00533829	0	2948
RRAGB	0.00532737	0	2949
TRIM37	0.0053243	0	2950
ETF1	0.00532237	0	2951
MIR191	0.00531905	0	2952
OASL	0.0053161	0	2953
PSMD10	0.00531564	0	2954
MCAT	0.00531542	0	2955
MPDZ	0.00531171	0	2956
ANTXR1	0.00531164	0	2957
SLC36A1	0.00530392	0	2958
JMJD6	0.00530346	0	2959
GPX1	0.00530062	0	2960
IGKV2D-24	0.00528016	0	2961
STRN	0.00527804	0	2962

CAV3	0.00527462	0	2963
PHF11	0.00527392	0	2964
COPS8	0.00527171	0	2965
AMPD1	0.00526756	0	2966
MIR22	0.00525874	0	2967
FGB	0.00525525	0	2968
PLA2G6	0.00525462	0	2969
ERVW-3	0.00525296	0	2970
MAPK8IP1	0.00525214	0	2971
TNFSF15	0.0052513	0	2972
SLC1A5	0.00524751	0	2973
CTTNBP2	0.00524363	0	2974
MFN1	0.00523525	0	2975
EPHB3	0.00523256	0	2976
NUP214	0.00523061	0	2977
SET	0.00521967	0	2978
IRX5	0.00521791	0	2979
SLA	0.00521736	0	2980
HOXB7	0.0052164	0	2981
GLDN	0.00521375	0	2982
DGCR14	0.00520516	0	2983
DLX3	0.00520434	0	2984
PCYT2	0.00520301	0	2985
CLEC12A	0.00520239	0	2986
ABCC10	0.00519798	0	2987
SLC25A20	0.00519191	0	2988
DHX36	0.00519186	0	2989
OCRL	0.00518668	0	2990
DPF3	0.00518362	0	2991
GGPS1	0.00516616	0	2992
STAU1	0.00515811	0	2993
SNRPD1	0.0051578	0	2994
CYCSP3	0.0051524	0	2995
TFG	0.00514744	0	2996
EXOSC1	0.0051474	0	2997
WNK2	0.00514006	0	2998
SSTR1	0.00513995	0	2999
AJUBA	0.00513446	0	3000
TRPC2	0.00512949	0	3001
PRKX	0.00511128	0	3002
POU2AF1	0.00510407	0	3003
MSX1	0.00510215	0	3004
IVNS1ABP	0.00510119	0	3005

UBE3C	0.00509289	0	3006
CRLF3	0.00509043	0	3007
CHI3L1	0.00508765	0	3008
DHCR24	0.00508574	0	3009
RNPEP	0.00508548	0	3010
SLC9A5	0.00508345	0	3011
FNBP1	0.00508064	0	3012
S1PR3	0.00507714	0	3013
CRAT	0.00507502	0	3014
G6PC	0.00507135	0	3015
TMEM37	0.00507053	0	3016
RAB12	0.00506943	0	3017
COL4A5	0.00506873	0	3018
NCAPG2	0.0050682	0	3019
MYBL1	0.00506675	0	3020
RHAG	0.00505938	0	3021
PPM1J	0.00505542	0	3022
PIPOX	0.00505419	0	3023
KCNC4	0.0050511	0	3024
BCRP3	0.0050493	0	3025
THAS	0.00504056	0	3026
GNPAT	0.00503858	0	3027
AFA1	0.00503825	0	3028
ABCA3	0.00503484	0	3029
ZNF711	0.00503471	0	3030
PPIC	0.00503298	0	3031
CMIP	0.005032	0	3032
VARS2	0.00503059	0	3033
CYTIP	0.00502756	0	3034
GALNT2	0.00502518	0	3035
ZNF224	0.00501965	0	3036
SMPD3	0.00501912	0	3037
HAPLN1	0.00501567	0	3038
PDXK	0.00501183	0	3039
EAF1	0.0050113	0	3040
RAB8A	0.00500823	0	3041
ACLY	0.00500744	0	3042
ALPI	0.00500316	0	3043
API5	0.00500152	0	3044
CCL24	0.00500038	0	3045
FAM132B	0.00499624	0	3046
HLA-DMA	0.00499034	0	3047
ABI3	0.00498809	0	3048

KARS	0.00496814	0	3049
ARL4C	0.00496707	0	3050
ADD1	0.0049624	0	3051
KRT27	0.00495639	0	3052
ANO1	0.00495379	0	3053
BST1	0.00495141	0	3054
EMC10	0.00495045	0	3055
CRYGD	0.00494835	0	3056
EPHA7	0.00494611	0	3057
FXYD2	0.00494263	0	3058
CMAS	0.00494116	0	3059
TNIP3	0.00494109	0	3060
PRPF40A	0.00493759	0	3061
PITPNM3	0.00493733	0	3062
CSNK1D	0.00492981	0	3063
GZMM	0.00491479	0	3064
WASF1	0.00491474	0	3065
G6PC3	0.00491313	0	3066
SCAMP2	0.00490967	0	3067
REXO2	0.00490629	0	3068
MS4A1	0.00490619	0	3069
HOTAIRM1	0.00490418	0	3070
DLX1	0.00489818	0	3071
TRPM3	0.00489593	0	3072
CEP350	0.00488906	0	3073
SHFM1	0.0048811	0	3074
ABCB5	0.0048781	0	3075
IGSF8	0.00487343	0	3076
PSMA4	0.00487075	0	3077
HEXDC	0.00486558	0	3078
HHEX	0.00486037	0	3079
CXXC5	0.00485714	0	3080
DNAJB6	0.00485531	0	3081
RBM10	0.00485083	0	3082
FGA	0.00484967	0	3083
RNY4	0.00484625	0	3084
SPRR1A	0.0048428	0	3085
FUNDC1	0.00484103	0	3086
UBE2G2	0.00483289	0	3087
KIAA1524	0.00483072	0	3088
PVRL2	0.00483042	0	3089
TCRA	0.00482979	0	3090
FHOD1	0.00482478	0	3091

ADAM12	0.00481427	0	3092
B4GALT7	0.00481365	0	3093
FZD5	0.00480764	0	3094
IREB2	0.00480494	0	3095
DTX4	0.00480215	0	3096
USP38	0.00480215	0	3097
VPS37C	0.00480215	0	3098
PPP1R15A	0.0047986	0	3099
SMCR8	0.00479733	0	3100
ATG4B	0.00479367	0	3101
VPS51	0.00479367	0	3102
DSTN	0.00478926	0	3103
GLIPR2	0.00478388	0	3104
STRA13	0.00476988	0	3105
ENDOU	0.00476504	0	3106
LDLRAD4	0.00476325	0	3107
COTL1	0.00476138	0	3108
MSH6	0.00475756	0	3109
SP3	0.00475377	0	3110
PAR1	0.00475149	0	3111
KRT88P	0.00474448	0	3112
RGS13	0.00474244	0	3113
KCNH1	0.00473582	0	3114
OGT	0.00473459	0	3115
FAS-AS1	0.00473186	0	3116
SLC25A19	0.00473048	0	3117
MIR98	0.00472952	0	3118
SLC25A46	0.00472653	0	3119
RAB14	0.00472101	0	3120
AP1M1	0.00471834	0	3121
FSD2	0.00471669	0	3122
XCE	0.00471493	0	3123
PPP3R1	0.00471178	0	3124
RING1	0.00470497	0	3125
LMO1	0.00470395	0	3126
NME2	0.00469422	0	3127
KRT15	0.00469315	0	3128
EXOC1	0.00468439	0	3129
COIL	0.00468106	0	3130
CD74	0.00467985	0	3131
MIR185	0.00467871	0	3132
POLG2	0.00467526	0	3133
CNN3	0.00467303	0	3134

HAT1	0.00467092	0	3135
THRSP	0.00466804	0	3136
ASZ1	0.00466719	0	3137
GSX1	0.00465933	0	3138
HBD	0.00465191	0	3139
CKMT1B	0.0046462	0	3140
FPR2	0.00464081	0	3141
ANKRD11	0.00463882	0	3142
SGK223	0.00462889	0	3143
GTF2H1	0.00462845	0	3144
ITGA1	0.00462587	0	3145
AOS	0.00462532	0	3146
DTX1	0.00461472	0	3147
SECISBP2	0.00461205	0	3148
MS4A2	0.00461086	0	3149
RNU4-1	0.0046107	0	3150
MAP3K7CL	0.00461	0	3151
OR1J2	0.00460956	0	3152
RPL7P	0.00460956	0	3153
SLC29A1	0.00460432	0	3154
ING3	0.00460329	0	3155
RICTOR	0.00460289	0	3156
C14ORF166	0.00459517	0	3157
MAGED1	0.00459142	0	3158
SHF	0.00458869	0	3159
DGKA	0.00457895	0	3160
CDCA5	0.00457681	0	3161
ING4	0.00457407	0	3162
EQTN	0.00457216	0	3163
BCL11B	0.00457205	0	3164
PTGER4	0.00457109	0	3165
ARHGEF15	0.00456695	0	3166
ZFYVE20	0.00456593	0	3167
BMPR1A	0.00456568	0	3168
CENPE	0.00456322	0	3169
SALL1	0.00456267	0	3170
ZNF300	0.00456227	0	3171
KLK8	0.00455832	0	3172
CLCN2	0.00455487	0	3173
PTLS	0.0045541	0	3174
CDH3	0.00455224	0	3175
ZNF107	0.00454964	0	3176
GLMN	0.00453858	0	3177

TUFM	0.00453792	0	3178
CRG	0.00453209	0	3179
CD3G	0.0045306	0	3180
NDFIP2	0.00452942	0	3181
THRB	0.00452253	0	3182
PLEKHA1	0.00451859	0	3183
5-Sep	0.00451789	0	3184
KCNN4	0.00451601	0	3185
KRT13	0.0045083	0	3186
VPS11	0.00450772	0	3187
VHLL	0.00450588	0	3188
CCRL1	0.00450419	0	3189
CALCR	0.00450392	0	3190
FERMT1	0.00449925	0	3191
MARK2	0.00449053	0	3192
DOCK5	0.00447582	0	3193
SOX13	0.00447511	0	3194
SLC4A4	0.00447288	0	3195
PEG3	0.00446726	0	3196
NRF1	0.00446019	0	3197
PSMB4	0.0044592	0	3198
NDUFB4	0.00445709	0	3199
CTF1	0.00445329	0	3200
PROCR	0.00444707	0	3201
HFE2	0.00444368	0	3202
CDC42EP2	0.00444134	0	3203
CCL7	0.00443817	0	3204
LPAR1	0.00443479	0	3205
HMGCL	0.0044309	0	3206
MXD3	0.00443085	0	3207
BPI	0.00443052	0	3208
SENP7	0.00442693	0	3209
SH3GLB1	0.00442596	0	3210
PDK2	0.00442468	0	3211
REG3A	0.00441437	0	3212
HOXB1	0.00441372	0	3213
PDZD2	0.0044119	0	3214
EHHADH	0.00441155	0	3215
RALGPS1	0.00441075	0	3216
HIC1	0.00441022	0	3217
DBP	0.00440822	0	3218
DEFB103A	0.00440803	0	3219
CASS4	0.00440389	0	3220

PON1	0.00440364	0	3221
CSH2	0.00439428	0	3222
ZBTB20	0.00439105	0	3223
FABP4	0.00438511	0	3224
PLF	0.00438232	0	3225
CD5L	0.00438125	0	3226
SLC48A1	0.00437752	0	3227
FGD5	0.00437362	0	3228
PTPN9	0.00437189	0	3229
TBL1Y	0.00437125	0	3230
DTNBP1	0.00436807	0	3231
LRSAM1	0.00436711	0	3232
RD3	0.004367	0	3233
LRP4	0.00435988	0	3234
CWC15	0.00435977	0	3235
FMNL1	0.00435719	0	3236
C1QA	0.00435276	0	3237
PLA1A	0.0043502	0	3238
ATXN2	0.00434747	0	3239
MARCKSL1	0.00434483	0	3240
GSS	0.00433937	0	3241
SRSF10	0.00433765	0	3242
NFKBID	0.00433537	0	3243
DDN	0.00433174	0	3244
GIT2	0.00432945	0	3245
RHOV	0.00432675	0	3246
TNFRSF6B	0.00431604	0	3247
FOXD3	0.00431469	0	3248
TOMM20	0.00431345	0	3249
RTCB	0.00431313	0	3250
PLK4	0.00431302	0	3251
CCP110	0.00430738	0	3252
KIF2A	0.00430715	0	3253
ANXA7	0.00430323	0	3254
RAB11B	0.00430297	0	3255
DIEXF	0.004296	0	3256
GDF2	0.00429312	0	3257
MYLK	0.00428758	0	3258
MUSK	0.00428681	0	3259
USP42	0.00428597	0	3260
ZFP36L2	0.00428254	0	3261
PSMB7	0.0042803	0	3262
NRM	0.00428029	0	3263

MYH2	0.00427979	0	3264
EPC2	0.00427799	0	3265
TRIL	0.00427799	0	3266
TLN1	0.00427614	0	3267
ACSS2	0.00427582	0	3268
SERPINF2	0.00426815	0	3269
NANS	0.00426685	0	3270
ZNF331	0.00426658	0	3271
EYA4	0.0042652	0	3272
WIPI2	0.00425947	0	3273
LIMK2	0.00425375	0	3274
DNASE1	0.00423948	0	3275
RXRB	0.0042383	0	3276
SPAG9	0.00423801	0	3277
UGT1A7	0.00423333	0	3278
SND1-IT1	0.00422886	0	3279
AKAP11	0.0042277	0	3280
EXOC5	0.00422428	0	3281
BLOC1S6	0.00422038	0	3282
POU2F3	0.00421916	0	3283
CDH15	0.00421573	0	3284
CELF1	0.00421168	0	3285
RPS6KA5	0.00420669	0	3286
C4BPA	0.0042037	0	3287
PAPPA2	0.00420335	0	3288
AKT3	0.00419917	0	3289
FEM1B	0.0041932	0	3290
TSFM	0.00418778	0	3291
SMAP1	0.00418769	0	3292
PSMA1	0.00418477	0	3293
JTB	0.0041844	0	3294
SPRY4	0.00417835	0	3295
TASP1	0.00417096	0	3296
PIK3IP1	0.00417001	0	3297
HEXA	0.00416541	0	3298
CLSTN1	0.00416388	0	3299
AIM1	0.00416325	0	3300
PFKFB4	0.00415889	0	3301
MXD4	0.00415804	0	3302
NAT2	0.00415159	0	3303
CHRNA4	0.00415108	0	3304
ARNTL	0.00415071	0	3305
FIGF	0.0041492	0	3306

PTPRZ1	0.00414861	0	3307
LGI1	0.0041418	0	3308
BCL2L15	0.00413982	0	3309
RCD1	0.00413729	0	3310
FBXL2	0.00413194	0	3311
CHMP4B	0.00412446	0	3312
BCS1L	0.00412114	0	3313
DLX4	0.00412036	0	3314
MADCAM1	0.0041193	0	3315
TALDO1	0.00411579	0	3316
CYP24A1	0.00411455	0	3317
MIRLET7I	0.00410865	0	3318
SASS6	0.00410853	0	3319
GTF2H3	0.00410135	0	3320
FGL2	0.00409927	0	3321
GYPB	0.0040981	0	3322
FGFRL1	0.00409788	0	3323
NUDC	0.00409722	0	3324
CLYBL	0.00409678	0	3325
TRIM5	0.00409556	0	3326
ZNF135	0.00409108	0	3327
IFNA14	0.00409071	0	3328
PACS1	0.00408764	0	3329
ALPPL2	0.00408752	0	3330
FPR3	0.00408669	0	3331
ARHGAP29	0.0040862	0	3332
PINK1	0.00408036	0	3333
SERPINB13	0.00407974	0	3334
ARFGAP1	0.00407828	0	3335
SNRNP70	0.00407445	0	3336
MSD	0.00407371	0	3337
CALU	0.00406874	0	3338
BTNL2	0.00406698	0	3339
PIK3R4	0.00406464	0	3340
TARBP2P	0.0040634	0	3341
TRIM10	0.00405678	0	3342
APBA1	0.00405519	0	3343
RPS27A	0.00405291	0	3344
ADAMTS10	0.00405243	0	3345
FBXO18	0.00404997	0	3346
CLIP1	0.00404927	0	3347
ICAM2	0.0040456	0	3348
SH3RF1	0.00404148	0	3349

HMGN2	0.00403997	0	3350
VPS18	0.00403979	0	3351
BLOC1S1	0.00403912	0	3352
DNAJC15	0.00403542	0	3353
TRIM72	0.00403503	0	3354
MYBPC3	0.00403405	0	3355
IFNA7	0.00403388	0	3356
DGKD	0.00402671	0	3357
SLC15A2	0.00402654	0	3358
STMN1	0.00402459	0	3359
SDCBP	0.00401994	0	3360
GPR143	0.00401561	0	3361
HNF1B	0.00401513	0	3362
RAPGEF6	0.00401326	0	3363
RNF13	0.00401212	0	3364
POLD3	0.00400187	0	3365
ZMYND10	0.00399975	0	3366
EHD3	0.00399599	0	3367
FOXN1	0.00398727	0	3368
BRCC3	0.00398005	0	3369
SHROOM3	0.00397789	0	3370
RARRES1	0.00397687	0	3371
RUVBL1	0.00397508	0	3372
STH	0.00397263	0	3373
OSBPL3	0.0039726	0	3374
SYNJ2	0.00397245	0	3375
HAX1	0.00397018	0	3376
MOS	0.00396854	0	3377
DDX60	0.00396334	0	3378
SDC3	0.0039626	0	3379
RSPO3	0.00395658	0	3380
NKG7	0.00395086	0	3381
KCTD15	0.00394868	0	3382
HSCB	0.00393832	0	3383
DNAJA1	0.00393801	0	3384
FUT2	0.00393622	0	3385
LILRA3	0.00393593	0	3386
C19ORF10	0.003935	0	3387
LECT2	0.00393393	0	3388
SEL1L	0.00393382	0	3389
UGT1A9	0.00393204	0	3390
PILRA	0.00393066	0	3391
SNX18	0.00392998	0	3392

BRI3	0.00392771	0	3393
UBE2L6	0.00392635	0	3394
KRT3	0.00392395	0	3395
ATP6V1E1	0.00392179	0	3396
UGT1A3	0.00392146	0	3397
CYP2C18	0.00392087	0	3398
DDIAS	0.00392043	0	3399
COLQ	0.0039169	0	3400
ATOH8	0.00391364	0	3401
BCAM	0.00390927	0	3402
PTOV1	0.00390814	0	3403
NFYB	0.00390265	0	3404
SNAI1	0.00389981	0	3405
RAB3A	0.00389564	0	3406
NR1D2	0.00389486	0	3407
CASP10	0.00389479	0	3408
PAM16	0.00389104	0	3409
RAB33B	0.00388645	0	3410
SEC63	0.00388622	0	3411
GALT	0.0038849	0	3412
ARL13B	0.00388257	0	3413
KIR2DS2	0.003882	0	3414
NFKBIB	0.00387887	0	3415
MIR573	0.00387017	0	3416
MRPS7	0.00386976	0	3417
SLC22A2	0.00386719	0	3418
PHIP	0.00386633	0	3419
EHD1	0.00386504	0	3420
GLRX5	0.00386148	0	3421
HLX	0.00385235	0	3422
POSTN	0.00384749	0	3423
ASIC1	0.00384252	0	3424
MYBBP1A	0.00384113	0	3425
MIR145	0.00384023	0	3426
SUZ12	0.00383424	0	3427
GTF2A2	0.0038334	0	3428
HTATIP2	0.00383022	0	3429
SFRP2	0.00382967	0	3430
PIP	0.00382845	0	3431
PACSIN1	0.00382428	0	3432
GTF2F1	0.00382333	0	3433
SNIP1	0.00382207	0	3434
DPP9	0.00381781	0	3435

FOXD1	0.00381532	0	3436
GJB3	0.00381204	0	3437
PLVAP	0.00380563	0	3438
CPN2	0.00380561	0	3439
ADCY7	0.00380469	0	3440
UTS2R	0.00380469	0	3441
KDM3B	0.00380403	0	3442
CHRD	0.00380129	0	3443
ATG4C	0.00380026	0	3444
ARHGEF9	0.00379443	0	3445
MMVP1	0.00379424	0	3446
KALRN	0.00379051	0	3447
GSTT2	0.0037889	0	3448
RYK	0.00378513	0	3449
TNS4	0.00377996	0	3450
CTBS	0.00377906	0	3451
AFAP1L2	0.0037705	0	3452
PAK6	0.00377024	0	3453
MBD3	0.00375935	0	3454
RA3	0.00375897	0	3455
ARHGAP9	0.00375882	0	3456
COX6B1	0.00375882	0	3457
OSBPL10	0.00375882	0	3458
PLEK2	0.00375882	0	3459
PLEKHA3	0.00375882	0	3460
PLEKHA5	0.00375882	0	3461
PLEKHA6	0.00375882	0	3462
PLEKHB2	0.00375882	0	3463
TBC1D2	0.00375882	0	3464
ELMO2	0.0037577	0	3465
FAM172A	0.00375387	0	3466
KPNA5	0.00375387	0	3467
SLIT3	0.00374581	0	3468
PDE1B	0.00374515	0	3469
RAB9A	0.00374111	0	3470
FKBP8	0.00373954	0	3471
LRP1B	0.00373953	0	3472
HOXB9	0.00373942	0	3473
HSPB9	0.00373874	0	3474
CDC42EP5	0.00372607	0	3475
MAML3	0.00372342	0	3476
MIR576	0.00371879	0	3477
DNAJC3	0.00371745	0	3478

MSRA	0.00371348	0	3479
RPS14	0.00371304	0	3480
GSTA2	0.0037103	0	3481
SLC7A8	0.00370924	0	3482
RALGAPB	0.0037088	0	3483
ODAM	0.00370859	0	3484
BNIP2	0.00370494	0	3485
PHKA2	0.00369988	0	3486
IPO7	0.00369662	0	3487
SLC20A2	0.00369483	0	3488
NAPA	0.00369432	0	3489
POGLUT1	0.00369066	0	3490
CEP68	0.00369028	0	3491
TYMS	0.00368979	0	3492
GP2	0.00368781	0	3493
MEF2B	0.00368603	0	3494
NUDT1	0.0036852	0	3495
RPS20	0.00368159	0	3496
MYCBP	0.00367906	0	3497
ACVR2A	0.00367903	0	3498
GPRC5A	0.00367731	0	3499
PTPN18	0.003677	0	3500
TECR	0.00367307	0	3501
MT-TG	0.0036723	0	3502
ZNF667	0.00366773	0	3503
PGM3	0.00366647	0	3504
SIRPG	0.00366577	0	3505
CORD1	0.00366458	0	3506
ST5	0.00366407	0	3507
GDI2	0.00366093	0	3508
GPR56	0.00365781	0	3509
PARP14	0.00365562	0	3510
COL4A3BP	0.00365418	0	3511
ARHGAP32	0.00365399	0	3512
SPNS1	0.00365371	0	3513
UGP2	0.0036495	0	3514
RFPL3	0.00364822	0	3515
TAC3	0.00364721	0	3516
CHN1	0.00364349	0	3517
RAB17	0.00363911	0	3518
LRIG3	0.00363679	0	3519
CCPG1	0.00362857	0	3520
FIMG1	0.00362475	0	3521

SM2	0.00362435	0	3522
SERPING1	0.00362381	0	3523
ITPR2	0.00362279	0	3524
LAMP5	0.00362098	0	3525
SNX33	0.0036164	0	3526
COPG1	0.00361405	0	3527
PSS	0.00361387	0	3528
NT5C3	0.0036134	0	3529
ALKBH3	0.00361031	0	3530
ACAP2	0.00360742	0	3531
NAALADL2	0.00360742	0	3532
MLIP	0.00360246	0	3533
IFNL4	0.00360095	0	3534
GPS1	0.00360017	0	3535
AIDA	0.00359928	0	3536
SH3GL3	0.00359887	0	3537
DUSP22	0.00359846	0	3538
SUCNR1	0.00359818	0	3539
VDAC2	0.00359763	0	3540
PAX8	0.00359658	0	3541
HNRNPF	0.00359579	0	3542
RPL23A	0.00359075	0	3543
RAB36	0.00359006	0	3544
GM2A	0.00358431	0	3545
EDA2R	0.00357717	0	3546
NRP2	0.00357473	0	3547
SLIT1	0.0035717	0	3548
ESRRG	0.0035708	0	3549
HFM	0.00356756	0	3550
ANAPC7	0.00356438	0	3551
PHACTR1	0.00356297	0	3552
S100A7	0.00356277	0	3553
UNC119	0.0035548	0	3554
ANGPT4	0.00355225	0	3555
SCGB3A2	0.0035517	0	3556
TPM4	0.00355018	0	3557
CAPNS2	0.00354726	0	3558
PAK1IP1	0.00354617	0	3559
SLC36A2	0.00354585	0	3560
HSD17B10	0.00353431	0	3561
MAP1S	0.00353369	0	3562
UPK1B	0.00353065	0	3563
HLA-DRA	0.00352749	0	3564

MIR765	0.00352646	0	3565
PPA2	0.00352646	0	3566
TOR1AIP1	0.00352646	0	3567
CHP1	0.00352349	0	3568
RCC1	0.00352128	0	3569
PES1	0.00351945	0	3570
HSPA7	0.00351929	0	3571
KLK15	0.00351733	0	3572
LMNB1	0.00351573	0	3573
KIF5A	0.00351456	0	3574
PIEZO1	0.00351162	0	3575
CD99L2	0.00351083	0	3576
CUL7	0.00351033	0	3577
ARF3	0.00350881	0	3578
MIR340	0.00350845	0	3579
EIF4G1	0.00350697	0	3580
ADCY2	0.00349523	0	3581
RASA3	0.00349304	0	3582
GGA1	0.00349251	0	3583
PCP4	0.00348913	0	3584
UGT2B7	0.00348671	0	3585
EIF3B	0.00348283	0	3586
FECH	0.00348273	0	3587
COL8A2	0.00348118	0	3588
CLCA4	0.00348115	0	3589
TULP1	0.00347391	0	3590
MARK3	0.00347108	0	3591
CNTN1	0.00346999	0	3592
TST	0.00346984	0	3593
PLA2G10	0.00346972	0	3594
ARHGAP21	0.00346731	0	3595
RELN	0.0034658	0	3596
VPS41	0.00345402	0	3597
SLC7A10	0.00344735	0	3598
IL17RC	0.0034473	0	3599
LRRC4B	0.00344453	0	3600
PPIP5K1	0.0034421	0	3601
PSMC3	0.00343817	0	3602
ATP2B4	0.00343205	0	3603
NPHP4	0.00343116	0	3604
KCNG1	0.00343114	0	3605
SNORD3@	0.00343079	0	3606
SNRPB	0.00343061	0	3607

SPEN	0.00342618	0	3608
MAGEA3	0.00342516	0	3609
FBN1	0.00342488	0	3610
GUCY2C	0.00341894	0	3611
PPP1R14B	0.00341316	0	3612
HOXB2	0.00341251	0	3613
ETV1	0.00340918	0	3614
CDC42BPA	0.00340889	0	3615
GPHN	0.00340502	0	3616
HEY	0.00340306	0	3617
STK16	0.00339866	0	3618
UBL3	0.00339861	0	3619
ANAPC1	0.00339764	0	3620
AKR1B10	0.00339735	0	3621
TRNAK2	0.00339682	0	3622
VRK2	0.00339638	0	3623
HOXC13	0.00339522	0	3624
UBE2H	0.00339103	0	3625
OR4G3P	0.0033896	0	3626
CFB	0.00338757	0	3627
PLA2G15	0.00338725	0	3628
CNKSR1	0.00338336	0	3629
MIR34C	0.00338167	0	3630
CA6	0.00337885	0	3631
IGHMBP2	0.00335557	0	3632
EEF1A2	0.00335211	0	3633
IL28RA	0.00335116	0	3634
DUSP16	0.00335034	0	3635
RCE1	0.0033493	0	3636
EAF2	0.0033474	0	3637
SLC51A	0.00334509	0	3638
PTCH2	0.00334433	0	3639
FAIM3	0.00334103	0	3640
NID1	0.00333986	0	3641
CRADD	0.00333817	0	3642
CLINT1	0.00333593	0	3643
CGNL1	0.00333501	0	3644
PNKD	0.0033334	0	3645
KIR2DL2	0.00333322	0	3646
TES	0.00332266	0	3647
BVES	0.00332165	0	3648
LTK	0.00331938	0	3649
PLXNB2	0.00331742	0	3650

HRK	0.00330794	0	3651
EIF3C	0.00330651	0	3652
PRPF31	0.00329958	0	3653
C21ORF33	0.00329838	0	3654
APOC2	0.00329756	0	3655
HLA-DOB	0.00329078	0	3656
PPAN	0.00328934	0	3657
ARAP1	0.00328773	0	3658
TRAPPC9	0.00328567	0	3659
ATP2A1	0.00328189	0	3660
WNT6	0.00328137	0	3661
MLF1	0.00327981	0	3662
PDCD2	0.00327923	0	3663
EEF1E1	0.00327876	0	3664
CYB5A	0.00327663	0	3665
CLDN2	0.00327585	0	3666
ZYX	0.00327359	0	3667
GSTM1	0.00327331	0	3668
CCNT1	0.00326858	0	3669
RAB40C	0.00326702	0	3670
TRIP4	0.00326652	0	3671
CTSC	0.00326501	0	3672
IGFBP6	0.00326416	0	3673
AUTS2	0.00326198	0	3674
COPS4	0.00325844	0	3675
DLG1	0.00325809	0	3676
CASC5	0.0032488	0	3677
HBBP1	0.00324826	0	3678
NT5C	0.00324723	0	3679
YEATS4	0.00324666	0	3680
MYCL1	0.00324654	0	3681
ARFGEF2	0.00324586	0	3682
SERPINB4	0.00324553	0	3683
TRIM54	0.00324452	0	3684
ARHGEF3	0.00324389	0	3685
CDK2AP1	0.00324192	0	3686
UBQLN1	0.00324065	0	3687
TOMM22	0.0032389	0	3688
PSMD13	0.00323731	0	3689
RAB2A	0.00323646	0	3690
MYL7	0.0032328	0	3691
CNOT7	0.00323262	0	3692
USF1	0.0032321	0	3693

CRMP1	0.00323135	0	3694
ATP2B3	0.00323026	0	3695
USP19	0.00322646	0	3696
CEP70	0.00322618	0	3697
DHH	0.00321899	0	3698
FAM188A	0.00321535	0	3699
EBNA1BP2	0.00321399	0	3700
TNFAIP8L1	0.00321389	0	3701
F9	0.00321363	0	3702
PAK7	0.00321179	0	3703
PDIA3	0.00320986	0	3704
UBA2	0.00320788	0	3705
HLA-B	0.00320039	0	3706
ZNF346	0.00319973	0	3707
IL17RE	0.00319318	0	3708
AMBRA1	0.00318925	0	3709
SLC6A7	0.00318595	0	3710
TMEFF1	0.00318446	0	3711
PIP4K2A	0.00318049	0	3712
INHA	0.00317969	0	3713
VAX2	0.00317375	0	3714
MIR337	0.003169	0	3715
HES3	0.00316788	0	3716
CHGB	0.00316695	0	3717
FAM72A	0.00316606	0	3718
CIR1	0.00316586	0	3719
OR5I1	0.00316396	0	3720
PTPRD	0.00315974	0	3721
DEFA1	0.00315752	0	3722
ABLIM1	0.00315642	0	3723
PRSS3P2	0.00315489	0	3724
STON2	0.00315489	0	3725
NLRP7	0.00315368	0	3726
ATAD3A	0.00314982	0	3727
NLRP2	0.00314962	0	3728
SLC25A22	0.00314879	0	3729
ADRA1A	0.00314685	0	3730
RANBP1	0.0031462	0	3731
CSDE1	0.00314289	0	3732
APOBEC1	0.00314053	0	3733
CYP2S1	0.00313521	0	3734
GLDC	0.0031352	0	3735
ATP8A2	0.00313483	0	3736

KCNQ2	0.00313272	0	3737
ODF2	0.00313214	0	3738
KIAA1598	0.00312835	0	3739
ASB2	0.00312313	0	3740
SAP25	0.00312313	0	3741
LYPD5	0.00312116	0	3742
ASH1L	0.0031205	0	3743
BCAS1	0.00311981	0	3744
MPP2	0.00311957	0	3745
SRCAP	0.00311687	0	3746
ATP5J2	0.00311661	0	3747
ELL2	0.00311476	0	3748
FOXA3	0.00311265	0	3749
HERC2	0.00311197	0	3750
CA8	0.00311111	0	3751
TRIB1	0.00310882	0	3752
BW56	0.00310835	0	3753
WLS	0.00310832	0	3754
JDP2	0.00310831	0	3755
AA1	0.00310646	0	3756
NOTUM	0.00310464	0	3757
MLLT6	0.00309955	0	3758
COL18A1	0.00309951	0	3759
ELK3	0.00309887	0	3760
NAV2	0.00309656	0	3761
SLC25A10	0.00309506	0	3762
RBBP5	0.00309384	0	3763
DPP7	0.00308971	0	3764
MICALL1	0.00308835	0	3765
POLR1E	0.00308538	0	3766
EOS	0.00308435	0	3767
ASAH1	0.00308133	0	3768
LSM4	0.00307985	0	3769
FAM134B	0.00307952	0	3770
PAN3	0.00307701	0	3771
ATAT1	0.00307693	0	3772
TMEM201	0.00307245	0	3773
S100A6	0.00306847	0	3774
ZDHHC7	0.00306795	0	3775
ZNF35	0.00306719	0	3776
TFE3	0.00306276	0	3777
PCBD1	0.00306157	0	3778
EPB41L4B	0.00306078	0	3779

PDGFD	0.00306022	0	3780
KDM5B	0.00305946	0	3781
RNF25	0.00305887	0	3782
LYPLA2	0.00305832	0	3783
TNS1	0.00305743	0	3784
CD164	0.00305726	0	3785
SNORA73A	0.00305681	0	3786
SMIM1	0.00305657	0	3787
CDAN1	0.00305495	0	3788
PAX1	0.00305428	0	3789
IGHV3-21	0.00305047	0	3790
TIPRL	0.0030465	0	3791
MANF	0.00304573	0	3792
CHST4	0.00304365	0	3793
ZBP1	0.00304278	0	3794
POLR2G	0.00304041	0	3795
LGALS8	0.00303925	0	3796
MAML1	0.00303663	0	3797
SERINC5	0.00303638	0	3798
PRMT8	0.00303549	0	3799
RTN2	0.0030342	0	3800
SERPINH1	0.00303269	0	3801
FOXO4	0.00303181	0	3802
REC8	0.00303086	0	3803
SPAG17	0.00302826	0	3804
CIAO1	0.00302633	0	3805
ASH2L	0.00302576	0	3806
RASGRF2	0.00302534	0	3807
HSPBP1	0.00302507	0	3808
OSGEP	0.00302163	0	3809
MT4	0.00301761	0	3810
PHRF1	0.00301577	0	3811
UPK2	0.00301427	0	3812
CD2BP2	0.00301013	0	3813
NR4A3	0.00300981	0	3814
HES2	0.00300932	0	3815
PEX5	0.0030072	0	3816
DDX19A	0.00300614	0	3817
SNORD50B	0.00300614	0	3818
AGAP4	0.00300498	0	3819
FOXRED2	0.0030049	0	3820
SLC51B	0.00300343	0	3821
CORO7	0.00300196	0	3822

DUSP2	0.00300183	0	3823
CNPY4	0.00299958	0	3824
LBX2	0.00299946	0	3825
PDE4A	0.00299927	0	3826
DFNB31	0.00299917	0	3827
ARHGAP8	0.00299296	0	3828
SLC9A7	0.00298986	0	3829
PLB1	0.00298627	0	3830
MIS12	0.00298562	0	3831
DEAF1	0.0029853	0	3832
ERVK-18	0.00298287	0	3833
L3MBTL1	0.0029787	0	3834
CCT4	0.0029781	0	3835
FCRL2	0.00297583	0	3836
DMTN	0.00297289	0	3837
OPN1LW	0.00297258	0	3838
STK17B	0.00296853	0	3839
LDB1	0.00296535	0	3840
DGCR8	0.00296381	0	3841
RTKN	0.00296289	0	3842
TECRL	0.00295931	0	3843
MOK	0.0029561	0	3844
HERC5	0.00295467	0	3845
MIR198	0.00295424	0	3846
SLC9A3R2	0.00295393	0	3847
GLG1	0.0029537	0	3848
APCDD1	0.00295203	0	3849
MIMT1	0.00295172	0	3850
GPX8	0.00295165	0	3851
CCL18	0.00295092	0	3852
HPCA	0.0029502	0	3853
SYTL2	0.00294945	0	3854
PQBP1	0.00294699	0	3855
PBCA	0.00294317	0	3856
BTN2A2	0.0029418	0	3857
MIR648	0.00294164	0	3858
ITPA	0.00293951	0	3859
RYR2	0.00293812	0	3860
RGS7BP	0.00293611	0	3861
POM121	0.00293589	0	3862
DHS	0.00293535	0	3863
ZDHHC17	0.00293505	0	3864
MLLT11	0.00293493	0	3865

IRAK1BP1	0.00292974	0	3866
ELAVL3	0.00292818	0	3867
PDSS2	0.00292757	0	3868
GFM1	0.00292655	0	3869
EPB42	0.0029253	0	3870
SUMO4	0.00292363	0	3871
ANK3	0.00292298	0	3872
CRLF1	0.00292207	0	3873
POU4F1	0.00291863	0	3874
TCF7L2	0.00291725	0	3875
CDKAL1	0.00291653	0	3876
FHOD3	0.00291638	0	3877
PNMA6A	0.00291377	0	3878
ZNF22	0.00291049	0	3879
HSD17B7	0.00290603	0	3880
TMC8	0.00290575	0	3881
NDUFC2	0.0029052	0	3882
LYPD3	0.00290077	0	3883
PKD3	0.00289881	0	3884
GTF2E1	0.00289781	0	3885
MAP3K10	0.00289595	0	3886
SLC35A2	0.00289294	0	3887
RTP4	0.00289098	0	3888
LARS	0.00289038	0	3889
IQCG	0.00288906	0	3890
KIAA0586	0.00288759	0	3891
GGA3	0.00288655	0	3892
EFTUD2	0.00288629	0	3893
AP3B1	0.00288525	0	3894
OTUB1	0.00288485	0	3895
DOCK4	0.00288338	0	3896
AFF3	0.00288162	0	3897
EIF4E2	0.0028791	0	3898
BBC3	0.00287823	0	3899
NEK4	0.00287778	0	3900
CLN3	0.00287672	0	3901
ENO3	0.00287609	0	3902
NXT1	0.00287481	0	3903
PVRL1	0.00287434	0	3904
TRAF7	0.00287405	0	3905
PLEKHG5	0.00287209	0	3906
CNDP2	0.0028685	0	3907
RPL3	0.00286825	0	3908

ATP4A	0.00286801	0	3909
IRF6	0.00286514	0	3910
SPECC1	0.00286409	0	3911
JPH3	0.0028593	0	3912
RASSF7	0.00285817	0	3913
HECTD2	0.00285743	0	3914
ZNF133	0.00285743	0	3915
GAPVD1	0.00285314	0	3916
GDF11	0.00285208	0	3917
EIF3E	0.00285095	0	3918
MIXL1	0.0028469	0	3919
CALM3	0.00284684	0	3920
CENPB	0.0028447	0	3921
VIM2P	0.00284316	0	3922
MASTL	0.00283983	0	3923
GPR161	0.00283811	0	3924
SLC7A9	0.00283527	0	3925
RNF4	0.00283499	0	3926
CYP2C8	0.00283443	0	3927
KRTAP3-4P	0.00283318	0	3928
NDUFB7	0.00282964	0	3929
GSG1L	0.00282585	0	3930
MIR4493	0.00282585	0	3931
TST1	0.00282585	0	3932
ZNF568	0.00282585	0	3933
CEP135	0.00282539	0	3934
CD163L1	0.00282417	0	3935
PNPO	0.00282234	0	3936
MLXIP	0.00282084	0	3937
SLC9A6	0.00282048	0	3938
MICA	0.00281902	0	3939
RPL7	0.00281811	0	3940
PSMB9	0.00281541	0	3941
KRT39	0.00280952	0	3942
AOPEP	0.00280894	0	3943
PRDX4	0.00280713	0	3944
TNFRSF10C	0.00280675	0	3945
PIDD	0.00280655	0	3946
NIT1	0.00280622	0	3947
BARX2	0.00280587	0	3948
SCAF1	0.0028027	0	3949
DHRS11	0.00280209	0	3950
PDE3B	0.00279913	0	3951

ARL6	0.00279908	0	3952
DOCK11	0.0027975	0	3953
UGT1A8	0.00279713	0	3954
ASGR1	0.00279529	0	3955
CETN1	0.00279518	0	3956
LMAN1	0.00279001	0	3957
DOCK9	0.00278885	0	3958
MLPH	0.00278781	0	3959
TBC1D3	0.00278765	0	3960
GPR15	0.00278658	0	3961
SNTG1	0.00278031	0	3962
NUCB1	0.00277613	0	3963
DEPDC1	0.00277598	0	3964
VAX1	0.00277414	0	3965
MUC20	0.00276906	0	3966
IPO13	0.00276747	0	3967
UGT1A4	0.00276742	0	3968
G6PD	0.00276688	0	3969
GATC	0.00276461	0	3970
PRNT	0.00276333	0	3971
SCGB1A1	0.00276256	0	3972
PDHA2	0.00275843	0	3973
POLA1	0.00275221	0	3974
CFL1	0.00275123	0	3975
CRYGC	0.00274822	0	3976
CENPK	0.00274784	0	3977
GCNT1	0.00274773	0	3978
CHMP6	0.00274675	0	3979
AK2	0.00274585	0	3980
GPR37	0.00274529	0	3981
KIF5C	0.00274293	0	3982
TEX10	0.00273497	0	3983
RBFOX1	0.00273395	0	3984
MIR127	0.00273174	0	3985
CYP26B1	0.00273111	0	3986
SNHG5	0.00273073	0	3987
RPL30	0.00272818	0	3988
KIF18A	0.00272697	0	3989
HPSE2	0.00272606	0	3990
EN1	0.00272554	0	3991
HRM2	0.00272496	0	3992
JKAMP	0.0027179	0	3993
IGHD6-13	0.00271732	0	3994

NDEL1	0.00271352	0	3995
HLA-DMB	0.00271207	0	3996
ELSPBP1	0.00270885	0	3997
SLC22A8	0.00270844	0	3998
PTAFR	0.00270346	0	3999
NAF1	0.00270269	0	4000
RA1	0.00269978	0	4001
PRSS3	0.00269886	0	4002
MS	0.00269514	0	4003
TWF2	0.00269434	0	4004
PROS1	0.00269354	0	4005
CABP1	0.00269128	0	4006
BRMS1	0.00269008	0	4007
MED25	0.00269008	0	4008
SYS1	0.00268896	0	4009
STAB1	0.00268731	0	4010
DDX54	0.00268621	0	4011
ZNF85	0.00268541	0	4012
PPOX	0.00268519	0	4013
KIDINS220	0.00268501	0	4014
CNPY3	0.00268498	0	4015
BRPF1	0.00268469	0	4016
AKAP10	0.00268323	0	4017
STK4	0.00268199	0	4018
HPS1	0.00268181	0	4019
SIN3A	0.00268137	0	4020
DDR1	0.00267849	0	4021
MYOM2	0.00267641	0	4022
PKHD1	0.00267562	0	4023
ASB4	0.00267281	0	4024
ONECUT1	0.00267009	0	4025
PTGFRN	0.00266993	0	4026
TLL1	0.00266802	0	4027
CLTB	0.00266608	0	4028
PEX3	0.00266365	0	4029
ISL1	0.00265541	0	4030
NAB1	0.00265237	0	4031
SYNPO2	0.00265217	0	4032
GSDMD	0.00265207	0	4033
TBC1D10A	0.00265057	0	4034
HIVEP3	0.00264705	0	4035
TBC1D24	0.00264562	0	4036
TRMT1	0.00264491	0	4037

HECTD3	0.00264198	0	4038
LHX3	0.00264187	0	4039
CCHCR1	0.00264165	0	4040
PAX6	0.00264153	0	4041
LIM2	0.00263951	0	4042
AP2M1	0.00263655	0	4043
MAPK8IP2	0.00263466	0	4044
MYO5A	0.00263383	0	4045
TOB1	0.00263292	0	4046
FRYL	0.00262897	0	4047
ZC3H13	0.00262897	0	4048
ZFYVE19	0.00262897	0	4049
CFSS	0.00262652	0	4050
MCC	0.0026261	0	4051
IFIT5	0.00262579	0	4052
FSHB	0.00262443	0	4053
MIB1	0.00262373	0	4054
MYO1F	0.00262127	0	4055
USP11	0.00261873	0	4056
SPR	0.002616	0	4057
TAF15	0.0026146	0	4058
PGAP2	0.00261354	0	4059
SEMA6D	0.00261323	0	4060
SURF6	0.00261283	0	4061
STOML3	0.0026125	0	4062
SSH1	0.00261249	0	4063
ITGB1BP1	0.00260955	0	4064
ADAP1	0.00260896	0	4065
SLC15A4	0.00260857	0	4066
SLC7A11	0.00260379	0	4067
ADAT2	0.00260182	0	4068
EP400	0.00260156	0	4069
EXOC6	0.00260111	0	4070
KMT2E	0.00260032	0	4071
RGMA	0.00259874	0	4072
GLB1	0.00259811	0	4073
SLC22A4	0.00259726	0	4074
HES5	0.00259693	0	4075
IL18RAP	0.00259421	0	4076
SPANXA1	0.00259413	0	4077
OCIAD1	0.00259035	0	4078
OTP	0.00258874	0	4079
CTR9	0.00258721	0	4080

ENGASE	0.00258563	0	4081
CAPZB	0.00258549	0	4082
LPHN1	0.00258501	0	4083
CNGA1	0.00258388	0	4084
TTN	0.00258386	0	4085
H1F0	0.00258176	0	4086
BCL2L13	0.00258094	0	4087
CLEC14A	0.00258085	0	4088
RAX	0.00257903	0	4089
DMTF1	0.00257707	0	4090
GC	0.00257577	0	4091
SH3BGRL	0.00257543	0	4092
FBXW2	0.00257377	0	4093
CNTLN	0.00257198	0	4094
P2RY13	0.00257037	0	4095
SCAR2	0.00256934	0	4096
RAB6B	0.00256659	0	4097
MUCL1	0.0025651	0	4098
PPFIA3	0.00256262	0	4099
FETUB	0.00256255	0	4100
KCNK13	0.00256168	0	4101
CYP2A13	0.00255941	0	4102
TFIP11	0.00255544	0	4103
FGF6	0.00255178	0	4104
MYOZ2	0.0025503	0	4105
SLC18A1	0.00254968	0	4106
TMC6	0.00254961	0	4107
UQCC2	0.00254653	0	4108
CHD4	0.00254301	0	4109
MUC21	0.00253937	0	4110
ADAM8	0.00253724	0	4111
RXRG	0.00253573	0	4112
HAND1	0.00253535	0	4113
WBP2	0.00253439	0	4114
PRSS8	0.002532	0	4115
SP8	0.00253025	0	4116
MZF1	0.00252871	0	4117
ZFHX3	0.00252589	0	4118
EGLN1	0.00252444	0	4119
HADH	0.00252281	0	4120
PPP1R10	0.00252229	0	4121
IPMK	0.00251851	0	4122
ERGIC2	0.00251834	0	4123

PLSCR4	0.00251781	0	4124
NKTR	0.00251753	0	4125
CD97	0.00251649	0	4126
ST3GAL5	0.00251594	0	4127
PPP1R7	0.00251167	0	4128
EPC1	0.00250883	0	4129
MRF	0.00250744	0	4130
GDE1	0.0025057	0	4131
FOXJ1	0.00250563	0	4132
SUPV3L1	0.00250533	0	4133
IFT172	0.00250361	0	4134
MT-CO2	0.00250226	0	4135
PHF2	0.00250188	0	4136
ANKRD17	0.00250095	0	4137
KIAA0226	0.00249796	0	4138
NOXO1	0.00249733	0	4139
SATB1	0.00249726	0	4140
RRAD	0.00249431	0	4141
GEMIN6	0.00249429	0	4142
ANXA8	0.0024941	0	4143
RLS	0.0024931	0	4144
PDZK1IP1	0.00249277	0	4145
CRABP1	0.00249165	0	4146
PAX9	0.00248717	0	4147
ARF4	0.00248416	0	4148
PIAS2	0.00248352	0	4149
EMP1	0.00247986	0	4150
TRAT1	0.00247701	0	4151
RASSF6	0.00247506	0	4152
RNU4ATAC	0.00247452	0	4153
GLYCAM1	0.00247354	0	4154
DHX15	0.00246993	0	4155
SLC45A2	0.00246988	0	4156
NEUROG3	0.00246982	0	4157
PDCL3	0.00246669	0	4158
MIR342	0.00245887	0	4159
IL11RA	0.0024581	0	4160
LRRC10	0.00245676	0	4161
PCBP1	0.00245561	0	4162
FLNC	0.00245262	0	4163
OR10A4	0.00244917	0	4164
SS18L1	0.002449	0	4165
FIG4	0.00244852	0	4166

AD5	0.00244782	0	4167
CSHL1	0.00244768	0	4168
CFP	0.00244607	0	4169
ACTN1	0.00244487	0	4170
SNURF	0.00244458	0	4171
RAB25	0.00244203	0	4172
LPHN3	0.00243911	0	4173
HHIP	0.00243619	0	4174
FTX	0.00243361	0	4175
MIR545	0.00243361	0	4176
DCD	0.00243302	0	4177
ZNF77	0.00243202	0	4178
UBP1	0.00242994	0	4179
LACC1	0.00242601	0	4180
LYPD6	0.00242601	0	4181
MYO1G	0.00242385	0	4182
LPAL2	0.00242362	0	4183
HOXA13	0.00242334	0	4184
HNRNPH3	0.00242151	0	4185
WIPF2	0.00242022	0	4186
MIIP	0.00242014	0	4187
SMYD3	0.00241864	0	4188
NDUFA5	0.00241844	0	4189
EXOSC5	0.00241666	0	4190
RTN4R	0.0024141	0	4191
MYO5C	0.00241174	0	4192
MICALL2	0.0024103	0	4193
LRRFIP1	0.00240733	0	4194
SRPK2	0.00240729	0	4195
RNU6-1	0.00240207	0	4196
PHF13	0.00240199	0	4197
AD10	0.00240093	0	4198
DEL13Q14	0.00240046	0	4199
TMEM158	0.00239968	0	4200
FRMPD2	0.00239898	0	4201
KMT2D	0.0023964	0	4202
RNASE7	0.0023955	0	4203
PNMA1	0.0023944	0	4204
TRNAA1	0.00239381	0	4205
CDKN2D	0.00239278	0	4206
PCDHB3	0.0023903	0	4207
EDAR	0.00238936	0	4208
PPP1R8	0.00238892	0	4209

ARSB	0.00238775	0	4210
CRTC3	0.00238615	0	4211
CCNB3	0.00238478	0	4212
CORIN	0.00238131	0	4213
NAP1L1	0.00238061	0	4214
SNTA1	0.00238029	0	4215
PDGFA	0.00237924	0	4216
FST	0.00237874	0	4217
EPHX4	0.00237867	0	4218
ZNF268	0.00237396	0	4219
CHST14	0.00237092	0	4220
CDK19	0.0023704	0	4221
WFDC1	0.00237029	0	4222
TBPL2	0.00236692	0	4223
BCKDHB	0.00236248	0	4224
VAMP3	0.00236159	0	4225
FAM83H	0.00236115	0	4226
FGF19	0.00236032	0	4227
WIPI1	0.00235964	0	4228
UBE2Q1	0.0023589	0	4229
KRT32	0.00235811	0	4230
LAMA5	0.00235703	0	4231
RNASEK	0.00235125	0	4232
RPL29	0.00234279	0	4233
SDE2	0.00234265	0	4234
ZNRF1	0.00234265	0	4235
SPNS2	0.00234122	0	4236
SPRR2A	0.00234062	0	4237
YTHDC2	0.00234059	0	4238
SSTR4	0.00233577	0	4239
GTF2H5	0.00233444	0	4240
8-Mar	0.00233145	0	4241
GPHA2	0.00233075	0	4242
METTL1	0.00233035	0	4243
ASD1	0.0023284	0	4244
CHCHD2	0.0023284	0	4245
TBX6	0.00232733	0	4246
WISP1	0.00232621	0	4247
ATP2B1	0.00232601	0	4248
C9ORF3	0.00231941	0	4249
ASCC3	0.00231782	0	4250
SLC26A1	0.00231371	0	4251
RIT2	0.00231028	0	4252

ELAVL1	0.00230925	0	4253
OTULIN	0.0023078	0	4254
NACC1	0.00230775	0	4255
NAV1	0.00230448	0	4256
TAOK1	0.00230423	0	4257
RAP2B	0.00230198	0	4258
NCKIPSD	0.0022993	0	4259
GSTM5	0.00229809	0	4260
RPL17	0.00229571	0	4261
KCNIP2	0.00229451	0	4262
SAG	0.00229334	0	4263
ZNF197	0.00229301	0	4264
SBDS	0.00229268	0	4265
DSC1	0.00229023	0	4266
SYNCRIP	0.00229019	0	4267
ATAD2	0.00228935	0	4268
MT-ND1	0.00228876	0	4269
BIRC6	0.00228586	0	4270
LHB	0.00228341	0	4271
HLA-F	0.00228106	0	4272
L3MBTL2	0.00228055	0	4273
FBXO15	0.00227938	0	4274
KDELR1	0.00227875	0	4275
MBD3L2	0.00227807	0	4276
UBXN6	0.00227657	0	4277
HEG1	0.00227463	0	4278
KCNN3	0.0022746	0	4279
UBE2O	0.00227152	0	4280
NCR3LG1	0.00227103	0	4281
PTBP2	0.00227063	0	4282
WARS	0.00226931	0	4283
MDC1	0.0022668	0	4284
AD12	0.00226661	0	4285
FHL3	0.00226399	0	4286
TOX4	0.00226331	0	4287
MIR193A	0.00226309	0	4288
PTPMT1	0.00226167	0	4289
GBF1	0.00226164	0	4290
PAK3	0.00225804	0	4291
NCOA4	0.00225484	0	4292
FCP1	0.00225474	0	4293
SIVA1	0.002254	0	4294
GRHL2	0.00225093	0	4295

BTBD9	0.00225036	0	4296
RGL1	0.00224996	0	4297
DEDD2	0.00224995	0	4298
ADCY5	0.00224797	0	4299
CCDC115	0.00224788	0	4300
CYGB	0.00224602	0	4301
AHSA2	0.00224465	0	4302
ANP32C	0.00224465	0	4303
NCAPD2	0.00224465	0	4304
SUGT1P1	0.00224465	0	4305
PIWIL2	0.00224457	0	4306
ITIH3	0.00224176	0	4307
RPS6KB2	0.00224121	0	4308
TACC1	0.00224021	0	4309
ERMAP	0.00223696	0	4310
TYRL	0.00223696	0	4311
THRA	0.00223681	0	4312
SNORD86	0.00223635	0	4313
PTRH2	0.00223412	0	4314
AMOTL1	0.00223291	0	4315
IGHJ@	0.00223255	0	4316
MFF	0.00222981	0	4317
GALR2	0.00222959	0	4318
NOP16	0.00222622	0	4319
RBMS1	0.00222598	0	4320
IBD2	0.00222435	0	4321
EFNA4	0.00222402	0	4322
CCR8	0.00222384	0	4323
REG4	0.00222102	0	4324
GPC1	0.00221739	0	4325
GGH	0.00221533	0	4326
BMPR1B	0.00221522	0	4327
PPARD	0.00221416	0	4328
RPPH1	0.00220692	0	4329
PLXNA4	0.00220402	0	4330
IL1RAPL1	0.00219877	0	4331
DNAJA2	0.00219827	0	4332
MIR134	0.00219701	0	4333
ST3GAL2	0.00219233	0	4334
MIA3	0.00219019	0	4335
LURAP1	0.00218557	0	4336
TSSK4	0.0021847	0	4337
LIX1L	0.0021831	0	4338

ANKRD27	0.00218198	0	4339
ARSC2	0.00217988	0	4340
RPL39	0.00217988	0	4341
NAA50	0.00217981	0	4342
KIF26B	0.00217741	0	4343
HAO1	0.00217638	0	4344
DNAJC14	0.00217191	0	4345
RGMB	0.0021702	0	4346
PTPN21	0.00217003	0	4347
SCYL3	0.00217003	0	4348
KRT28	0.00216805	0	4349
MCRS1	0.00216681	0	4350
SF3B2	0.00216632	0	4351
TRBV30	0.00216533	0	4352
TIFA	0.00216445	0	4353
CDX4	0.00216407	0	4354
ZNF202	0.0021633	0	4355
AOC1	0.00216243	0	4356
CLA3	0.00216151	0	4357
UOX	0.00216113	0	4358
RSU1	0.00216013	0	4359
GER	0.00215959	0	4360
COX11	0.00215926	0	4361
CLSTN2	0.0021547	0	4362
PPP1R3B	0.00215374	0	4363
NFKBIE	0.00215122	0	4364
TNFAIP8L2	0.00215035	0	4365
GDF1	0.00215009	0	4366
ROMO1	0.00214858	0	4367
SLC22A18	0.00214574	0	4368
TBC1D17	0.00214474	0	4369
SLCO1B1	0.00214231	0	4370
INHBC	0.00214158	0	4371
MIR216B	0.00213998	0	4372
DIP2A	0.00213995	0	4373
PRUNE	0.00213994	0	4374
ARFRP1	0.00213638	0	4375
C19ORF70	0.00213586	0	4376
NBEAL2	0.00213468	0	4377
DYRK1B	0.00213199	0	4378
GINGF2	0.00213191	0	4379
TRDD3	0.00213167	0	4380
HPRT1	0.00213151	0	4381

APOC3	0.00213041	0	4382
SLC25A32	0.00212958	0	4383
TNIP2	0.00212897	0	4384
NUP88	0.00212747	0	4385
GTF2H2	0.00212332	0	4386
GNRHR	0.00212161	0	4387
HSPA14	0.00212158	0	4388
PSRC1	0.0021201	0	4389
TFPT	0.00211969	0	4390
CCDC12	0.00211849	0	4391
PAPOLG	0.00211845	0	4392
WNT11	0.00211727	0	4393
SLC37A4	0.00211428	0	4394
BTN2A1	0.00211414	0	4395
KHDRBS2	0.00211184	0	4396
CRTC2	0.00211018	0	4397
MAP3K9	0.00210989	0	4398
ZKSCAN3	0.00210839	0	4399
UGDH	0.0021068	0	4400
DUOXA2	0.00210664	0	4401
LAIR1	0.0021059	0	4402
MT-TK	0.00210563	0	4403
ND4L	0.00210462	0	4404
SPOP	0.00210277	0	4405
BLOC1S2	0.00210243	0	4406
RUFY1	0.00210218	0	4407
RRS1	0.00210111	0	4408
MT-TH	0.0021	0	4409
MTPN	0.00209946	0	4410
H3F3B	0.00209875	0	4411
PBX3	0.00209847	0	4412
C9ORF72	0.0020974	0	4413
AAGAB	0.0020973	0	4414
RAB28	0.00209658	0	4415
CREB3L4	0.00209524	0	4416
AACS	0.0020951	0	4417
RADIL	0.0020914	0	4418
FGL1	0.0020867	0	4419
PPL	0.00208608	0	4420
MAU2	0.0020843	0	4421
RN7SL1	0.00208426	0	4422
NUP153	0.00208281	0	4423
UNC13D	0.00208134	0	4424

ADRA2A	0.00207946	0	4425
PAN2	0.00207516	0	4426
RNH1	0.00207481	0	4427
ASXL2	0.00207367	0	4428
TSHB	0.00207041	0	4429
TEAD2	0.00206875	0	4430
GCK	0.00206746	0	4431
CTRC	0.00206691	0	4432
FAR1	0.00206683	0	4433
HIST1H3A	0.00206541	0	4434
PHKA1	0.00206494	0	4435
UQCRFS1	0.00206478	0	4436
CT60	0.00206283	0	4437
ANE1	0.00205894	0	4438
CAMK1	0.00205891	0	4439
SLC12A8	0.00205855	0	4440
SMARCE1	0.00205689	0	4441
CPO	0.0020566	0	4442
RAB34	0.00205523	0	4443
IL22RA2	0.00205409	0	4444
OBSL1	0.00204813	0	4445
ABHD2	0.0020477	0	4446
EIF3F	0.00204512	0	4447
UQCRC2	0.00204488	0	4448
IVD	0.00204449	0	4449
SH3GL2	0.00204115	0	4450
ARPC1B	0.00204097	0	4451
RUFY3	0.00204097	0	4452
XRCC6	0.00203905	0	4453
SLC34A1	0.00203862	0	4454
IDDM4	0.00203713	0	4455
ZNHIT3	0.00203713	0	4456
RPL10A	0.00203616	0	4457
UST	0.0020347	0	4458
RPL22	0.00203289	0	4459
SYPL1	0.0020288	0	4460
RAB39A	0.00202321	0	4461
APOF	0.00202315	0	4462
PLAGL1	0.00202149	0	4463
PRAF2	0.00202117	0	4464
SOX10	0.00201837	0	4465
MARCO	0.00201704	0	4466
MKL2	0.00201316	0	4467

UTF1	0.00201247	0	4468
EARS2	0.00201093	0	4469
ZFP90	0.00201037	0	4470
IGKV3D-15	0.00200933	0	4471
PRDM8	0.0020078	0	4472
BCLAF1	0.00200562	0	4473
RNFT1	0.00200419	0	4474
ZFP91	0.00200419	0	4475
NR2F2	0.00200417	0	4476
WHSC1	0.00200346	0	4477
FAT1	0.00200271	0	4478
ATXN2L	0.00200174	0	4479
PTPN7	0.00200084	0	4480
EXOC3	0.0020008	0	4481
ERF	0.00199685	0	4482
MIR126	0.00199448	0	4483
DPT	0.00199406	0	4484
TRDN	0.00199394	0	4485
BOK	0.00199374	0	4486
SLC29A4	0.0019927	0	4487
INSC	0.00199116	0	4488
RILP	0.00199047	0	4489
MAPKAPK5	0.0019891	0	4490
SLCO2A1	0.00198745	0	4491
SLC22A16	0.00198692	0	4492
SENP1	0.00198503	0	4493
ART5	0.00198442	0	4494
MOXD1	0.00198437	0	4495
HRASLS5	0.00198416	0	4496
IRF2BP1	0.00198416	0	4497
RHOC	0.00198299	0	4498
VANGL1	0.00198186	0	4499
TRNAL1	0.00198183	0	4500
MESP1	0.00197818	0	4501
VMP1	0.00197798	0	4502
KREMEN2	0.00197795	0	4503
MIR128-2	0.00197668	0	4504
SHISA6	0.00197553	0	4505
NAGA	0.00197543	0	4506
RFX2	0.00197331	0	4507
PLEKHG4	0.0019726	0	4508
B3GNT2	0.00196963	0	4509
BDP1	0.00196958	0	4510

DNAJC2	0.00196934	0	4511
KIF15	0.00196679	0	4512
GHS	0.00196648	0	4513
HYOU1	0.00196528	0	4514
RYR3	0.0019647	0	4515
BCRP1	0.00196343	0	4516
DET1	0.00196028	0	4517
CRYBB2	0.00195952	0	4518
KLHL20	0.00195741	0	4519
PIK3R5	0.00195653	0	4520
PIK3R6	0.00195653	0	4521
EID1	0.00195582	0	4522
NEK1	0.00195517	0	4523
ATP1A3	0.0019548	0	4524
SLC16A3	0.00195284	0	4525
CASC3	0.00195168	0	4526
NASP	0.00195051	0	4527
CLEC4M	0.00195023	0	4528
XPO4	0.00194339	0	4529
MYL12A	0.00193958	0	4530
MKNK2	0.00193906	0	4531
GABPAP	0.00193896	0	4532
CCL4L1	0.00193871	0	4533
BTC	0.00193385	0	4534
NMT2	0.00193242	0	4535
MESDC2	0.00193189	0	4536
DYNC1H1	0.00193013	0	4537
TCL1A	0.0019283	0	4538
MARK1	0.0019268	0	4539
CASP5	0.00192205	0	4540
SENP5	0.00192009	0	4541
RNF6	0.00191986	0	4542
BGN	0.00191627	0	4543
SMARCC1	0.00191553	0	4544
CDH23	0.00191532	0	4545
CHM	0.00191324	0	4546
ZASP	0.0019125	0	4547
HSBP1	0.00190971	0	4548
PAGE2	0.0019089	0	4549
SDPR	0.00190881	0	4550
MYF6	0.00190688	0	4551
PRSS12	0.00190523	0	4552
RPS9	0.00190283	0	4553

RPS17	0.0019028	0	4554
SDCBP2	0.00190247	0	4555
PLS3	0.00190056	0	4556
DIMT1	0.00189803	0	4557
RBP4	0.00189665	0	4558
TROVE2	0.00189574	0	4559
AGGF1	0.00189548	0	4560
CAPN10	0.00189541	0	4561
SEC62	0.00189491	0	4562
MIR326	0.00189468	0	4563
LNP1	0.00189337	0	4564
DDX25	0.00189325	0	4565
APOBEC3C	0.00189288	0	4566
HIF1AN	0.00189187	0	4567
UBE2F	0.00189147	0	4568
ABO	0.00188938	0	4569
ATXN8	0.00188913	0	4570
TCERG1	0.00188913	0	4571
MPC1	0.00188821	0	4572
USP6	0.00188798	0	4573
KLF14	0.001886	0	4574
PDHA1	0.00188492	0	4575
IL20RB	0.00188355	0	4576
C3ORF58	0.0018833	0	4577
ELN	0.00188303	0	4578
RAB11FIP1	0.00188266	0	4579
IGKV1D-43	0.00188196	0	4580
OMP	0.0018809	0	4581
SNX5	0.00187913	0	4582
TOM1L2	0.00187702	0	4583
INO80C	0.00187367	0	4584
ACTR3C	0.00187201	0	4585
DGUOK	0.00187197	0	4586
NYX	0.00187013	0	4587
HPS3	0.00186898	0	4588
MYP3	0.0018682	0	4589
ARSI	0.00186779	0	4590
MBNL2	0.00186723	0	4591
TRIM3	0.00186483	0	4592
FCRL1	0.00186429	0	4593
ARHGEF5	0.00186313	0	4594
ATG3P1	0.00186219	0	4595
BP8	0.00186219	0	4596

DEM1	0.00186219	0	4597
FASA	0.00186219	0	4598
SERF1A	0.00186219	0	4599
SNORD79	0.00186219	0	4600
TCP11	0.00186219	0	4601
POP4	0.0018621	0	4602
RPP21	0.0018621	0	4603
RPP30	0.0018621	0	4604
DHX8	0.00186116	0	4605
WDFY3	0.00186034	0	4606
MDN1	0.0018583	0	4607
GALC	0.00185816	0	4608
PELO	0.00185745	0	4609
OLIG1	0.0018564	0	4610
CXORF21	0.00185554	0	4611
CYR61	0.00185413	0	4612
ZNF350	0.00185315	0	4613
SFXN2	0.00185181	0	4614
USMG5	0.00185181	0	4615
3-Mar	0.00184794	0	4616
NDUFAB1	0.00184686	0	4617
AIFM2	0.00184163	0	4618
CYP2A6	0.00183914	0	4619
STX11	0.00183874	0	4620
BP14	0.00183744	0	4621
BP36	0.00183744	0	4622
PHF23	0.00183699	0	4623
KLHDC2	0.00183679	0	4624
ARHGEF16	0.00183203	0	4625
UCHL3	0.00183153	0	4626
UBE2A	0.00183107	0	4627
CYC1	0.0018309	0	4628
ATF6B	0.00183085	0	4629
IL17RD	0.00182934	0	4630
SH2D4A	0.00182555	0	4631
ITPK1	0.00182444	0	4632
ADD2	0.00182416	0	4633
RAET1L	0.00182382	0	4634
RGL3	0.00182259	0	4635
SPDL1	0.00182115	0	4636
KLHL22	0.00181975	0	4637
PISD	0.00181957	0	4638
TOX3	0.00181943	0	4639

SRP68	0.00181739	0	4640
TMEM134	0.00181737	0	4641
SORCS2	0.00181381	0	4642
CNTN4	0.00181371	0	4643
DESI2	0.00181246	0	4644
FCN1	0.00181106	0	4645
AAT1	0.00180928	0	4646
SCIMP	0.00180887	0	4647
RPS6KA6	0.00180831	0	4648
HAUS3	0.00180587	0	4649
C16ORF80	0.00180518	0	4650
GPR78	0.00180512	0	4651
RNU1-1	0.00180461	0	4652
GSTO2	0.0018041	0	4653
HOXC6	0.00180393	0	4654
ATP6V0A4	0.00180209	0	4655
HS3ST1	0.00180051	0	4656
PSPN	0.00179957	0	4657
ZDHHC19	0.00179768	0	4658
VSX1	0.0017972	0	4659
FARS2	0.00179604	0	4660
C5AR2	0.00179455	0	4661
CTBP1	0.00179436	0	4662
TAF8	0.0017937	0	4663
NDUFA2	0.00179247	0	4664
VPS25	0.00179243	0	4665
CLCN1	0.00179242	0	4666
SEC13	0.00178983	0	4667
TMPRSS11D	0.00178918	0	4668
GCLM	0.00178845	0	4669
RIC8A	0.00178772	0	4670
LCOR	0.00178698	0	4671
MCCC2	0.00178659	0	4672
FABP9	0.00178646	0	4673
GPR18	0.00178273	0	4674
PIH1D1	0.00178264	0	4675
PELI2	0.00178234	0	4676
PRKAR2B	0.00178015	0	4677
ND5	0.00178009	0	4678
DDX20	0.00177981	0	4679
C2CD4A	0.00177967	0	4680
C2CD4B	0.00177967	0	4681
MICU1	0.00177912	0	4682

ARHGAP11A	0.00177309	0	4683
DGKQ	0.00177309	0	4684
MEOX1	0.00177034	0	4685
NEXN	0.00176987	0	4686
CERKL	0.00176854	0	4687
AAR2	0.0017619	0	4688
ARHGAP15	0.00176118	0	4689
ENOPH1	0.00176043	0	4690
PRDX1	0.00175838	0	4691
CD109	0.00175516	0	4692
WT4	0.00175024	0	4693
EGFR-AS1	0.00174973	0	4694
TRAPPC1	0.00174907	0	4695
AURKC	0.00174868	0	4696
GOSR2	0.00174532	0	4697
GRASP	0.00174499	0	4698
SNORD126	0.00174499	0	4699
RPL35	0.00174493	0	4700
BEX2	0.00174315	0	4701
RP24	0.00174293	0	4702
PDE4DIP	0.00174256	0	4703
GPR50	0.00174209	0	4704
ILVBL	0.00174166	0	4705
RAB21	0.00173889	0	4706
DDX39A	0.00173705	0	4707
TBX22	0.00173697	0	4708
VPRBP	0.00173667	0	4709
OPRK1	0.00173605	0	4710
DDX19B	0.00173532	0	4711
SLC52A3	0.00173134	0	4712
BRAT1	0.00173025	0	4713
PSG2	0.0017287	0	4714
AKR1C1	0.00172728	0	4715
RPL21	0.00172603	0	4716
PNPT1	0.00172533	0	4717
AKR7A2	0.00172345	0	4718
NFE2L1	0.00172229	0	4719
CORO2A	0.00171934	0	4720
IFNGR2	0.00171847	0	4721
DZIP3	0.00171758	0	4722
EHD2	0.00171695	0	4723
XRCC5	0.00171592	0	4724
PDB1	0.00171543	0	4725

DHX33	0.00171303	0	4726
AKR1C4	0.0017123	0	4727
RPL10	0.001712	0	4728
KLF11	0.00171049	0	4729
MIR183	0.00171006	0	4730
MIR212	0.00170991	0	4731
TRIM50	0.00170975	0	4732
SRP72	0.0017097	0	4733
TPH1	0.00170657	0	4734
LY6D	0.00170605	0	4735
CLEC2D	0.00170595	0	4736
SYNJ1	0.00170531	0	4737
STYXL1	0.00170507	0	4738
PSMD14	0.00170494	0	4739
DNAJA3	0.00170443	0	4740
6-Sep	0.00170107	0	4741
GNAT1	0.00170025	0	4742
NKRF	0.00170014	0	4743
NDC1	0.00169386	0	4744
IQSEC2	0.00169301	0	4745
ALRH	0.00169143	0	4746
MINK1	0.00169003	0	4747
FBXO21	0.00168992	0	4748
LHX4	0.00168512	0	4749
CASP14	0.00168461	0	4750
FMN1	0.0016828	0	4751
DAD1	0.00168275	0	4752
NME1-NME2	0.00168211	0	4753
NMS	0.0016819	0	4754
CHMP4A	0.00167951	0	4755
NEGR1	0.00167701	0	4756
LEFTY1	0.00166981	0	4757
BBIP1	0.00166863	0	4758
UBAP2L	0.00166741	0	4759
TPST1	0.00166741	0	4760
DOT1L	0.00166694	0	4761
DCBLD2	0.00166576	0	4762
HTN3	0.0016656	0	4763
HS3ST2	0.00166468	0	4764
NT5C3A	0.00166464	0	4765
UNC45A	0.0016642	0	4766
ZMIZ2	0.00166388	0	4767
UEVLD	0.00166326	0	4768

CDH17	0.00166205	0	4769
IL17B	0.00166184	0	4770
KREMEN1	0.00165893	0	4771
CYP3A43	0.00165709	0	4772
ASB6	0.00165203	0	4773
TERF1	0.0016516	0	4774
WDR20	0.00165125	0	4775
GCM2	0.00165044	0	4776
MOB1B	0.00164934	0	4777
PRR3	0.00164797	0	4778
NUCKS1	0.00164653	0	4779
UBE2S	0.00164576	0	4780
ILKAP	0.00164099	0	4781
ULBP1	0.00163987	0	4782
EMC3	0.00163891	0	4783
CCDC141	0.00163864	0	4784
ARFIP1	0.00163728	0	4785
SPINT1	0.00163533	0	4786
RNF146	0.00163384	0	4787
KIAA0391	0.00163371	0	4788
PCSK9	0.00163357	0	4789
ALYREF	0.00163346	0	4790
C4A	0.0016321	0	4791
SERINC3	0.00162933	0	4792
RPIA	0.00162793	0	4793
KRT24	0.001626	0	4794
CASQ2	0.001622	0	4795
UGT1A5	0.00162067	0	4796
PPP1R3A	0.0016201	0	4797
ARHGAP26	0.00161798	0	4798
LSL	0.00161696	0	4799
NHS	0.00161675	0	4800
UCP1	0.00161665	0	4801
STK38L	0.00161564	0	4802
TCEB2	0.00161496	0	4803
MIR559	0.00161439	0	4804
AP2A1	0.00161219	0	4805
SERP1	0.00161149	0	4806
MIA	0.00161091	0	4807
GULP1	0.00160955	0	4808
TUBB	0.0016071	0	4809
MIR193B	0.00160409	0	4810
SDF2	0.00160329	0	4811

FBXO11	0.00160293	0	4812
SLC7A7	0.00160258	0	4813
NAPG	0.00160027	0	4814
EMR3	0.0015976	0	4815
CCDC59	0.00159385	0	4816
INSL4	0.00159271	0	4817
ISX	0.00159262	0	4818
SPG20	0.00159252	0	4819
PLEKHA2	0.00158949	0	4820
AEBP1	0.00158851	0	4821
LYRM4	0.00158839	0	4822
KCNK1	0.00158837	0	4823
SLC44A1	0.00158794	0	4824
SEC31A	0.00158708	0	4825
UGT1A10	0.00158707	0	4826
HLA-DRB3	0.00158662	0	4827
HDX	0.00158616	0	4828
PARP4	0.00158456	0	4829
MAPKAPK3	0.00158329	0	4830
EIM	0.00158301	0	4831
SNRPF	0.00158113	0	4832
HS1BP3	0.00158109	0	4833
RTN3	0.00158058	0	4834
EIF2C2	0.00158041	0	4835
CYP2D7P1	0.00158011	0	4836
FBXO3	0.00157901	0	4837
CTAG2	0.0015788	0	4838
MTF1	0.00157734	0	4839
TMEM216	0.00157601	0	4840
MIR502	0.00157515	0	4841
FXYD3	0.001575	0	4842
CELSR1	0.00157413	0	4843
TAF3	0.00157374	0	4844
CA10	0.00157315	0	4845
LRRFIP2	0.00157231	0	4846
ADRB1	0.00157057	0	4847
CDB2	0.00156969	0	4848
MAPRE1	0.00156958	0	4849
NSD1	0.00156942	0	4850
GBX2	0.00156702	0	4851
XPO5	0.00156571	0	4852
BDH1	0.00156435	0	4853
STK19	0.0015629	0	4854

ETV7	0.00156183	0	4855
TPD52	0.0015617	0	4856
CAPZA1	0.0015591	0	4857
VNN2	0.00155874	0	4858
IGKV3-11	0.00155749	0	4859
BMP3	0.00155598	0	4860
FBXO17	0.00155516	0	4861
FEM1A	0.00155516	0	4862
KCTD20	0.00155516	0	4863
SHMT2	0.00155442	0	4864
KIF13B	0.00155369	0	4865
PJA1	0.0015518	0	4866
SUGP1	0.00155162	0	4867
TCOF1	0.00154973	0	4868
CDCA2	0.00154905	0	4869
CFHR4	0.00154882	0	4870
CEBPD	0.00154682	0	4871
ZNF382	0.00154256	0	4872
FUT5	0.00154203	0	4873
SIN3B	0.00154137	0	4874
FGF23	0.00154115	0	4875
CYP4F2	0.00153912	0	4876
NPAT	0.00153903	0	4877
BRD9	0.0015382	0	4878
INCA1	0.00153755	0	4879
TSPAN8	0.00153685	0	4880
GAGE12G	0.00153664	0	4881
C14ORF159	0.00153589	0	4882
NUP205	0.00153468	0	4883
NEK9	0.00153278	0	4884
LRRK1	0.00153219	0	4885
SETX	0.00153212	0	4886
FAM129B	0.00153139	0	4887
ECHDC1	0.00153077	0	4888
DNAJC13	0.0015254	0	4889
NOTCH4	0.00152388	0	4890
ABCA7	0.00152312	0	4891
IGKV3-31	0.00152049	0	4892
STARD3	0.00152046	0	4893
LIMS1	0.00152007	0	4894
TRIM7	0.00151664	0	4895
SSU72	0.00151629	0	4896
TEAD4	0.00151608	0	4897

QKI	0.00151602	0	4898
FLNB	0.00151489	0	4899
CR1L	0.00151486	0	4900
SGOL1	0.00151384	0	4901
BDH2	0.00151348	0	4902
DENND3	0.00151254	0	4903
DYNC1LI2	0.00151254	0	4904
PSD3	0.00151254	0	4905
LGALS7	0.00151047	0	4906
IGKV2D-26	0.00150919	0	4907
MTM1	0.00150812	0	4908
CLDN9	0.00150726	0	4909
FAM58A	0.00150371	0	4910
GR6	0.00150371	0	4911
MAFD1	0.00150371	0	4912
PCDHGB3	0.00150371	0	4913
SNORD21	0.00150371	0	4914
DDX46	0.0014979	0	4915
SH2D3C	0.00149764	0	4916
MIR1236	0.00149721	0	4917
GOSR1	0.00149685	0	4918
TSR1	0.00149677	0	4919
CACYBP	0.00149665	0	4920
DGKE	0.00149522	0	4921
HIST1H1T	0.00149352	0	4922
ALS7	0.0014924	0	4923
HOXA6	0.00149128	0	4924
KRT1	0.00148962	0	4925
CDR2	0.00148955	0	4926
SOX11	0.00148932	0	4927
PSMD3	0.00148748	0	4928
GTF3C1	0.00148608	0	4929
IMUP	0.00148509	0	4930
SLC17A1	0.00148434	0	4931
KIRREL3	0.00148193	0	4932
SIGLEC14	0.00148191	0	4933
SGCE	0.00148148	0	4934
PSMD1	0.00148124	0	4935
FGF12	0.00147809	0	4936
PKD1P2	0.00147558	0	4937
CENPW	0.00147449	0	4938
DIAPH2	0.00147365	0	4939
PPIB	0.00147289	0	4940

SUDS3	0.00146911	0	4941
PTP4A2	0.00146831	0	4942
SP110	0.00146689	0	4943
CHCHD4	0.00146592	0	4944
AIPL1	0.00146512	0	4945
DMRT3	0.00146324	0	4946
PMF1	0.00146274	0	4947
KLHL1	0.0014624	0	4948
DEFA3	0.00146198	0	4949
CRTC1	0.00146159	0	4950
FRAT1	0.00145949	0	4951
SNX2	0.00145948	0	4952
MIR99B	0.00145769	0	4953
EFNA3	0.00145617	0	4954
HYPK	0.00145505	0	4955
CTSS	0.00145453	0	4956
HECA	0.0014509	0	4957
WNT10A	0.00145074	0	4958
SPTAN1	0.00144824	0	4959
RAPGEF2	0.00144773	0	4960
GDF9	0.00144748	0	4961
SLC22A5	0.00144713	0	4962
DCXR	0.00144581	0	4963
APELA	0.00144575	0	4964
ADARB2	0.00144501	0	4965
ABCC6	0.00144492	0	4966
DNAJC21	0.00144468	0	4967
MOB4	0.00144232	0	4968
SOST	0.00143986	0	4969
BAG4	0.00143923	0	4970
CITED1	0.00143894	0	4971
CFAP97	0.00143715	0	4972
YIF1A	0.00143663	0	4973
ADAM15	0.00143256	0	4974
AHSP	0.00143126	0	4975
IFT57	0.0014296	0	4976
TESK1	0.0014269	0	4977
GNAI2	0.00142689	0	4978
SLC19A2	0.00142645	0	4979
PTP4A1	0.00142567	0	4980
SWI5	0.00142506	0	4981
MIR186	0.00142477	0	4982
PTGDS	0.00142455	0	4983

PRKACG	0.00142405	0	4984
PSMB6	0.00142381	0	4985
APEX1	0.00142356	0	4986
M1	0.00142295	0	4987
CLK3	0.00141983	0	4988
YIPF5	0.00141983	0	4989
SVIL	0.0014196	0	4990
TADA2A	0.00141717	0	4991
UBA6	0.00141223	0	4992
COL11A2	0.00141177	0	4993
RNF216	0.00141151	0	4994
NRTN	0.00140447	0	4995
NLN	0.00140332	0	4996
DDX39B	0.00140189	0	4997
CUL5	0.00140151	0	4998
SDTY1	0.00140006	0	4999
NEURL1	0.00139952	0	5000
NEURL1B	0.00139952	0	5001
TPCN2	0.00139925	0	5002
FAAP20	0.00139917	0	5003
EHBP1	0.00139847	0	5004
ANKRD28	0.00139729	0	5005
GYPE	0.00139615	0	5006
MS2	0.00139253	0	5007
MPZ	0.00139164	0	5008
MYPN	0.00139085	0	5009
DGAT2L6	0.00139082	0	5010
ATP5A1	0.00139053	0	5011
GSTM3	0.00139027	0	5012
BPIFA2	0.00138976	0	5013
PCCB	0.00138843	0	5014
KRT38	0.00138658	0	5015
FAM210B	0.00138643	0	5016
RAB13	0.00138584	0	5017
HSD17B8	0.00138531	0	5018
HDAC11	0.00138483	0	5019
INSRR	0.00138413	0	5020
THTPA	0.00138347	0	5021
CRX	0.00138228	0	5022
RNF115	0.00138151	0	5023
WHSC1L1	0.00138126	0	5024
CHID1	0.0013808	0	5025
CYP20A1	0.00137869	0	5026

PTRF	0.00137863	0	5027
RBM42	0.0013762	0	5028
LRCH1	0.00137539	0	5029
TREML1	0.00137459	0	5030
SLC30A4	0.00137422	0	5031
GJA8	0.00137294	0	5032
UBE2D3	0.00137242	0	5033
AIC	0.00137155	0	5034
PMPCA	0.0013706	0	5035
GALNT14	0.00137026	0	5036
FAM3C	0.00136784	0	5037
MID2	0.00136695	0	5038
GEMIN2	0.00136292	0	5039
ARAFP2	0.0013612	0	5040
PXYLP1	0.0013612	0	5041
DKKL1	0.00135959	0	5042
NFE2L3	0.0013593	0	5043
APPL2	0.00135917	0	5044
ESAT	0.00135905	0	5045
GLYR1	0.00135897	0	5046
EXOC4	0.00135895	0	5047
NR1H2	0.00135849	0	5048
OSR2	0.00135776	0	5049
CA12	0.00135478	0	5050
CRB3	0.00135419	0	5051
DCK	0.00135412	0	5052
UBE2W	0.00135402	0	5053
BAG5	0.00135401	0	5054
C11ORF73	0.00135401	0	5055
DNAJB11	0.00135401	0	5056
GCNT3	0.00135401	0	5057
TOMM34	0.00135401	0	5058
XPNPEP1	0.00135009	0	5059
RARB	0.00134905	0	5060
MYO7A	0.00134863	0	5061
SCX	0.00134738	0	5062
CYP4A11	0.00134732	0	5063
MOSPD1	0.00134701	0	5064
OAZ1	0.00134351	0	5065
UTP11L	0.00134333	0	5066
TACR3	0.00133943	0	5067
SMPD2	0.00133817	0	5068
ITGB3BP	0.00133757	0	5069

LRFN3	0.00133593	0	5070
PLAC1	0.00133347	0	5071
OPN3	0.00133316	0	5072
SMYD1	0.00133259	0	5073
DOCK7	0.00132918	0	5074
PLCB4	0.00132888	0	5075
OLA1	0.0013287	0	5076
TMEM2	0.00132847	0	5077
MEIS3	0.00132841	0	5078
MCCC1	0.00132773	0	5079
DAAM1	0.0013276	0	5080
ATF5	0.00132755	0	5081
ZDHHC3	0.00132745	0	5082
SRL	0.00132552	0	5083
MIR495	0.0013255	0	5084
CD177P1	0.00132392	0	5085
CNOT8	0.00132175	0	5086
GOPC	0.00132057	0	5087
EPB49	0.00131915	0	5088
RIOK1	0.00131855	0	5089
CSRNP1	0.00131646	0	5090
RAB3GAP2	0.00131492	0	5091
CNOT3	0.00131283	0	5092
NADK	0.0013127	0	5093
NAA25	0.00131173	0	5094
REXO1L1	0.00131061	0	5095
KDM1A	0.00130991	0	5096
HDAC10	0.00130977	0	5097
FGG	0.00130872	0	5098
IGKV3-15	0.00130764	0	5099
KCNMB1	0.00130568	0	5100
STXBP5L	0.00130553	0	5101
ZRSR2	0.00130537	0	5102
PROP1	0.00130509	0	5103
ZBTB33	0.00130472	0	5104
EXO5	0.00130252	0	5105
CELIAC2	0.0013015	0	5106
PHF5A	0.00130055	0	5107
MICB	0.00129968	0	5108
TPP2	0.00129801	0	5109
XDH	0.00129731	0	5110
C1GALT1C1	0.00129423	0	5111
BIN2	0.001294	0	5112

CKAP2L	0.00129338	0	5113
MYH7	0.00128993	0	5114
KIF21A	0.00128973	0	5115
GOLGA3	0.00128924	0	5116
MEI1	0.00128623	0	5117
ZKSCAN7	0.00128618	0	5118
KLHL12	0.00128574	0	5119
GMDS	0.00128412	0	5120
ADSSL1	0.00128196	0	5121
TWSG1	0.00127987	0	5122
ADRM1	0.00127978	0	5123
PCDH15	0.00127858	0	5124
GAS5-AS1	0.00127814	0	5125
ATP7B	0.00127779	0	5126
DHX9	0.00127746	0	5127
RGS7	0.00127738	0	5128
ALS3	0.00127611	0	5129
TFEB	0.00127569	0	5130
CYP2J2	0.00127295	0	5131
LBH	0.00127234	0	5132
CHRNG	0.00127194	0	5133
CPT1B	0.00127147	0	5134
MRRF	0.00126945	0	5135
CREB3	0.00126726	0	5136
PLS1	0.00126668	0	5137
IGHDOR15@	0.0012665	0	5138
LYPLA1	0.00126478	0	5139
CER1	0.00126278	0	5140
FRMD6	0.00126275	0	5141
CCT8	0.00126264	0	5142
IPO4	0.00125944	0	5143
GPX7	0.00125823	0	5144
RNF114	0.00125812	0	5145
AD14	0.00125745	0	5146
SON	0.00125276	0	5147
CYP11A1	0.00125128	0	5148
LRRN3	0.00125089	0	5149
MYL6	0.00125038	0	5150
MIR382	0.0012475	0	5151
G3BP2	0.00124538	0	5152
CYP2U1	0.00124482	0	5153
GPER1	0.0012448	0	5154
KIN	0.00124447	0	5155

ANK1	0.00124367	0	5156
PLK3	0.00124333	0	5157
GPC3	0.00124308	0	5158
COPD	0.0012412	0	5159
CCV	0.00123468	0	5160
TRAPPC10	0.00123449	0	5161
UBA3	0.00123437	0	5162
GBE1	0.00123315	0	5163
CORO1A	0.00123246	0	5164
CDC20P1	0.00123131	0	5165
BUB3	0.0012311	0	5166
DDX56	0.00123061	0	5167
PI4KB	0.00122969	0	5168
RCOR1	0.00122858	0	5169
STK11IP	0.00122725	0	5170
MYBPH	0.00122716	0	5171
TPBG	0.00122529	0	5172
SLC25A37	0.00122203	0	5173
GPIHBP1	0.00122125	0	5174
CDH22	0.00121963	0	5175
TBX20	0.00121873	0	5176
SPTLC2	0.00121818	0	5177
TRG	0.00121742	0	5178
SIAH2	0.00121547	0	5179
S1PR5	0.00121389	0	5180
PPP2R5E	0.00121379	0	5181
FLAD1	0.00121294	0	5182
BSN	0.00121253	0	5183
UGT2B4	0.00121202	0	5184
ARRB2	0.00121164	0	5185
ANAPC11	0.00121104	0	5186
EFCAB11	0.0012082	0	5187
HLA-DQB3	0.0012078	0	5188
STIL	0.00120725	0	5189
TRPC5	0.00120602	0	5190
USP30	0.00120262	0	5191
SLIRP	0.00120261	0	5192
ATG3	0.00120172	0	5193
CEACAM4	0.00119787	0	5194
UNC13B	0.00119742	0	5195
NUFIP1	0.0011951	0	5196
EMP2	0.00119456	0	5197
RPS6KA4	0.00119365	0	5198

SHMT1	0.00119319	0	5199
UBE2Q2	0.0011928	0	5200
NPDC1	0.00119082	0	5201
CEPT1	0.00119006	0	5202
EIF3J	0.00119006	0	5203
FITM2	0.00119006	0	5204
INSL6	0.00119006	0	5205
TOX2	0.00119001	0	5206
CNTN6	0.00118961	0	5207
KHDRBS3	0.00118876	0	5208
NMNAT2	0.00118773	0	5209
ARVCF	0.00118703	0	5210
TMPRSS13	0.00118621	0	5211
HIP1R	0.00118477	0	5212
FTSJ1	0.00118348	0	5213
ZNF79	0.00118322	0	5214
LEFTY2	0.00118034	0	5215
HIST1H1C	0.00117739	0	5216
GBD2	0.00117682	0	5217
PLP1	0.00117509	0	5218
MIR4498	0.00117197	0	5219
LBX1	0.00117147	0	5220
PPEF2	0.00117036	0	5221
TMSB10	0.00116947	0	5222
RFWD2	0.00116888	0	5223
GREM2	0.00116782	0	5224
TSC22D1	0.00116772	0	5225
MYH14	0.00116637	0	5226
NKX3-2	0.00116495	0	5227
DBC1	0.00116426	0	5228
SECISBP2L	0.00116424	0	5229
SFTA3	0.00116424	0	5230
PDR	0.00116345	0	5231
NUF2	0.00116122	0	5232
AGO3	0.00116058	0	5233
STX8	0.00115792	0	5234
NOP58	0.00115756	0	5235
EXOSC9	0.00115645	0	5236
TSPAN1	0.00115425	0	5237
THYN1	0.00115346	0	5238
EIF3L	0.00115307	0	5239
NDN	0.00115089	0	5240
KLHL41	0.00115038	0	5241

SPHK2	0.00114944	0	5242
ATP1B2	0.00114939	0	5243
RPGRIP1	0.001149	0	5244
VAMP7	0.00114749	0	5245
MORC1	0.00114484	0	5246
ZSCAN4	0.00114463	0	5247
PSMD4	0.00114448	0	5248
BCAS3	0.00114353	0	5249
TAS2R67P	0.00114216	0	5250
SNORD13P2	0.00114114	0	5251
NPEPPS	0.00114086	0	5252
EGFL7	0.00113913	0	5253
CRYBA4	0.001138	0	5254
LRRC31	0.0011374	0	5255
CA2	0.00113483	0	5256
MAP1LC3C	0.00113475	0	5257
SPATA13	0.00113466	0	5258
RNR4	0.00113455	0	5259
TBC1D14	0.0011345	0	5260
USP1	0.0011343	0	5261
PRKCI	0.00113225	0	5262
HPS5	0.00113214	0	5263
HRTRT1	0.00112881	0	5264
NUP155	0.00112703	0	5265
FBXW8	0.00112695	0	5266
VIPAS39	0.00112611	0	5267
SNAPC4	0.00112476	0	5268
BPIFA1	0.00112378	0	5269
EIF3G	0.00112259	0	5270
CAPN11	0.0011219	0	5271
PRCC	0.00112178	0	5272
FARSA	0.00112175	0	5273
PLEKHA7	0.00112112	0	5274
VPS4B	0.00111794	0	5275
FAM167A	0.00111774	0	5276
SOX5	0.00111732	0	5277
COL1A2	0.00111629	0	5278
REPS1	0.00111465	0	5279
FATE1	0.00111386	0	5280
EDIL3	0.00111162	0	5281
SPEG	0.00110956	0	5282
PTPRB	0.00110946	0	5283
GMCL1	0.00110762	0	5284

ZFYVE27	0.00110569	0	5285
IGBP1	0.00110564	0	5286
RFXANK	0.00110509	0	5287
LY86	0.00110498	0	5288
KLK5	0.00110472	0	5289
HPFH	0.00110242	0	5290
LXN	0.00110171	0	5291
NSDHL	0.00110164	0	5292
MLTK	0.00110149	0	5293
AFM	0.0011011	0	5294
PTPRS	0.00110058	0	5295
ZNF652	0.00110046	0	5296
DSG4	0.00110036	0	5297
ILF2	0.00109665	0	5298
AQP2	0.00109583	0	5299
ELOVL6	0.00109547	0	5300
VPS39	0.00109253	0	5301
IFT80	0.00109163	0	5302
FNTA	0.00108755	0	5303
EVA1A	0.00108222	0	5304
TRD	0.00108182	0	5305
ATG16L1	0.00107895	0	5306
YPEL4	0.0010785	0	5307
KTN1	0.00107828	0	5308
CMTM7	0.00107757	0	5309
FAM83A	0.00107757	0	5310
SCNN1D	0.00107652	0	5311
KIF20B	0.00107566	0	5312
CCDC25	0.00107353	0	5313
ZNF354C	0.00107324	0	5314
DPM3	0.00107152	0	5315
GEMIN4	0.00106793	0	5316
SCAF11	0.00106756	0	5317
KIF23	0.00106479	0	5318
SERPINA7	0.0010641	0	5319
FBXL12	0.00106215	0	5320
NSMAF	0.00106023	0	5321
EPHX1	0.00106021	0	5322
RSPO2	0.00105818	0	5323
ARL8B	0.0010578	0	5324
RNF187	0.0010575	0	5325
PLCZ1	0.00105559	0	5326
SPRY3	0.00105348	0	5327

OTOA	0.00105335	0	5328
FEM1C	0.00105001	0	5329
GNL3	0.0010488	0	5330
DAP3	0.00104548	0	5331
EGFL6	0.00104446	0	5332
BHLHE22	0.00104434	0	5333
DTX3L	0.00104328	0	5334
BTN1A1	0.00104312	0	5335
ATP6V0C	0.00104031	0	5336
SCN10A	0.00104003	0	5337
TIPARP	0.00103847	0	5338
FNBP1L	0.00103743	0	5339
RHD	0.00103701	0	5340
MED23	0.00103688	0	5341
TRIO	0.00103618	0	5342
KEL	0.00103524	0	5343
GTPBP1	0.00103503	0	5344
CIAPIN1	0.00103421	0	5345
SBF1	0.00103421	0	5346
SLC39A2	0.00103406	0	5347
DAZ1	0.00103294	0	5348
CKM	0.00103045	0	5349
TNFSF18	0.00102954	0	5350
PPP6R3	0.00102949	0	5351
SOX18	0.00102926	0	5352
SAP18	0.00102915	0	5353
JAM2	0.0010288	0	5354
MOB1A	0.00102524	0	5355
MKS1	0.00102498	0	5356
PCGF6	0.0010237	0	5357
KCNH7	0.00102276	0	5358
DLGAP5	0.00102161	0	5359
NCDN	0.00102044	0	5360
USP20	0.00101967	0	5361
PAPOLA	0.00101862	0	5362
FHL5	0.00101783	0	5363
GGCX	0.00101728	0	5364
ELP6	0.00101688	0	5365
AK3	0.00101648	0	5366
MANBA	0.00101568	0	5367
SLC5A1	0.00101502	0	5368
CYP7B1	0.00101363	0	5369
HBHR	0.00101282	0	5370

RGL2	0.00101278	0	5371
BLID	0.00101196	0	5372
RGS10	0.00101186	0	5373
SRPX2	0.00101164	0	5374
NKX6-3	0.00101055	0	5375
ZP2	0.00100927	0	5376
SPINK1	0.00100921	0	5377
MIR586	0.00100809	0	5378
GMFB	0.00100741	0	5379
DCUN1D1	0.00100711	0	5380
HCL1	0.00100705	0	5381
RPS21	0.00100688	0	5382
SIGLEC12	0.00100555	0	5383
PLOD1	0.00100553	0	5384
DENND1B	0.00100546	0	5385
CTSA	0.00100513	0	5386
CCL23	0.00100437	0	5387
CLEC11A	0.00100322	0	5388
PRIM1	0.00100322	0	5389
ZFP1	0.00100317	0	5390
SPIN1	0.00100296	0	5391
RBM17	0.0010025	0	5392
KATNB1	0.00100032	0	5393
WWP2	1.00E-03	0	5394
PAQR7	9.99E-04	0	5395
MRX8	9.99E-04	0	5396
NDST1	9.97E-04	0	5397
PRDM14	9.97E-04	0	5398
TRBV7-9	9.96E-04	0	5399
GMIP	9.94E-04	0	5400
TRIM61	9.94E-04	0	5401
GPR45	9.91E-04	0	5402
CACNA1C	9.91E-04	0	5403
MAP4K4	9.88E-04	0	5404
LMF1	9.86E-04	0	5405
FRAT2	9.85E-04	0	5406
CBX3	9.85E-04	0	5407
FRG1	9.85E-04	0	5408
MNAT1	9.84E-04	0	5409
GPR179	9.83E-04	0	5410
RFXAP	9.82E-04	0	5411
ATP1A4	9.81E-04	0	5412
DIRC2	9.79E-04	0	5413

MIR125B2	9.78E-04	0	5414
WNT9B	9.75E-04	0	5415
SELM	9.75E-04	0	5416
TRIM68	9.74E-04	0	5417
ELOVL5	9.74E-04	0	5418
EPB41L5	9.74E-04	0	5419
SNW1	9.73E-04	0	5420
2-Mar	9.73E-04	0	5421
HCAR1	9.73E-04	0	5422
IGHD3-3	9.72E-04	0	5423
OR1K1	9.72E-04	0	5424
TRDV1	9.72E-04	0	5425
NAGPA	9.70E-04	0	5426
SLN	9.69E-04	0	5427
SRD5A3	9.69E-04	0	5428
LY6E	9.68E-04	0	5429
ND6	9.68E-04	0	5430
GNAI1	9.68E-04	0	5431
DGKB	9.67E-04	0	5432
CDC42EP3	9.66E-04	0	5433
DMRTA1	9.66E-04	0	5434
BBS1	9.64E-04	0	5435
FBXO40	9.63E-04	0	5436
USP13	9.63E-04	0	5437
SUPT3H	9.63E-04	0	5438
CYP1B1	9.62E-04	0	5439
DOHH	9.62E-04	0	5440
ZBTB32	9.61E-04	0	5441
TLE3	9.59E-04	0	5442
MSMO1	9.58E-04	0	5443
IGHV3-48	9.58E-04	0	5444
VBP1	9.57E-04	0	5445
QPRT	9.55E-04	0	5446
EIF3H	9.53E-04	0	5447
USP5	9.53E-04	0	5448
CASR	9.51E-04	0	5449
RFNG	9.51E-04	0	5450
YWHAG	9.51E-04	0	5451
ARL4D	9.50E-04	0	5452
MIR92B	9.50E-04	0	5453
KIF16B	9.48E-04	0	5454
SLC44A2	9.47E-04	0	5455
SLC22A12	9.43E-04	0	5456

MUPP	9.42E-04	0	5457
MRPL42	9.42E-04	0	5458
ADRA1D	9.41E-04	0	5459
C3ORF37	9.40E-04	0	5460
NR1D1	9.40E-04	0	5461
PSMB1	9.39E-04	0	5462
KRT126P	9.39E-04	0	5463
COG6	9.39E-04	0	5464
AGER	9.38E-04	0	5465
UBL5	9.37E-04	0	5466
UBC	9.37E-04	0	5467
NEURL2	9.36E-04	0	5468
MIR1258	9.33E-04	0	5469
PRUNE2	9.28E-04	0	5470
CLDN23	9.27E-04	0	5471
MIR548P	9.27E-04	0	5472
PLA2G12B	9.27E-04	0	5473
NRARP	9.27E-04	0	5474
SIGLEC6	9.25E-04	0	5475
ZNF74	9.24E-04	0	5476
EIF5A	9.24E-04	0	5477
RN7SK	9.23E-04	0	5478
MIR760	9.23E-04	0	5479
MIR433	9.22E-04	0	5480
BNIPL	9.22E-04	0	5481
RNF213	9.21E-04	0	5482
DRC1	9.20E-04	0	5483
MAGEA10	9.20E-04	0	5484
MIR598	9.20E-04	0	5485
TREML4	9.20E-04	0	5486
MIR147B	9.19E-04	0	5487
POMP	9.18E-04	0	5488
ADH5	9.18E-04	0	5489
EIF5	9.17E-04	0	5490
SEPSECS	9.14E-04	0	5491
TPM1	9.14E-04	0	5492
UGT2B15	9.12E-04	0	5493
AKR1C2	9.11E-04	0	5494
ERGIC1	9.10E-04	0	5495
FCHO1	9.10E-04	0	5496
HIST2H3D	9.10E-04	0	5497
ITGA10	9.10E-04	0	5498
LEMD3	9.09E-04	0	5499

MIR595	9.08E-04	0	5500
FBXL18	9.07E-04	0	5501
COX5B	9.07E-04	0	5502
MIR542	9.06E-04	0	5503
NISCH	9.05E-04	0	5504
FZD8	9.04E-04	0	5505
SEMA5A	9.03E-04	0	5506
XPOT	9.03E-04	0	5507
TOC	9.03E-04	0	5508
ATP6V0D2	9.02E-04	0	5509
SNORD34	9.00E-04	0	5510
CCNK	9.00E-04	0	5511
CEP290	8.96E-04	0	5512
WDR82	8.96E-04	0	5513
NUP188	8.95E-04	0	5514
COL6A1	8.93E-04	0	5515
MIR641	8.92E-04	0	5516
EIF2AK4	8.91E-04	0	5517
DNAJC22	8.91E-04	0	5518
PODXL2	8.91E-04	0	5519
DEFB105A	8.91E-04	0	5520
MAK	8.91E-04	0	5521
NRCAM	8.88E-04	0	5522
NRBP1	8.88E-04	0	5523
COX4I2	8.88E-04	0	5524
PPP6R1	8.88E-04	0	5525
ZNF461	8.87E-04	0	5526
PSORS4	8.86E-04	0	5527
CAMLG	8.86E-04	0	5528
FCN2	8.84E-04	0	5529
KIAA1967	8.84E-04	0	5530
CUX2	8.84E-04	0	5531
COCH	8.83E-04	0	5532
ENDO1	8.82E-04	0	5533
GPC2	8.81E-04	0	5534
MUC17	8.81E-04	0	5535
CAAP1	8.79E-04	0	5536
GRAMD4	8.79E-04	0	5537
SNORD105	8.79E-04	0	5538
WRAP53	8.79E-04	0	5539
COPS6	8.79E-04	0	5540
CYP3AP1	8.77E-04	0	5541
DGKG	8.76E-04	0	5542

GHITM	8.75E-04	0	5543
RAB38	8.75E-04	0	5544
RBMS2	8.74E-04	0	5545
GNG4	8.74E-04	0	5546
MEAF6	8.73E-04	0	5547
SNUPN	8.71E-04	0	5548
AVEN	8.71E-04	0	5549
TRMU	8.71E-04	0	5550
CENPV	8.70E-04	0	5551
PSORS1C1	8.70E-04	0	5552
PSMD2	8.69E-04	0	5553
KHK	8.69E-04	0	5554
ELP3	8.69E-04	0	5555
SLC15A1	8.68E-04	0	5556
TMPRSS11E	8.67E-04	0	5557
WDR18	8.66E-04	0	5558
NFATC4	8.66E-04	0	5559
ATOH1	8.65E-04	0	5560
CCNY	8.65E-04	0	5561
OSBP2	8.65E-04	0	5562
ARL15	8.64E-04	0	5563
AKR1A1	8.63E-04	0	5564
PTPLA	8.62E-04	0	5565
RCCD1	8.62E-04	0	5566
KLHL17	8.61E-04	0	5567
SNRNP40	8.61E-04	0	5568
SNORD68	8.60E-04	0	5569
ATOH7	8.58E-04	0	5570
SCARB2	8.58E-04	0	5571
SCN1A	8.57E-04	0	5572
TRR	8.56E-04	0	5573
DNAJB4	8.56E-04	0	5574
ATG9B	8.55E-04	0	5575
HARS2	8.54E-04	0	5576
CAP2	8.54E-04	0	5577
CHMP3	8.52E-04	0	5578
ATE1	8.50E-04	0	5579
ABHD16A	8.49E-04	0	5580
CADPS2	8.49E-04	0	5581
EMC2	8.48E-04	0	5582
CHD3	8.47E-04	0	5583
MIR137	8.46E-04	0	5584
FCF1	8.46E-04	0	5585

AFA	8.46E-04	0	5586
PLCD4	8.46E-04	0	5587
RPL18	8.45E-04	0	5588
CTNNAL1	8.44E-04	0	5589
MIR4295	8.44E-04	0	5590
EMB	8.44E-04	0	5591
ACOX1	8.41E-04	0	5592
MED16	8.41E-04	0	5593
GPR1	8.41E-04	0	5594
RNPS1	8.39E-04	0	5595
ARL5B	8.39E-04	0	5596
MIR324	8.38E-04	0	5597
KIF1A	8.37E-04	0	5598
C4ORF6	8.36E-04	0	5599
ZNF444	8.35E-04	0	5600
SATB2	8.35E-04	0	5601
ALG8	8.35E-04	0	5602
KIF7	8.35E-04	0	5603
COL6A2	8.30E-04	0	5604
NEURL	8.30E-04	0	5605
FCRL5	8.29E-04	0	5606
D2HGDH	8.29E-04	0	5607
BMS1	8.28E-04	0	5608
LMCD1	8.28E-04	0	5609
KIAA0020	8.27E-04	0	5610
NRGN	8.27E-04	0	5611
KANK2	8.25E-04	0	5612
SOAT2	8.22E-04	0	5613
BLOC1S3	8.22E-04	0	5614
ARID1B	8.22E-04	0	5615
IGF2BP3	8.21E-04	0	5616
SSFA2	8.21E-04	0	5617
TESPA1	8.21E-04	0	5618
HMGCS1	8.19E-04	0	5619
AP1AR	8.18E-04	0	5620
LPPR4	8.17E-04	0	5621
PRICKLE1	8.17E-04	0	5622
DEFB128	8.17E-04	0	5623
NIM1	8.17E-04	0	5624
MAFK	8.16E-04	0	5625
VEGFB	8.15E-04	0	5626
MIR455	8.15E-04	0	5627
S100A10	8.14E-04	0	5628

HYPLIP2	8.12E-04	0	5629
SV2A	8.11E-04	0	5630
PSMD8	8.11E-04	0	5631
MIR3189	8.10E-04	0	5632
TNFSF12	8.09E-04	0	5633
PSD4	8.07E-04	0	5634
PKI55	8.07E-04	0	5635
ACP5	8.04E-04	0	5636
KIAA0825	8.03E-04	0	5637
LHX9	8.02E-04	0	5638
NEK8	8.02E-04	0	5639
UBR2	8.01E-04	0	5640
RIOK2	8.01E-04	0	5641
PDCL	8.01E-04	0	5642
TMBIM6	8.00E-04	0	5643
NME2P1	8.00E-04	0	5644
ZG16	8.00E-04	0	5645
HOXD13	7.99E-04	0	5646
NDUFB11	7.98E-04	0	5647
ZC2HC1A	7.98E-04	0	5648
NKX2-3	7.98E-04	0	5649
ARID3B	7.98E-04	0	5650
RINT1	7.97E-04	0	5651
ERV3-1	7.96E-04	0	5652
ERVFRD-1	7.96E-04	0	5653
RRP9	7.95E-04	0	5654
MYH4	7.95E-04	0	5655
GYG1	7.94E-04	0	5656
DYNC2H1	7.94E-04	0	5657
PSG4	7.93E-04	0	5658
LRAT	7.93E-04	0	5659
DPY30	7.92E-04	0	5660
MIPEP	7.90E-04	0	5661
SF3A2	7.89E-04	0	5662
ZP3	7.88E-04	0	5663
GPSM3	7.88E-04	0	5664
DTX2	7.87E-04	0	5665
RSL24D1	7.87E-04	0	5666
MAP9	7.86E-04	0	5667
SETD1A	7.84E-04	0	5668
USP17L2	7.83E-04	0	5669
EVX1	7.82E-04	0	5670
MIR718	7.80E-04	0	5671

GATAD2A	7.79E-04	0	5672
MIR548AN	7.79E-04	0	5673
PRMT6	7.79E-04	0	5674
PEX2	7.79E-04	0	5675
FUK	7.77E-04	0	5676
PXDNL	7.76E-04	0	5677
RABEPK	7.76E-04	0	5678
LY6K	7.73E-04	0	5679
APITD1	7.73E-04	0	5680
NPLOC4	7.72E-04	0	5681
MIR19B2	7.72E-04	0	5682
PDA1	7.72E-04	0	5683
ZNF410	7.71E-04	0	5684
TRNAS2	7.69E-04	0	5685
PRKAG3	7.69E-04	0	5686
FRA5C	7.68E-04	0	5687
SLC2A10	7.68E-04	0	5688
MAGOH	7.68E-04	0	5689
FNDC1	7.67E-04	0	5690
LMLN	7.67E-04	0	5691
VTA1	7.66E-04	0	5692
NBEA	7.65E-04	0	5693
WDR13	7.64E-04	0	5694
RAI1	7.64E-04	0	5695
FZD10	7.63E-04	0	5696
CBR3	7.63E-04	0	5697
SCGB1D4	7.63E-04	0	5698
TMEM100	7.61E-04	0	5699
C2ORF68	7.60E-04	0	5700
PRSS55	7.60E-04	0	5701
ABCF2	7.60E-04	0	5702
BBS4	7.59E-04	0	5703
AKAP3	7.57E-04	0	5704
ATG10	7.55E-04	0	5705
GDPD1	7.51E-04	0	5706
VPS36	7.50E-04	0	5707
KLRF1	7.48E-04	0	5708
AGFG2	7.47E-04	0	5709
IPO5	7.47E-04	0	5710
AQR	7.47E-04	0	5711
GLRX3	7.47E-04	0	5712
SCN4A	7.47E-04	0	5713
YBX3	7.46E-04	0	5714

CYTH3	7.46E-04	0	5715
HSPA4L	7.46E-04	0	5716
TRAM2	7.46E-04	0	5717
TSSC1	7.46E-04	0	5718
AOAH	7.44E-04	0	5719
DIDO1	7.43E-04	0	5720
MRVI1	7.43E-04	0	5721
FUT3	7.43E-04	0	5722
SRXN1	7.42E-04	0	5723
LINC00520	7.41E-04	0	5724
METRN	7.41E-04	0	5725
PWP1	7.41E-04	0	5726
SH3BGR	7.41E-04	0	5727
PARD6B	7.41E-04	0	5728
ASLP1	7.40E-04	0	5729
DYRK3	7.40E-04	0	5730
PDIA5	7.40E-04	0	5731
MMP12	7.39E-04	0	5732
PPT2	7.39E-04	0	5733
ANGPTL7	7.37E-04	0	5734
RAB24	7.35E-04	0	5735
MT1XP1	7.35E-04	0	5736
UBE2J1	7.35E-04	0	5737
OPHN1	7.35E-04	0	5738
MTFR1	7.33E-04	0	5739
GTF2F2	7.32E-04	0	5740
ESYT2	7.31E-04	0	5741
SPEM1	7.31E-04	0	5742
SYTL5	7.29E-04	0	5743
LEPROTL1	7.29E-04	0	5744
MREG	7.28E-04	0	5745
MIR885	7.28E-04	0	5746
BTG3	7.28E-04	0	5747
CENPC1	7.27E-04	0	5748
LRRC25	7.27E-04	0	5749
CHRDL2	7.26E-04	0	5750
PARP3	7.25E-04	0	5751
ABCE1	7.23E-04	0	5752
EIF1	7.23E-04	0	5753
PRRC2A	7.22E-04	0	5754
FZD6	7.22E-04	0	5755
RAP1GAP2	7.21E-04	0	5756
KCNH3	7.21E-04	0	5757

IHG1	7.20E-04	0	5758
GSTO1	7.20E-04	0	5759
MIR769	7.20E-04	0	5760
PSMC4	7.20E-04	0	5761
GABARAPL1	7.20E-04	0	5762
CLASP1	7.19E-04	0	5763
SUPT20H	7.18E-04	0	5764
POLM	7.18E-04	0	5765
RASGRP4	7.18E-04	0	5766
ANKRA2	7.18E-04	0	5767
SOD3	7.18E-04	0	5768
KLK10	7.18E-04	0	5769
FBXL7	7.18E-04	0	5770
DNTTIP1	7.18E-04	0	5771
SH3BGRL3	7.16E-04	0	5772
PPAP2A	7.16E-04	0	5773
HOXD1	7.16E-04	0	5774
RAD54L2	7.15E-04	0	5775
RAB40AL	7.14E-04	0	5776
NEUROG1	7.13E-04	0	5777
GORASP2	7.12E-04	0	5778
ACTR1A	7.12E-04	0	5779
RPL22L1	7.11E-04	0	5780
MIR622	7.11E-04	0	5781
HAL	7.09E-04	0	5782
CHMP2A	7.09E-04	0	5783
DGKI	7.08E-04	0	5784
TIMM22	7.08E-04	0	5785
FCRL4	7.07E-04	0	5786
PPP1R17	7.06E-04	0	5787
FKBP15	7.05E-04	0	5788
MIR381	7.04E-04	0	5789
EPGN	7.04E-04	0	5790
HMHA1	7.04E-04	0	5791
DERL3	7.00E-04	0	5792
WBP1	7.00E-04	0	5793
TRNAC1	6.99E-04	0	5794
TP53BP2	6.97E-04	0	5795
CATSPERB	6.97E-04	0	5796
CYP26C1	6.96E-04	0	5797
DNM3	6.95E-04	0	5798
DNAJC6	6.94E-04	0	5799
VPS52	6.94E-04	0	5800

EHD4	6.93E-04	0	5801
TEX19	6.91E-04	0	5802
MAB21L1	6.91E-04	0	5803
SHPK	6.91E-04	0	5804
DYNLL2	6.91E-04	0	5805
DYNC1LI1	6.91E-04	0	5806
HVCN1	6.91E-04	0	5807
GFER	6.90E-04	0	5808
LHX5	6.90E-04	0	5809
NELF	6.89E-04	0	5810
AIS1	6.88E-04	0	5811
IDDM8	6.88E-04	0	5812
IFT43	6.86E-04	0	5813
COL1A1	6.86E-04	0	5814
VPS13B	6.86E-04	0	5815
VPS37B	6.85E-04	0	5816
RNR3	6.85E-04	0	5817
DNAH6	6.84E-04	0	5818
DDRGK1	6.83E-04	0	5819
RNY1	6.83E-04	0	5820
OPA3	6.82E-04	0	5821
SLC4A5	6.81E-04	0	5822
RNU2-1	6.81E-04	0	5823
SLC35A1	6.81E-04	0	5824
PEAK1	6.81E-04	0	5825
SEMA3D	6.81E-04	0	5826
TMEM205	6.81E-04	0	5827
TMEM230	6.81E-04	0	5828
DNASE2B	6.80E-04	0	5829
ARSE	6.79E-04	0	5830
ORAI2	6.79E-04	0	5831
MYH7B	6.79E-04	0	5832
SWSAP1	6.78E-04	0	5833
MIR448	6.77E-04	0	5834
NIF3L1	6.77E-04	0	5835
VCPIP1	6.77E-04	0	5836
MTMR12	6.76E-04	0	5837
PNMA2	6.76E-04	0	5838
MGAT3	6.76E-04	0	5839
IGKV3-25	6.74E-04	0	5840
MED6	6.74E-04	0	5841
PARG	6.74E-04	0	5842
OXSM	6.73E-04	0	5843

PPP2R2A	6.73E-04	0	5844
IFT20	6.73E-04	0	5845
ALX4	6.72E-04	0	5846
PLCL1	6.70E-04	0	5847
CSRP2BP	6.70E-04	0	5848
OFC3	6.69E-04	0	5849
PITPNA	6.68E-04	0	5850
ZNF654	6.68E-04	0	5851
WDR62	6.67E-04	0	5852
KCP	6.67E-04	0	5853
TIAF1	6.66E-04	0	5854
TRGV2	6.65E-04	0	5855
OXT	6.64E-04	0	5856
AKIP1	6.64E-04	0	5857
UGGT1	6.63E-04	0	5858
SLC15A3	6.62E-04	0	5859
MTRF1	6.62E-04	0	5860
IGLJ7	6.60E-04	0	5861
ARFIP2	6.60E-04	0	5862
GSTM4	6.60E-04	0	5863
SP2	6.59E-04	0	5864
CSTA	6.58E-04	0	5865
TNPO3	6.56E-04	0	5866
LEO1	6.54E-04	0	5867
GFRA4	6.54E-04	0	5868
MGR	6.54E-04	0	5869
GRK1	6.54E-04	0	5870
TADA3	6.53E-04	0	5871
CHCHD3	6.53E-04	0	5872
AAT2	6.53E-04	0	5873
FTCD	6.53E-04	0	5874
ESRP1	6.52E-04	0	5875
PLIN1	6.51E-04	0	5876
HNP1	6.50E-04	0	5877
SDHAF1	6.50E-04	0	5878
RTF1	6.49E-04	0	5879
ASCL1	6.48E-04	0	5880
ATXN7L3	6.48E-04	0	5881
HBG1	6.47E-04	0	5882
PTGR1	6.47E-04	0	5883
DKK4	6.46E-04	0	5884
OFD1	6.46E-04	0	5885
VILL	6.46E-04	0	5886

MIA2	6.46E-04	0	5887
SPATA7	6.46E-04	0	5888
POLR2E	6.46E-04	0	5889
MRGBP	6.46E-04	0	5890
IL18BP	6.45E-04	0	5891
RASAL1	6.44E-04	0	5892
BIRC7	6.42E-04	0	5893
KLF9	6.41E-04	0	5894
USP6NL	6.41E-04	0	5895
LPPR1	6.41E-04	0	5896
NMD3	6.41E-04	0	5897
IGKV2D-18	6.39E-04	1	5898
IGKV1-35	6.39E-04	0	5899
MTMR6	6.38E-04	0	5900
GUCY2F	6.38E-04	0	5901
DSCAM-AS1	6.36E-04	0	5902
SNX9	6.35E-04	0	5903
MTCP1	6.35E-04	0	5904
NOBOX	6.35E-04	0	5905
RHPN1	6.35E-04	0	5906
TAAR8	6.34E-04	0	5907
CYB5B	6.33E-04	0	5908
TSPAN18	6.32E-04	0	5909
TSHZ3	6.32E-04	0	5910
NOMO1	6.32E-04	0	5911
ABCC3	6.30E-04	0	5912
SYNRG	6.29E-04	0	5913
SLC26A5	6.27E-04	0	5914
FDPS	6.27E-04	0	5915
TNXA	6.26E-04	0	5916
OSCP1	6.26E-04	0	5917
IGKV2-26	6.25E-04	0	5918
H2AFB2	6.23E-04	0	5919
KIF11	6.22E-04	0	5920
ALOXE3	6.22E-04	0	5921
UBXN2B	6.21E-04	0	5922
ERV9-1	6.21E-04	0	5923
CEBPG	6.20E-04	0	5924
YIPF1	6.20E-04	0	5925
SOHLH1	6.19E-04	0	5926
GSM1	6.19E-04	0	5927
ALAS2	6.18E-04	0	5928
AP1G2	6.17E-04	0	5929

SP140	6.17E-04	0	5930
SURF4	6.16E-04	0	5931
NADSYN1	6.15E-04	0	5932
TUBA1B	6.13E-04	0	5933
SLC2A11	6.12E-04	0	5934
NEURL4	6.12E-04	0	5935
TRIM47	6.12E-04	0	5936
ZNF238	6.11E-04	0	5937
SLC50A1	6.11E-04	0	5938
HMGN2P46	6.11E-04	0	5939
SNX13	6.11E-04	0	5940
DUSP13	6.10E-04	0	5941
PDCD5	6.10E-04	0	5942
LEPREL4	6.10E-04	0	5943
CASD1	6.09E-04	0	5944
CCDC101	6.07E-04	0	5945
SERBP1	6.07E-04	0	5946
TSPAN4	6.07E-04	0	5947
DSCAML1	6.07E-04	0	5948
GNPDA1	6.06E-04	0	5949
NDUFA3	6.05E-04	0	5950
RPL18A	6.05E-04	0	5951
GOLPH3L	6.04E-04	0	5952
NRAP	6.02E-04	0	5953
OPN1MW	6.00E-04	0	5954
PIN4	5.99E-04	0	5955
SOX15	5.99E-04	0	5956
PDCD7	5.99E-04	0	5957
OA15	5.96E-04	0	5958
AKR7A3	5.95E-04	0	5959
SQRDL	5.95E-04	0	5960
RPS10	5.95E-04	0	5961
TAAR2	5.95E-04	0	5962
MAGOH2	5.94E-04	0	5963
MIR519C	5.94E-04	0	5964
SARS2	5.93E-04	0	5965
PSORS1C2	5.93E-04	0	5966
LEAP2	5.92E-04	0	5967
ZZZ3	5.92E-04	0	5968
C5ORF25	5.91E-04	0	5969
ARHGAP6	5.91E-04	0	5970
GLIPR1	5.88E-04	0	5971
NCEH1	5.88E-04	0	5972

UFM1	5.88E-04	0	5973
ADH6	5.87E-04	0	5974
HNRNPM	5.86E-04	0	5975
HTATSF1	5.86E-04	0	5976
WHSC2	5.86E-04	0	5977
FBL	5.86E-04	0	5978
IGKV2-30	5.85E-04	0	5979
CYP4V2	5.85E-04	0	5980
ZRANB1	5.84E-04	0	5981
SARAF	5.84E-04	0	5982
PCLO	5.84E-04	0	5983
CBX1	5.82E-04	0	5984
ZC3H3	5.81E-04	0	5985
AP1M2	5.81E-04	0	5986
GOA1	5.81E-04	0	5987
SNORD42A	5.81E-04	0	5988
NRL	5.79E-04	0	5989
CILP2	5.78E-04	0	5990
SNORD31	5.78E-04	0	5991
SH3YL1	5.78E-04	0	5992
SYVN1	5.78E-04	0	5993
SCML2	5.78E-04	0	5994
FAM161A	5.77E-04	0	5995
PGM2	5.75E-04	0	5996
RNF26	5.75E-04	0	5997
MT-CO1	5.73E-04	0	5998
KIR2DS4	5.73E-04	0	5999
DDHD1	5.73E-04	0	6000
TMEM189	5.72E-04	0	6001
UBE2NL	5.72E-04	0	6002
CELSR3	5.72E-04	0	6003
FHAD1	5.72E-04	0	6004
CASP12	5.72E-04	0	6005
TXNIP	5.71E-04	0	6006
PRIMPOL	5.71E-04	0	6007
FADS3	5.71E-04	0	6008
SYBU	5.71E-04	0	6009
AOX1	5.70E-04	0	6010
RNF157	5.70E-04	0	6011
NEK3	5.69E-04	0	6012
LSMD1	5.69E-04	0	6013
EEFSEC	5.63E-04	0	6014
ATG13	5.62E-04	0	6015

UGT2B10	5.62E-04	0	6016
ARHGAP25	5.62E-04	0	6017
GNE	5.62E-04	0	6018
TSPAN2	5.61E-04	0	6019
CREBRF	5.61E-04	0	6020
FREM1	5.61E-04	0	6021
SFTPA1	5.60E-04	0	6022
RPN2	5.60E-04	0	6023
AFF2	5.59E-04	0	6024
PIN1P1	5.59E-04	0	6025
ZFX	5.58E-04	0	6026
FOXN4	5.58E-04	0	6027
SFMBT1	5.57E-04	0	6028
BTAF1	5.57E-04	0	6029
TENM4	5.57E-04	0	6030
MIR574	5.56E-04	0	6031
SCGB2A2	5.55E-04	0	6032
ULBP3	5.54E-04	0	6033
HIF1A-AS2	5.54E-04	0	6034
MT1L	5.54E-04	0	6035
6-Mar	5.53E-04	0	6036
TIMM44	5.53E-04	0	6037
CHD8	5.53E-04	0	6038
RGR	5.53E-04	0	6039
PGS1	5.51E-04	0	6040
SNORD3A	5.49E-04	0	6041
TAS1R2	5.48E-04	0	6042
CACNA1I	5.47E-04	0	6043
SRRM1	5.47E-04	0	6044
DFNA53	5.46E-04	0	6045
GABARAP	5.46E-04	0	6046
RARS2	5.46E-04	0	6047
PDGFC	5.46E-04	0	6048
ARPC5L	5.45E-04	0	6049
USP46	5.45E-04	0	6050
PROSP	5.44E-04	0	6051
MSRB3	5.44E-04	0	6052
FEZF1	5.44E-04	0	6053
GKAP1	5.43E-04	0	6054
TRIM39-RPP:	5.43E-04	0	6055
NDUFAF3	5.43E-04	0	6056
TAF12	5.40E-04	0	6057
MIR496	5.40E-04	0	6058

ALG3	5.39E-04	0	6059
INPP4A	5.39E-04	0	6060
CACUL1	5.38E-04	0	6061
DNAJB2	5.38E-04	0	6062
DNASE1L2	5.37E-04	0	6063
FAM175A	5.36E-04	0	6064
SLC8A3	5.36E-04	0	6065
ARPC1A	5.35E-04	0	6066
WDR11	5.35E-04	0	6067
SHPRH	5.34E-04	0	6068
DOCK6	5.34E-04	0	6069
ZNF366	5.34E-04	0	6070
MIR330	5.32E-04	0	6071
PERM1	5.32E-04	0	6072
COQ2	5.32E-04	0	6073
MIR492	5.31E-04	0	6074
SPAM1	5.31E-04	0	6075
RCSD1	5.30E-04	0	6076
FBXL5	5.29E-04	0	6077
RAB31	5.29E-04	0	6078
EXTL3	5.29E-04	0	6079
STL	5.28E-04	0	6080
HEBP2	5.27E-04	0	6081
RNF185	5.27E-04	0	6082
RNF186	5.27E-04	0	6083
PCDHGA3	5.26E-04	0	6084
TMX2	5.25E-04	0	6085
UBN1	5.25E-04	0	6086
MRPS26	5.23E-04	0	6087
PPP2R3C	5.23E-04	0	6088
PAOX	5.23E-04	0	6089
ENTPD8	5.22E-04	0	6090
SNAP29	5.22E-04	0	6091
UNC45B	5.20E-04	0	6092
LILRA4	5.20E-04	0	6093
ANKFY1	5.20E-04	0	6094
MOCS2	5.19E-04	0	6095
KIF3A	5.19E-04	0	6096
DDB1	5.17E-04	0	6097
BLMH	5.17E-04	0	6098
ZNF622	5.16E-04	0	6099
NAT10	5.16E-04	0	6100
MPP1	5.16E-04	0	6101

GRM8	5.15E-04	0	6102
ANO6	5.13E-04	0	6103
CHRM5	5.11E-04	0	6104
AGMO	5.10E-04	0	6105
DTWD1	5.09E-04	0	6106
MAATS1	5.07E-04	0	6107
ND3	5.06E-04	0	6108
NACC2	5.06E-04	0	6109
ZBTB14	5.06E-04	0	6110
WNT16	5.04E-04	0	6111
EGI	5.03E-04	0	6112
CISD1	5.03E-04	0	6113
ACKR1	5.03E-04	0	6114
IER3IP1	5.03E-04	0	6115
SEMA6A	5.03E-04	0	6116
PAAF1	5.02E-04	0	6117
EEF1B2	5.02E-04	0	6118
SNORD15B	5.02E-04	0	6119
PIP5K1B	5.01E-04	0	6120
KDM5D	5.00E-04	0	6121
NEU4	5.00E-04	0	6122
SNCG	4.99E-04	0	6123
PALM	4.98E-04	0	6124
DENND1C	4.98E-04	0	6125
TMEM88	4.98E-04	0	6126
RPS12	4.96E-04	0	6127
AP3D1	4.96E-04	0	6128
MIR300	4.95E-04	0	6129
SLC26A2	4.94E-04	0	6130
MCOLN3	4.94E-04	0	6131
KLHL8	4.93E-04	0	6132
MAPRE2	4.92E-04	0	6133
STK10	4.92E-04	0	6134
ZP4	4.92E-04	0	6135
TIMM17A	4.91E-04	0	6136
MIR3960	4.90E-04	0	6137
DDX23	4.89E-04	0	6138
ZSWIM2	4.89E-04	0	6139
TPSG1	4.87E-04	0	6140
PMM1	4.87E-04	0	6141
ZBED4	4.87E-04	0	6142
RABGAP1	4.86E-04	0	6143
TAS2R15P	4.86E-04	0	6144

TRMT112	4.85E-04	0	6145
KDM5C	4.84E-04	0	6146
NEK5	4.84E-04	0	6147
MYOZ3	4.84E-04	0	6148
PGLYRP2	4.83E-04	0	6149
GNL2	4.83E-04	0	6150
WNT8A	4.82E-04	0	6151
ITGB1BP2	4.82E-04	0	6152
AGBL1	4.82E-04	0	6153
PROB1	4.81E-04	0	6154
SKP1P2	4.81E-04	0	6155
AP4M1	4.81E-04	0	6156
MPV17	4.81E-04	0	6157
IGKV1-39	4.80E-04	0	6158
ATF7	4.79E-04	0	6159
GIMAP7	4.78E-04	0	6160
FOXH1	4.78E-04	0	6161
UCMA	4.77E-04	0	6162
OVCH2	4.76E-04	0	6163
CD300LB	4.76E-04	0	6164
EHBP1L1	4.74E-04	0	6165
C16ORF70	4.74E-04	0	6166
CHRNE	4.73E-04	0	6167
DNAJA4	4.73E-04	0	6168
HSPA13	4.73E-04	0	6169
HLTF	4.73E-04	0	6170
DPM2	4.72E-04	0	6171
PTDSS1	4.72E-04	0	6172
ASPA	4.71E-04	0	6173
MOV10L1	4.71E-04	0	6174
KCNG4	4.71E-04	0	6175
ARHGEF10	4.70E-04	0	6176
MBOAT7	4.69E-04	0	6177
PRCD	4.69E-04	0	6178
TREML2	4.68E-04	0	6179
AZGP1	4.68E-04	0	6180
SOX8	4.68E-04	0	6181
RPS24	4.67E-04	0	6182
DAK	4.67E-04	0	6183
RNASE4	4.66E-04	0	6184
PPP2R2B	4.66E-04	0	6185
MMP10	4.66E-04	0	6186
KIR2DL5A	4.66E-04	0	6187

NDUFB2	4.66E-04	0	6188
PAQR3	4.65E-04	0	6189
ZAK	4.65E-04	0	6190
FIS1	4.65E-04	0	6191
MPHOSPH8	4.64E-04	0	6192
PADI2	4.64E-04	0	6193
GBA2	4.64E-04	0	6194
AP3S2	4.64E-04	0	6195
SEC24A	4.63E-04	0	6196
B3GAT2	4.63E-04	0	6197
FBXO6	4.63E-04	0	6198
HOXC8	4.61E-04	0	6199
FMO5	4.61E-04	0	6200
NOA1	4.61E-04	0	6201
IMPA1	4.60E-04	0	6202
RP1	4.58E-04	0	6203
DGCR6L	4.58E-04	0	6204
PARK10	4.58E-04	0	6205
RASD1	4.58E-04	0	6206
OS9	4.57E-04	0	6207
SOX1	4.57E-04	0	6208
CSGALNACT1	4.56E-04	0	6209
VGLL1	4.55E-04	0	6210
BW61	4.54E-04	0	6211
HLA-DRB6	4.54E-04	0	6212
ZCCHC11	4.53E-04	0	6213
MIRLET7A1	4.52E-04	0	6214
HTN1	4.51E-04	0	6215
TGM4	4.51E-04	0	6216
C8B	4.51E-04	0	6217
SUV420H1	4.51E-04	0	6218
TRMT61A	4.50E-04	0	6219
THOC2	4.50E-04	0	6220
CRACR2A	4.50E-04	0	6221
PHLDB3	4.47E-04	0	6222
DUSP28	4.46E-04	0	6223
ZNF675	4.45E-04	0	6224
AMT	4.45E-04	0	6225
SNORD22	4.45E-04	0	6226
MED20	4.45E-04	0	6227
DDX52	4.44E-04	0	6228
ACVR2B	4.42E-04	0	6229
ARSJ	4.40E-04	0	6230

DNAJB5	4.40E-04	0	6231
TXNDC2	4.40E-04	0	6232
CD101	4.40E-04	0	6233
TRIP13	4.39E-04	0	6234
RBM8A	4.39E-04	0	6235
DEFA5	4.38E-04	0	6236
C1D	4.38E-04	0	6237
HTR5A	4.38E-04	0	6238
MAT2B	4.37E-04	0	6239
RELT	4.37E-04	0	6240
HECW1	4.37E-04	0	6241
DOC2B	4.36E-04	0	6242
HDHD1	4.36E-04	0	6243
MRPL49	4.36E-04	0	6244
SETD1B	4.35E-04	0	6245
TMTC1	4.34E-04	0	6246
GSTM2	4.33E-04	0	6247
HTA	4.32E-04	0	6248
KCNJ12	4.32E-04	0	6249
MIR30A	4.32E-04	0	6250
ADAMDEC1	4.32E-04	0	6251
B4GALNT4	4.32E-04	0	6252
FERP1	4.32E-04	0	6253
PRPF4B	4.32E-04	0	6254
SCN9A	4.29E-04	0	6255
MT1DP	4.29E-04	0	6256
COL1AR	4.26E-04	0	6257
MZB1	4.26E-04	0	6258
OOEP	4.25E-04	0	6259
SLC25A27	4.24E-04	0	6260
BPTF	4.23E-04	0	6261
HIPK4	4.22E-04	0	6262
LIN7B	4.21E-04	0	6263
CLPX	4.20E-04	0	6264
HUG1	4.18E-04	0	6265
OPALIN	4.17E-04	0	6266
MAS1L	4.17E-04	0	6267
ATP6V1B1	4.16E-04	0	6268
ENY2	4.15E-04	0	6269
IRX6	4.15E-04	0	6270
CHD1L	4.15E-04	0	6271
MT-ND6	4.14E-04	0	6272
MED21	4.14E-04	0	6273

KDELR2	4.13E-04	0	6274
ZIC1	4.13E-04	0	6275
CHMP2B	4.11E-04	0	6276
GCY	4.10E-04	0	6277
WDR61	4.10E-04	0	6278
SERP2	4.10E-04	0	6279
H2BFWT	4.09E-04	0	6280
PPIE	4.09E-04	0	6281
LPCAT1	4.09E-04	0	6282
MFAP4	4.08E-04	0	6283
POLR2K	4.08E-04	0	6284
MIR507	4.08E-04	0	6285
ACLS	4.04E-04	0	6286
CEP72	4.04E-04	0	6287
MUC22	4.04E-04	0	6288
MTUS2	4.03E-04	0	6289
IGSF1	4.03E-04	0	6290
GSDMA	4.02E-04	0	6291
DUP17Q12	4.00E-04	0	6292
CNNM3	4.00E-04	0	6293
NEU1	4.00E-04	0	6294
PCBP3	4.00E-04	0	6295
POLR2M	4.00E-04	0	6296
PI4KA	3.99E-04	0	6297
ANP32E	3.99E-04	0	6298
SEC14L3	3.98E-04	0	6299
CALR3	3.97E-04	0	6300
SRRT	3.96E-04	0	6301
POFUT1	3.96E-04	0	6302
TRAPPC2	3.95E-04	0	6303
MEIS2	3.95E-04	0	6304
TAF6	3.95E-04	0	6305
TNNI3K	3.95E-04	0	6306
C9ORF156	3.94E-04	0	6307
BP28	3.93E-04	0	6308
MTRNR2L11	3.93E-04	0	6309
FAM83B	3.93E-04	0	6310
TBXA2R	3.92E-04	0	6311
ATP10B	3.92E-04	0	6312
PUS3	3.91E-04	0	6313
SLCO4C1	3.91E-04	0	6314
CLEC1A	3.91E-04	0	6315
PNPLA8	3.91E-04	0	6316

MYEOV2	3.89E-04	0	6317
NOC3L	3.87E-04	0	6318
FAM175B	3.86E-04	0	6319
KRT34	3.85E-04	0	6320
DUPD1	3.85E-04	0	6321
GET4	3.84E-04	0	6322
GAGE6	3.84E-04	0	6323
HEBP1	3.83E-04	0	6324
NIDDM1	3.81E-04	0	6325
GPR182	3.81E-04	0	6326
FBXO2	3.81E-04	0	6327
BCC1	3.81E-04	0	6328
FGD3	3.80E-04	0	6329
PARP10	3.80E-04	0	6330
CNTFR	3.79E-04	0	6331
PDZRN4	3.79E-04	0	6332
EMX2	3.79E-04	0	6333
APOBR	3.79E-04	0	6334
MIR921	3.79E-04	0	6335
CABP2	3.78E-04	0	6336
LMO3	3.78E-04	0	6337
CHORDC1	3.78E-04	0	6338
ARFGAP3	3.77E-04	0	6339
ZNF469	3.76E-04	0	6340
DNAJB8	3.76E-04	0	6341
TTI2	3.75E-04	0	6342
CELSR2	3.75E-04	0	6343
TPTE	3.73E-04	0	6344
ADIG	3.73E-04	0	6345
CYP4X1	3.73E-04	0	6346
IDI2	3.73E-04	0	6347
SYT5	3.73E-04	0	6348
MPST	3.71E-04	0	6349
SAFB2	3.71E-04	0	6350
ORM2	3.71E-04	0	6351
OPCML	3.68E-04	0	6352
RPL27	3.67E-04	0	6353
VPS8	3.67E-04	0	6354
CRLS1	3.66E-04	0	6355
EVI5L	3.66E-04	0	6356
PSORS9	3.65E-04	0	6357
FAM109A	3.65E-04	0	6358
MED31	3.64E-04	0	6359

TTC37	3.64E-04	0	6360
KCNJ10	3.64E-04	0	6361
SLC13A1	3.64E-04	0	6362
MIR1231	3.64E-04	0	6363
CYP2D7P	3.63E-04	0	6364
DRGX	3.63E-04	0	6365
PPP3CB	3.63E-04	0	6366
PODN	3.63E-04	0	6367
PUM2	3.62E-04	0	6368
KMT2C	3.61E-04	0	6369
ZBTB1	3.60E-04	0	6370
POMT1	3.59E-04	0	6371
ELMOD2	3.59E-04	0	6372
HLA-DRB1	3.59E-04	0	6373
AA2	3.59E-04	0	6374
PRB3	3.58E-04	0	6375
HERC3	3.58E-04	0	6376
WDR36	3.58E-04	0	6377
RIBC2	3.56E-04	0	6378
RPL9	3.56E-04	0	6379
MIR506	3.56E-04	0	6380
GGCT	3.55E-04	0	6381
CDK11A	3.55E-04	0	6382
HSD17B4	3.54E-04	0	6383
TPRKB	3.52E-04	0	6384
TBC1D20	3.52E-04	0	6385
MAN1A1	3.51E-04	0	6386
DFNB68	3.51E-04	0	6387
DRG1	3.51E-04	0	6388
KIR2DL5B	3.51E-04	0	6389
GFPT2	3.51E-04	0	6390
TSPAN3	3.50E-04	0	6391
ZNF467	3.49E-04	0	6392
ARPC4	3.48E-04	0	6393
BOLA2	3.48E-04	0	6394
NEK7	3.48E-04	0	6395
ACE3P	3.47E-04	0	6396
RHBDD3	3.47E-04	0	6397
SF3A3	3.47E-04	0	6398
NUP54	3.44E-04	0	6399
HAB1	3.43E-04	0	6400
S12	3.43E-04	0	6401
MED24	3.43E-04	0	6402

LCO	3.43E-04	0	6403
MIR539	3.40E-04	0	6404
KRT82	3.40E-04	0	6405
SPTBN2	3.39E-04	0	6406
ALDH7A1	3.38E-04	0	6407
ENPP7	3.38E-04	0	6408
LIPH	3.38E-04	0	6409
UBA1	3.37E-04	0	6410
AMHR2	3.36E-04	0	6411
SGCD	3.35E-04	0	6412
SESTD1	3.35E-04	0	6413
MFSD4	3.34E-04	0	6414
TMEM119	3.34E-04	0	6415
ZBTB24	3.34E-04	0	6416
ZKSCAN4	3.34E-04	0	6417
SAMD8	3.33E-04	0	6418
IFNLR1	3.33E-04	0	6419
SYTL1	3.33E-04	0	6420
IL36G	3.31E-04	0	6421
CCAR1	3.31E-04	0	6422
ETHE1	3.31E-04	0	6423
FAM83G	3.30E-04	0	6424
PIGC	3.30E-04	0	6425
SUPT16H	3.30E-04	0	6426
CDYL	3.29E-04	0	6427
FOXO3B	3.29E-04	0	6428
RAP2C	3.29E-04	0	6429
AMN	3.29E-04	0	6430
GCNT7	3.28E-04	0	6431
BP37	3.28E-04	0	6432
DAOA-AS1	3.28E-04	0	6433
MIR4775	3.27E-04	0	6434
KIF3C	3.27E-04	0	6435
RGPD5	3.26E-04	0	6436
PHF17	3.26E-04	0	6437
FOXK1	3.25E-04	0	6438
DOK5	3.25E-04	0	6439
MIR551B	3.25E-04	0	6440
SLC22A10	3.24E-04	0	6441
CDC42SE1	3.24E-04	0	6442
ENC1	3.24E-04	0	6443
MLF1IP	3.23E-04	0	6444
CYP3A7	3.23E-04	0	6445

AWAT1	3.23E-04	0	6446
MIR4782	3.23E-04	0	6447
EPRS	3.23E-04	0	6448
TUBB4A	3.21E-04	0	6449
TSC22D4	3.19E-04	0	6450
IGKV6D-41	3.18E-04	0	6451
GPR148	3.18E-04	0	6452
COG3	3.17E-04	0	6453
TFCP2	3.17E-04	0	6454
ATCAY	3.17E-04	0	6455
SLC6A15	3.17E-04	0	6456
PRDM9	3.16E-04	0	6457
KCNF1	3.16E-04	0	6458
BFIC	3.16E-04	0	6459
CDC42EP4	3.16E-04	0	6460
SI	3.16E-04	0	6461
MIR656	3.15E-04	0	6462
SRM	3.15E-04	0	6463
DES	3.14E-04	0	6464
CD302	3.14E-04	0	6465
SULT1C2	3.14E-04	0	6466
PIGQ	3.13E-04	0	6467
G7P1	3.13E-04	0	6468
PPP4R2	3.11E-04	0	6469
TAF6L	3.11E-04	0	6470
MAN2B1	3.11E-04	0	6471
IFNL2	3.11E-04	0	6472
RANBP3	3.10E-04	0	6473
LIME1	3.09E-04	0	6474
ZNF750	3.08E-04	0	6475
CHODL	3.08E-04	0	6476
VEPH1	3.07E-04	0	6477
ABCA12	3.07E-04	0	6478
SYN3	3.05E-04	0	6479
SPAG11A	3.05E-04	0	6480
NME3	3.05E-04	0	6481
VPS28	3.03E-04	0	6482
STX16	3.02E-04	0	6483
XPNPEP2	3.01E-04	0	6484
CYP2A	3.01E-04	0	6485
PRKCDBP	2.99E-04	0	6486
CCL8	2.98E-04	0	6487
SNORD27	2.97E-04	0	6488

XIRP2	2.97E-04	0	6489
ZNF16	2.97E-04	0	6490
PLD6	2.95E-04	0	6491
MTMR9	2.95E-04	0	6492
MTMR4	2.95E-04	0	6493
STK25	2.94E-04	0	6494
MAPK15	2.94E-04	0	6495
SRPK3	2.94E-04	0	6496
METAP1	2.94E-04	0	6497
MUC6	2.94E-04	0	6498
UBE2E3	2.94E-04	0	6499
SLC39A10	2.93E-04	0	6500
POFUT2	2.92E-04	0	6501
CRELD2	2.91E-04	0	6502
ABCA5	2.90E-04	0	6503
PKN3	2.89E-04	0	6504
FNTB	2.89E-04	0	6505
PLA2G2C	2.88E-04	0	6506
C12ORF57	2.88E-04	0	6507
CAPN8	2.88E-04	0	6508
FAM20C	2.87E-04	0	6509
CALCOCO1	2.87E-04	0	6510
CLCA3P	2.87E-04	0	6511
MIR432	2.87E-04	0	6512
KLHL7	2.87E-04	0	6513
GRM7	2.87E-04	0	6514
DHDH	2.87E-04	0	6515
GPR171	2.86E-04	0	6516
CABS1	2.86E-04	0	6517
NPAS1	2.86E-04	0	6518
PLA2G3	2.86E-04	0	6519
RPL27A	2.86E-04	0	6520
GLRA2	2.85E-04	0	6521
KLF10	2.84E-04	0	6522
NDUFA6	2.84E-04	0	6523
XKR3	2.84E-04	0	6524
ADCY8	2.82E-04	0	6525
GDF3	2.82E-04	0	6526
TXNDC12	2.82E-04	0	6527
OTUD4	2.82E-04	0	6528
PHF10	2.81E-04	0	6529
FEZ1	2.81E-04	0	6530
LYPD4	2.81E-04	0	6531

LCLAT1	2.81E-04	0	6532
LCN1	2.80E-04	0	6533
PFKFB2	2.80E-04	0	6534
ZNF436	2.80E-04	0	6535
KRTAP1-1	2.79E-04	0	6536
RBKS	2.78E-04	0	6537
JADE2	2.78E-04	0	6538
STX10	2.77E-04	0	6539
TLX3	2.77E-04	0	6540
STIP1	2.77E-04	0	6541
GBP5	2.76E-04	0	6542
NAT6	2.76E-04	0	6543
MTERF1	2.76E-04	0	6544
TCFL5	2.75E-04	0	6545
UBAC1	2.75E-04	0	6546
MIR1238	2.74E-04	0	6547
CRNKL1	2.74E-04	0	6548
SULT1A2	2.73E-04	0	6549
BCL2L10	2.72E-04	0	6550
ACBD5	2.72E-04	0	6551
TIMM21	2.72E-04	0	6552
MRPL41	2.71E-04	0	6553
ACOT13	2.71E-04	0	6554
SPTLC3	2.71E-04	0	6555
HECTD1	2.71E-04	0	6556
UTRN	2.70E-04	0	6557
MNS1	2.70E-04	0	6558
PCIF1	2.70E-04	0	6559
ZAN	2.69E-04	0	6560
TTLL5	2.69E-04	0	6561
LRRTM4	2.68E-04	0	6562
RHOBTB2	2.67E-04	0	6563
HYALP1	2.67E-04	0	6564
HMU	2.67E-04	0	6565
MYL1	2.66E-04	0	6566
GUCA2B	2.66E-04	0	6567
MIR592	2.65E-04	0	6568
BAD	2.65E-04	0	6569
GAS7	2.65E-04	0	6570
NPRL2	2.64E-04	0	6571
GNRHR2	2.62E-04	0	6572
MAGT1	2.62E-04	0	6573
CYFIP1	2.61E-04	0	6574

UFL1	2.61E-04	0	6575
GNPNAT1	2.61E-04	0	6576
CHRM2	2.61E-04	0	6577
CNNM2	2.60E-04	0	6578
ALKBH8	2.60E-04	0	6579
HIST1H1B	2.60E-04	0	6580
MBTD1	2.60E-04	0	6581
ALX3	2.59E-04	0	6582
TMEM147	2.59E-04	0	6583
RNU6ATAC	2.59E-04	0	6584
MIR367	2.59E-04	0	6585
ALOX12B	2.59E-04	0	6586
LDHD	2.59E-04	0	6587
ARMC10	2.58E-04	0	6588
ADAP2	2.58E-04	0	6589
STARD10	2.58E-04	0	6590
COG4	2.58E-04	0	6591
SRGAP2	2.58E-04	0	6592
ALX1	2.58E-04	0	6593
CYP2F1	2.58E-04	0	6594
CCDC67	2.57E-04	0	6595
KLB	2.57E-04	0	6596
SYT14	2.57E-04	0	6597
KCNK18	2.56E-04	0	6598
SPATA4	2.56E-04	0	6599
TPM2	2.56E-04	0	6600
CLSTN3	2.55E-04	0	6601
FUNDC2	2.54E-04	0	6602
CCDC110	2.54E-04	0	6603
CRNN	2.54E-04	0	6604
ST7	2.54E-04	0	6605
CHMP4C	2.53E-04	0	6606
RFX3	2.53E-04	0	6607
ONECUT3	2.53E-04	0	6608
NNAT	2.52E-04	0	6609
PSCA	2.51E-04	0	6610
OPLAH	2.51E-04	0	6611
RAB15	2.51E-04	0	6612
PLEKHA8	2.50E-04	0	6613
SDAD1	2.48E-04	0	6614
RAPGEF4	2.48E-04	0	6615
DMRTA2	2.48E-04	0	6616
UQCC1	2.48E-04	0	6617

TBC1D16	2.48E-04	0	6618
PKD1L3	2.47E-04	0	6619
NOP14	2.47E-04	0	6620
KCNA5	2.47E-04	0	6621
MFAP5	2.47E-04	0	6622
UBR7	2.46E-04	0	6623
BOP1	2.46E-04	0	6624
LLGL1	2.45E-04	0	6625
CALML5	2.45E-04	0	6626
HIST2H3A	2.43E-04	0	6627
MIR1261	2.43E-04	0	6628
NDUFB6	2.43E-04	0	6629
IGF2BP1	2.41E-04	0	6630
SCAND1	2.41E-04	0	6631
VTRNA2-1	2.40E-04	0	6632
CXXC4	2.40E-04	0	6633
HCFC2	2.39E-04	0	6634
CCRL2	2.38E-04	0	6635
COL4A2	2.38E-04	0	6636
CPN1	2.37E-04	0	6637
NTNG1	2.37E-04	0	6638
PURG	2.36E-04	0	6639
SERHL	2.36E-04	0	6640
POLR2F	2.36E-04	0	6641
PAFC	2.36E-04	0	6642
ZMYND8	2.36E-04	0	6643
AOX2P	2.35E-04	0	6644
SNRNP35	2.35E-04	0	6645
PAPSS2	2.35E-04	0	6646
KIF18B	2.35E-04	0	6647
GIMAP5	2.35E-04	0	6648
IGKV2D-28	2.34E-04	0	6649
AARS2	2.34E-04	0	6650
MKI67	2.33E-04	0	6651
SH2D3A	2.33E-04	0	6652
EMX1	2.32E-04	0	6653
AASS	2.32E-04	0	6654
HS6ST1	2.30E-04	0	6655
SOBP	2.30E-04	0	6656
SVIP	2.29E-04	0	6657
GALNT10	2.29E-04	0	6658
ITPKC	2.28E-04	0	6659
INPP5A	2.27E-04	0	6660

AGR3	2.26E-04	0	6661
EMSY	2.26E-04	0	6662
SIX5	2.26E-04	0	6663
PPP2R5B	2.25E-04	0	6664
TTLL11	2.25E-04	0	6665
DNAI1	2.25E-04	0	6666
DYNLRB2	2.25E-04	0	6667
EIF2B4	2.25E-04	0	6668
GGA2	2.25E-04	0	6669
PLEKHF2	2.24E-04	0	6670
EXOSC4	2.24E-04	0	6671
TPST2	2.23E-04	0	6672
CPSF1	2.23E-04	0	6673
COPS2	2.23E-04	0	6674
COX19	2.23E-04	0	6675
CHMP5	2.22E-04	0	6676
FASTKD2	2.22E-04	0	6677
BIK	2.22E-04	0	6678
ADSS	2.22E-04	0	6679
ADFN	2.22E-04	0	6680
PARP9	2.22E-04	0	6681
CLIC3	2.21E-04	0	6682
SLC29A3	2.21E-04	0	6683
COL27A1	2.20E-04	0	6684
CMT2G	2.20E-04	0	6685
GTPBP2	2.19E-04	0	6686
PCYT1B	2.19E-04	0	6687
CDHR1	2.18E-04	0	6688
BPY2	2.18E-04	0	6689
NDST3	2.17E-04	0	6690
MDP1	2.17E-04	0	6691
ZNF175	2.17E-04	0	6692
3-Sep	2.16E-04	0	6693
FENDRR	2.14E-04	0	6694
PCDHB5	2.14E-04	0	6695
ANCR	2.14E-04	0	6696
MIRLET7C	2.14E-04	0	6697
DNAH3	2.13E-04	0	6698
MYO16	2.13E-04	0	6699
WIBG	2.13E-04	0	6700
BAZ1A	2.13E-04	0	6701
S1PR2	2.12E-04	0	6702
IL36A	2.12E-04	0	6703

MIR938	2.12E-04	0	6704
RNR2	2.11E-04	0	6705
MGAT2	2.11E-04	0	6706
SEC22B	2.11E-04	0	6707
DMGDH	2.10E-04	0	6708
MYOZ1	2.10E-04	0	6709
SNORD14C	2.09E-04	0	6710
MCTP2	2.08E-04	0	6711
EDF1	2.08E-04	0	6712
HYAL4	2.08E-04	0	6713
ANO3	2.08E-04	0	6714
TRIM67	2.08E-04	0	6715
WTIP	2.07E-04	0	6716
AKAP9	2.07E-04	0	6717
BRF2	2.06E-04	0	6718
SH3GL1	2.06E-04	0	6719
SGSH	2.06E-04	0	6720
ANBC	2.06E-04	0	6721
NDUFS4	2.05E-04	0	6722
AURKAIP1	2.05E-04	0	6723
FKBP6	2.05E-04	0	6724
SLC1A4	2.05E-04	0	6725
BW15	2.04E-04	0	6726
CCDC54	2.03E-04	0	6727
FOLR4	2.03E-04	0	6728
SEC24C	2.03E-04	0	6729
MAP7D1	2.03E-04	0	6730
RNF167	2.03E-04	0	6731
CTNS	2.03E-04	0	6732
ARAP2	2.03E-04	0	6733
NDUFS8	2.03E-04	0	6734
FBXL17	2.02E-04	0	6735
UBE2J2	2.02E-04	0	6736
PIGH	2.01E-04	0	6737
FRAXE	2.01E-04	0	6738
ILDR2	2.01E-04	0	6739
USP49	2.01E-04	0	6740
GSDMB	2.00E-04	0	6741
RERE	2.00E-04	0	6742
IFNA6	2.00E-04	0	6743
SMTNL2	2.00E-04	0	6744
HIST1H2BN	1.99E-04	0	6745
RPTN	1.99E-04	0	6746

MYADM	1.99E-04	0	6747
CATSPER2	1.98E-04	0	6748
ITPKA	1.98E-04	0	6749
LRRTM3	1.98E-04	0	6750
TAS2R46	1.98E-04	0	6751
NXPH1	1.98E-04	0	6752
SLC25A25	1.98E-04	0	6753
MGME1	1.97E-04	0	6754
INPP1	1.97E-04	0	6755
CERS3	1.95E-04	0	6756
EXOC3L1	1.95E-04	0	6757
RPS6KB3	1.94E-04	0	6758
ITGB5	1.94E-04	0	6759
SMEK3P	1.94E-04	0	6760
CCNG2	1.93E-04	0	6761
KYNU	1.92E-04	0	6762
BROX	1.92E-04	0	6763
FRMD4A	1.92E-04	0	6764
ROPN1	1.92E-04	0	6765
EJM2	1.92E-04	0	6766
KCNK7	1.91E-04	0	6767
LIPB	1.91E-04	0	6768
DNAJC10	1.90E-04	0	6769
DAND5	1.89E-04	0	6770
DDX42	1.89E-04	0	6771
RGP1	1.89E-04	0	6772
GNAL	1.87E-04	0	6773
S100A1	1.87E-04	0	6774
ST3GAL4	1.86E-04	0	6775
ALG10	1.86E-04	0	6776
PFN3	1.85E-04	0	6777
PKIB	1.84E-04	0	6778
IRG1	1.84E-04	0	6779
CCT3	1.82E-04	0	6780
IKBIP	1.80E-04	0	6781
ALL2	1.80E-04	0	6782
ZCCHC12	1.78E-04	0	6783
ALG2	1.78E-04	0	6784
PFKL	1.78E-04	0	6785
KIF6	1.77E-04	0	6786
TOE1	1.77E-04	0	6787
GBP3	1.77E-04	0	6788
SNORD3B-1	1.77E-04	0	6789

TRIM35	1.77E-04	0	6790
VDAC3	1.77E-04	0	6791
CA11	1.77E-04	0	6792
IGHV4-39	1.77E-04	0	6793
TBC1D13	1.77E-04	0	6794
TNKS2	1.76E-04	0	6795
MGST2	1.76E-04	0	6796
CDSN	1.76E-04	0	6797
QSOX1	1.76E-04	0	6798
RA2	1.75E-04	0	6799
BUD13	1.74E-04	0	6800
ABT1	1.74E-04	0	6801
APPBP2	1.74E-04	0	6802
EPPIN	1.72E-04	0	6803
TLE2	1.72E-04	0	6804
CACNA1S	1.72E-04	0	6805
PSMG1	1.71E-04	0	6806
DLST	1.71E-04	0	6807
WDR6	1.71E-04	0	6808
KIF1C	1.70E-04	0	6809
PPAPDC2	1.70E-04	0	6810
RAB26	1.70E-04	0	6811
RAB33A	1.70E-04	0	6812
CDH4	1.70E-04	0	6813
BAP18	1.70E-04	0	6814
TRNAG1	1.69E-04	0	6815
NDUFS2	1.69E-04	0	6816
GCN1L1	1.67E-04	0	6817
MT-ATP6	1.67E-04	0	6818
ATP5B	1.67E-04	0	6819
PGLYRP4	1.67E-04	0	6820
PGLYRP3	1.67E-04	0	6821
UQCR10	1.66E-04	0	6822
FAAH2	1.66E-04	0	6823
LMX1B	1.65E-04	0	6824
FAM35A	1.65E-04	0	6825
NIPAL1	1.65E-04	0	6826
SCARF2	1.64E-04	0	6827
LIMS2	1.64E-04	0	6828
EDEM3	1.64E-04	0	6829
MAPK4	1.64E-04	0	6830
RPL28	1.63E-04	0	6831
SERPINB6	1.63E-04	0	6832

PHF3	1.63E-04	0	6833
HID1	1.62E-04	0	6834
RQCD1	1.62E-04	0	6835
MAGOHB	1.62E-04	0	6836
USP9Y	1.61E-04	0	6837
NKAIN2	1.60E-04	0	6838
FILIP1	1.60E-04	0	6839
SPRED3	1.59E-04	0	6840
MGCT	1.58E-04	0	6841
SERAC1	1.58E-04	0	6842
FOXN3	1.56E-04	0	6843
TMEM8A	1.56E-04	0	6844
WDR26	1.55E-04	0	6845
LAMB3	1.55E-04	0	6846
RPL12P1	1.54E-04	0	6847
CYP21A1P	1.53E-04	0	6848
LSM14A	1.52E-04	0	6849
FCRLA	1.52E-04	0	6850
ARSA	1.52E-04	0	6851
DOC2A	1.51E-04	0	6852
HMG20A	1.51E-04	0	6853
DSN1	1.51E-04	0	6854
1-Sep	1.51E-04	0	6855
SHCBP1	1.51E-04	0	6856
SRIP1	1.51E-04	0	6857
GTPBP3	1.51E-04	0	6858
VKORC1L1	1.50E-04	0	6859
MIR1269A	1.50E-04	0	6860
MTO1	1.50E-04	0	6861
HSD17B12	1.49E-04	0	6862
CERS2	1.49E-04	0	6863
MIR3666	1.48E-04	0	6864
MTCL1	1.47E-04	0	6865
FMN2	1.47E-04	0	6866
ASTL	1.47E-04	0	6867
TNPO2	1.47E-04	0	6868
TCF7L1	1.46E-04	0	6869
USP17	1.46E-04	0	6870
RIPPLY1	1.45E-04	0	6871
COG1	1.44E-04	0	6872
TBC1D15	1.44E-04	0	6873
MARS2	1.44E-04	0	6874
PTPRR	1.43E-04	0	6875

RPS4X	1.43E-04	0	6876
SMG7	1.43E-04	0	6877
ABCC13	1.42E-04	0	6878
IDDM11	1.41E-04	0	6879
DROSHA	1.40E-04	0	6880
PSME1	1.40E-04	0	6881
KLHL15	1.40E-04	0	6882
RNU3P4	1.39E-04	0	6883
G6B	1.39E-04	0	6884
UGT2A1	1.38E-04	0	6885
ADAM29	1.38E-04	0	6886
WM1	1.38E-04	0	6887
DUSP12	1.38E-04	0	6888
ZFAND6	1.37E-04	0	6889
SERPINA11	1.37E-04	0	6890
RPS6KC1	1.36E-04	0	6891
DACH1	1.36E-04	0	6892
DNAJB3	1.35E-04	0	6893
SEMA6B	1.35E-04	0	6894
PIGN	1.35E-04	0	6895
RHBDD1	1.35E-04	0	6896
VCF	1.34E-04	0	6897
ME2	1.34E-04	0	6898
TDRD3	1.33E-04	0	6899
ARRDC3	1.33E-04	0	6900
FAM189B	1.33E-04	0	6901
ZNF155	1.32E-04	0	6902
LUC7L	1.32E-04	0	6903
RBM25	1.32E-04	0	6904
BMP10	1.32E-04	0	6905
TAF11	1.32E-04	0	6906
DFNB60	1.31E-04	0	6907
JPH1	1.30E-04	0	6908
ZNF28	1.30E-04	0	6909
ZNF30	1.30E-04	0	6910
RAB20	1.29E-04	0	6911
PPIL3	1.29E-04	0	6912
IP6K3	1.29E-04	0	6913
PPCDC	1.29E-04	0	6914
GUCA1B	1.29E-04	0	6915
VPS37A	1.28E-04	0	6916
PDE9A	1.28E-04	0	6917
MED26	1.28E-04	0	6918

TRAPPC12	1.28E-04	0	6919
UROS	1.27E-04	0	6920
FANK1	1.26E-04	0	6921
CYP1D1P	1.25E-04	0	6922
NSMCE2	1.25E-04	0	6923
TXNL4A	1.24E-04	0	6924
ZPR1	1.24E-04	0	6925
LAMC3	1.23E-04	0	6926
ARL6IP4	1.22E-04	0	6927
ALKBH5	1.22E-04	0	6928
CHST10	1.22E-04	0	6929
AKIRIN1	1.21E-04	0	6930
SSBP1	1.21E-04	0	6931
ADAT3	1.20E-04	0	6932
PHF6	1.20E-04	0	6933
HSDL2	1.20E-04	0	6934
SNORD20	1.20E-04	0	6935
WDR12	1.20E-04	0	6936
TRA2B	1.19E-04	0	6937
DBR1	1.19E-04	0	6938
SNORD33	1.19E-04	0	6939
TAPBPL	1.19E-04	0	6940
RASIP1	1.19E-04	0	6941
IGHV3-23	1.18E-04	0	6942
TENM3	1.18E-04	0	6943
PCYOX1	1.18E-04	0	6944
WDR47	1.18E-04	0	6945
MIR374A	1.18E-04	0	6946
POU6F2	1.17E-04	0	6947
PPP1R11	1.17E-04	0	6948
NKIRAS1	1.17E-04	0	6949
ANKS3	1.17E-04	0	6950
ZNF804A	1.17E-04	0	6951
CKLF	1.16E-04	0	6952
SLC6A19	1.16E-04	0	6953
SNX4	1.15E-04	0	6954
NRXN1	1.15E-04	0	6955
OSTM1	1.15E-04	0	6956
TRNAT1	1.15E-04	0	6957
MIR3613	1.13E-04	0	6958
TM6SF2	1.12E-04	0	6959
SIPA1L1	1.12E-04	0	6960
ABCG4	1.11E-04	0	6961

SAPCD2	1.10E-04	0	6962
EIF2C1	1.08E-04	0	6963
TCP1	1.07E-04	0	6964
GTF3C2	1.07E-04	0	6965
ASPN	1.07E-04	0	6966
PRKAR2A	1.06E-04	0	6967
COG7	1.06E-04	0	6968
KLC3	1.05E-04	0	6969
COL6A3	1.05E-04	0	6970
SMOC2	1.05E-04	0	6971
KIF3B	1.05E-04	0	6972
GPR37L1	1.03E-04	0	6973
MLYCD	1.02E-04	0	6974
LRRC26	1.01E-04	0	6975
GLC1M	1.01E-04	0	6976
FAM13A	1.00E-04	0	6977
COL4A6	1.00E-04	0	6978
DUH	1.00E-04	0	6979
RANBP10	1.00E-04	0	6980
RPL6	9.98E-05	0	6981
STX3	9.98E-05	0	6982
IL36B	9.98E-05	0	6983
NT5C3B	9.96E-05	0	6984
TRIM55	9.90E-05	0	6985
SPANXB1	9.86E-05	0	6986
SPANXD	9.86E-05	0	6987
SPANXN1	9.86E-05	0	6988
SPANXN2	9.86E-05	0	6989
SPANXN3	9.86E-05	0	6990
SPANXN4	9.86E-05	0	6991
CGB2	9.85E-05	0	6992
SPANXA2	9.84E-05	0	6993
ATP6	9.75E-05	0	6994
IGKV@	9.72E-05	0	6995
MACST	9.72E-05	0	6996
WASH1	9.72E-05	0	6997
PRC1	9.70E-05	0	6998
GRM6	9.69E-05	0	6999
SYCP2	9.65E-05	0	7000
DLGAP4	9.55E-05	0	7001
UBA52	9.46E-05	0	7002
MTMR14	9.45E-05	0	7003
MPC1L	9.42E-05	0	7004

MAGEA12	9.34E-05	0	7005
COA1	9.29E-05	0	7006
CHURC1	9.28E-05	0	7007
MESP2	9.23E-05	0	7008
E2F8	9.19E-05	0	7009
FBXL3	9.18E-05	0	7010
CA5A	9.18E-05	0	7011
OGDH	9.16E-05	0	7012
TENM2	9.10E-05	0	7013
MSL3	9.09E-05	0	7014
EPS8L1	9.09E-05	0	7015
EPS8L2	9.09E-05	0	7016
EPS8L3	9.09E-05	0	7017
AGA2	9.08E-05	0	7018
CES1	9.08E-05	0	7019
VPS4A	9.02E-05	0	7020
INHBA	9.00E-05	0	7021
EPN2	8.96E-05	0	7022
WFS1	8.94E-05	0	7023
SPACA3	8.93E-05	0	7024
NXNL1	8.85E-05	0	7025
LARGE	8.83E-05	0	7026
SEMA4A	8.81E-05	0	7027
XG	8.79E-05	0	7028
TONSL	8.77E-05	0	7029
VSTM1	8.73E-05	0	7030
KIR2DS5	8.73E-05	0	7031
ESF1	8.72E-05	0	7032
DYX8	8.69E-05	0	7033
EVC	8.68E-05	0	7034
LCA5	8.67E-05	0	7035
MGAT5B	8.62E-05	0	7036
IGKV6-21	8.62E-05	0	7037
GPR64	8.60E-05	0	7038
C8ORF17	8.59E-05	0	7039
IDI1	8.56E-05	0	7040
ACVR1C	8.55E-05	0	7041
SNORA54	8.54E-05	0	7042
JMJD1C	8.51E-05	0	7043
MAD1L1	8.49E-05	0	7044
PARK16	8.45E-05	0	7045
OCR1	8.39E-05	0	7046
LY6G6D	8.36E-05	0	7047

TIMM8B	8.34E-05	0	7048
MRC2	8.31E-05	0	7049
POTEE	8.22E-05	0	7050
SULT2B1	8.22E-05	0	7051
PTPRH	8.22E-05	0	7052
CIRBP	8.22E-05	0	7053
KIR2DS1	8.22E-05	0	7054
EMR2	8.18E-05	0	7055
FCHSD2	8.04E-05	0	7056
DARS	8.03E-05	0	7057
CBR4	7.97E-05	0	7058
ACKR3	7.90E-05	0	7059
ELP2	7.86E-05	0	7060
RPRM	7.83E-05	0	7061
GCC1	7.80E-05	0	7062
NAA60	7.66E-05	0	7063
LYZL6	7.65E-05	0	7064
HIST1H2AE	7.64E-05	0	7065
AP4B1	7.63E-05	0	7066
S11	7.59E-05	0	7067
APOBEC3H	7.56E-05	0	7068
ADTRP	7.55E-05	0	7069
ANKS6	7.53E-05	0	7070
SHOX	7.51E-05	0	7071
CHERP	7.48E-05	0	7072
CLNS1A	7.40E-05	0	7073
CAMK1G	7.38E-05	0	7074
CSTF3	7.30E-05	0	7075
TCEAL7	7.27E-05	0	7076
ARPC5	7.25E-05	0	7077
YKT6	7.22E-05	0	7078
NADK2	7.19E-05	0	7079
RPL36	7.15E-05	0	7080
CLEC2B	7.15E-05	0	7081
INIP	7.11E-05	0	7082
MIR30B	7.10E-05	0	7083
ZNF629	7.06E-05	0	7084
SELRC1	7.05E-05	0	7085
PCF11	7.01E-05	0	7086
PAFAH1B2	6.99E-05	0	7087
GOLPH3	6.96E-05	0	7088
C2ORF71	6.96E-05	0	7089
GLCCI1	6.93E-05	0	7090

ATG9A	6.91E-05	0	7091
PKD1P1	6.91E-05	0	7092
IGKV2-40	6.89E-05	0	7093
SNAI3	6.82E-05	0	7094
LZTR1	6.81E-05	0	7095
DNAI2	6.77E-05	0	7096
G0S2	6.72E-05	0	7097
TCEA3	6.65E-05	0	7098
FBXL14	6.56E-05	0	7099
FOXD4L4	6.54E-05	0	7100
HAAO	6.54E-05	0	7101
MTHFD1	6.53E-05	0	7102
POLR2C	6.49E-05	0	7103
IGKV2D-36	6.49E-05	0	7104
UBA5	6.48E-05	0	7105
PDZD7	6.41E-05	0	7106
HDGFRP3	6.41E-05	0	7107
UPRT	6.31E-05	0	7108
COG5	6.29E-05	0	7109
NOL4	6.26E-05	0	7110
LSM10	6.22E-05	0	7111
SHKBP1	6.21E-05	0	7112
MIR935	6.18E-05	0	7113
ATP6V1B2	6.17E-05	0	7114
HCP5	6.12E-05	0	7115
DTD1	6.08E-05	0	7116
NFS1	6.05E-05	0	7117
SLC35C2	6.03E-05	0	7118
FRAS1	6.03E-05	0	7119
UAP1	6.01E-05	0	7120
NPCDR1	5.95E-05	0	7121
PRPF18	5.95E-05	0	7122
SLC23A3	5.91E-05	0	7123
TRNAG2	5.87E-05	0	7124
PDE4C	5.85E-05	0	7125
AGTRAP	5.83E-05	0	7126
HEATR5B	5.82E-05	0	7127
PLEKHH3	5.82E-05	0	7128
ALKBH4	5.75E-05	0	7129
TSSK6	5.69E-05	0	7130
SLC34A3	5.69E-05	0	7131
SNORD116@	5.64E-05	0	7132
SPRR3	5.55E-05	0	7133

CLTCL1	5.49E-05	0	7134
PKIA	5.48E-05	0	7135
OSR1	5.39E-05	0	7136
IL1R2	5.39E-05	0	7137
LRRC4C	5.39E-05	0	7138
PGAP1	5.34E-05	0	7139
ZBTB25	5.33E-05	0	7140
GTF3C3	5.33E-05	0	7141
PSMF1	5.32E-05	0	7142
AFG3L1P	5.31E-05	0	7143
PLP2	5.31E-05	0	7144
TSTA3	5.23E-05	0	7145
MIR638	5.23E-05	0	7146
DHRS7C	5.19E-05	0	7147
TRNAG4	5.19E-05	0	7148
SPSB4	5.19E-05	0	7149
SS18	5.15E-05	0	7150
BMP8A	5.15E-05	0	7151
SYM2	5.13E-05	0	7152
HCP5B	5.04E-05	0	7153
DIRAS2	5.00E-05	0	7154
IGKV1-33	5.00E-05	0	7155
BCDIN3D	4.97E-05	0	7156
SERPINB5	4.94E-05	0	7157
PRDM13	4.92E-05	0	7158
KIAA1199	4.85E-05	0	7159
BBS10	4.85E-05	0	7160
ATP8A1	4.85E-05	0	7161
C6ORF48	4.83E-05	0	7162
CHIC2	4.79E-05	0	7163
BEST4	4.79E-05	0	7164
LPIN2	4.78E-05	0	7165
ACOX2	4.71E-05	0	7166
ZG16B	4.70E-05	0	7167
OLFML3	4.67E-05	0	7168
CYB5R4	4.57E-05	0	7169
FLD1	4.52E-05	0	7170
GSTA5	4.51E-05	0	7171
SYNDIG1L	4.50E-05	0	7172
VRTN	4.50E-05	0	7173
YLPM1	4.50E-05	0	7174
ZNF354A	4.49E-05	0	7175
PRR7	4.42E-05	0	7176

ATP13A4	4.38E-05	0	7177
COL25A1	4.36E-05	0	7178
SCRN3	4.36E-05	0	7179
PCDH7	4.34E-05	0	7180
DHODH	4.34E-05	0	7181
COG8	4.31E-05	0	7182
PRKAB2	4.30E-05	0	7183
ZDHHC5	4.29E-05	0	7184
MIR770	4.27E-05	0	7185
FBXO39	4.27E-05	0	7186
MDFI	4.25E-05	0	7187
HOXD12	4.25E-05	0	7188
P2RX5	4.15E-05	0	7189
NEBL	4.15E-05	0	7190
ILDR1	4.10E-05	0	7191
MGAM	4.04E-05	0	7192
CORD8	4.03E-05	0	7193
CENPH	3.97E-05	0	7194
AARS	3.93E-05	0	7195
SKA1	3.90E-05	0	7196
BAZ1B	3.83E-05	0	7197
GADL1	3.80E-05	0	7198
LINC00052	3.78E-05	0	7199
AMPD3	3.77E-05	0	7200
RDH12	3.76E-05	0	7201
ETNK1	3.74E-05	0	7202
ACPT	3.73E-05	0	7203
PPAPDC1B	3.68E-05	0	7204
ALG11	3.66E-05	0	7205
IGKV1D-32	3.63E-05	0	7206
KRT2	3.63E-05	0	7207
ELP4	3.63E-05	0	7208
AMER2	3.60E-05	0	7209
TRNAG5	3.57E-05	0	7210
TRIM41	3.56E-05	0	7211
SLC24A6	3.56E-05	0	7212
DHX34	3.53E-05	0	7213
AGTPBP1	3.49E-05	0	7214
CLEC2A	3.48E-05	0	7215
CYP2G1P	3.45E-05	0	7216
CYP4F8	3.45E-05	0	7217
DNAH14	3.45E-05	0	7218
NCAPD3	3.45E-05	0	7219

PDE8B	3.38E-05	0	7220
CENPI	3.30E-05	0	7221
FSBP	3.30E-05	0	7222
ERGIC3	3.19E-05	0	7223
PRPF4	3.18E-05	0	7224
SNORA67	3.15E-05	0	7225
HCL2	3.14E-05	0	7226
TARS2	3.13E-05	0	7227
AGBL2	3.11E-05	0	7228
MIR645	3.09E-05	0	7229
KPNA2	3.02E-05	0	7230
TGM5	3.02E-05	0	7231
MEP1AP1	3.00E-05	0	7232
TMOD1	2.97E-05	0	7233
MIR505	2.93E-05	0	7234
CACTIN	2.92E-05	0	7235
CDHR5	2.91E-05	0	7236
GRID2IP	2.91E-05	0	7237
PGA4	2.90E-05	0	7238
TTC21B	2.90E-05	0	7239
ZNF33A	2.89E-05	0	7240
ZNF45	2.88E-05	0	7241
FOPNL	2.87E-05	0	7242
MIR362	2.86E-05	0	7243
ATP6V0A1	2.86E-05	0	7244
RBPMS2	2.85E-05	0	7245
ATXN10	2.82E-05	0	7246
GPAA1	2.81E-05	0	7247
PIGK	2.81E-05	0	7248
COQ3	2.78E-05	0	7249
EXOC6B	2.74E-05	0	7250
HOAC	2.70E-05	0	7251
TS13	2.69E-05	0	7252
RBL1	2.68E-05	0	7253
WRB	2.64E-05	0	7254
DNAJB12	2.61E-05	0	7255
DNASE1L3	2.59E-05	0	7256
ZHX3	2.59E-05	0	7257
FTH1P13	2.57E-05	0	7258
ALS2CL	2.55E-05	0	7259
IGLV3-19	2.52E-05	0	7260
BANF2	2.40E-05	0	7261
AMDP1	2.40E-05	0	7262

SMR3B	2.38E-05	0	7263
CASQ1	2.29E-05	0	7264
CNA1	2.28E-05	0	7265
CRYZL1	2.28E-05	0	7266
ADHFE1	2.26E-05	0	7267
CNKSR2	2.26E-05	0	7268
TMED9	2.23E-05	0	7269
RPL35A	2.16E-05	0	7270
TFEC	2.15E-05	0	7271
TLL2	2.13E-05	0	7272
NXF4	2.11E-05	0	7273
INPP5J	2.08E-05	0	7274
DEPP	2.08E-05	0	7275
OCTN3	2.07E-05	0	7276
FCGR1C	2.06E-05	0	7277
AATK	2.05E-05	0	7278
PRRT1	2.05E-05	0	7279
TPD52L2	2.04E-05	0	7280
SPG18	2.03E-05	0	7281
BBS5	1.95E-05	0	7282
RPL10P1	1.94E-05	0	7283
ADAMTS19	1.89E-05	0	7284
PDCD2L	1.88E-05	0	7285
ABCD4	1.84E-05	0	7286
CLDND1	1.83E-05	0	7287
GMPR	1.78E-05	0	7288
NCMAP	1.76E-05	0	7289
OSBPL9	1.74E-05	0	7290
TAAR3	1.71E-05	0	7291
TULP4	1.70E-05	0	7292
AP5B1	1.69E-05	0	7293
AKR1D1	1.69E-05	0	7294
SYNPO2L	1.68E-05	0	7295
ENPP6	1.67E-05	0	7296
TECTA	1.65E-05	0	7297
TRPV2	1.62E-05	0	7298
DOLK	1.62E-05	0	7299
GPN1	1.61E-05	0	7300
MTMR3	1.60E-05	0	7301
AKAP6	1.59E-05	0	7302
BID	1.58E-05	0	7303
12-Sep	1.58E-05	0	7304
SLC26A4	1.58E-05	0	7305

FA2H	1.57E-05	0	7306
MSL2	1.55E-05	0	7307
BCYRN1	1.55E-05	0	7308
PIGZ	1.55E-05	0	7309
DNPH1	1.50E-05	0	7310
GATAD2B	1.50E-05	0	7311
GPN3	1.49E-05	0	7312
ACER3	1.43E-05	0	7313
KIR3DS1	1.39E-05	0	7314
YARS2	1.37E-05	0	7315
FKBPL	1.37E-05	0	7316
WDR81	1.36E-05	0	7317
GTF2F2P1	1.32E-05	0	7318
CDS2	1.31E-05	0	7319
KLP6	1.29E-05	0	7320
CTPS2	1.28E-05	0	7321
TRANK1	1.28E-05	0	7322
CSTF2	1.26E-05	0	7323
MTBS	1.23E-05	0	7324
CCDC109B	1.21E-05	0	7325
NDUFAF1	1.19E-05	0	7326
ACAD10	1.18E-05	0	7327
ACAD11	1.18E-05	0	7328
VPS26B	1.17E-05	0	7329
RAD51AP1	1.16E-05	0	7330
PRPF38A	1.14E-05	0	7331
ANO5	1.14E-05	0	7332
DYX1	1.13E-05	0	7333
PRTG	1.13E-05	0	7334
CAPN3	1.12E-05	0	7335
FOXR2	1.12E-05	0	7336
WDR41	1.11E-05	0	7337
ZFP64	1.08E-05	0	7338
TMEM59	1.07E-05	0	7339
CC2D2A	1.03E-05	0	7340
EVX1-AS	1.02E-05	0	7341
HSDL1	1.02E-05	0	7342
ABCA6	1.02E-05	0	7343
ABCA8	1.02E-05	0	7344
TRAFD1	8.92E-06	0	7345
NUBPL	8.48E-06	0	7346
CTSF	8.12E-06	0	7347
BEX4	7.95E-06	0	7348

KRR1	7.89E-06	0	7349
HOXC12	7.80E-06	0	7350
MYL6B	7.66E-06	0	7351
MT-ND3	7.39E-06	0	7352
BAHD1	7.28E-06	0	7353
LDB2	7.21E-06	0	7354
MZT1	7.19E-06	0	7355
LRG1	7.10E-06	0	7356
SMPDL3B	6.91E-06	0	7357
SERTAD1	6.80E-06	0	7358
PDLIM1	6.62E-06	0	7359
GIPC3	6.56E-06	0	7360
ZNF19	6.50E-06	0	7361
ZNF44	6.50E-06	0	7362
FSCB	6.00E-06	0	7363
METTL22	5.74E-06	0	7364
PPP1R18	5.68E-06	0	7365
WNK3	5.61E-06	0	7366
FASTKD1	5.48E-06	0	7367
ALG5	5.47E-06	0	7368
PBX4	5.32E-06	0	7369
LCORL	5.31E-06	0	7370
B3GALT2	5.20E-06	0	7371
ENTPD6	5.20E-06	0	7372
HSPBAP1	5.20E-06	0	7373
IGLV11-55	5.20E-06	0	7374
TRNAG3	5.13E-06	0	7375
LSM5	5.00E-06	0	7376
LSM6	5.00E-06	0	7377
MIR101-1	4.68E-06	0	7378
TAL2	4.68E-06	0	7379
CC2D1B	4.68E-06	0	7380
SLC7A2	4.29E-06	0	7381
OPN5	4.25E-06	0	7382
AGK	4.22E-06	0	7383
RBM47	3.97E-06	0	7384
IFT88	3.91E-06	0	7385
EPB41L1	3.77E-06	0	7386
UFSP1	3.77E-06	0	7387
CARS	3.69E-06	0	7388
URM1	3.61E-06	0	7389
CCDC78	3.56E-06	0	7390
FER1L5	3.48E-06	0	7391

MT-TW	3.33E-06	0	7392
NREP	3.16E-06	0	7393
EXPH5	3.13E-06	0	7394
NARS2	3.01E-06	0	7395
TDRD7	2.96E-06	0	7396
CUTA	2.91E-06	0	7397
MYRIP	2.76E-06	0	7398
MT-TM	2.76E-06	0	7399
NDUFAF4	2.69E-06	0	7400
RNU4-2	2.54E-06	0	7401
MICU2	2.49E-06	0	7402
SNORD95	2.46E-06	0	7403
GUCA1C	2.41E-06	0	7404
CHI3L2	2.40E-06	0	7405
SLC6A14	2.28E-06	0	7406
B3GALT6	2.22E-06	0	7407
ISCA2	1.38E-06	0	7408
PHAX	1.37E-06	0	7409
ZFHX2	1.34E-06	0	7410
MYO19	1.32E-06	0	7411
SLC25A16	1.32E-06	0	7412
PPFIA4	1.31E-06	0	7413
SDF2L1	1.29E-06	0	7414
RFK	1.22E-06	0	7415
ALG13	1.18E-06	0	7416
ANO9	1.08E-06	0	7417
ITGA11	1.07E-06	0	7418
NDST2	1.02E-06	0	7419
IBGC1	8.51E-07	0	7420
CLIC2	8.33E-07	0	7421
MTMR7	8.18E-07	0	7422
COX14	7.49E-07	0	7423
TSEN15	7.41E-07	0	7424
CES3	6.89E-07	0	7425
NDUFV3	6.76E-07	0	7426
WBP11	6.63E-07	0	7427
RDH8	5.43E-07	0	7428
LRRC8B	5.11E-07	0	7429
LRRC8C	5.11E-07	0	7430
AGPAT2	4.52E-07	0	7431
CAPN5	2.52E-07	0	7432
LYRM7	2.37E-07	0	7433
CIB3	2.14E-07	0	7434

ZNF33B	1.19E-07	0	7435
ZNF37A	1.19E-07	0	7436
TSKS	6.49E-08	0	7437
PTPRQ	3.65E-34	0	7438
OTOGL	2.55E-34	0	7439
IGLC5	8.91E-37	0	7440
FRA12A	7.68E-37	0	7441
MRPL13	1.93E-37	0	7442
MRPL27	1.93E-37	0	7443
SNHG4	1.34E-37	0	7444
SNORA74A	1.34E-37	0	7445
TUBGCP2	1.31E-37	0	7446
UGT2B11	5.00E-38	0	7447
UGT2B28	5.00E-38	0	7448
FTSJ3	4.93E-38	0	7449
NIP7	4.93E-38	0	7450
C20ORF201	3.16E-38	0	7451
TMEM191C	3.16E-38	0	7452
KCNA10	1.72E-42	0	7453
COX15	1.65E-42	0	7454
TGIF2LY	1.53E-42	0	7455
SLC24A4	1.45E-42	0	7456
COBLL1	1.36E-42	0	7457
2-Mar	1.25E-42	0	7458
CLMN	1.15E-42	0	7459
ZNF277	1.14E-42	0	7460
XKR8	1.13E-42	0	7461
GSC2	1.10E-42	0	7462
IBTK	1.05E-42	0	7463
TRNAM2	1.01E-42	0	7464
MTERF4	9.96E-43	0	7465
SLC25A40	9.77E-43	0	7466
DCAF12L2	9.58E-43	0	7467
RA27	9.57E-43	0	7468
KCTD8	9.32E-43	0	7469
ZNF25	9.29E-43	0	7470
GUCY1A3	9.19E-43	0	7471
LUC7L2	9.19E-43	0	7472
ZNF496	9.15E-43	0	7473
RPL36AL	8.80E-43	0	7474
ADHD2	8.77E-43	0	7475
NSUN7	8.63E-43	0	7476
KRTAP24-1	8.55E-43	0	7477

HPLH1	8.34E-43	0	7478
H3.Y	8.23E-43	0	7479
BW58	7.92E-43	0	7480
CCIN	7.78E-43	0	7481
KCNJ18	7.57E-43	0	7482
NDST4	7.40E-43	0	7483
INTS1	7.08E-43	0	7484
UCK1	6.96E-43	0	7485
OTOS	6.74E-43	0	7486
TMEM66	6.50E-43	0	7487
DPY19L2	6.46E-43	0	7488
MEIG1	6.29E-43	0	7489
ELMOD1	6.28E-43	0	7490
SNORD55	6.26E-43	0	7491
MIR1304	6.17E-43	0	7492
CWC27	5.72E-43	0	7493
NT5M	5.62E-43	0	7494
NUDT11	5.30E-43	0	7495
EMC4	5.23E-43	0	7496
MYP15	5.07E-43	0	7497
WBSCR22	5.04E-43	0	7498
CCDC136	5.03E-43	0	7499
DHRS4L2	5.02E-43	0	7500
DEAR	4.97E-43	0	7501
PHBP3	4.70E-43	0	7502
POLR3B	4.68E-43	0	7503
WDR74	4.62E-43	0	7504
ZAR1L	4.53E-43	0	7505
CACD	4.33E-43	0	7506
TRAV26-2	4.33E-43	0	7507
TRAV15	4.16E-43	0	7508
TMEM38B	4.08E-43	0	7509
CDHR3	3.97E-43	0	7510
IDDM10	3.83E-43	0	7511
TAS2R39	3.48E-43	0	7512
ABHD15	3.35E-43	0	7513
KPTN	3.21E-43	0	7514
ACTBP2	3.12E-43	0	7515
TUBD1	2.89E-43	0	7516
CNOT10	2.87E-43	0	7517
TRAPPC11	2.84E-43	0	7518
NUDT9	2.62E-43	0	7519
TUSC1	2.42E-43	0	7520

TTC26	2.28E-43	0	7521
UNC80	2.28E-43	0	7522
TMEM182	2.26E-43	0	7523
GTPBP10	2.23E-43	0	7524
SPATA22	2.17E-43	0	7525
CDY1B	1.84E-43	0	7526
CABP7	1.65E-43	0	7527
HHLA2	1.58E-43	0	7528
RP29	1.42E-43	0	7529
IGHV1-69	1.40E-43	0	7530
PRTFDC1	1.37E-43	0	7531
IZUMO2	1.14E-43	0	7532
FBLN7	1.14E-43	0	7533
ZC3HC1	1.00E-43	0	7534
C2CD2L	8.30E-44	0	7535
OA4	8.10E-44	0	7536
TTLL8	7.60E-44	0	7537
OPA5	6.70E-44	0	7538
ACTBP8	3.10E-44	0	7539
RAVER1	2.70E-44	0	7540
ECEL1	2.10E-44	0	7541
COPD3	1.80E-44	0	7542
WDR72	8.40E-45	0	7543
TUBG1	5.60E-45	0	7544
CAPN9	-2.80E-45	0	7545
DDX43	-4.20E-45	0	7546
PTOS2	-8.40E-45	0	7547
FASTKD3	-2.10E-44	0	7548
SUMO1P1	-6.70E-44	0	7549
DFNA16	-8.70E-44	0	7550
RTTN	-9.10E-44	0	7551
TUBGCP3	-9.20E-44	0	7552
PCNAP3	-1.04E-43	0	7553
ANO7	-1.06E-43	0	7554
PKD2L2	-1.10E-43	0	7555
TTC19	-1.26E-43	0	7556
ACSM2B	-1.39E-43	0	7557
MYH13	-1.49E-43	0	7558
FN3K	-2.30E-43	0	7559
PAND1	-2.31E-43	0	7560
TXNDC17	-2.35E-43	0	7561
KIF12	-2.56E-43	0	7562
DEL3PTERP2	-2.59E-43	0	7563

OLFML2A	-2.59E-43	0	7564
GPBP1	-2.68E-43	0	7565
DFNB26	-3.20E-43	0	7566
DPY19L2P1	-3.32E-43	0	7567
MGR2	-3.64E-43	0	7568
NUDT4	-3.70E-43	0	7569
SCZD10	-3.83E-43	0	7570
TPT1P1	-3.84E-43	0	7571
TRBV6-1	-3.92E-43	0	7572
HCG22	-4.02E-43	0	7573
MIR3162	-4.08E-43	0	7574
BRD7P3	-4.10E-43	0	7575
ZNF18	-4.30E-43	0	7576
ATP6V1D	-4.39E-43	0	7577
ARRDC4	-4.51E-43	0	7578
BRINP1	-4.57E-43	0	7579
SMPDL3A	-4.70E-43	0	7580
DYT4	-4.71E-43	0	7581
AGBL5	-4.89E-43	0	7582
LPPR5	-4.89E-43	0	7583
EDS8	-4.95E-43	0	7584
ALDH1L2	-5.09E-43	0	7585
ANKRD54	-5.16E-43	0	7586
TRAPPC6B	-5.68E-43	0	7587
LOXHD1	-5.69E-43	0	7588
CYHR1	-5.97E-43	0	7589
SNAPC1	-6.17E-43	0	7590
RNF170	-6.25E-43	0	7591
ZCWPW1	-6.30E-43	0	7592
MFAP1	-6.64E-43	0	7593
AP2S1	-6.73E-43	0	7594
GLM7	-6.84E-43	0	7595
ELMOD3	-6.94E-43	0	7596
MIR1193	-7.09E-43	0	7597
MTFP1	-7.60E-43	0	7598
TAS2R31	-7.74E-43	0	7599
FAM43B	-7.79E-43	0	7600
IYD	-7.86E-43	0	7601
PYGL	-8.04E-43	0	7602
CHPF2	-8.20E-43	0	7603
PKD1L2	-8.86E-43	0	7604
PUS1	-9.07E-43	0	7605
GABRR2	-9.11E-43	0	7606

WDR34	-9.42E-43	0	7607
DUS2	-9.68E-43	0	7608
GLULP1	-9.90E-43	0	7609
ZNF10	-1.12E-42	0	7610
PRDM12	-1.15E-42	0	7611
PSG6	-1.20E-42	0	7612
SLC35B1	-1.24E-42	0	7613
TRNAL2	-1.30E-42	0	7614
HDDC3	-1.31E-42	0	7615
TRAV1-2	-1.38E-42	0	7616
C7ORF13	-1.38E-42	0	7617
DEFB126	-1.44E-42	0	7618
PLGLB1	-1.48E-42	0	7619
DEL18P	-1.49E-42	0	7620
ZNF106	-1.56E-42	0	7621
GLC1I	-1.67E-42	0	7622
SRGAP2C	-4.78E-38	0	7623
SRGAP2D	-4.78E-38	0	7624
ATP5I	-6.52E-38	0	7625
ASB17	-6.86E-38	0	7626
ASB9	-6.86E-38	0	7627
GPR21	-9.09E-38	0	7628
GPR22	-9.09E-38	0	7629
AK7	-1.09E-37	0	7630
MIR4707	-1.74E-37	0	7631
MIR4767	-1.74E-37	0	7632
MIR597	-1.91E-37	0	7633
SNORD13	-1.91E-37	0	7634
DFNB59	-2.46E-08	0	7635
MT-TC	-2.59E-08	0	7636
CHST15	-2.68E-08	0	7637
PECR	-9.78E-08	0	7638
PRSS23	-9.78E-08	0	7639
ZNF365	-9.78E-08	0	7640
PLEKHM2	-2.42E-07	0	7641
RBMXL2	-3.73E-07	0	7642
CDY1	-4.78E-07	0	7643
UBTD2	-5.42E-07	0	7644
CHIC1	-6.68E-07	0	7645
PGM2L1	-7.13E-07	0	7646
MUC3A	-9.42E-07	0	7647
PSTK	-9.69E-07	0	7648
SPG43	-1.02E-06	0	7649

TIMM10	-1.04E-06	0	7650
PHACTR2	-1.16E-06	0	7651
MIR876	-1.27E-06	0	7652
TEX12	-1.28E-06	0	7653
GAL3ST4	-1.35E-06	0	7654
TAF2	-1.40E-06	0	7655
FITM1	-1.50E-06	0	7656
PIGX	-1.53E-06	0	7657
MUC3B	-1.63E-06	0	7658
HSD11B1L	-1.64E-06	0	7659
TRAJ12	-1.71E-06	0	7660
TRAJ20	-1.71E-06	0	7661
TRAJ33	-1.71E-06	0	7662
ALOX15B	-1.84E-06	0	7663
TKTL2	-2.26E-06	0	7664
USP29	-2.32E-06	0	7665
MKRN2	-2.33E-06	0	7666
PLXDC2	-2.44E-06	0	7667
MIR3667	-2.63E-06	0	7668
SNORD25	-2.70E-06	0	7669
ARV1	-2.75E-06	0	7670
LHFPL5	-2.83E-06	0	7671
TMIE	-2.83E-06	0	7672
PKHD1L1	-3.03E-06	0	7673
ELOVL4	-3.15E-06	0	7674
CTDSP1	-3.16E-06	0	7675
DLG5	-3.35E-06	0	7676
C1ORF64	-3.38E-06	0	7677
IMPA2	-3.61E-06	0	7678
CHED1	-3.63E-06	0	7679
WNT10B	-3.67E-06	0	7680
GLOD4	-3.67E-06	0	7681
KCND1	-3.70E-06	0	7682
MCTS2P	-3.70E-06	0	7683
MT-RNR2	-3.80E-06	0	7684
COMMD3	-3.82E-06	0	7685
CHST6	-3.96E-06	0	7686
C11ORF95	-4.04E-06	0	7687
TSSK1A	-4.09E-06	0	7688
NME5	-4.15E-06	0	7689
USP47	-4.47E-06	0	7690
FOXD4	-4.58E-06	0	7691
IS4	-4.92E-06	0	7692

MPPE1	-4.99E-06	0	7693
NOL8	-5.11E-06	0	7694
CST7	-5.29E-06	0	7695
IGKJ@	-5.88E-06	0	7696
FAM120A	-6.08E-06	0	7697
ARL17A	-6.13E-06	0	7698
ARL17B	-6.13E-06	0	7699
ZNF80	-6.56E-06	0	7700
PJVK	-6.62E-06	0	7701
RP1L1	-6.77E-06	0	7702
OAS2	-6.79E-06	0	7703
LRRC52	-6.85E-06	0	7704
LRRC55	-6.85E-06	0	7705
MAP6D1	-6.88E-06	0	7706
GEMIN7	-7.07E-06	0	7707
SEMA4G	-7.20E-06	0	7708
C8A	-7.31E-06	0	7709
PEX26	-7.43E-06	0	7710
STIM2	-7.52E-06	0	7711
FKBP11	-7.82E-06	0	7712
IFITM10	-7.82E-06	0	7713
CCT7	-8.06E-06	0	7714
TSSK3	-8.14E-06	0	7715
TSSK2	-8.21E-06	0	7716
SDR16C5	-8.44E-06	0	7717
SLC1A7	-8.63E-06	0	7718
CCBL2	-8.65E-06	0	7719
TLX2	-8.66E-06	0	7720
AD8	-8.70E-06	0	7721
CDC40	-8.72E-06	0	7722
CEACAMP11	-8.81E-06	0	7723
DGCR6	-8.93E-06	0	7724
MIR491	-8.94E-06	0	7725
MIR18B	-8.97E-06	0	7726
SLBP	-9.00E-06	0	7727
SLC39A12	-9.51E-06	0	7728
CCSER1	-9.72E-06	0	7729
RSPH9	-1.00E-05	0	7730
MT-ATP8	-1.01E-05	0	7731
ZNF101	-1.06E-05	0	7732
NXPH3	-1.06E-05	0	7733
MYLIP	-1.07E-05	0	7734
EIF4G3	-1.09E-05	0	7735

LRRC8A	-1.11E-05	0	7736
PDIA4	-1.15E-05	0	7737
UGT2A2	-1.15E-05	0	7738
DNAAF2	-1.16E-05	0	7739
IGHD1-14	-1.18E-05	0	7740
PIGV	-1.23E-05	0	7741
ANKS4B	-1.23E-05	0	7742
TBCK	-1.26E-05	0	7743
SNX31	-1.28E-05	0	7744
SLC5A10	-1.30E-05	0	7745
CWC25	-1.30E-05	0	7746
USP39	-1.30E-05	0	7747
ELP5	-1.31E-05	0	7748
FDX1L	-1.34E-05	0	7749
FRA10D	-1.37E-05	0	7750
FRA2F	-1.37E-05	0	7751
FRA7I	-1.37E-05	0	7752
FRAXC	-1.37E-05	0	7753
CHMP7	-1.37E-05	0	7754
TMIGD2	-1.38E-05	0	7755
PMPCB	-1.38E-05	0	7756
MRPL40	-1.39E-05	0	7757
BBS12	-1.39E-05	0	7758
SNRPA1	-1.39E-05	0	7759
ZDHHC8	-1.39E-05	0	7760
XRCC2	-1.40E-05	0	7761
FBXL19	-1.41E-05	0	7762
KDF1	-1.44E-05	0	7763
MPDU1	-1.47E-05	0	7764
E11S	-1.53E-05	0	7765
AGBL3	-1.54E-05	0	7766
HP1BP3	-1.57E-05	0	7767
DHX38	-1.58E-05	0	7768
PITPNB	-1.60E-05	0	7769
HABP2	-1.61E-05	0	7770
TNRC6B	-1.62E-05	0	7771
SLC19A3	-1.62E-05	0	7772
AADAT	-1.65E-05	0	7773
SRP9	-1.73E-05	0	7774
COA7	-1.74E-05	0	7775
RGPD2	-1.75E-05	0	7776
NUDT3	-1.76E-05	0	7777
WARS2	-1.78E-05	0	7778

VPS26A	-1.81E-05	0	7779
LOC643714	-1.82E-05	0	7780
MRPS30	-1.82E-05	0	7781
EVX2	-1.83E-05	0	7782
BIRC6-AS1	-1.85E-05	0	7783
LANCL2	-1.85E-05	0	7784
ROPN1L	-1.87E-05	0	7785
CMAHP	-1.88E-05	0	7786
AIS2	-1.88E-05	0	7787
CMYA5	-1.88E-05	0	7788
SNORD54	-1.88E-05	0	7789
MED18	-1.89E-05	0	7790
NOMO3	-1.91E-05	0	7791
PRR5	-1.96E-05	0	7792
PIGA	-1.98E-05	0	7793
MT-TY	-1.98E-05	0	7794
TGM3	-1.99E-05	0	7795
MAB21L2	-2.00E-05	0	7796
ULK4	-2.02E-05	0	7797
PITPNM2	-2.02E-05	0	7798
CNTN3	-2.04E-05	0	7799
PTPRM	-2.04E-05	0	7800
LARP7	-2.06E-05	0	7801
MYO1D	-2.10E-05	0	7802
ZFP37	-2.10E-05	0	7803
GLRX2	-2.13E-05	0	7804
SNORD30	-2.22E-05	0	7805
SCA32	-2.23E-05	0	7806
RCN2	-2.24E-05	0	7807
ST6GALNAC5	-2.25E-05	0	7808
GPR6	-2.26E-05	0	7809
DNAAF3	-2.26E-05	0	7810
BCL2L12	-2.28E-05	0	7811
INA	-2.28E-05	0	7812
FXYD6P3	-2.33E-05	0	7813
IBD6	-2.34E-05	0	7814
BPHL	-2.37E-05	0	7815
DLGAP1	-2.41E-05	0	7816
CDH10	-2.41E-05	0	7817
ZC3H14	-2.46E-05	0	7818
KNTC1	-2.46E-05	0	7819
MUC12	-2.47E-05	0	7820
RPL31	-2.49E-05	0	7821

PYGB	-2.52E-05	0	7822
KCNV2	-2.54E-05	0	7823
MT-ND2	-2.55E-05	0	7824
COPZ1	-2.56E-05	0	7825
SLC23A1	-2.59E-05	0	7826
CDA	-2.59E-05	0	7827
CRELD1	-2.59E-05	0	7828
ARMCX2	-2.63E-05	0	7829
RCC2	-2.63E-05	0	7830
VARS	-2.63E-05	0	7831
CAPRIN2	-2.64E-05	0	7832
CNPY1	-2.64E-05	0	7833
FOXI1	-2.64E-05	0	7834
RNASE13	-2.64E-05	0	7835
CHST12	-2.65E-05	0	7836
CNIH2	-2.66E-05	0	7837
AAA2	-2.68E-05	0	7838
RPS8	-2.74E-05	0	7839
BEST2	-2.76E-05	0	7840
LMOD3	-2.76E-05	0	7841
SNORD73A	-2.77E-05	0	7842
CYCSP53	-2.77E-05	0	7843
F8A1	-2.78E-05	0	7844
ABHD12	-2.78E-05	0	7845
LSG1	-2.80E-05	0	7846
TMEM165	-2.83E-05	0	7847
HERPUD1	-2.84E-05	0	7848
CNGA4	-2.86E-05	0	7849
TCTN1	-2.89E-05	0	7850
COX16	-2.91E-05	0	7851
CPA2	-2.91E-05	0	7852
MID1IP1	-2.96E-05	0	7853
APM2	-2.96E-05	0	7854
OSBPL1A	-2.97E-05	0	7855
GK2	-2.99E-05	0	7856
SPINK4	-3.04E-05	0	7857
MIR378B	-3.13E-05	0	7858
CENPN	-3.16E-05	0	7859
NAT14	-3.16E-05	0	7860
NME4	-3.23E-05	0	7861
SSX3	-3.27E-05	0	7862
PLEKHM3	-3.28E-05	0	7863
BCL7A	-3.29E-05	0	7864

GIMAP6	-3.30E-05	0	7865
POMT2	-3.30E-05	0	7866
SIT1	-3.30E-05	0	7867
MYO7B	-3.36E-05	0	7868
KCMF1	-3.38E-05	0	7869
PHC3	-3.38E-05	0	7870
PKD1L1	-3.41E-05	0	7871
CEND1	-3.42E-05	0	7872
TEX28	-3.44E-05	0	7873
CLDN15	-3.46E-05	0	7874
SLC24A1	-3.51E-05	0	7875
BAI3	-3.51E-05	0	7876
C1QL4	-3.51E-05	0	7877
EIF2C4	-3.52E-05	0	7878
MIR1244-1	-3.54E-05	0	7879
NKS1	-3.54E-05	0	7880
NRXN2	-3.60E-05	0	7881
SCAX1	-3.61E-05	0	7882
MED13L	-3.63E-05	0	7883
DOM3Z	-3.63E-05	0	7884
RNF24	-3.64E-05	0	7885
TST2	-3.65E-05	0	7886
PAPOLB	-3.66E-05	0	7887
ERP29	-3.72E-05	0	7888
SOCS6	-3.74E-05	0	7889
MT-ND5	-3.77E-05	0	7890
FREM2	-3.77E-05	0	7891
MMRN2	-3.81E-05	0	7892
MIR2861	-3.81E-05	0	7893
ELOVL1	-3.83E-05	0	7894
SLC26A8	-3.86E-05	0	7895
OPA1	-3.91E-05	0	7896
PAQR5	-3.96E-05	0	7897
NATP	-3.96E-05	0	7898
ARL13A	-3.99E-05	0	7899
GABRA5	-4.00E-05	0	7900
PCBD2	-4.00E-05	0	7901
BTNL3	-4.01E-05	0	7902
GCC2	-4.02E-05	0	7903
ABHD14A	-4.03E-05	0	7904
ZIC4	-4.03E-05	0	7905
MIR525	-4.04E-05	0	7906
TPD52L1	-4.05E-05	0	7907

WT3	-4.06E-05	0	7908
TCEA2	-4.06E-05	0	7909
HHIPL2	-4.07E-05	0	7910
OXSR1	-4.09E-05	0	7911
SRGAP1	-4.09E-05	0	7912
NUTM2A	-4.11E-05	0	7913
NKX6-2	-4.12E-05	0	7914
GALE	-4.13E-05	0	7915
CAPN7	-4.14E-05	0	7916
SKAP	-4.15E-05	0	7917
ZIC3	-4.17E-05	0	7918
ST6GALNAC3	-4.18E-05	0	7919
MIR1269B	-4.23E-05	0	7920
RAB30	-4.23E-05	0	7921
SNORD35A	-4.23E-05	0	7922
SPAG6	-4.24E-05	0	7923
USP24	-4.41E-05	0	7924
CHDH	-4.43E-05	0	7925
CCDC39	-4.44E-05	0	7926
RFX6	-4.46E-05	0	7927
IMMP2L	-4.50E-05	0	7928
COA3	-4.56E-05	0	7929
TMEM237	-4.56E-05	0	7930
LY6G6F	-4.61E-05	0	7931
DCAF11	-4.62E-05	0	7932
MAK16	-4.62E-05	0	7933
PDZD11	-4.64E-05	0	7934
SNORA70	-4.64E-05	0	7935
KLHDC3	-4.68E-05	0	7936
DNAH5	-4.68E-05	0	7937
KIAA0196	-4.71E-05	0	7938
CPNE2	-4.73E-05	0	7939
CPNE6	-4.73E-05	0	7940
CPNE7	-4.73E-05	0	7941
MAP7D3	-4.74E-05	0	7942
RNF152	-4.77E-05	0	7943
CDC20B	-4.79E-05	0	7944
TJAP1	-4.82E-05	0	7945
LPRS1	-4.87E-05	0	7946
STAU2	-4.90E-05	0	7947
JSRP1	-4.94E-05	0	7948
SEMA4B	-4.98E-05	0	7949
RBM22	-5.03E-05	0	7950

RTP1	-5.08E-05	0	7951
KLRF2	-5.10E-05	0	7952
SCN2B	-5.20E-05	0	7953
ZNF644	-5.21E-05	0	7954
RSPH3	-5.23E-05	0	7955
DPP10	-5.23E-05	0	7956
SLC34A2	-5.25E-05	0	7957
BCMO1	-5.35E-05	0	7958
CEMIP	-5.36E-05	0	7959
EPHA6	-5.41E-05	0	7960
GABRB2	-5.50E-05	0	7961
PYCR2	-5.53E-05	0	7962
EBF3	-5.55E-05	0	7963
NCBP2	-5.55E-05	0	7964
CABYR	-5.57E-05	0	7965
RLF	-5.59E-05	0	7966
TFAP2E	-5.62E-05	0	7967
KCTD18	-5.63E-05	0	7968
HSPH1	-5.65E-05	0	7969
ATP10A	-5.66E-05	0	7970
LGI4	-5.67E-05	0	7971
UBQLN4	-5.68E-05	0	7972
ANKRD2	-5.69E-05	0	7973
CMPK1	-5.69E-05	0	7974
CLGN	-5.70E-05	0	7975
CPPED1	-5.70E-05	0	7976
MIER2	-5.70E-05	0	7977
NFU1	-5.71E-05	0	7978
PDS5B	-5.76E-05	0	7979
OA2	-5.84E-05	0	7980
GEMIN5	-5.90E-05	0	7981
PLEKHM1	-5.99E-05	0	7982
SPICE1	-6.04E-05	0	7983
ZNHIT2	-6.08E-05	0	7984
MICAL3	-6.11E-05	0	7985
BCO2	-6.15E-05	0	7986
LGMD1G	-6.16E-05	0	7987
LGMD1H	-6.16E-05	0	7988
ISL2	-6.17E-05	0	7989
MIR196A2	-6.17E-05	0	7990
LMAN2L	-6.19E-05	0	7991
STX5	-6.20E-05	0	7992
C1QL2	-6.25E-05	0	7993

PMS2P4	-6.32E-05	0	7994
NXPH4	-6.35E-05	0	7995
TFP1	-6.46E-05	0	7996
SRPR	-6.54E-05	0	7997
CAMK2A	-6.55E-05	0	7998
CFAP54	-6.59E-05	0	7999
SLC16A12	-6.62E-05	0	8000
BTD	-6.64E-05	0	8001
SLC39A7	-6.69E-05	0	8002
ACADVL	-6.71E-05	0	8003
LRRTM2	-6.72E-05	0	8004
AFMID	-6.73E-05	0	8005
EIF1AY	-6.79E-05	0	8006
RASSF4	-6.80E-05	0	8007
CMC1	-6.82E-05	0	8008
UBE2G1	-6.89E-05	0	8009
B4GALT3	-6.98E-05	0	8010
SGPP1	-6.98E-05	0	8011
ZBTB10	-6.98E-05	0	8012
ASTN1	-7.00E-05	0	8013
MCM3AP	-7.01E-05	0	8014
ASB7	-7.01E-05	0	8015
FBXO44	-7.03E-05	0	8016
SPATA5	-7.04E-05	0	8017
NUDT10	-7.05E-05	0	8018
CCNI	-7.06E-05	0	8019
TDRD12	-7.18E-05	0	8020
EXT1	-7.21E-05	0	8021
TMEM127	-7.22E-05	0	8022
GGNBP1	-7.29E-05	0	8023
PCP4L1	-7.37E-05	0	8024
SMARCB1	-7.52E-05	0	8025
MBS2	-7.55E-05	0	8026
KLC1	-7.56E-05	0	8027
CRYM	-7.57E-05	0	8028
EIF1AX	-7.59E-05	0	8029
IMPDH2	-7.62E-05	0	8030
MUC19	-7.65E-05	0	8031
OXGR1	-7.67E-05	0	8032
CCDC7	-7.67E-05	0	8033
AMELY	-7.68E-05	0	8034
TTBK2	-7.71E-05	0	8035
LGALS13	-7.74E-05	0	8036

BRAP	-7.76E-05	0	8037
MIR591	-7.76E-05	0	8038
ZNF384	-7.81E-05	0	8039
COLEC10	-7.85E-05	0	8040
RIC1	-7.87E-05	0	8041
MIOX	-7.93E-05	0	8042
SMR3A	-7.93E-05	0	8043
ANKRD37	-8.00E-05	0	8044
NGFRAP1	-8.03E-05	0	8045
SF3A1	-8.04E-05	0	8046
HTRA4	-8.10E-05	0	8047
FAT2	-8.11E-05	0	8048
MIR548B	-8.16E-05	0	8049
SLC6A12	-8.19E-05	0	8050
DGKZ	-8.27E-05	0	8051
PRPS1	-8.30E-05	0	8052
PEX6	-8.36E-05	0	8053
DHCR7	-8.37E-05	0	8054
SNORA23	-8.38E-05	0	8055
SPAG1	-8.44E-05	0	8056
OTC	-8.44E-05	0	8057
PLEKHB1	-8.46E-05	0	8058
ING5	-8.47E-05	0	8059
C22ORF29	-8.47E-05	0	8060
SREK1	-8.49E-05	0	8061
MEMO1	-8.49E-05	0	8062
NRG3	-8.50E-05	0	8063
SMU1	-8.56E-05	0	8064
SCARNA2	-8.59E-05	0	8065
RHBDL3	-8.59E-05	0	8066
VPS33B	-8.60E-05	0	8067
SEMG2	-8.60E-05	0	8068
AJAP1	-8.63E-05	0	8069
CDH16	-8.66E-05	0	8070
MRPL1	-8.76E-05	0	8071
KIFC1	-8.83E-05	0	8072
HCAR3	-8.86E-05	0	8073
TAS1R3	-8.86E-05	0	8074
MAGEA9	-8.93E-05	0	8075
GPR137	-8.96E-05	0	8076
RPAP2	-9.01E-05	0	8077
GPANK1	-9.03E-05	0	8078
MIR543	-9.04E-05	0	8079

PACRG	-9.05E-05	0	8080
TOR1B	-9.05E-05	0	8081
TUBB2A	-9.05E-05	0	8082
MAGEB1	-9.21E-05	0	8083
SPACA5	-9.22E-05	0	8084
MIR610	-9.27E-05	0	8085
COPS3	-9.27E-05	0	8086
AGPHD1	-9.31E-05	0	8087
FAM3D	-9.33E-05	0	8088
CHST1	-9.34E-05	0	8089
GPR4	-9.36E-05	0	8090
NFKBIL1	-9.37E-05	0	8091
TBX3	-9.38E-05	0	8092
RNF139	-9.44E-05	0	8093
MTX3	-9.45E-05	0	8094
MED22	-9.46E-05	0	8095
RNASEH1	-9.53E-05	0	8096
LMO7	-9.54E-05	0	8097
VPS33A	-9.58E-05	0	8098
METAP2	-9.59E-05	0	8099
FFAR4	-9.59E-05	0	8100
NOP56	-9.62E-05	0	8101
OTSC1	-9.62E-05	0	8102
FAM206A	-9.63E-05	0	8103
DUX4L9	-9.66E-05	0	8104
MSL1	-9.67E-05	0	8105
RORB	-9.67E-05	0	8106
THUMPD2	-9.68E-05	0	8107
GAPDHS	-9.75E-05	0	8108
PEPC	-9.79E-05	0	8109
UNC79	-9.79E-05	0	8110
TRGC2	-9.84E-05	0	8111
DEFB106A	-9.94E-05	0	8112
SNORD72	-9.95E-05	0	8113
KCNK15	-9.98E-05	0	8114
PAPA3	-1.00E-04	0	8115
KCTD13	-1.00E-04	0	8116
COPR5	-1.00E-04	0	8117
RCBTB2	-1.00E-04	0	8118
CNOT4	-1.01E-04	0	8119
CDK10	-1.02E-04	0	8120
HIST1H1A	-1.02E-04	0	8121
MIR1908	-1.02E-04	0	8122

MIR3131	-1.02E-04	0	8123
BW16	-1.02E-04	0	8124
GRHPR	-1.02E-04	0	8125
RFT1	-1.02E-04	0	8126
ETFDH	-1.02E-04	0	8127
SNORD41	-1.02E-04	0	8128
RBM3	-1.03E-04	0	8129
HELT	-1.03E-04	0	8130
DLGAP3	-1.04E-04	0	8131
ZNF639	-1.04E-04	0	8132
ZCCHC7	-1.05E-04	0	8133
HRC	-1.05E-04	0	8134
ANO4	-1.05E-04	0	8135
OR6A2	-1.05E-04	0	8136
SYNE3	-1.06E-04	0	8137
SOCS4	-1.06E-04	0	8138
CTU1	-1.06E-04	0	8139
SLFN5	-1.07E-04	0	8140
TTYH1	-1.07E-04	0	8141
TTYH2	-1.07E-04	0	8142
TTYH3	-1.07E-04	0	8143
MAGEC1	-1.08E-04	0	8144
ITIH5	-1.08E-04	0	8145
SLMAP	-1.08E-04	0	8146
DDO	-1.08E-04	0	8147
MPD3	-1.09E-04	0	8148
ZFPL1	-1.09E-04	0	8149
CENPT	-1.09E-04	0	8150
TBR1	-1.09E-04	0	8151
CRCT1	-1.10E-04	0	8152
AMER3	-1.10E-04	0	8153
SCN4B	-1.10E-04	0	8154
LFNG	-1.11E-04	0	8155
BRCA3	-1.11E-04	0	8156
RPSAP14	-1.11E-04	0	8157
HPV18I2	-1.12E-04	0	8158
RABL2B	-1.12E-04	0	8159
IGSF6	-1.13E-04	0	8160
PPP2R5D	-1.13E-04	0	8161
BARHL1	-1.14E-04	0	8162
C2ORF40	-1.14E-04	0	8163
SAR1B	-1.14E-04	0	8164
RNASEH2A	-1.15E-04	0	8165

PRKAR1A	-1.15E-04	0	8166
PREPL	-1.15E-04	0	8167
PLA2G2F	-1.15E-04	0	8168
GRINA	-1.15E-04	0	8169
VPS13A	-1.16E-04	0	8170
KDM2B	-1.16E-04	0	8171
RMI2	-1.16E-04	0	8172
BPGM	-1.17E-04	0	8173
MIR644A	-1.17E-04	0	8174
ARMC4	-1.17E-04	0	8175
MPP7	-1.17E-04	0	8176
FAF2	-1.18E-04	0	8177
GAMT	-1.18E-04	0	8178
RNF144B	-1.19E-04	0	8179
COQ9	-1.19E-04	0	8180
CHCHD6	-1.19E-04	0	8181
ATP5G2	-1.19E-04	0	8182
GPR98	-1.19E-04	0	8183
IMMP1L	-1.19E-04	0	8184
RPAP3	-1.19E-04	0	8185
MGAT4A	-1.20E-04	0	8186
UBR4	-1.21E-04	0	8187
ATP2C2	-1.21E-04	0	8188
SLCO3A1	-1.21E-04	0	8189
DYX3	-1.21E-04	0	8190
DFNB66	-1.21E-04	0	8191
MRPL19	-1.21E-04	0	8192
TRPC7	-1.21E-04	0	8193
ANKRD42	-1.21E-04	0	8194
RTKN2	-1.22E-04	0	8195
BIN3	-1.23E-04	0	8196
CAMSAP1	-1.23E-04	0	8197
SETD5	-1.23E-04	0	8198
CLIC5	-1.23E-04	0	8199
ST3GAL6	-1.24E-04	0	8200
PCSK4	-1.25E-04	0	8201
PEX12	-1.25E-04	0	8202
ARHGEF17	-1.25E-04	0	8203
CSTF1	-1.25E-04	0	8204
PLA2G4C	-1.26E-04	0	8205
NPM2	-1.26E-04	0	8206
ADAMTS20	-1.26E-04	0	8207
GON4L	-1.27E-04	0	8208

GSPT2	-1.27E-04	0	8209
CRYBA1	-1.29E-04	0	8210
CPEB2	-1.29E-04	0	8211
SLC25A38	-1.30E-04	0	8212
ICMT	-1.30E-04	0	8213
LGALS14	-1.31E-04	0	8214
RABGAP1L	-1.31E-04	0	8215
EDEM2	-1.31E-04	0	8216
SSAV1	-1.31E-04	0	8217
ESX1	-1.32E-04	0	8218
CADM4	-1.33E-04	0	8219
HUS1B	-1.33E-04	0	8220
NDUFB5	-1.33E-04	0	8221
TXNRD3NB	-1.33E-04	0	8222
LACTB	-1.34E-04	0	8223
BICC1	-1.34E-04	0	8224
CBX8	-1.34E-04	0	8225
KRT74	-1.34E-04	0	8226
TBC1D10B	-1.35E-04	0	8227
ZFYVE26	-1.35E-04	0	8228
SPAG16	-1.36E-04	0	8229
GAN	-1.37E-04	0	8230
TBCB	-1.37E-04	0	8231
CEP78	-1.37E-04	0	8232
RMND5B	-1.37E-04	0	8233
GORAB	-1.38E-04	0	8234
PDE11A	-1.38E-04	0	8235
DCHS1	-1.38E-04	0	8236
FJX1	-1.38E-04	0	8237
MBD3L1	-1.38E-04	0	8238
10-Sep	-1.38E-04	0	8239
FAM65B	-1.38E-04	0	8240
TMEM50A	-1.38E-04	0	8241
ADAMTS17	-1.39E-04	0	8242
ADAMTSL2	-1.39E-04	0	8243
GPR42	-1.39E-04	0	8244
PRSS56	-1.39E-04	0	8245
MKX	-1.40E-04	0	8246
GJB4	-1.40E-04	0	8247
UTP18	-1.40E-04	0	8248
FARP1	-1.41E-04	0	8249
DAPK2	-1.41E-04	0	8250
GALK1	-1.43E-04	0	8251

KCNK12	-1.43E-04	0	8252
DCTN3	-1.43E-04	0	8253
C1RL	-1.44E-04	0	8254
FOLR1P1	-1.44E-04	0	8255
PRSS36	-1.44E-04	0	8256
SPINK9	-1.44E-04	0	8257
PYHIN1	-1.44E-04	0	8258
ZDHHC13	-1.45E-04	0	8259
SPANXC	-1.46E-04	0	8260
SYT11	-1.47E-04	0	8261
NEB	-1.47E-04	0	8262
APBB2	-1.47E-04	0	8263
PLA2R1	-1.48E-04	0	8264
RPS25	-1.49E-04	0	8265
EXT3	-1.49E-04	0	8266
SOX12	-1.51E-04	0	8267
AACP	-1.51E-04	0	8268
TEAD3	-1.51E-04	0	8269
COA6	-1.51E-04	0	8270
TANC2	-1.51E-04	0	8271
LGALS12	-1.52E-04	0	8272
CRISP2	-1.52E-04	0	8273
GGT2	-1.53E-04	0	8274
MMD2	-1.53E-04	0	8275
TRIM16L	-1.54E-04	0	8276
VPS16	-1.54E-04	0	8277
PHF19	-1.54E-04	0	8278
VIS1	-1.54E-04	0	8279
HEMK1	-1.54E-04	0	8280
CLDN16	-1.55E-04	0	8281
CEP97	-1.55E-04	0	8282
EID2	-1.55E-04	0	8283
NDUFS7	-1.55E-04	0	8284
CHST8	-1.56E-04	0	8285
LINGO4	-1.56E-04	0	8286
MIR599	-1.56E-04	0	8287
DENND2D	-1.56E-04	0	8288
RRAGD	-1.56E-04	0	8289
SEC61A1	-1.57E-04	0	8290
MURC	-1.58E-04	0	8291
TDRKH	-1.58E-04	0	8292
MTPAP	-1.59E-04	0	8293
PPDPF	-1.59E-04	0	8294

SNX8	-1.60E-04	0	8295
HERC1	-1.60E-04	0	8296
KRT76	-1.61E-04	0	8297
FMO1	-1.61E-04	0	8298
PSME4	-1.61E-04	0	8299
COX17P1	-1.62E-04	0	8300
MFSD11	-1.62E-04	0	8301
NDUFA4	-1.62E-04	0	8302
UQCRQ	-1.62E-04	0	8303
GLUD2	-1.62E-04	0	8304
SNORD16	-1.63E-04	0	8305
RDBP	-1.63E-04	0	8306
RRAGC	-1.64E-04	0	8307
BPIFB1	-1.64E-04	0	8308
CADPS	-1.64E-04	0	8309
ORAI3	-1.64E-04	0	8310
ADPRM	-1.66E-04	0	8311
DNAJC24	-1.66E-04	0	8312
ZNF83	-1.66E-04	0	8313
TOMM7	-1.66E-04	0	8314
ALDH1A3	-1.66E-04	0	8315
AASDH	-1.67E-04	0	8316
COX18	-1.67E-04	0	8317
VAMP1	-1.67E-04	0	8318
LAIR2	-1.68E-04	0	8319
METTL14	-1.69E-04	0	8320
OIP5	-1.70E-04	0	8321
GOLIM4	-1.70E-04	0	8322
SEPN1	-1.70E-04	0	8323
CLRN1	-1.70E-04	0	8324
DAZ4	-1.71E-04	0	8325
SLC2A9	-1.71E-04	0	8326
VRK3	-1.71E-04	0	8327
SOX14	-1.72E-04	0	8328
MSRB1	-1.72E-04	0	8329
MYO3B	-1.73E-04	0	8330
CELA3A	-1.74E-04	0	8331
MIR552	-1.74E-04	0	8332
ISM2	-1.74E-04	0	8333
CHD9	-1.74E-04	0	8334
POU6F1	-1.74E-04	0	8335
BW60	-1.75E-04	0	8336
MCFD2	-1.75E-04	0	8337

GINS1	-1.76E-04	0	8338
DNPEP	-1.78E-04	0	8339
ATRAID	-1.78E-04	0	8340
EPS15L1	-1.78E-04	0	8341
SLC25A41	-1.79E-04	0	8342
NMBR	-1.79E-04	0	8343
SEPHS2	-1.79E-04	0	8344
RLTPR	-1.79E-04	0	8345
BW6	-1.80E-04	0	8346
STX17	-1.81E-04	0	8347
MIR135B	-1.81E-04	0	8348
SPATA19	-1.81E-04	0	8349
FMO2	-1.81E-04	0	8350
TPRN	-1.81E-04	0	8351
MKL1	-1.82E-04	0	8352
NECAB3	-1.82E-04	0	8353
AK2P1	-1.82E-04	0	8354
TARS	-1.83E-04	0	8355
EBAG9	-1.83E-04	0	8356
NUDT5	-1.83E-04	0	8357
UBXN1	-1.83E-04	0	8358
TOB2	-1.83E-04	0	8359
GPC6	-1.83E-04	0	8360
POLRMT	-1.83E-04	0	8361
RDM1	-1.84E-04	0	8362
ADPRH	-1.84E-04	0	8363
PPAP2B	-1.84E-04	0	8364
LDHB	-1.85E-04	0	8365
PRSS21	-1.86E-04	0	8366
SRR	-1.87E-04	0	8367
DYNC2LI1	-1.87E-04	0	8368
SNAP91	-1.87E-04	0	8369
NETO1	-1.87E-04	0	8370
ZRSR1	-1.87E-04	0	8371
MBL2	-1.88E-04	0	8372
FAU	-1.88E-04	0	8373
OR3A1	-1.88E-04	0	8374
SNX14	-1.88E-04	0	8375
C11ORF83	-1.88E-04	0	8376
ZYG11B	-1.88E-04	0	8377
CABP4	-1.89E-04	0	8378
SAMM50	-1.89E-04	0	8379
KCNIP1	-1.89E-04	0	8380

STRN3	-1.89E-04	0	8381
AP1B1	-1.90E-04	0	8382
PIGW	-1.90E-04	0	8383
POLR2J	-1.90E-04	0	8384
USH1G	-1.90E-04	0	8385
CDCA3	-1.91E-04	0	8386
FIGN	-1.91E-04	0	8387
GALK2	-1.91E-04	0	8388
LGR6	-1.91E-04	0	8389
UBTFL1	-1.91E-04	0	8390
NPFFR2	-1.91E-04	0	8391
GBGT1	-1.92E-04	0	8392
TRIM9	-1.92E-04	0	8393
SLC31A1P1	-1.92E-04	0	8394
MT1H	-1.92E-04	0	8395
PANX3	-1.93E-04	0	8396
GPR152	-1.93E-04	0	8397
ARHGAP33	-1.93E-04	0	8398
HMG20B	-1.93E-04	0	8399
H2AFV	-1.93E-04	0	8400
ST8	-1.94E-04	0	8401
SCGN	-1.95E-04	0	8402
VASN	-1.95E-04	0	8403
SQLE	-1.96E-04	0	8404
KPNB1	-1.96E-04	0	8405
HIST1H3B	-1.96E-04	0	8406
BPPV	-1.96E-04	0	8407
S100A16	-1.96E-04	0	8408
KCNT2	-1.98E-04	0	8409
CCM2	-1.98E-04	0	8410
TAF7	-1.99E-04	0	8411
ZC3H15	-2.00E-04	0	8412
UTY	-2.00E-04	0	8413
EIF2B2	-2.01E-04	0	8414
OR51E2	-2.01E-04	0	8415
TCERG1L	-2.01E-04	0	8416
15-Sep	-2.01E-04	0	8417
RPL34	-2.02E-04	0	8418
VPS35	-2.02E-04	0	8419
HIGD1A	-2.03E-04	0	8420
GAREM	-2.03E-04	0	8421
DSCR8	-2.04E-04	0	8422
HMX3	-2.04E-04	0	8423

BW62	-2.04E-04	0	8424
MIR767	-2.04E-04	0	8425
MPPED2	-2.04E-04	0	8426
RER1	-2.04E-04	0	8427
TDGF1P7	-2.04E-04	0	8428
CT55	-2.04E-04	0	8429
ACO2	-2.04E-04	0	8430
PEX10	-2.04E-04	0	8431
EYS	-2.05E-04	0	8432
FRG2	-2.05E-04	0	8433
POMGNT1	-2.05E-04	0	8434
TMEM30A	-2.05E-04	0	8435
TROAP	-2.06E-04	0	8436
TRMT10C	-2.06E-04	0	8437
VAPB	-2.06E-04	0	8438
HPPD	-2.06E-04	0	8439
TFAP4	-2.07E-04	0	8440
PEE1	-2.08E-04	0	8441
MAGEB16	-2.08E-04	0	8442
NME8	-2.08E-04	0	8443
PIPSL	-2.08E-04	0	8444
TUBA3C	-2.08E-04	0	8445
ZIM3	-2.08E-04	0	8446
ZKSCAN5	-2.08E-04	0	8447
ZSCAN12	-2.08E-04	0	8448
SUSD2	-2.09E-04	0	8449
GOLGA7	-2.09E-04	0	8450
ST3GAL3	-2.09E-04	0	8451
FRA16D	-2.10E-04	0	8452
MSRB2	-2.10E-04	0	8453
FDX1	-2.10E-04	0	8454
CLF	-2.10E-04	0	8455
PGA3	-2.11E-04	0	8456
ARID4A	-2.11E-04	0	8457
PJA2	-2.11E-04	0	8458
GAGE2C	-2.12E-04	0	8459
ATP13A2	-2.12E-04	0	8460
SNORD47	-2.12E-04	0	8461
BEGAIN	-2.12E-04	0	8462
ACOT7	-2.12E-04	0	8463
IGF2-AS	-2.13E-04	0	8464
LRRC6	-2.13E-04	0	8465
GPX5	-2.14E-04	0	8466

WAPAL	-2.15E-04	0	8467
CAPZA2	-2.16E-04	0	8468
XS	-2.16E-04	0	8469
RIOK3	-2.17E-04	0	8470
CBLN2	-2.18E-04	0	8471
RBPJL	-2.18E-04	0	8472
SLC5A5	-2.18E-04	0	8473
ECM1	-2.18E-04	0	8474
RHOBTB1	-2.19E-04	0	8475
SGPP2	-2.19E-04	0	8476
ME1	-2.19E-04	0	8477
EYA3	-2.19E-04	0	8478
EFHC1	-2.20E-04	0	8479
PUF60	-2.20E-04	0	8480
ICK	-2.20E-04	0	8481
RPL8	-2.20E-04	0	8482
TBL2	-2.21E-04	0	8483
MAGEA6	-2.21E-04	0	8484
MIR1250	-2.21E-04	0	8485
TSKU	-2.21E-04	0	8486
CADM3	-2.22E-04	0	8487
SRP19	-2.23E-04	0	8488
MFSD2A	-2.23E-04	0	8489
ADAMTSL1	-2.23E-04	0	8490
MIR218-1	-2.24E-04	0	8491
BRD3	-2.25E-04	0	8492
KLK12	-2.25E-04	0	8493
KLK9	-2.25E-04	0	8494
RTN4RL1	-2.25E-04	0	8495
CCDC62	-2.25E-04	0	8496
GANAB	-2.26E-04	0	8497
ARL14	-2.27E-04	0	8498
LGMD1D	-2.27E-04	0	8499
FAM189A1	-2.27E-04	0	8500
LTN1	-2.27E-04	0	8501
SMIM3	-2.27E-04	0	8502
MIR3940	-2.27E-04	0	8503
RBMX	-2.28E-04	0	8504
MHS4	-2.28E-04	0	8505
DNAJC1	-2.28E-04	0	8506
GJB7	-2.28E-04	0	8507
TMCO1	-2.28E-04	0	8508
VPS53	-2.28E-04	0	8509

ZCCHC17	-2.28E-04	0	8510
C1ORF228	-2.29E-04	0	8511
STX18	-2.29E-04	0	8512
MIR501	-2.29E-04	0	8513
IPO8	-2.29E-04	0	8514
RIMS3	-2.29E-04	0	8515
INTS3	-2.29E-04	0	8516
PLA2G5	-2.29E-04	0	8517
9-Mar	-2.30E-04	0	8518
LAMTOR3	-2.30E-04	0	8519
CTSZ	-2.30E-04	0	8520
KRT37	-2.31E-04	0	8521
ADCK4	-2.31E-04	0	8522
AOC3	-2.31E-04	0	8523
SLC2A4RG	-2.31E-04	0	8524
MYSM1	-2.32E-04	0	8525
CPLX3	-2.32E-04	0	8526
YTHDF1	-2.33E-04	0	8527
ZP1	-2.33E-04	0	8528
TMEM106B	-2.33E-04	0	8529
NOVA1	-2.33E-04	0	8530
ITPR1	-2.36E-04	0	8531
DARS2	-2.38E-04	0	8532
WDR45	-2.38E-04	0	8533
MIR602	-2.38E-04	0	8534
FOXP4	-2.38E-04	0	8535
ANTXR2	-2.39E-04	0	8536
CAMSAP3	-2.39E-04	0	8537
RNF40	-2.39E-04	0	8538
SEMG1	-2.39E-04	0	8539
THADA	-2.39E-04	0	8540
MAP1A	-2.40E-04	0	8541
STT3B	-2.40E-04	0	8542
NONO	-2.40E-04	0	8543
HPE1	-2.41E-04	0	8544
FRA6E	-2.41E-04	0	8545
RAB37	-2.42E-04	0	8546
SLC5A6	-2.43E-04	0	8547
YTHDF3	-2.43E-04	0	8548
RSPRY1	-2.43E-04	0	8549
DCTD	-2.44E-04	0	8550
SELT	-2.44E-04	0	8551
MIR1182	-2.44E-04	0	8552

NUBP2	-2.45E-04	0	8553
GM2AP1	-2.45E-04	0	8554
GUF1	-2.45E-04	0	8555
PLA2G2A	-2.47E-04	0	8556
MKRN1	-2.47E-04	0	8557
LPAR4	-2.48E-04	0	8558
DPYS	-2.48E-04	0	8559
NEUROG2	-2.48E-04	0	8560
GPRC5D	-2.48E-04	0	8561
PRDX3	-2.49E-04	0	8562
ALG6	-2.49E-04	0	8563
ABCC4	-2.49E-04	0	8564
HLA-H	-2.49E-04	0	8565
NSUN4	-2.49E-04	0	8566
SLC46A1	-2.49E-04	0	8567
NKX2-8	-2.49E-04	0	8568
MICC	-2.50E-04	0	8569
MICF	-2.50E-04	0	8570
TFAP2D	-2.51E-04	0	8571
NOP10	-2.52E-04	0	8572
NENF	-2.52E-04	0	8573
PDZD4	-2.53E-04	0	8574
KCTD14	-2.53E-04	0	8575
KDM2A	-2.54E-04	0	8576
VEZT	-2.54E-04	0	8577
IGKJ3	-2.55E-04	0	8578
MIR374B	-2.55E-04	0	8579
LACRT	-2.56E-04	0	8580
PGA5	-2.56E-04	0	8581
MGR3	-2.56E-04	0	8582
ST6GALNAC4	-2.56E-04	0	8583
ATP8	-2.58E-04	0	8584
TCTN2	-2.59E-04	0	8585
MEX3A	-2.59E-04	0	8586
MIR568	-2.60E-04	0	8587
MIR663B	-2.60E-04	0	8588
UPK1A	-2.61E-04	0	8589
PRRG1	-2.61E-04	0	8590
MTIF2	-2.61E-04	0	8591
AZIN2	-2.61E-04	0	8592
SPA17	-2.62E-04	0	8593
DISP1	-2.62E-04	0	8594
SLC4A10	-2.62E-04	0	8595

NIFK	-2.65E-04	0	8596
DTX3	-2.65E-04	0	8597
RSPH1	-2.65E-04	0	8598
IS5	-2.66E-04	0	8599
BNC1	-2.66E-04	0	8600
GRIPAP1	-2.67E-04	0	8601
BRD8	-2.67E-04	0	8602
ABTB1	-2.67E-04	0	8603
KCTD5	-2.67E-04	0	8604
KCTD6	-2.67E-04	0	8605
KLHL10	-2.67E-04	0	8606
KLHL25	-2.67E-04	0	8607
KLHL9	-2.67E-04	0	8608
RGS19	-2.69E-04	0	8609
EID3	-2.69E-04	0	8610
QRFP	-2.69E-04	0	8611
LGSN	-2.70E-04	0	8612
METTL21D	-2.70E-04	0	8613
UBXN4	-2.70E-04	0	8614
YOD1	-2.70E-04	0	8615
RTE1	-2.71E-04	0	8616
POLI	-2.72E-04	0	8617
KRT40	-2.73E-04	0	8618
BARX1	-2.73E-04	0	8619
NHP2	-2.73E-04	0	8620
NDUFA7	-2.74E-04	0	8621
PRPSAP1	-2.75E-04	0	8622
ZBTB7C	-2.75E-04	0	8623
FXYD4	-2.77E-04	0	8624
ZNF443	-2.77E-04	0	8625
CLCP1	-2.78E-04	0	8626
SLC25A31	-2.79E-04	0	8627
BMP2K	-2.79E-04	0	8628
CSRNP2	-2.79E-04	0	8629
MYNN	-2.79E-04	0	8630
NCKAP5L	-2.79E-04	0	8631
SLC35B4	-2.79E-04	0	8632
PEX16	-2.79E-04	0	8633
CENPF	-2.79E-04	0	8634
IL20RA	-2.80E-04	0	8635
PGK2	-2.80E-04	0	8636
SEC61G	-2.81E-04	0	8637
SRSF6	-2.81E-04	0	8638

KCNJ13	-2.81E-04	0	8639
RNF112	-2.82E-04	0	8640
CDC42EP1	-2.82E-04	0	8641
DCTN2	-2.82E-04	0	8642
ATP5O	-2.84E-04	0	8643
TRO	-2.84E-04	0	8644
CDO1	-2.85E-04	0	8645
AUTS1	-2.86E-04	0	8646
MAP6	-2.86E-04	0	8647
WHRN	-2.86E-04	0	8648
FSHMD1A	-2.87E-04	0	8649
GRPEL1	-2.87E-04	0	8650
UPB1	-2.87E-04	0	8651
GABRA2	-2.87E-04	0	8652
MIR877	-2.87E-04	0	8653
PIRT	-2.88E-04	0	8654
CSAG2	-2.89E-04	0	8655
PTGR2	-2.89E-04	0	8656
CASC1	-2.89E-04	0	8657
ZNF266	-2.89E-04	0	8658
RHBG	-2.90E-04	0	8659
SCO1	-2.90E-04	0	8660
POLA2	-2.93E-04	0	8661
CDK11B	-2.93E-04	0	8662
ANAPC16	-2.94E-04	0	8663
CSNK2A2	-2.95E-04	0	8664
RPL13	-2.95E-04	0	8665
IFT27	-2.96E-04	0	8666
AKAP7	-2.96E-04	0	8667
CBLN1	-2.97E-04	0	8668
HS3ST3B1	-2.97E-04	0	8669
TRAF3IP1	-2.98E-04	0	8670
HPS6	-2.98E-04	0	8671
LINC00092	-2.98E-04	0	8672
STXBP2	-2.98E-04	0	8673
C8ORF4	-2.99E-04	0	8674
AFD1	-2.99E-04	0	8675
SMC1B	-2.99E-04	0	8676
PSG7	-2.99E-04	0	8677
MIR384	-3.00E-04	0	8678
MIR569	-3.00E-04	0	8679
ASAP2	-3.00E-04	0	8680
DBA2	-3.00E-04	0	8681

TRAK2	-3.01E-04	0	8682
TCF20	-3.01E-04	0	8683
ZKSCAN1	-3.02E-04	0	8684
SAGE1	-3.02E-04	0	8685
TXNDC9	-3.03E-04	0	8686
SMEK2	-3.03E-04	0	8687
HKR1	-3.04E-04	0	8688
SNORD24	-3.04E-04	0	8689
TAS2R4	-3.04E-04	0	8690
RGS11	-3.04E-04	0	8691
MTRNR2L10	-3.05E-04	0	8692
MYRF	-3.05E-04	0	8693
AD9	-3.06E-04	0	8694
ADCK3	-3.06E-04	0	8695
SCN1B	-3.06E-04	0	8696
LPAR6	-3.07E-04	0	8697
CSDA	-3.07E-04	0	8698
NCLN	-3.08E-04	0	8699
OTUD7A	-3.09E-04	0	8700
INADL	-3.09E-04	0	8701
CNGB1	-3.12E-04	0	8702
PGM5	-3.14E-04	0	8703
PDK3	-3.14E-04	0	8704
ARCN1	-3.14E-04	0	8705
TULP3	-3.14E-04	0	8706
ENAM	-3.15E-04	0	8707
CCL3L1	-3.16E-04	0	8708
WDR7	-3.16E-04	0	8709
FERD3L	-3.17E-04	0	8710
COL11A1	-3.17E-04	0	8711
HPVC1	-3.18E-04	0	8712
CHIT1	-3.18E-04	0	8713
MYO18A	-3.20E-04	0	8714
ATG4D	-3.20E-04	0	8715
DPH3	-3.20E-04	0	8716
IPO11	-3.21E-04	0	8717
TAOK2	-3.21E-04	0	8718
IGKV2-10	-3.23E-04	0	8719
STRA8	-3.23E-04	0	8720
KRTAP1-3	-3.24E-04	0	8721
PALD1	-3.24E-04	0	8722
MTMR2	-3.24E-04	0	8723
TOMM6	-3.25E-04	0	8724

SDK1	-3.25E-04	0	8725
L1RE2	-3.25E-04	0	8726
ZNF177	-3.25E-04	0	8727
DPYD	-3.26E-04	0	8728
NPBWR2	-3.27E-04	0	8729
HOGA1	-3.28E-04	0	8730
GPD2	-3.28E-04	0	8731
KPNA6	-3.28E-04	0	8732
MOCS3	-3.29E-04	0	8733
PEPB	-3.29E-04	0	8734
AMY1C	-3.29E-04	0	8735
UNC5B	-3.29E-04	0	8736
PPME1	-3.31E-04	0	8737
PRKRA	-3.31E-04	0	8738
NUP35	-3.32E-04	0	8739
DSC3	-3.32E-04	0	8740
CDC26	-3.32E-04	0	8741
ZNF185	-3.33E-04	0	8742
RPL7A	-3.34E-04	0	8743
PRICKLE2	-3.35E-04	0	8744
LRIG1	-3.35E-04	0	8745
PLBD2	-3.35E-04	0	8746
TAF5	-3.35E-04	0	8747
SLC25A21	-3.36E-04	0	8748
KIF27	-3.36E-04	0	8749
MYL9	-3.36E-04	0	8750
MYO15A	-3.38E-04	0	8751
WDFY1	-3.39E-04	0	8752
DIS3	-3.39E-04	0	8753
BTBD3	-3.39E-04	0	8754
USP12	-3.39E-04	0	8755
FKBP14	-3.39E-04	0	8756
CRYGB	-3.40E-04	0	8757
SUCO	-3.40E-04	0	8758
FAM120C	-3.41E-04	0	8759
P4HA2	-3.41E-04	0	8760
SPAG4	-3.41E-04	0	8761
CCNYL1	-3.41E-04	0	8762
SAP130	-3.42E-04	0	8763
ZCCHC5	-3.43E-04	0	8764
NIPA1	-3.44E-04	0	8765
CPNE3	-3.44E-04	0	8766
PLXNA3	-3.44E-04	0	8767

DNAH12	-3.44E-04	0	8768
AZIN1	-3.44E-04	0	8769
SFTPA2	-3.45E-04	0	8770
FOLR2	-3.46E-04	0	8771
KDM6A	-3.46E-04	0	8772
WT1-AS	-3.47E-04	0	8773
RSL1D1	-3.47E-04	0	8774
CDR2L	-3.47E-04	0	8775
GABRA1	-3.47E-04	0	8776
PHA2A	-3.48E-04	0	8777
PPFIA1	-3.48E-04	0	8778
ZNF503	-3.49E-04	0	8779
IARS2	-3.49E-04	0	8780
IS2	-3.49E-04	0	8781
CELA2A	-3.49E-04	0	8782
NDUFS6	-3.50E-04	0	8783
CYLC1	-3.51E-04	0	8784
SSX5	-3.52E-04	0	8785
PMCH	-3.53E-04	0	8786
HOXC5	-3.54E-04	0	8787
SLC39A13	-3.55E-04	0	8788
C10ORF2	-3.56E-04	0	8789
RGS12	-3.56E-04	0	8790
UPF3B	-3.58E-04	0	8791
TINAGL1	-3.59E-04	0	8792
FBXW5	-3.60E-04	0	8793
UNC5C	-3.60E-04	0	8794
TAF1B	-3.61E-04	0	8795
GLRA1	-3.61E-04	0	8796
SNX6	-3.62E-04	0	8797
TAGLN3	-3.62E-04	0	8798
CHRNB4	-3.62E-04	0	8799
SLC36A4	-3.62E-04	0	8800
DLK2	-3.63E-04	0	8801
MIR544A	-3.63E-04	0	8802
ZNF296	-3.63E-04	0	8803
TMOD3	-3.64E-04	0	8804
FBXW10	-3.64E-04	0	8805
CNGA3	-3.64E-04	0	8806
CDH20	-3.65E-04	0	8807
HHATL	-3.65E-04	0	8808
RNF32	-3.65E-04	0	8809
STOX1	-3.65E-04	0	8810

DSP	-3.66E-04	0	8811
LAPTM4B	-3.66E-04	0	8812
SF3B3	-3.66E-04	0	8813
SPPL2C	-3.66E-04	0	8814
CBY1	-3.68E-04	0	8815
KDM1B	-3.68E-04	0	8816
BLOC1S4	-3.69E-04	0	8817
ACTG2	-3.69E-04	0	8818
C1QL3	-3.69E-04	0	8819
TPD52L3	-3.70E-04	0	8820
HGD	-3.71E-04	0	8821
LIPI	-3.72E-04	0	8822
CSAD	-3.72E-04	0	8823
CACNA2D4	-3.72E-04	0	8824
IFT46	-3.73E-04	0	8825
RPE65	-3.73E-04	0	8826
CPSF3	-3.73E-04	0	8827
GK5	-3.73E-04	0	8828
MIR708	-3.73E-04	0	8829
MEG8	-3.74E-04	0	8830
PSG3	-3.74E-04	0	8831
CDCA4	-3.75E-04	0	8832
SLC22A11	-3.75E-04	0	8833
XIRP1	-3.75E-04	0	8834
TAF7L	-3.75E-04	0	8835
SYCP1	-3.76E-04	0	8836
TNNC1	-3.76E-04	0	8837
CENPC	-3.76E-04	0	8838
MIR873	-3.77E-04	0	8839
NAPB	-3.78E-04	0	8840
SNORA44	-3.78E-04	0	8841
PEO1	-3.78E-04	0	8842
OAZ2	-3.78E-04	0	8843
DEL2P21	-3.80E-04	0	8844
CARM1	-3.80E-04	0	8845
STARD7	-3.80E-04	0	8846
STARD5	-3.82E-04	0	8847
FRA2E	-3.82E-04	0	8848
BLVRB	-3.83E-04	0	8849
SAMD4B	-3.83E-04	0	8850
SLC35B3	-3.84E-04	0	8851
GPR101	-3.84E-04	0	8852
TLK2	-3.84E-04	0	8853

KRT84	-3.85E-04	0	8854
DAOA	-3.85E-04	0	8855
UBB	-3.85E-04	0	8856
PTCH1	-3.86E-04	0	8857
ALS2CR8	-3.87E-04	0	8858
SYMPK	-3.87E-04	0	8859
AMOTL2	-3.88E-04	0	8860
MATN4	-3.89E-04	0	8861
MIR215	-3.90E-04	0	8862
F11	-3.90E-04	0	8863
MIR150	-3.90E-04	0	8864
RREB1	-3.91E-04	0	8865
NAP1L2	-3.92E-04	0	8866
TRPM4	-3.92E-04	0	8867
SLC5A4	-3.92E-04	0	8868
MIEF1	-3.92E-04	0	8869
IGKV3-20	-3.93E-04	0	8870
XRN2	-3.94E-04	0	8871
ZFAND2A	-3.94E-04	0	8872
SFR1	-3.94E-04	0	8873
GSAP	-3.94E-04	0	8874
SNX10	-3.94E-04	0	8875
IFITM2	-3.95E-04	0	8876
GPR135	-3.95E-04	0	8877
FAM171A1	-3.96E-04	0	8878
TOR4A	-3.96E-04	0	8879
RASD2	-3.98E-04	0	8880
CST2	-3.98E-04	0	8881
OPN4	-3.98E-04	0	8882
RPSA	-3.98E-04	0	8883
PRKAB1	-3.99E-04	0	8884
ODR4	-3.99E-04	0	8885
BDMR	-4.00E-04	0	8886
MID1	-4.00E-04	0	8887
SAMD9	-4.00E-04	0	8888
CCT6A	-4.00E-04	0	8889
CLTA	-4.00E-04	0	8890
COQ6	-4.01E-04	0	8891
CYP2R1	-4.01E-04	0	8892
LSAMP	-4.01E-04	0	8893
MPZL2	-4.01E-04	0	8894
BCORL1	-4.01E-04	0	8895
OTUD5	-4.02E-04	0	8896

FAM92B	-4.02E-04	0	8897
RXFP4	-4.03E-04	0	8898
HOXD3	-4.03E-04	0	8899
PSME2	-4.04E-04	0	8900
RNF43	-4.04E-04	0	8901
BTN3A2	-4.04E-04	0	8902
ULK3	-4.05E-04	0	8903
MIR96	-4.05E-04	0	8904
MTA3	-4.05E-04	0	8905
LYPLAL1	-4.06E-04	0	8906
CFAP53	-4.06E-04	0	8907
CCNT2	-4.06E-04	0	8908
YWHAB	-4.07E-04	0	8909
ROM1	-4.07E-04	0	8910
MIR493	-4.09E-04	0	8911
C22ORF28	-4.09E-04	0	8912
USH1A	-4.10E-04	0	8913
S100A7A	-4.10E-04	0	8914
GNL1	-4.11E-04	0	8915
CACNA2D2	-4.11E-04	0	8916
GOLM1	-4.11E-04	0	8917
TRAK1	-4.11E-04	0	8918
DNER	-4.12E-04	0	8919
SMTNL1	-4.13E-04	0	8920
MIR372	-4.13E-04	0	8921
ANXA11	-4.15E-04	0	8922
SLC35C1	-4.15E-04	0	8923
MIR299	-4.16E-04	0	8924
AFAP1-AS1	-4.16E-04	0	8925
MYOT	-4.16E-04	0	8926
NSF	-4.17E-04	0	8927
SLC35A4	-4.18E-04	0	8928
IDNK	-4.18E-04	0	8929
MEX3D	-4.18E-04	0	8930
THAP11	-4.19E-04	0	8931
GPR125	-4.19E-04	0	8932
PRBNS	-4.19E-04	0	8933
FAM98B	-4.20E-04	0	8934
TBX18	-4.21E-04	0	8935
KCNIP3	-4.22E-04	0	8936
APH1A	-4.22E-04	0	8937
AP1G1	-4.23E-04	0	8938
COX10	-4.23E-04	0	8939

MST4	-4.23E-04	0	8940
PSMD6	-4.23E-04	0	8941
NYS3	-4.24E-04	0	8942
ACSS1	-4.25E-04	0	8943
CLIP3	-4.26E-04	0	8944
CEP152	-4.26E-04	0	8945
RRP1B	-4.27E-04	0	8946
KLHL6	-4.27E-04	0	8947
IGKJ5	-4.28E-04	0	8948
CCAR2	-4.29E-04	0	8949
LCA9	-4.29E-04	0	8950
RRP8	-4.29E-04	0	8951
MIR4458	-4.30E-04	0	8952
ADAMTS9-AS	-4.30E-04	0	8953
PCGF1	-4.30E-04	0	8954
TMEM67	-4.31E-04	0	8955
SLC4A8	-4.32E-04	0	8956
MEA1	-4.32E-04	0	8957
RGS8	-4.33E-04	0	8958
SNAPC3	-4.33E-04	0	8959
SPINK6	-4.33E-04	0	8960
IGKV3D-7	-4.34E-04	0	8961
NXN	-4.34E-04	0	8962
TDRD6	-4.36E-04	0	8963
CND	-4.36E-04	0	8964
SCA29	-4.36E-04	0	8965
MIR520B	-4.36E-04	0	8966
TGM1	-4.37E-04	0	8967
LDOC1	-4.39E-04	0	8968
GBP2	-4.39E-04	0	8969
HIBADH	-4.40E-04	0	8970
TRNAT2	-4.40E-04	0	8971
MCO	-4.41E-04	0	8972
CEP192	-4.41E-04	0	8973
ADAM2	-4.41E-04	0	8974
TRDMT1	-4.42E-04	0	8975
MIR218-2	-4.43E-04	0	8976
EVL	-4.43E-04	0	8977
DGCR	-4.43E-04	0	8978
TRAPPC8	-4.43E-04	0	8979
S100A3	-4.44E-04	0	8980
RANGRF	-4.45E-04	0	8981
TSEN54	-4.45E-04	0	8982

SSR1	-4.45E-04	0	8983
DYRK1A	-4.45E-04	0	8984
CLCNKA	-4.46E-04	0	8985
ST18	-4.50E-04	0	8986
RNF183	-4.50E-04	0	8987
PIGG	-4.51E-04	0	8988
SNORD118	-4.51E-04	0	8989
BAG6	-4.52E-04	0	8990
HMP19	-4.52E-04	0	8991
PDZD3	-4.53E-04	0	8992
ECA1	-4.53E-04	0	8993
COBL	-4.53E-04	0	8994
PRR13	-4.54E-04	0	8995
ZNF607	-4.54E-04	0	8996
ABCB7	-4.57E-04	0	8997
CAND2	-4.58E-04	0	8998
LSM1	-4.58E-04	0	8999
HORMAD1	-4.58E-04	0	9000
NARF	-4.58E-04	0	9001
OLFM2	-4.59E-04	0	9002
DACT1	-4.59E-04	0	9003
IGFBP3	-4.60E-04	0	9004
HOXD11	-4.60E-04	0	9005
CLPP	-4.60E-04	0	9006
TNXB	-4.60E-04	0	9007
SKIV2L2	-4.61E-04	0	9008
NCBP1	-4.62E-04	0	9009
GALM	-4.63E-04	0	9010
SCHIP1	-4.63E-04	0	9011
KBTBD5	-4.65E-04	0	9012
IGHV4-34	-4.66E-04	0	9013
DRP2	-4.67E-04	0	9014
NOL10	-4.67E-04	0	9015
TAC4	-4.68E-04	0	9016
ACER2	-4.68E-04	0	9017
MUC13	-4.69E-04	0	9018
C1QTNF6	-4.71E-04	0	9019
GAR1	-4.71E-04	0	9020
UBE2M	-4.72E-04	0	9021
PDE6C	-4.73E-04	0	9022
SRPRB	-4.73E-04	0	9023
C7ORF10	-4.73E-04	0	9024
PLXDC1	-4.75E-04	0	9025

CIART	-4.75E-04	0	9026
KCNN1	-4.75E-04	0	9027
LEKR1	-4.75E-04	0	9028
SLC24A5	-4.75E-04	0	9029
CAMTA1	-4.77E-04	0	9030
TWIST2	-4.77E-04	0	9031
HSPB8	-4.78E-04	0	9032
PPP1R15B	-4.78E-04	0	9033
CDH7	-4.79E-04	0	9034
KIF13A	-4.80E-04	0	9035
ZNF580	-4.80E-04	0	9036
NECAB1	-4.80E-04	0	9037
ABCA2	-4.80E-04	0	9038
SS3	-4.81E-04	0	9039
SLC38A1	-4.81E-04	0	9040
KCNA7	-4.82E-04	0	9041
GZF1	-4.82E-04	0	9042
TXLNG	-4.84E-04	0	9043
SEPT7P2	-4.84E-04	0	9044
EVC2	-4.84E-04	0	9045
PLA2G2D	-4.84E-04	0	9046
ENTPD3	-4.85E-04	0	9047
ZNF362	-4.85E-04	0	9048
CACNB2	-4.85E-04	0	9049
RNF17	-4.86E-04	0	9050
CREBL2	-4.88E-04	0	9051
TEF	-4.88E-04	0	9052
SRRM2	-4.88E-04	0	9053
CDH8	-4.88E-04	0	9054
SMC5	-4.88E-04	0	9055
RGS9BP	-4.88E-04	0	9056
TCP10	-4.89E-04	0	9057
HEPACAM2	-4.90E-04	0	9058
CISD2	-4.90E-04	0	9059
AP5M1	-4.91E-04	0	9060
TMEM5	-4.91E-04	0	9061
KIFAP3	-4.91E-04	0	9062
CBLL1	-4.91E-04	0	9063
ENOX2	-4.92E-04	0	9064
COL4A4	-4.92E-04	0	9065
PSMA3	-4.93E-04	0	9066
NUP93	-4.94E-04	0	9067
IL13RA1	-4.94E-04	0	9068

P2RY14	-4.94E-04	0	9069
ARL6IP5	-4.95E-04	0	9070
CENPU	-4.95E-04	0	9071
TMEM245	-4.95E-04	0	9072
ZFP57	-4.95E-04	0	9073
MIR1293	-4.95E-04	0	9074
MOGS	-4.96E-04	0	9075
QTRT1	-4.96E-04	0	9076
ATXN3L	-4.96E-04	0	9077
PLLP	-4.96E-04	0	9078
POLR3D	-4.96E-04	0	9079
HRNR	-4.97E-04	0	9080
CASK	-4.97E-04	0	9081
MF4	-4.97E-04	0	9082
TMEM154	-4.97E-04	0	9083
CDK12	-4.98E-04	0	9084
MFAP2	-4.99E-04	0	9085
GPR26	-4.99E-04	0	9086
RPF2	-5.00E-04	0	9087
STARD4	-5.00E-04	0	9088
CD3EAP	-5.01E-04	0	9089
ANAPC10	-5.01E-04	0	9090
PARP16	-5.01E-04	0	9091
NLGN3	-5.03E-04	0	9092
BCPR	-5.04E-04	0	9093
FAM180A	-5.05E-04	0	9094
FIBIN	-5.05E-04	0	9095
TRAP	-5.05E-04	0	9096
HOXC10	-5.06E-04	0	9097
IGKJ1	-5.06E-04	0	9098
HTR3E	-5.07E-04	0	9099
FANCL	-5.08E-04	0	9100
CAMK1D	-5.09E-04	0	9101
ASMD	-5.10E-04	0	9102
SVEP1	-5.10E-04	0	9103
TSPAN10	-5.12E-04	0	9104
TSPAN14	-5.12E-04	0	9105
TSPAN17	-5.12E-04	0	9106
TSPAN33	-5.12E-04	0	9107
SRP54	-5.13E-04	0	9108
MIR208B	-5.13E-04	0	9109
DHFRL1	-5.13E-04	0	9110
RBM7	-5.13E-04	0	9111

DIS3L2	-5.14E-04	0	9112
SLC39A11	-5.14E-04	0	9113
APOBEC3D	-5.15E-04	0	9114
RAX2	-5.15E-04	0	9115
SAP30BP	-5.15E-04	0	9116
SEZ6L2	-5.16E-04	0	9117
LMAN2	-5.16E-04	0	9118
MRS2	-5.16E-04	0	9119
RAB3C	-5.17E-04	0	9120
GML	-5.17E-04	0	9121
YBX2	-5.17E-04	0	9122
P2RX6	-5.18E-04	0	9123
CRTAP	-5.18E-04	0	9124
PHF1	-5.20E-04	0	9125
DDA1	-5.21E-04	0	9126
CENPQ	-5.21E-04	0	9127
PLK1S1	-5.21E-04	0	9128
PLK5	-5.21E-04	0	9129
RUSC2	-5.21E-04	0	9130
PARS2	-5.21E-04	0	9131
GSPT1	-5.22E-04	0	9132
MYCT1	-5.22E-04	0	9133
MRFAP1	-5.23E-04	0	9134
AMPD2	-5.23E-04	0	9135
CCDC22	-5.24E-04	0	9136
CNGA2	-5.24E-04	0	9137
RGS3	-5.24E-04	0	9138
ATOX1	-5.24E-04	0	9139
TRA	-5.24E-04	0	9140
RABEP2	-5.25E-04	0	9141
IFNL1	-5.25E-04	0	9142
ERCC6L	-5.26E-04	0	9143
BCAT1	-5.26E-04	0	9144
PTTG1IP	-5.26E-04	0	9145
ARNTL2	-5.26E-04	0	9146
DUSP8	-5.26E-04	0	9147
GJD3	-5.27E-04	0	9148
FUSE	-5.27E-04	0	9149
SUN2	-5.27E-04	0	9150
HLA-DQA2	-5.27E-04	0	9151
SCA19	-5.28E-04	0	9152
DSCAM	-5.29E-04	0	9153
REEP1	-5.29E-04	0	9154

KLC2	-5.30E-04	0	9155
ERP27	-5.30E-04	0	9156
ZBTB2	-5.30E-04	0	9157
DEPDC5	-5.31E-04	0	9158
PMP2	-5.31E-04	0	9159
GSG2	-5.32E-04	0	9160
PATL1	-5.32E-04	0	9161
FOLR1	-5.32E-04	0	9162
GPR27	-5.34E-04	0	9163
IST1	-5.36E-04	0	9164
CLUAP1	-5.36E-04	0	9165
PASK	-5.37E-04	0	9166
C1ORF61	-5.37E-04	0	9167
IKZF4	-5.37E-04	0	9168
IGKV1-37	-5.40E-04	0	9169
MTCH2	-5.40E-04	0	9170
STX12	-5.41E-04	0	9171
PRKCG	-5.41E-04	0	9172
GNA11	-5.42E-04	0	9173
PICK1	-5.44E-04	0	9174
UROC1	-5.44E-04	0	9175
ARID5B	-5.45E-04	0	9176
MIEF2	-5.46E-04	0	9177
H3.X	-5.47E-04	0	9178
GALNT1	-5.47E-04	0	9179
RNF144A	-5.48E-04	0	9180
GSDMC	-5.50E-04	0	9181
SEZ6	-5.50E-04	0	9182
SUMF1	-5.51E-04	0	9183
SAE1	-5.52E-04	0	9184
MIR320A	-5.52E-04	0	9185
ANGEL1	-5.53E-04	0	9186
CCDC91	-5.53E-04	0	9187
PIGS	-5.53E-04	0	9188
APBB3	-5.54E-04	0	9189
NUP50	-5.54E-04	0	9190
LLGL2	-5.56E-04	0	9191
FAM162A	-5.56E-04	0	9192
HOXC11	-5.57E-04	0	9193
MARS	-5.57E-04	0	9194
PNISR	-5.57E-04	0	9195
SLC39A3	-5.58E-04	0	9196
LST1	-5.60E-04	0	9197

CHRND	-5.60E-04	0	9198
NBPF14	-5.60E-04	0	9199
ZNF692	-5.61E-04	0	9200
STOX2	-5.62E-04	0	9201
SNORD82	-5.63E-04	0	9202
IGKV2-18	-5.63E-04	0	9203
MCM9	-5.63E-04	0	9204
SULT1B1	-5.63E-04	0	9205
PSMD12	-5.65E-04	0	9206
IGKV2-24	-5.65E-04	0	9207
XIC	-5.65E-04	0	9208
PDCD11	-5.66E-04	0	9209
PGD	-5.66E-04	0	9210
LINC00473	-5.67E-04	0	9211
KIF26A	-5.67E-04	0	9212
UTS2B	-5.67E-04	0	9213
PSMG3	-5.68E-04	0	9214
IRX4	-5.70E-04	0	9215
ZNF24	-5.70E-04	0	9216
KCNIP4	-5.70E-04	0	9217
ZNF709	-5.71E-04	0	9218
SLC23A2	-5.71E-04	0	9219
NPAS3	-5.71E-04	0	9220
AOC2	-5.72E-04	0	9221
ELF1	-5.72E-04	0	9222
OA1	-5.73E-04	0	9223
FOXS1	-5.74E-04	0	9224
NPRL3	-5.74E-04	0	9225
TPSD1	-5.74E-04	0	9226
TDRD1	-5.75E-04	0	9227
TREX2	-5.76E-04	0	9228
PABPC1L	-5.76E-04	0	9229
VTI1B	-5.77E-04	0	9230
UPK3A	-5.78E-04	0	9231
PVRL3	-5.78E-04	0	9232
GYLTL1B	-5.78E-04	0	9233
CDKN3	-5.78E-04	0	9234
SOGA1	-5.78E-04	0	9235
CPA4	-5.79E-04	0	9236
ANXA2R	-5.79E-04	0	9237
PPP1R3C	-5.80E-04	0	9238
FOXL1	-5.81E-04	0	9239
TANC1	-5.81E-04	0	9240

CECR2	-5.81E-04	0	9241
WBP5	-5.82E-04	0	9242
APOC4	-5.82E-04	0	9243
CWC22	-5.83E-04	0	9244
FCN3	-5.84E-04	0	9245
OAZ3	-5.84E-04	0	9246
PGRMC2	-5.86E-04	0	9247
LTA4H	-5.86E-04	0	9248
NANOS1	-5.87E-04	0	9249
RAB11FIP2	-5.88E-04	0	9250
SERPINB9	-5.88E-04	0	9251
ACKR4	-5.89E-04	0	9252
ERLIN2	-5.89E-04	0	9253
KRT80	-5.90E-04	0	9254
MIR487B	-5.93E-04	0	9255
OR5AC2	-5.93E-04	0	9256
POT1	-5.93E-04	0	9257
CRB1	-5.94E-04	0	9258
PRB4	-5.95E-04	0	9259
SLC16A8	-5.95E-04	0	9260
FBXO10	-5.95E-04	0	9261
PPP2R2C	-5.95E-04	0	9262
RFTN1	-5.95E-04	0	9263
MOV10	-5.96E-04	0	9264
NANP	-5.97E-04	0	9265
CYP46A1	-5.97E-04	0	9266
RNU7-24P	-5.98E-04	0	9267
DCAF7	-5.98E-04	0	9268
EXOC8	-5.98E-04	0	9269
WIZ	-5.98E-04	0	9270
BSND	-5.99E-04	0	9271
PLXNA2	-6.00E-04	0	9272
PKP4	-6.00E-04	0	9273
MS4	-6.01E-04	0	9274
ERMN	-6.01E-04	0	9275
TM4SF4	-6.01E-04	0	9276
H3F3A	-6.02E-04	0	9277
BRMS1L	-6.03E-04	0	9278
ACADL	-6.03E-04	0	9279
RPS16	-6.03E-04	0	9280
SNRNP200	-6.04E-04	0	9281
MIR154	-6.04E-04	0	9282
CHMP1B	-6.04E-04	0	9283

MIF4GD	-6.05E-04	0	9284
SLC6A9	-6.05E-04	0	9285
GLRB	-6.05E-04	0	9286
GMFG	-6.06E-04	0	9287
CBLN4	-6.06E-04	0	9288
RAD51D	-6.07E-04	0	9289
HMCN1	-6.10E-04	0	9290
DZIP1	-6.12E-04	0	9291
SUPT4H1	-6.12E-04	0	9292
KANSL3	-6.13E-04	0	9293
COL8A1	-6.13E-04	0	9294
CLDN18	-6.14E-04	0	9295
SHROOM2	-6.14E-04	0	9296
DDAH1	-6.15E-04	0	9297
GSX2	-6.15E-04	0	9298
UPF3A	-6.15E-04	0	9299
CD160	-6.16E-04	0	9300
MRSD	-6.17E-04	0	9301
ANKRD30A	-6.17E-04	0	9302
CDK5RAP2	-6.18E-04	0	9303
SIX6	-6.18E-04	0	9304
KCNG3	-6.19E-04	0	9305
GLCE	-6.21E-04	0	9306
DANCR	-6.21E-04	0	9307
PYGO1	-6.21E-04	0	9308
OMA1	-6.23E-04	0	9309
VAMP4	-6.23E-04	0	9310
SDHC	-6.25E-04	0	9311
KIFC3	-6.25E-04	0	9312
GYS2	-6.27E-04	0	9313
OSBPL6	-6.28E-04	0	9314
PEBP4	-6.29E-04	0	9315
ARIH1	-6.29E-04	0	9316
SLC25A14	-6.29E-04	0	9317
NUPR1L	-6.31E-04	0	9318
BAZ2A	-6.31E-04	0	9319
SOSTDC1	-6.32E-04	0	9320
RAB3D	-6.32E-04	0	9321
CTAGE1	-6.33E-04	0	9322
MIR577	-6.35E-04	0	9323
CASKIN1	-6.37E-04	0	9324
PCMT1	-6.37E-04	0	9325
LSM11	-6.37E-04	0	9326

RNU12-2P	-6.37E-04	0	9327
NO66	-6.39E-04	0	9328
PSAT1	-6.40E-04	0	9329
IMPG2	-6.40E-04	0	9330
TAF4	-6.40E-04	0	9331
PPP1R14C	-6.40E-04	0	9332
IL12RB2	-6.42E-04	0	9333
INTS4	-6.42E-04	0	9334
TBC1D5	-6.42E-04	0	9335
LRP8	-6.43E-04	0	9336
ANKRD36B	-6.43E-04	0	9337
ST8SIA4	-6.43E-04	0	9338
MIR26B	-6.44E-04	0	9339
GTF2E2	-6.45E-04	0	9340
TRIM36	-6.46E-04	0	9341
PDE10A	-6.48E-04	0	9342
RERG	-6.49E-04	0	9343
ACADS	-6.49E-04	0	9344
GDAP1	-6.49E-04	0	9345
RITA1	-6.50E-04	0	9346
PAIP1	-6.50E-04	0	9347
ADAMTS13	-6.52E-04	0	9348
WNT3	-6.52E-04	0	9349
RDPA	-6.52E-04	0	9350
PHLDA2	-6.53E-04	0	9351
HCA1	-6.53E-04	0	9352
COPRS	-6.55E-04	0	9353
EVI2A	-6.55E-04	0	9354
ENSA	-6.56E-04	0	9355
SNORD28	-6.56E-04	0	9356
MEX3B	-6.56E-04	0	9357
HNF4G	-6.57E-04	0	9358
SMEK1	-6.57E-04	0	9359
MINA	-6.58E-04	0	9360
MYEOV	-6.58E-04	0	9361
NOL7	-6.58E-04	0	9362
PCAT1	-6.58E-04	0	9363
MIR490	-6.58E-04	0	9364
EMID1	-6.59E-04	0	9365
MIR1260B	-6.59E-04	0	9366
GLUD1	-6.61E-04	0	9367
ORC3	-6.62E-04	0	9368
MIR369	-6.63E-04	0	9369

VGLL4	-6.63E-04	0	9370
EIF2B5	-6.63E-04	0	9371
EYA2	-6.65E-04	0	9372
SF3B4	-6.66E-04	0	9373
CHRNA10	-6.67E-04	0	9374
SCAMP3	-6.67E-04	0	9375
ADHD1	-6.67E-04	0	9376
MICAL1	-6.67E-04	0	9377
MIR129-2	-6.68E-04	0	9378
TNP2	-6.68E-04	0	9379
STAG3	-6.69E-04	0	9380
PPBPP1	-6.69E-04	0	9381
AZI1	-6.69E-04	0	9382
ARHGAP20	-6.70E-04	0	9383
HMGN4	-6.71E-04	0	9384
SCN3B	-6.71E-04	0	9385
ERCC8	-6.71E-04	0	9386
OTX2	-6.71E-04	0	9387
ALG12	-6.73E-04	0	9388
LRRC7	-6.73E-04	0	9389
SLITRK1	-6.74E-04	0	9390
ZNF195	-6.75E-04	0	9391
GAS8	-6.75E-04	0	9392
SGCG	-6.77E-04	0	9393
SNORA11	-6.77E-04	0	9394
SFXN1	-6.77E-04	0	9395
SMCHD1	-6.77E-04	0	9396
EPSTI1	-6.78E-04	0	9397
NLRP5	-6.78E-04	0	9398
FLRT2	-6.78E-04	0	9399
MUC15	-6.79E-04	0	9400
ACRBP	-6.79E-04	0	9401
TGFB3	-6.79E-04	0	9402
GGT5	-6.79E-04	0	9403
KCNK2	-6.80E-04	0	9404
LHX8	-6.80E-04	0	9405
LOR	-6.81E-04	0	9406
CLK4	-6.82E-04	0	9407
LIN52	-6.82E-04	0	9408
PHYHIP	-6.82E-04	0	9409
PIGO	-6.83E-04	0	9410
MIR940	-6.84E-04	0	9411
HIST1H1D	-6.84E-04	0	9412

HAGH	-6.85E-04	0	9413
SIDT1	-6.86E-04	0	9414
MPP6	-6.86E-04	0	9415
ALDH4A1	-6.87E-04	0	9416
CCDC183	-6.88E-04	0	9417
FABP6	-6.88E-04	0	9418
CNNM4	-6.89E-04	0	9419
ZFYVE9	-6.89E-04	0	9420
CITED4	-6.90E-04	0	9421
MIR1297	-6.90E-04	0	9422
NPY1R	-6.92E-04	0	9423
RNR1	-6.92E-04	0	9424
CEP85	-6.94E-04	0	9425
KIF24	-6.94E-04	0	9426
LRRC45	-6.94E-04	0	9427
HOOK2	-6.94E-04	0	9428
MIR3151	-6.94E-04	0	9429
SETD6	-6.94E-04	0	9430
TNKS1BP1	-6.94E-04	0	9431
AQP12A	-6.94E-04	0	9432
SARNP	-6.95E-04	0	9433
KLHL24	-6.95E-04	0	9434
DDI2	-6.96E-04	0	9435
ENOX1	-6.96E-04	0	9436
FAM134C	-6.96E-04	0	9437
FASTK	-6.96E-04	0	9438
TLN2	-6.98E-04	0	9439
ISCA1	-6.99E-04	0	9440
USPL1	-6.99E-04	0	9441
OIP5-AS1	-7.00E-04	0	9442
TGFBI	-7.00E-04	0	9443
GIGYF2	-7.01E-04	0	9444
FUT6	-7.01E-04	0	9445
CCDC6	-7.01E-04	0	9446
TTI1	-7.02E-04	0	9447
TRPC4	-7.03E-04	0	9448
GPR162	-7.04E-04	0	9449
ZNF423	-7.04E-04	0	9450
ESPN	-7.05E-04	0	9451
ZFYVE16	-7.05E-04	0	9452
TSPY1	-7.06E-04	0	9453
LRP10	-7.06E-04	0	9454
TSPAN31	-7.06E-04	0	9455

RFC3	-7.07E-04	0	9456
NAA15	-7.07E-04	0	9457
ANO10	-7.08E-04	0	9458
HOMER3	-7.08E-04	0	9459
SEC24D	-7.09E-04	0	9460
BAG2	-7.10E-04	0	9461
GPA33	-7.11E-04	0	9462
FANCI	-7.11E-04	0	9463
TMEM98	-7.11E-04	0	9464
CSRP2	-7.11E-04	0	9465
RSPO4	-7.12E-04	0	9466
MIR452	-7.12E-04	0	9467
HPCX	-7.13E-04	0	9468
CADM2	-7.14E-04	0	9469
CFDP1	-7.15E-04	0	9470
EGR4	-7.16E-04	0	9471
PAPD7	-7.16E-04	0	9472
ORAOV1	-7.18E-04	0	9473
MS3	-7.20E-04	0	9474
MIR15A	-7.21E-04	0	9475
RA4	-7.21E-04	0	9476
SGK2	-7.22E-04	0	9477
PPM1A	-7.24E-04	0	9478
CMM	-7.24E-04	0	9479
SLC22A7	-7.26E-04	0	9480
CALML6	-7.29E-04	0	9481
MIS18BP1	-7.29E-04	0	9482
WDM	-7.30E-04	0	9483
POU4F3	-7.30E-04	0	9484
MIR7-1	-7.33E-04	0	9485
MFNG	-7.35E-04	0	9486
HOXC9	-7.35E-04	0	9487
SMARCAD1	-7.36E-04	0	9488
MAN1A2	-7.38E-04	0	9489
SLC45A3	-7.38E-04	0	9490
CD1E	-7.38E-04	0	9491
ZNF777	-7.38E-04	0	9492
PDE6A	-7.41E-04	0	9493
ATP5G1	-7.42E-04	0	9494
ALDH18A1	-7.42E-04	0	9495
DCLRE1A	-7.44E-04	0	9496
14-Sep	-7.46E-04	0	9497
SNAI2	-7.46E-04	0	9498

SPP2	-7.46E-04	0	9499
MIR449A	-7.46E-04	0	9500
MPP4	-7.47E-04	0	9501
STRADA	-7.48E-04	0	9502
RPS23	-7.49E-04	0	9503
ACBD3	-7.49E-04	0	9504
GTF2H4	-7.49E-04	0	9505
MPP3	-7.50E-04	0	9506
RTN4RL2	-7.52E-04	0	9507
POLR2H	-7.53E-04	0	9508
CMC4	-7.53E-04	0	9509
PPFIBP2	-7.53E-04	0	9510
RLN1	-7.54E-04	0	9511
MEP1B	-7.55E-04	0	9512
ANXA13	-7.55E-04	0	9513
RAMP1	-7.55E-04	0	9514
TACC2	-7.56E-04	0	9515
HTR1D	-7.56E-04	0	9516
SSX4	-7.56E-04	0	9517
KCNAB1	-7.57E-04	0	9518
SLC25A11	-7.58E-04	0	9519
PLBD1	-7.59E-04	0	9520
STRC	-7.59E-04	0	9521
TRNAN1	-7.59E-04	0	9522
UBQLN2	-7.62E-04	0	9523
DIO1	-7.64E-04	0	9524
PERP	-7.65E-04	0	9525
PRKAG2	-7.65E-04	0	9526
MIR579	-7.65E-04	0	9527
FUZ	-7.66E-04	0	9528
CCDC130	-7.66E-04	0	9529
DEFB131	-7.66E-04	0	9530
SNX24	-7.66E-04	0	9531
MGA	-7.66E-04	0	9532
NUP210	-7.68E-04	0	9533
CDNF	-7.69E-04	0	9534
MAGED2	-7.70E-04	0	9535
PROC	-7.70E-04	0	9536
SLC30A7	-7.71E-04	0	9537
ABHD6	-7.71E-04	0	9538
KCNK9	-7.72E-04	0	9539
GALNT6	-7.72E-04	0	9540
SNX12	-7.72E-04	0	9541

ARHGAP19	-7.74E-04	0	9542
FGF11	-7.74E-04	0	9543
NAIF1	-7.74E-04	0	9544
TMEM92	-7.74E-04	0	9545
NABP1	-7.75E-04	0	9546
RPAP1	-7.75E-04	0	9547
CXCL16	-7.76E-04	0	9548
TFAP2B	-7.77E-04	0	9549
L2HGDH	-7.78E-04	0	9550
CFHR3	-7.79E-04	0	9551
ATG101	-7.79E-04	0	9552
GALNT3	-7.80E-04	0	9553
SPRY4-IT1	-7.81E-04	0	9554
MDFIC	-7.82E-04	0	9555
LRBA	-7.82E-04	0	9556
NTMT1	-7.82E-04	0	9557
TCEB3	-7.83E-04	0	9558
YRDC	-7.83E-04	0	9559
HHC3	-7.84E-04	0	9560
MYO18B	-7.86E-04	0	9561
LINC00511	-7.86E-04	0	9562
MLVI2	-7.86E-04	0	9563
NHP2L1	-7.87E-04	0	9564
ABCB9	-7.91E-04	0	9565
GTPBP6	-7.93E-04	0	9566
LRR1	-7.93E-04	0	9567
PPP3CC	-7.93E-04	0	9568
RAB3IL1	-7.93E-04	0	9569
RPL36A	-7.93E-04	0	9570
SYT9	-7.93E-04	0	9571
MIR582	-7.93E-04	0	9572
PPIH	-7.94E-04	0	9573
TAX1BP3	-7.94E-04	0	9574
MLL3	-7.94E-04	0	9575
MIR125B1	-7.94E-04	0	9576
TRPM1	-7.96E-04	0	9577
LILRA6	-7.97E-04	0	9578
AGMAT	-7.97E-04	0	9579
TP53BP1	-7.97E-04	0	9580
PLSCR3	-7.99E-04	0	9581
FMR1-AS1	-7.99E-04	0	9582
FRAXA	-7.99E-04	0	9583
MIR711	-8.01E-04	0	9584

CXCL17	-8.02E-04	0	9585
LINGO1	-8.03E-04	0	9586
TEAD1	-8.04E-04	0	9587
KCNH6	-8.05E-04	0	9588
RBFA	-8.05E-04	0	9589
LAMB2	-8.08E-04	0	9590
SMDT1	-8.08E-04	0	9591
TGM7	-8.09E-04	0	9592
PMS2CL	-8.11E-04	0	9593
PPP1R12C	-8.11E-04	0	9594
PPP1R16B	-8.12E-04	0	9595
RABIF	-8.15E-04	0	9596
ITGBL1	-8.16E-04	0	9597
MIR661	-8.16E-04	0	9598
MAP3K6	-8.16E-04	0	9599
RGSL1	-8.16E-04	0	9600
CNOT6	-8.17E-04	0	9601
ASIC4	-8.17E-04	0	9602
MRPL32	-8.18E-04	0	9603
APH1B	-8.18E-04	0	9604
LOC1001446	-8.18E-04	0	9605
NAALAD2	-8.18E-04	0	9606
STXBP6	-8.18E-04	0	9607
CHRNA5	-8.18E-04	0	9608
HTRA3	-8.18E-04	0	9609
DCR	-8.18E-04	0	9610
RPRD1A	-8.18E-04	0	9611
GCSAM	-8.18E-04	0	9612
TNNC2	-8.20E-04	0	9613
TNNI1	-8.20E-04	0	9614
PRDX6	-8.20E-04	0	9615
MARK4	-8.21E-04	0	9616
PFKM	-8.22E-04	0	9617
TMBIM4	-8.24E-04	0	9618
KLF3	-8.24E-04	0	9619
YAF2	-8.25E-04	0	9620
CATSPER1	-8.26E-04	0	9621
C19ORF48	-8.27E-04	0	9622
HELZ	-8.27E-04	0	9623
IDO2	-8.29E-04	0	9624
STEAP1	-8.29E-04	0	9625
GGT1	-8.30E-04	0	9626
KDM8	-8.30E-04	0	9627

MIR1180	-8.30E-04	0	9628
MIR639	-8.30E-04	0	9629
ZBTB8A	-8.30E-04	0	9630
WFIKKN1	-8.30E-04	0	9631
NEK6	-8.31E-04	0	9632
TIPIN	-8.32E-04	0	9633
PYDC1	-8.33E-04	0	9634
MIR197	-8.33E-04	0	9635
H2AFY2	-8.34E-04	0	9636
SAP30	-8.36E-04	0	9637
STK17A	-8.36E-04	0	9638
LEPRE1	-8.36E-04	0	9639
P4HTM	-8.36E-04	0	9640
TMEM185A	-8.39E-04	0	9641
COX8A	-8.41E-04	0	9642
LNP	-8.43E-04	0	9643
BARHL2	-8.43E-04	0	9644
MVK	-8.43E-04	0	9645
CELA1	-8.44E-04	0	9646
KRT75	-8.45E-04	0	9647
RASSF8	-8.45E-04	0	9648
SLC2A7	-8.45E-04	0	9649
TBC1D25	-8.46E-04	0	9650
ANKRD46	-8.46E-04	0	9651
SEC23B	-8.46E-04	0	9652
PAPSS1	-8.47E-04	0	9653
RPL13A	-8.47E-04	0	9654
CCBE1	-8.48E-04	0	9655
SLC13A3	-8.49E-04	0	9656
ICR1	-8.49E-04	0	9657
APOBEC2	-8.50E-04	0	9658
ARPC2	-8.50E-04	0	9659
DCPS	-8.53E-04	0	9660
KAL1	-8.53E-04	0	9661
SLC30A1	-8.53E-04	0	9662
ARMC9	-8.54E-04	0	9663
SLC22A9	-8.54E-04	0	9664
TGS1	-8.55E-04	0	9665
HPX	-8.55E-04	0	9666
SLC13A2	-8.55E-04	0	9667
IQGAP2	-8.56E-04	0	9668
SLCO2B1	-8.56E-04	0	9669
STK24	-8.57E-04	0	9670

CDH13	-8.57E-04	0	9671
PAIP2	-8.58E-04	0	9672
MIR1271	-8.59E-04	0	9673
PLEKHO2	-8.61E-04	0	9674
LIMA1	-8.62E-04	0	9675
NIPBL	-8.62E-04	0	9676
KRT72	-8.62E-04	0	9677
NALCN	-8.62E-04	0	9678
GABRB3	-8.63E-04	0	9679
JADE1	-8.65E-04	0	9680
GNAI3	-8.65E-04	0	9681
ERH	-8.65E-04	0	9682
TM7SF3	-8.66E-04	0	9683
CAB39	-8.67E-04	0	9684
PI4K2A	-8.68E-04	0	9685
DAZL	-8.70E-04	0	9686
RHOT1	-8.70E-04	0	9687
CRYAA	-8.70E-04	0	9688
AIFM3	-8.72E-04	0	9689
RP2	-8.72E-04	0	9690
MTRR	-8.75E-04	0	9691
OLFM3	-8.75E-04	0	9692
CHST11	-8.77E-04	0	9693
ID4	-8.77E-04	0	9694
SNORD51	-8.78E-04	0	9695
CDK5RAP3	-8.79E-04	0	9696
KLK14	-8.81E-04	0	9697
PRR5L	-8.81E-04	0	9698
SLC38A9	-8.81E-04	0	9699
GPM6A	-8.81E-04	0	9700
PRKCH	-8.81E-04	0	9701
TOP2B	-8.83E-04	0	9702
GDF7	-8.83E-04	0	9703
BYSL	-8.84E-04	0	9704
ALDH1L1	-8.85E-04	0	9705
CFHR2	-8.87E-04	0	9706
LIMD1	-8.87E-04	0	9707
KCNE1L	-8.87E-04	0	9708
PDIA6	-8.88E-04	0	9709
CASC2	-8.88E-04	0	9710
MRT5	-8.88E-04	0	9711
MT-ND4	-8.89E-04	0	9712
CCDC88C	-8.89E-04	0	9713

LY6G5B	-8.90E-04	0	9714
BCAS2	-8.91E-04	0	9715
GMPR2	-8.91E-04	0	9716
RRP1	-8.91E-04	0	9717
RFX7	-8.91E-04	0	9718
TARBP1	-8.91E-04	0	9719
ZSCAN10	-8.92E-04	0	9720
AMY1B	-8.93E-04	0	9721
HPR	-8.93E-04	0	9722
SPRN	-8.93E-04	0	9723
ADRBK2	-8.93E-04	0	9724
TMPRSS9	-8.94E-04	0	9725
MLX	-8.95E-04	0	9726
NXF2	-8.95E-04	0	9727
C1ORF87	-8.98E-04	0	9728
CELP	-8.98E-04	0	9729
SIX4	-8.99E-04	0	9730
TPK1	-9.00E-04	0	9731
CLCN4	-9.00E-04	0	9732
CYP3A5	-9.01E-04	0	9733
NCAPH2	-9.02E-04	0	9734
KANSL2	-9.03E-04	0	9735
POU5F1P3	-9.03E-04	0	9736
CETN2	-9.03E-04	0	9737
MIR204	-9.03E-04	0	9738
STS	-9.04E-04	0	9739
CPXM2	-9.04E-04	0	9740
GATM	-9.05E-04	0	9741
RABAC1	-9.05E-04	0	9742
CAP1	-9.07E-04	0	9743
ZNRF3	-9.09E-04	0	9744
RPS3A	-9.11E-04	0	9745
MGS	-9.12E-04	0	9746
NPM3	-9.12E-04	0	9747
OPLL	-9.12E-04	0	9748
PCDH11Y	-9.12E-04	0	9749
PSG9	-9.12E-04	0	9750
SNHG16	-9.12E-04	0	9751
STARD9	-9.12E-04	0	9752
DVL3	-9.13E-04	0	9753
GPD1L	-9.13E-04	0	9754
MIR378A	-9.13E-04	0	9755
SSPN	-9.14E-04	0	9756

CNTN5	-9.16E-04	0	9757
CA14	-9.16E-04	0	9758
IGKJ2	-9.16E-04	0	9759
MIR526B	-9.16E-04	0	9760
MARVELD3	-9.16E-04	0	9761
HFM1	-9.18E-04	0	9762
LTV1	-9.19E-04	0	9763
MAN2C1	-9.19E-04	0	9764
FBXO45	-9.20E-04	0	9765
CLOCK	-9.20E-04	0	9766
DMXL1	-9.20E-04	0	9767
LPCAT3	-9.21E-04	0	9768
TREX1	-9.21E-04	0	9769
PPIL2	-9.21E-04	0	9770
TOP3A	-9.21E-04	0	9771
SYF2	-9.22E-04	0	9772
DPH5	-9.22E-04	0	9773
TBX15	-9.23E-04	0	9774
SCARA5	-9.23E-04	0	9775
SESN3	-9.24E-04	0	9776
POU3F4	-9.25E-04	0	9777
CHML	-9.25E-04	0	9778
TMPO	-9.26E-04	0	9779
RMND5A	-9.26E-04	0	9780
SYN2	-9.26E-04	0	9781
ELTD1	-9.30E-04	0	9782
SLC30A5	-9.31E-04	0	9783
DNALI1	-9.31E-04	0	9784
UBE4B	-9.32E-04	0	9785
CELF3	-9.33E-04	0	9786
KRT77	-9.33E-04	0	9787
SLCO1C1	-9.34E-04	0	9788
HIST2H2AC	-9.34E-04	0	9789
ADAMTS12	-9.35E-04	0	9790
ZBTB4	-9.35E-04	0	9791
GTF2IRD1	-9.36E-04	0	9792
SLC47A1	-9.37E-04	0	9793
RB1CC1	-9.38E-04	0	9794
CDC16	-9.40E-04	0	9795
CCNL2	-9.40E-04	0	9796
DNAJC7	-9.41E-04	0	9797
PURB	-9.42E-04	0	9798
TUFT1	-9.43E-04	0	9799

B4GALNT1	-9.45E-04	0	9800
KIAA1279	-9.45E-04	0	9801
AVPI1	-9.46E-04	0	9802
ZNF72P	-9.46E-04	0	9803
WNT8B	-9.47E-04	0	9804
HIST1H3E	-9.48E-04	0	9805
SLC12A3	-9.49E-04	0	9806
DHRS9	-9.50E-04	0	9807
KCNK10	-9.50E-04	0	9808
AIS4	-9.50E-04	0	9809
FAM96B	-9.51E-04	0	9810
CIB2	-9.52E-04	0	9811
CRYGS	-9.54E-04	0	9812
RUVBL2	-9.54E-04	0	9813
MACF1	-9.55E-04	0	9814
MSS51	-9.55E-04	0	9815
GAA	-9.55E-04	0	9816
DDOST	-9.57E-04	0	9817
SYNC	-9.57E-04	0	9818
SLCO1A2	-9.58E-04	0	9819
PLXNB3	-9.58E-04	0	9820
WNT7B	-9.58E-04	0	9821
TAS1R1	-9.59E-04	0	9822
HOXB3	-9.59E-04	0	9823
PCDH17	-9.61E-04	0	9824
FNDC3A	-9.61E-04	0	9825
CCT	-9.62E-04	0	9826
SNORD50A	-9.63E-04	0	9827
RABL6	-9.64E-04	0	9828
HPCAL1	-9.64E-04	0	9829
ACSL5	-9.64E-04	0	9830
KIF17	-9.65E-04	0	9831
SRPK1	-9.66E-04	0	9832
ZDHHC2	-9.66E-04	0	9833
GINS3	-9.66E-04	0	9834
DHPS	-9.67E-04	0	9835
CRS	-9.69E-04	0	9836
FKBP3	-9.69E-04	0	9837
MIR376C	-9.70E-04	0	9838
PPP2R3B	-9.75E-04	0	9839
TNMD	-9.76E-04	0	9840
MYOM1	-9.77E-04	0	9841
SERPINF1	-9.78E-04	0	9842

NTN4	-9.79E-04	0	9843
TBX2	-9.81E-04	0	9844
GNB5	-9.82E-04	0	9845
MAGEA4	-9.82E-04	0	9846
IDH3A	-9.82E-04	0	9847
THAP5	-9.82E-04	0	9848
RPS18	-9.83E-04	0	9849
DONSON	-9.83E-04	0	9850
NIPSNAP1	-9.83E-04	0	9851
FLCN	-9.83E-04	0	9852
SLC31A2	-9.84E-04	0	9853
SV2C	-9.84E-04	0	9854
RPL32	-9.85E-04	0	9855
GNAT2	-9.86E-04	0	9856
TENC1	-9.87E-04	0	9857
MGST1	-9.89E-04	0	9858
PTGER1	-9.89E-04	0	9859
COLEC12	-9.90E-04	0	9860
IFNK	-9.90E-04	0	9861
SAMD3	-9.90E-04	0	9862
DGCR2	-9.90E-04	0	9863
EMILIN1	-9.90E-04	0	9864
P2RY11	-9.93E-04	0	9865
CHD6	-9.93E-04	0	9866
CHD2	-9.94E-04	0	9867
DBX1	-9.94E-04	0	9868
MIR589	-9.96E-04	0	9869
CSTB	-9.96E-04	0	9870
ACTN3	-9.97E-04	0	9871
SMCR7L	-9.98E-04	0	9872
DMBX1	-9.99E-04	0	9873
NUMBL	-1.00E-03	0	9874
APBA2	-0.0010005	0	9875
DNAJC19	-0.0010006	0	9876
QRFPR	-0.0010011	0	9877
POU3F1	-0.0010016	0	9878
XYLB	-0.0010028	0	9879
KLHDC10	-0.0010031	0	9880
CLPB	-0.0010038	0	9881
CDKN2AIP	-0.0010062	0	9882
NUMB	-0.0010062	0	9883
MAN2A1	-0.0010064	0	9884
CHRNA9	-0.0010065	0	9885

SH3TC2	-0.0010072	0	9886
LPAR5	-0.0010076	0	9887
ADCY3	-0.0010083	0	9888
FAM84B	-0.0010115	0	9889
PSAP	-0.0010121	0	9890
HSPB11	-0.0010142	0	9891
CPM	-0.0010146	0	9892
SUV39H1	-0.0010151	0	9893
CLDN10	-0.001016	0	9894
TSR3	-0.0010161	0	9895
LRIG2	-0.0010168	0	9896
MIR454	-0.0010191	0	9897
ANKS1A	-0.0010192	0	9898
ADH1A	-0.0010197	0	9899
VWA2	-0.0010207	0	9900
FGF17	-0.0010215	0	9901
PAEP	-0.0010218	0	9902
CHRNA7	-0.0010222	0	9903
RNASET2	-0.0010226	0	9904
SDHAF2	-0.0010227	0	9905
HEY2	-0.0010238	0	9906
TSIX	-0.0010245	0	9907
RNF123	-0.0010265	0	9908
SLC4A11	-0.0010268	0	9909
NGLY1	-0.001027	0	9910
GGN	-0.0010271	0	9911
CFM1	-0.0010299	0	9912
PPP2R1B	-0.0010301	0	9913
BRCA2	-0.0010335	0	9914
MGAT1	-0.0010335	0	9915
MTRNR2L5	-0.0010336	0	9916
AARD	-0.0010338	0	9917
PCGEM1	-0.0010338	0	9918
SLC39A9	-0.0010338	0	9919
WBSCR28	-0.0010338	0	9920
ZNF438	-0.0010338	0	9921
FOXA2	-0.0010339	0	9922
NGB	-0.0010339	0	9923
MIR766	-0.0010352	0	9924
LILRA1	-0.0010357	0	9925
ALPK3	-0.0010366	0	9926
QDPR	-0.0010379	0	9927
IGFBP5	-0.0010394	0	9928

ECM29	-0.0010394	0	9929
ADH7	-0.0010403	0	9930
ZWINT	-0.0010412	0	9931
SYT12	-0.0010413	0	9932
IGJ	-0.0010423	0	9933
SCAMP1	-0.001043	0	9934
CCDC50	-0.0010437	0	9935
DUS4L	-0.0010437	0	9936
ERVK-5	-0.0010437	0	9937
MVB12B	-0.0010437	0	9938
NTNG2	-0.0010437	0	9939
SH3D19	-0.0010437	0	9940
TRE17	-0.0010437	0	9941
DDR2	-0.001044	0	9942
GRSF1	-0.0010453	0	9943
RPL41	-0.0010455	0	9944
MIR224	-0.0010462	0	9945
BCAP29	-0.0010473	0	9946
C2ORF49	-0.0010482	0	9947
NIDDM2	-0.0010485	0	9948
ABCD3	-0.0010486	0	9949
DPH1	-0.0010496	0	9950
PRR24	-0.0010514	0	9951
OGFR	-0.0010532	0	9952
CPLX1	-0.0010542	0	9953
USH2A	-0.0010544	0	9954
SEMA6C	-0.0010553	0	9955
ODPF	-0.0010557	0	9956
PLAC8	-0.0010563	0	9957
MIR29B1	-0.0010564	0	9958
KCNQ1	-0.001057	0	9959
SLC37A1	-0.0010583	0	9960
MYO1E	-0.001059	0	9961
BFSP1	-0.0010608	0	9962
GUCA1A	-0.0010611	0	9963
FER1L4	-0.0010612	0	9964
PCDH19	-0.0010643	0	9965
TNNT3	-0.001065	0	9966
CLN9	-0.001066	0	9967
CHNG3	-0.0010663	0	9968
SPINK5	-0.0010667	0	9969
BORA	-0.0010674	0	9970
ATP6V0A2	-0.0010675	0	9971

CFHR5	-0.001068	0	9972
LINC00467	-0.0010706	0	9973
SNHG1	-0.0010706	0	9974
FRMD1	-0.0010711	0	9975
MAGEL2	-0.0010711	0	9976
SLC38A4	-0.0010711	0	9977
SCYL1	-0.0010718	0	9978
NMUR1	-0.001074	0	9979
GFRA2	-0.0010743	0	9980
MIR29A	-0.0010749	0	9981
PORCN	-0.0010761	0	9982
PDAP1	-0.0010764	0	9983
ACKR2	-0.0010766	0	9984
CCDC19	-0.001077	0	9985
ADORA1	-0.0010777	0	9986
DYX1C1	-0.0010779	0	9987
CASP8AP2	-0.0010782	0	9988
XRCC4	-0.0010787	0	9989
CYP2E1	-0.0010798	0	9990
KIR2DS3	-0.0010806	0	9991
KLK13	-0.0010813	0	9992
ANKRD1	-0.0010815	0	9993
C5ORF27	-0.0010819	0	9994
MORC2	-0.0010823	0	9995
IGKV2-19	-0.0010841	0	9996
MT1B	-0.0010844	0	9997
PPP2R2D	-0.0010858	0	9998
HOXA11	-0.0010864	0	9999
ABCD2	-0.0010872	0	10000
MIR518B	-0.0010872	0	10001
PHF12	-0.0010888	0	10002
ZNF420	-0.0010889	0	10003
APOLD1	-0.0010892	0	10004
C5ORF38	-0.0010896	0	10005
PRLHR	-0.0010911	0	10006
BEX1	-0.0010916	0	10007
KCNE3	-0.0010939	0	10008
KCNB2	-0.0010939	0	10009
NPTXR	-0.0010945	0	10010
AK5	-0.0010946	0	10011
PROL1	-0.0010968	0	10012
RASAL2	-0.0010978	0	10013
SERTAD2	-0.0010978	0	10014

CAMK2N2	-0.0011	0	10015
HTR1E	-0.0011004	0	10016
MIR944	-0.001101	0	10017
PRLH	-0.0011012	0	10018
KCNJ6	-0.001104	0	10019
PROX1	-0.0011041	0	10020
DDX11	-0.0011048	0	10021
ACP2	-0.0011048	0	10022
PON3	-0.0011049	0	10023
CDK13	-0.0011075	0	10024
PTGES2	-0.0011077	0	10025
ZBTB6	-0.001109	0	10026
MBOAT2	-0.0011095	0	10027
SNX17	-0.0011098	0	10028
IGKV1D-33	-0.0011105	0	10029
NBR2	-0.0011124	0	10030
MIR584	-0.001114	0	10031
CSNK1E	-0.0011154	0	10032
COBRA1	-0.0011169	0	10033
PPM1E	-0.0011192	0	10034
FLII	-0.0011201	0	10035
VIPR2	-0.0011201	0	10036
CBX6	-0.0011208	0	10037
KCNJ9	-0.0011215	0	10038
TAAR1	-0.0011219	0	10039
HIF3A	-0.0011277	0	10040
TPT1	-0.0011278	0	10041
DTNA	-0.0011285	0	10042
PPIL1	-0.0011289	0	10043
SNRPC	-0.0011295	0	10044
DEFB124	-0.0011307	0	10045
MPEG1	-0.0011307	0	10046
LRRC4	-0.001131	0	10047
ZWILCH	-0.0011312	0	10048
ZNF398	-0.0011315	0	10049
TENM1	-0.001133	0	10050
FAM64A	-0.0011338	0	10051
PCOLCE	-0.0011339	0	10052
MIR99A	-0.001134	0	10053
ADRB3	-0.0011341	0	10054
KCNC2	-0.0011357	0	10055
APC2	-0.0011369	0	10056
RND3	-0.0011372	0	10057

APBA3	-0.0011378	0	10058
MSSE	-0.001138	0	10059
TSPAN5	-0.0011386	0	10060
RCN1	-0.0011393	0	10061
AD11	-0.0011432	0	10062
AD16	-0.0011434	0	10063
DBX2	-0.0011452	0	10064
HHC2	-0.0011452	0	10065
NKX1-2	-0.0011452	0	10066
TLE6	-0.0011452	0	10067
FKBP1B	-0.0011455	0	10068
PAPLN	-0.001146	0	10069
MSX2	-0.0011469	0	10070
NUDT16	-0.0011469	0	10071
ALDOA	-0.0011471	0	10072
LGALS7B	-0.0011473	0	10073
GPRC5C	-0.0011482	0	10074
KANK1	-0.0011492	0	10075
FNIP2	-0.0011493	0	10076
CCNO	-0.0011516	0	10077
CTNNA2	-0.0011532	0	10078
MUC7	-0.0011536	0	10079
SLC36A3	-0.0011536	0	10080
ATP1A1	-0.0011549	0	10081
CRTAM	-0.0011551	0	10082
YES1	-0.0011564	0	10083
WNT2B	-0.0011586	0	10084
MACROD2	-0.0011601	0	10085
POLD2	-0.0011602	0	10086
KIAA1217	-0.0011615	0	10087
DHRS4	-0.0011639	0	10088
RNF138	-0.0011641	0	10089
TMEM8B	-0.0011645	0	10090
SIK3	-0.0011673	0	10091
RHEBP1	-0.0011675	0	10092
TSPAN15	-0.0011676	0	10093
HMSD	-0.001168	0	10094
CDH12	-0.0011685	0	10095
KDM4C	-0.001169	0	10096
HELZ2	-0.001169	0	10097
CUEDC2	-0.0011708	0	10098
SESN1	-0.0011708	0	10099
AFG3L2	-0.0011713	0	10100

PIGM	-0.0011714	0	10101
YESP	-0.0011742	0	10102
KMO	-0.0011744	0	10103
HOXD10	-0.0011747	0	10104
UBE4A	-0.001178	0	10105
DLG2	-0.0011793	0	10106
TMPRSS11A	-0.0011802	0	10107
GAS2	-0.0011808	0	10108
GLTP	-0.0011809	0	10109
CRIP1	-0.0011822	0	10110
ARL6IP1	-0.0011824	0	10111
SPPL2B	-0.001184	0	10112
RBBP9	-0.0011841	0	10113
HNRNPR	-0.0011845	0	10114
NAGK	-0.0011874	0	10115
BOC	-0.001192	0	10116
KRT26	-0.0011925	0	10117
LETM1	-0.0011928	0	10118
CTDSP2	-0.001193	0	10119
NRG4	-0.0011945	0	10120
MIR23C	-0.0011955	0	10121
MIR874	-0.0011961	0	10122
ATP2A3	-0.0011965	0	10123
SPSB1	-0.0011971	0	10124
ST11	-0.0011987	0	10125
NKIRAS2	-0.0011997	0	10126
CHE2	-0.0012003	0	10127
LRRC59	-0.0012004	0	10128
SLC12A2	-0.0012008	0	10129
TUBA4A	-0.0012031	0	10130
CNTROB	-0.0012044	0	10131
MMEL1	-0.0012045	0	10132
RS1	-0.0012054	0	10133
MIR933	-0.0012055	0	10134
MAGEA2	-0.001206	0	10135
POLDIP3	-0.0012061	0	10136
RHBDF1	-0.0012063	0	10137
CCL4L2	-0.0012077	0	10138
SNORD83A	-0.0012095	0	10139
MT3	-0.00121	0	10140
ND4	-0.0012111	0	10141
GNAT3	-0.0012122	0	10142
FCGBP	-0.0012135	0	10143

GH2	-0.001214	0	10144
CPEB3	-0.0012142	0	10145
CNOT2	-0.0012147	0	10146
VPS29	-0.0012175	0	10147
PRMT7	-0.0012183	0	10148
HMOX2	-0.0012185	0	10149
ACAP1	-0.0012208	0	10150
OED	-0.001221	0	10151
LRWD1	-0.0012213	0	10152
APOL2	-0.0012213	0	10153
SNORD1A	-0.0012213	0	10154
TOR1AIP2	-0.0012214	0	10155
MIR200C	-0.0012224	0	10156
ANAPC5	-0.0012229	0	10157
PAFAH1B1	-0.0012235	0	10158
PYGM	-0.0012236	0	10159
AHCTF1	-0.0012238	0	10160
AMCN	-0.0012238	0	10161
TSPYL5	-0.0012241	0	10162
EIF4H	-0.0012265	0	10163
RND2	-0.0012266	0	10164
SPSB2	-0.0012279	0	10165
IWS1	-0.0012289	0	10166
CRISP3	-0.0012301	0	10167
BANP	-0.0012319	0	10168
SORCS1	-0.0012324	0	10169
POLR1D	-0.0012335	0	10170
TTPA	-0.0012342	0	10171
ANKRD13A	-0.0012348	0	10172
OVOL2	-0.0012356	0	10173
RHEBL1	-0.0012373	0	10174
MIR423	-0.0012378	0	10175
NDC80	-0.0012386	0	10176
DLX5	-0.0012412	0	10177
TNFAIP2	-0.0012442	0	10178
AFAP1L1	-0.0012442	0	10179
RNF14	-0.0012479	0	10180
EXOC7	-0.0012492	0	10181
COL9A2	-0.00125	0	10182
MIR29B2	-0.001251	0	10183
PRPS2	-0.001252	0	10184
ADAM3A	-0.0012538	0	10185
DCLK2	-0.0012542	0	10186

CLCN6	-0.0012549	0	10187
ZNRF2	-0.0012551	0	10188
DFFB	-0.0012552	0	10189
CYSLTR1	-0.0012559	0	10190
JAWAD	-0.0012562	0	10191
KCNE2	-0.0012569	0	10192
HPDL	-0.0012572	0	10193
WFIKKN2	-0.0012584	0	10194
UTP6	-0.0012591	0	10195
SPARC	-0.0012613	0	10196
CLDN19	-0.001262	0	10197
MIR128-1	-0.0012628	0	10198
OSBPL8	-0.001263	0	10199
GDEP	-0.0012643	0	10200
MT1IP	-0.0012654	0	10201
HIST1H1E	-0.0012674	0	10202
EPM2A	-0.0012714	0	10203
ITGB8	-0.0012719	0	10204
SLC27A5	-0.001272	0	10205
TFPI	-0.0012729	0	10206
CCDC155	-0.0012731	0	10207
USP45	-0.0012734	0	10208
MAGEA1	-0.0012736	0	10209
8-Sep	-0.0012742	0	10210
TRIM24	-0.0012746	0	10211
H2BFM	-0.0012748	0	10212
EPYC	-0.0012756	0	10213
RMRP	-0.0012768	0	10214
TRIM11	-0.0012782	0	10215
MIR371A	-0.0012784	0	10216
HCN4	-0.0012799	0	10217
PRMT2	-0.0012805	0	10218
PEX5L	-0.0012841	0	10219
FZD3	-0.0012863	0	10220
SARM1	-0.0012871	0	10221
CLCA1	-0.0012892	0	10222
NAT8	-0.0012894	0	10223
PHPT1	-0.0012894	0	10224
MCHR2	-0.0012897	0	10225
NOB1	-0.00129	0	10226
HOXD4	-0.0012919	0	10227
GK	-0.001292	0	10228
LAMA2	-0.0012924	0	10229

CNTNAP2	-0.0012932	0	10230
HOXD9	-0.0012933	0	10231
MIR143	-0.0012945	0	10232
SYNGAP1	-0.001296	0	10233
RBM23	-0.0012966	0	10234
FBXW11	-0.0013042	0	10235
ITGA7	-0.0013048	0	10236
HOXD8	-0.0013049	0	10237
ORM1	-0.0013059	0	10238
LUC7L3	-0.001306	0	10239
PGLYRP1	-0.0013067	0	10240
PA2G4	-0.0013069	0	10241
HELQ	-0.001308	0	10242
RPL19	-0.001309	0	10243
KATNAL1	-0.0013118	0	10244
NPFF	-0.0013129	0	10245
MIR106A	-0.001313	0	10246
FGF22	-0.0013138	0	10247
ROBO3	-0.0013141	0	10248
PHACTR3	-0.0013143	0	10249
PPP1R9A	-0.0013143	0	10250
PEX7	-0.0013145	0	10251
MCOLN2	-0.001315	0	10252
UPF2	-0.0013152	0	10253
ITPKB	-0.0013179	0	10254
DPYSL5	-0.0013184	0	10255
CLP1	-0.0013187	0	10256
SPTLC1	-0.0013196	0	10257
IGKV2D-40	-0.0013201	0	10258
EXOSC7	-0.0013204	0	10259
HIST3H3	-0.0013205	0	10260
PTBP3	-0.0013206	0	10261
RIC3	-0.0013219	0	10262
GJC3	-0.0013221	0	10263
SCEL	-0.0013222	0	10264
GINS2	-0.0013225	0	10265
TLX1NB	-0.0013241	0	10266
VASH1	-0.0013244	0	10267
ZNF703	-0.0013245	0	10268
UBE2V2	-0.0013248	0	10269
LOXL3	-0.001326	0	10270
UXT	-0.0013266	0	10271
MT-TQ	-0.0013266	0	10272

XRS	-0.0013299	0	10273
STMN2	-0.0013318	0	10274
SMG5	-0.0013347	0	10275
SSX2IP	-0.001335	0	10276
DNMT3B	-0.0013363	0	10277
CTNNBL1	-0.001337	0	10278
FOLH1B	-0.0013383	0	10279
CFI	-0.0013391	0	10280
LIMK1	-0.0013394	0	10281
NOTCH2NL	-0.0013399	0	10282
SUCLA2	-0.0013399	0	10283
TNNI2	-0.0013401	0	10284
TRGJ2	-0.0013404	0	10285
FAM84A	-0.0013404	0	10286
HSD17B2	-0.0013406	0	10287
PHF21A	-0.0013413	0	10288
HSPA2	-0.0013421	0	10289
TAS2R12P	-0.001343	0	10290
WDR63	-0.001343	0	10291
UNC50	-0.0013433	0	10292
MIR449B	-0.0013446	0	10293
ORC5	-0.0013447	0	10294
DMBT1	-0.0013477	0	10295
NARS	-0.0013487	0	10296
FMNL2	-0.0013489	0	10297
MMS19	-0.0013526	0	10298
VUR	-0.0013533	0	10299
AAA3	-0.0013549	0	10300
TMPRSS3	-0.0013564	0	10301
SPC25	-0.0013631	0	10302
UTP20	-0.001364	0	10303
TUBA1A	-0.0013648	0	10304
VPS72	-0.0013649	0	10305
ATPIF1	-0.0013657	0	10306
CROCC	-0.0013687	0	10307
NPAS2	-0.0013691	0	10308
SULF1	-0.0013693	0	10309
MEI4	-0.0013694	0	10310
CDH9	-0.0013694	0	10311
LOXL4	-0.0013728	0	10312
SUOX	-0.0013732	0	10313
TMSB4X	-0.0013745	0	10314
PRDM5	-0.001375	0	10315

TRIM16	-0.0013769	0	10316
IP6K2	-0.0013774	0	10317
TAPBP	-0.0013784	0	10318
MIR34A	-0.0013817	0	10319
AKR1E2	-0.0013819	0	10320
BTF3	-0.0013825	0	10321
DENND4A	-0.001383	0	10322
RSC1A1	-0.0013857	0	10323
HDGFRP2	-0.0013864	0	10324
MIR4262	-0.0013874	0	10325
ASG	-0.0013877	0	10326
ZIC2	-0.0013901	0	10327
MEN1	-0.0013911	0	10328
SP6	-0.0013958	0	10329
MAGEB2	-0.0013965	0	10330
ZNF131	-0.0013969	0	10331
KCNJ16	-0.0013976	0	10332
SOX3	-0.0014007	0	10333
SCA31	-0.0014027	0	10334
CCDC8	-0.0014035	0	10335
SMOX	-0.0014066	0	10336
CST4	-0.0014074	0	10337
FAM168A	-0.0014099	0	10338
BLVRA	-0.0014107	0	10339
RNF126	-0.0014117	0	10340
ATG2B	-0.0014134	0	10341
CHSY1	-0.0014145	0	10342
GCA	-0.0014159	0	10343
PARPBP	-0.0014208	0	10344
BICD2	-0.0014209	0	10345
GPBAR1	-0.001421	0	10346
SNX15	-0.0014227	0	10347
HAUS8	-0.001424	0	10348
GALNS	-0.0014245	0	10349
AZF1	-0.0014252	0	10350
IDI2-AS1	-0.0014259	0	10351
SNHG15	-0.0014259	0	10352
VWA1	-0.0014271	0	10353
TIMM13	-0.0014276	0	10354
TAF9B	-0.0014288	0	10355
ACRV1	-0.0014305	0	10356
SPPL2A	-0.0014306	0	10357
LCN8	-0.0014327	0	10358

HSPE1	-0.0014362	0	10359
SNORD96A	-0.0014375	0	10360
PPIP5K2	-0.0014382	0	10361
SNF8	-0.0014396	0	10362
COL5A2	-0.0014402	0	10363
SNORD62A	-0.0014402	0	10364
CALML3	-0.0014412	0	10365
OPRD1	-0.0014421	0	10366
SLC8A2	-0.0014432	0	10367
IGIP	-0.0014432	0	10368
EYA1	-0.0014437	0	10369
SMARCA5	-0.0014462	0	10370
GPX2	-0.0014474	0	10371
PROCA1	-0.0014499	0	10372
EMG1	-0.0014501	0	10373
MIR181B1	-0.0014502	0	10374
HIST2H4A	-0.0014516	0	10375
ANXA3	-0.0014529	0	10376
SBF2	-0.0014529	0	10377
COQ7	-0.0014539	0	10378
MIEN1	-0.001454	0	10379
MIR942	-0.0014544	0	10380
PSPH	-0.0014554	0	10381
GJC2	-0.0014563	0	10382
LINC00673	-0.0014567	0	10383
ST7L	-0.0014583	0	10384
CEP57	-0.0014586	0	10385
C6ORF89	-0.0014634	0	10386
ZNF367	-0.0014638	0	10387
PRPF8	-0.0014649	0	10388
AGO4	-0.0014649	0	10389
CDIPT	-0.0014651	0	10390
MYH1	-0.0014656	0	10391
IFI27L1	-0.0014662	0	10392
IFI27L2	-0.0014662	0	10393
SLC4A7	-0.0014662	0	10394
FLRT3	-0.0014665	0	10395
MYO1C	-0.0014666	0	10396
OR1L1	-0.0014684	0	10397
NID2	-0.001471	0	10398
POU3F3	-0.0014711	0	10399
CACNA1H	-0.0014723	0	10400
HEMC	-0.0014728	0	10401

PDCL2	-0.0014731	0	10402
NEDD1	-0.0014757	0	10403
VWCE	-0.0014766	0	10404
SPG25	-0.001477	0	10405
MATN3	-0.0014803	0	10406
SLC31A1	-0.001481	0	10407
SYNE1	-0.0014816	0	10408
ECHS1	-0.0014816	0	10409
SURF1	-0.0014847	0	10410
PPP1CC	-0.0014899	0	10411
PCYT1A	-0.0014907	0	10412
RNF111	-0.0014911	0	10413
EGFLAM	-0.0014934	0	10414
NPHP3	-0.001494	0	10415
GPSM1	-0.0014946	0	10416
MASP2	-0.0014955	0	10417
SLC9A2	-0.0014957	0	10418
PARM1	-0.0014967	0	10419
EPT1	-0.0014976	0	10420
SEC24B	-0.001498	0	10421
TAAR9	-0.0014988	0	10422
ITM2A	-0.0015005	0	10423
NUP62CL	-0.001506	0	10424
STAC3	-0.0015082	0	10425
SNAPIN	-0.0015102	0	10426
HCG21	-0.0015104	0	10427
NCAPG	-0.0015104	0	10428
CA13	-0.001511	0	10429
HLA-S	-0.0015146	0	10430
P2RY2	-0.0015165	0	10431
CTDSPL	-0.0015167	0	10432
DNMT3L	-0.0015177	0	10433
RPLP0	-0.0015182	0	10434
GUCA2A	-0.0015183	0	10435
HSF4	-0.0015189	0	10436
SLC25A12	-0.0015225	0	10437
KRT86	-0.0015243	0	10438
B3GALNT1	-0.0015246	0	10439
MAP2	-0.0015248	0	10440
MTHFSD	-0.0015263	0	10441
HNRNPA3	-0.0015265	0	10442
ALMS1	-0.0015282	0	10443
ELMO3	-0.0015282	0	10444

HSD17B11	-0.0015282	0	10445
KLF16	-0.0015282	0	10446
PADI6	-0.0015282	0	10447
ZNF32	-0.0015282	0	10448
NPC2	-0.0015291	0	10449
RAB22A	-0.00153	0	10450
LHX2	-0.0015317	0	10451
ZNF318	-0.0015319	0	10452
NCKAP5	-0.0015329	0	10453
MIR616	-0.0015343	0	10454
PFAS	-0.0015358	0	10455
PCA3	-0.0015358	0	10456
CCNJ	-0.0015361	0	10457
TSN	-0.001537	0	10458
GLE1	-0.0015375	0	10459
SOX2-OT	-0.0015378	0	10460
DEPTOR	-0.0015383	0	10461
CEP164	-0.0015388	0	10462
MTA1	-0.0015394	0	10463
ZNF395	-0.0015395	0	10464
DPPA3	-0.0015401	0	10465
GDF10	-0.0015422	0	10466
MKLN1	-0.0015423	0	10467
MAP3K19	-0.0015424	0	10468
CSPG5	-0.0015429	0	10469
MMP20	-0.0015435	0	10470
SURF2	-0.0015443	0	10471
PFN1	-0.001545	0	10472
TAS2R18P	-0.0015452	0	10473
TFB2M	-0.0015455	0	10474
SPAG11B	-0.0015457	0	10475
BASP1	-0.0015463	0	10476
NEUROD4	-0.0015474	0	10477
CLEC3A	-0.0015475	0	10478
BHMT	-0.001549	0	10479
PSMB5	-0.0015499	0	10480
RRH	-0.0015508	0	10481
RPL23	-0.001551	0	10482
PHLDA3	-0.0015513	0	10483
PCBP2	-0.001552	0	10484
SSTR2	-0.0015526	0	10485
MIR503	-0.0015534	0	10486
FNIP1	-0.0015589	0	10487

TEX101	-0.0015589	0	10488
RIC8B	-0.0015597	0	10489
DAGLA	-0.0015605	0	10490
CTD	-0.0015612	0	10491
TNRC6C	-0.0015615	0	10492
HYLS1	-0.001562	0	10493
HS2ST1	-0.0015634	0	10494
ONECUT2	-0.0015659	0	10495
RGS17	-0.0015693	0	10496
DRG2	-0.0015694	0	10497
FABP1	-0.0015702	0	10498
CNST	-0.0015728	0	10499
CARD18	-0.0015742	0	10500
PLXNC1	-0.0015743	0	10501
HNRNPA0	-0.0015744	0	10502
VTRNA1-2	-0.0015751	0	10503
MT1E	-0.0015753	0	10504
POLD1	-0.001576	0	10505
IL13RA2	-0.0015766	0	10506
MTX2	-0.0015779	0	10507
BCAP31	-0.0015803	0	10508
PKNOX1	-0.001582	0	10509
AMIGO2	-0.0015828	0	10510
KIF4A	-0.0015834	0	10511
SUV39H2	-0.0015855	0	10512
DMXL2	-0.0015863	0	10513
RIMS1	-0.0015897	0	10514
MBD6	-0.0015911	0	10515
HADHB	-0.0015932	0	10516
GARS	-0.0015932	0	10517
NAT8L	-0.0015943	0	10518
MIR296	-0.0015943	0	10519
UQCRC1	-0.0015948	0	10520
ITGB6	-0.001596	0	10521
PTER	-0.0016011	0	10522
SALL4	-0.0016019	0	10523
TOPORS	-0.0016024	0	10524
DYNLL1	-0.0016051	0	10525
MTF2	-0.0016076	0	10526
ERAS	-0.0016093	0	10527
GDPD5	-0.0016095	0	10528
CALHM1	-0.0016097	0	10529
COQ4	-0.0016106	0	10530

TRIT1	-0.0016118	0	10531
BW4	-0.0016119	0	10532
MTHFD2	-0.001615	0	10533
INO80	-0.0016153	0	10534
ANO2	-0.0016166	0	10535
ARG1	-0.0016198	0	10536
EPG5	-0.0016222	0	10537
PANK2	-0.0016224	0	10538
ZFAT	-0.0016224	0	10539
CHAF1B	-0.0016261	0	10540
FBXO4	-0.0016269	0	10541
GRM1	-0.0016278	0	10542
SETD8	-0.0016313	0	10543
S100A2	-0.001632	0	10544
PPAT	-0.0016376	0	10545
LAMC1	-0.0016393	0	10546
SLC28A3	-0.0016398	0	10547
BABAM1	-0.0016437	0	10548
PEF1	-0.0016452	0	10549
DDT	-0.0016453	0	10550
HSD17B1	-0.0016457	0	10551
SYTL3	-0.0016474	0	10552
MIR124-2	-0.0016484	0	10553
HMGN1	-0.0016492	0	10554
MLEC	-0.0016502	0	10555
TNNT1	-0.0016514	0	10556
NFYC	-0.0016519	0	10557
IGKC	-0.0016522	0	10558
PLCE1	-0.0016524	0	10559
ZNF335	-0.0016529	0	10560
KCNK5	-0.0016536	0	10561
SEC14L2	-0.0016536	0	10562
ENO1	-0.0016542	0	10563
SPZ1	-0.0016547	0	10564
SCN3A	-0.0016548	0	10565
NPVF	-0.0016555	0	10566
SRFBP1	-0.0016584	0	10567
NARFL	-0.0016596	0	10568
DPAGT1	-0.0016608	0	10569
AAMP	-0.0016611	0	10570
FAM18B1	-0.0016611	0	10571
MIR762	-0.0016611	0	10572
PCDH12	-0.0016611	0	10573

ZNF124	-0.0016611	0	10574
SACM1L	-0.0016614	0	10575
WDR1	-0.001662	0	10576
LARP1	-0.0016634	0	10577
SELENBP1	-0.0016646	0	10578
PIP5K1A	-0.0016698	0	10579
RP23	-0.0016708	0	10580
TTL	-0.0016713	0	10581
SNORA22	-0.0016729	0	10582
PROA	-0.0016774	0	10583
PSENEN	-0.0016778	0	10584
AMOT	-0.0016783	0	10585
AP2B1	-0.0016798	0	10586
PGK1P1	-0.0016806	0	10587
ARID5A	-0.0016817	0	10588
IP6K1	-0.0016828	0	10589
KCNK3	-0.0016833	0	10590
SGOL2	-0.0016833	0	10591
NUPR1	-0.0016838	0	10592
SLC7A6	-0.001684	0	10593
PUM1	-0.0016853	0	10594
RHBDL2	-0.0016857	0	10595
AQP6	-0.0016858	0	10596
CTSE	-0.0016858	0	10597
DYNAP	-0.0016871	0	10598
MYL2	-0.0016878	0	10599
PADI1	-0.0016907	0	10600
ESCO2	-0.0016924	0	10601
PCNT	-0.0016932	0	10602
PDE5A	-0.0016939	0	10603
SLC25A6	-0.0016947	0	10604
CLPTM1L	-0.0016948	0	10605
MIR187	-0.0016964	0	10606
SAA4	-0.0016991	0	10607
GPR3	-0.0017004	0	10608
GIN1	-0.0017004	0	10609
FHL1	-0.0017009	0	10610
HOXA3	-0.0017011	0	10611
UBR3	-0.0017015	0	10612
PSMA2	-0.0017031	0	10613
FANCF	-0.0017073	0	10614
FAH	-0.0017079	0	10615
LRFN5	-0.0017083	0	10616

GDI1	-0.0017097	0	10617
ALKBH1	-0.0017116	0	10618
SPINT2	-0.0017133	0	10619
CASZ1	-0.0017158	0	10620
PER3	-0.0017168	0	10621
KCTD7	-0.0017176	0	10622
MFSD8	-0.0017176	0	10623
GPC5	-0.0017221	0	10624
NUP37	-0.0017225	0	10625
S100A13	-0.0017234	0	10626
TMEM115	-0.0017246	0	10627
PI16	-0.0017248	0	10628
CETN3	-0.0017255	0	10629
LDHC	-0.0017277	0	10630
NFATC2IP	-0.0017282	0	10631
RPS5	-0.0017288	0	10632
MACC1	-0.0017296	0	10633
MYLPF	-0.0017312	0	10634
RAB5C	-0.0017314	0	10635
SLC25A13	-0.0017314	0	10636
RNU11	-0.0017322	0	10637
JAZF1	-0.0017332	0	10638
KIF14	-0.0017342	0	10639
AGL	-0.0017371	0	10640
DDHD2	-0.0017372	0	10641
SLC7A3	-0.001738	0	10642
SMC2	-0.0017384	0	10643
TSNAX	-0.0017413	0	10644
NTM	-0.0017418	0	10645
HOXA1	-0.001746	0	10646
LNX2	-0.0017491	0	10647
UCA1	-0.0017516	0	10648
ATP6V0B	-0.0017531	0	10649
QARS	-0.0017534	0	10650
ICA1	-0.0017537	0	10651
CRCL	-0.0017543	0	10652
PIANP	-0.0017546	0	10653
UQCRB	-0.0017581	0	10654
USF2	-0.0017594	0	10655
PMS2P3	-0.0017596	0	10656
CHRNA3	-0.0017631	0	10657
C19ORF12	-0.001764	0	10658
PRSS2	-0.0017642	0	10659

CRIP2	-0.0017647	0	10660
SMG1	-0.0017647	0	10661
TRNAN2	-0.0017647	0	10662
THEM4	-0.0017679	0	10663
GLIS1	-0.0017703	0	10664
PCDHB1	-0.0017703	0	10665
TKT	-0.0017737	0	10666
HENMT1	-0.0017742	0	10667
KIF22	-0.0017751	0	10668
PTX3	-0.0017757	0	10669
KLK2	-0.0017824	0	10670
ELAVL2	-0.0017839	0	10671
SSX1	-0.0017854	0	10672
ALPK2	-0.0017862	0	10673
MIRLET7E	-0.0017874	0	10674
CD46P1	-0.0017883	0	10675
SNX3	-0.0017894	0	10676
MIR3188	-0.00179	0	10677
GLULP4	-0.0017903	0	10678
TUT1	-0.0017907	0	10679
NNMT	-0.0017911	0	10680
C1QTNF5	-0.0017926	0	10681
SDHA	-0.001793	0	10682
IKBKAP	-0.0017934	0	10683
TOMM40	-0.0017958	0	10684
CUZD1	-0.0017978	0	10685
MCMBP	-0.0017985	0	10686
SERPINA2	-0.0017986	0	10687
MYL3	-0.0017997	0	10688
BBS2	-0.0018017	0	10689
RPA3	-0.0018036	0	10690
HDLBP	-0.0018069	0	10691
SYT2	-0.0018075	0	10692
GCH1	-0.0018116	0	10693
ARNT2	-0.0018121	0	10694
TEX14	-0.0018131	0	10695
DPP8	-0.001814	0	10696
RCAN2	-0.0018162	0	10697
SUMF2	-0.0018172	0	10698
ZNF267	-0.0018184	0	10699
PITPNM1	-0.00182	0	10700
HTR3B	-0.0018212	0	10701
MYCL	-0.0018227	0	10702

ARPP19	-0.0018243	0	10703
ASB15	-0.0018253	0	10704
BP25	-0.0018253	0	10705
CORD4	-0.0018253	0	10706
LRMP	-0.0018274	0	10707
KCNA6	-0.0018304	0	10708
HSPA9	-0.0018331	0	10709
WT2	-0.0018337	0	10710
DLG3	-0.0018356	0	10711
MIR363	-0.0018376	0	10712
DTYMK	-0.0018379	0	10713
USP14	-0.0018392	0	10714
SNORA69	-0.0018403	0	10715
GNA13	-0.001841	0	10716
PTH2	-0.0018429	0	10717
ADH1B	-0.0018433	0	10718
OTOR	-0.0018438	0	10719
FOXJ3	-0.0018452	0	10720
WDR83	-0.0018469	0	10721
ATP5E	-0.0018496	0	10722
SNCB	-0.0018505	0	10723
EIF2A	-0.001853	0	10724
PAGE5	-0.001856	0	10725
RSS	-0.001856	0	10726
BRSK1	-0.0018575	0	10727
NETO2	-0.0018579	0	10728
SEMA3G	-0.0018586	0	10729
ERI1	-0.001862	0	10730
HES4	-0.001862	0	10731
OPTC	-0.0018634	0	10732
LIPE	-0.0018653	0	10733
BTG1	-0.0018664	0	10734
PTPRG	-0.0018675	0	10735
CYB5R3	-0.0018676	0	10736
ITIH1	-0.0018697	0	10737
PLAA	-0.0018702	0	10738
ST8SIA1	-0.0018715	0	10739
BRDT	-0.0018729	0	10740
MED4	-0.0018747	0	10741
ATP2C1	-0.0018757	0	10742
IZUMO1	-0.0018758	0	10743
CPSF3L	-0.0018795	0	10744
TMEM131	-0.0018795	0	10745

CYBRD1	-0.0018796	0	10746
ADIPOR2	-0.0018796	0	10747
CST8	-0.0018813	0	10748
MAPKAP1	-0.0018821	0	10749
CMKLR1	-0.0018821	0	10750
CLN6	-0.0018824	0	10751
TTC1	-0.001883	0	10752
MED8	-0.0018832	0	10753
KRT25	-0.001884	0	10754
P2RY12	-0.0018852	0	10755
IGKV1-5	-0.0018854	0	10756
ARR3	-0.0018856	0	10757
CCL15	-0.0018897	0	10758
MAGEH1	-0.0018903	0	10759
CHAT	-0.0018907	0	10760
TLK1	-0.0018923	0	10761
LYNX1	-0.0018927	0	10762
REEP5	-0.0018929	0	10763
USP33	-0.0018935	0	10764
CDC23	-0.0018957	0	10765
ESRP2	-0.0018979	0	10766
PRKCSH	-0.0018986	0	10767
SNX27	-0.0018991	0	10768
P2RY4	-0.0018994	0	10769
TCAP	-0.0019014	0	10770
GPR88	-0.0019015	0	10771
SLC39A4	-0.0019035	0	10772
MSGN1	-0.0019042	0	10773
FUT9	-0.0019044	0	10774
LMNB2	-0.0019083	0	10775
INSM1	-0.0019125	0	10776
PDS5A	-0.0019138	0	10777
TNP1	-0.0019157	0	10778
FAM57A	-0.0019162	0	10779
PFDN5	-0.0019175	0	10780
DENND1A	-0.0019178	0	10781
DRAXIN	-0.0019178	0	10782
ECSCR	-0.0019178	0	10783
HEATR1	-0.0019178	0	10784
HOXD-AS1	-0.0019178	0	10785
MIR587	-0.0019178	0	10786
MIR618	-0.0019178	0	10787
OXER1	-0.0019178	0	10788

RALGAPA1	-0.0019178	0	10789
SMARCD2	-0.0019178	0	10790
RBM24	-0.0019189	0	10791
BMP1	-0.0019192	0	10792
CRIM1	-0.0019212	0	10793
HINT2	-0.0019228	0	10794
ZBTB12	-0.0019234	0	10795
USP2	-0.0019245	0	10796
IGFBP1	-0.0019247	0	10797
ARIH2	-0.0019272	0	10798
PDHB	-0.0019285	0	10799
PARK3	-0.0019293	0	10800
ELAC1	-0.0019308	0	10801
TMEM11	-0.0019309	0	10802
OXCT1	-0.0019319	0	10803
TXNRD2	-0.001936	0	10804
MIR361	-0.0019366	0	10805
MLNR	-0.0019402	0	10806
ART3	-0.0019418	0	10807
ALLC	-0.0019427	0	10808
MUC5B	-0.0019467	0	10809
MADD	-0.0019482	0	10810
JPX	-0.0019506	0	10811
SFN	-0.001952	0	10812
SDSL	-0.0019529	0	10813
PCDH11X	-0.0019533	0	10814
TIMM23	-0.001954	0	10815
SCMH1	-0.0019555	0	10816
CYB561D2	-0.0019563	0	10817
SMC4	-0.0019567	0	10818
LIPF	-0.0019571	0	10819
NRXN3	-0.0019573	0	10820
LAMA3	-0.0019579	0	10821
CEP131	-0.0019581	0	10822
CARHSP1	-0.001959	0	10823
CARD8	-0.0019612	0	10824
STX6	-0.001965	0	10825
REEP6	-0.001965	0	10826
ATXN8OS	-0.0019674	0	10827
PEX14	-0.0019682	0	10828
NEUROD6	-0.001969	0	10829
PPP1R12B	-0.0019717	0	10830
PANX2	-0.0019726	0	10831

KCNC1	-0.0019752	0	10832
NUDT6	-0.0019756	0	10833
ZFP161	-0.0019786	0	10834
NUP85	-0.001979	0	10835
SBNO2	-0.0019828	0	10836
RAB11FIP5	-0.0019834	0	10837
KCNQ3	-0.0019843	0	10838
SLC6A5	-0.001985	0	10839
ACRC	-0.0019902	0	10840
SIX3	-0.0019903	0	10841
LYZL2	-0.0019909	0	10842
PHF8	-0.0019911	0	10843
SLC52A1	-0.0019931	0	10844
SLC1A1	-0.0019931	0	10845
SPDYA	-0.0019949	0	10846
ZMYM3	-0.0019951	0	10847
MSE	-0.0019956	0	10848
ASIC2	-0.0019958	0	10849
C12ORF39	-0.0019974	0	10850
SHOC2	-0.0019983	0	10851
LIAS	-0.0020002	0	10852
MPI	-0.0020018	0	10853
EAPP	-0.0020018	0	10854
PEX13	-0.0020032	0	10855
GKN2	-0.0020034	0	10856
RBMY1A1	-0.0020042	0	10857
SCN5A	-0.0020042	0	10858
BP4	-0.0020047	0	10859
MNT	-0.0020071	0	10860
SCNN1B	-0.0020145	0	10861
CHRM1	-0.002016	0	10862
MTRNR2L12	-0.0020162	0	10863
GPR132	-0.0020168	0	10864
SNRPD3	-0.0020184	0	10865
SLC28A1	-0.0020188	0	10866
LUM	-0.0020204	0	10867
SNRPN	-0.0020211	0	10868
GDF6	-0.0020219	0	10869
PGAM2	-0.0020225	0	10870
CHPT1	-0.0020225	0	10871
MAP3K12	-0.0020229	0	10872
BOLL	-0.0020239	0	10873
AD6	-0.002024	0	10874

PGM1	-0.0020242	0	10875
SCGB2B3P	-0.0020245	0	10876
SERPIND1	-0.0020247	0	10877
CAPN6	-0.0020255	0	10878
BAI2	-0.0020264	0	10879
STRAP	-0.0020285	0	10880
LMO4	-0.0020316	0	10881
MRAP2	-0.0020321	0	10882
REEP2	-0.0020321	0	10883
TEP1	-0.0020334	0	10884
ERBB2IP	-0.0020343	0	10885
MDH1	-0.0020347	0	10886
CCNB1IP1	-0.0020351	0	10887
HSPA12B	-0.0020359	0	10888
ALDH1B1	-0.0020413	0	10889
KRT36	-0.0020415	0	10890
FBP2	-0.0020452	0	10891
HM13	-0.0020457	0	10892
TMEM18	-0.0020502	0	10893
CSMD1	-0.0020511	0	10894
DEXI	-0.0020548	0	10895
JMY	-0.0020568	0	10896
BP6	-0.0020615	0	10897
PGPEP1	-0.0020628	0	10898
TRHDE	-0.0020628	0	10899
SFRP5	-0.0020631	0	10900
ATP1A2	-0.0020632	0	10901
GAS2L1	-0.0020636	0	10902
GLT8D1	-0.0020638	0	10903
CARF	-0.0020655	0	10904
C19ORF80	-0.0020705	0	10905
NNT	-0.0020714	0	10906
IGKV1-16	-0.0020717	0	10907
MHB	-0.0020722	0	10908
AIF1L	-0.0020775	0	10909
CP20	-0.0020785	0	10910
CAPN1	-0.0020796	0	10911
XIST	-0.0020808	0	10912
ANKH	-0.0020818	0	10913
SMARCC2	-0.0020825	0	10914
TMEM43	-0.0020827	0	10915
SNORD32A	-0.0020835	0	10916
MLRG	-0.0020851	0	10917

THBS4	-0.0020888	0	10918
MIR1915	-0.0020892	0	10919
TRIP12	-0.0020906	0	10920
ACT	-0.0020912	0	10921
OMD	-0.0020915	0	10922
RASL12	-0.0020969	0	10923
USH1C	-0.0020976	0	10924
LARS2	-0.0020988	0	10925
TUBGCP6	-0.0021005	0	10926
PNLIPRP2	-0.0021022	0	10927
CCNDBP1	-0.0021068	0	10928
MIR383	-0.002107	0	10929
FEV	-0.0021079	0	10930
ADAM19	-0.0021092	0	10931
TTF2	-0.0021104	0	10932
CNRIP1	-0.0021105	0	10933
SLC30A6	-0.0021158	0	10934
RPP14	-0.0021163	0	10935
PTCHD3	-0.0021173	0	10936
SIX2	-0.0021181	0	10937
TMUB1	-0.0021218	0	10938
CNR2	-0.002123	0	10939
MYO3A	-0.0021278	0	10940
GCNT2	-0.0021296	0	10941
DND1	-0.0021301	0	10942
NMNAT1	-0.0021313	0	10943
PATZ1	-0.0021321	0	10944
MAGI2	-0.0021324	0	10945
ASPM	-0.0021336	0	10946
OAF	-0.0021348	0	10947
TIAL1	-0.0021409	0	10948
SMARCD3	-0.002142	0	10949
BMPER	-0.0021435	0	10950
DNAJB9	-0.0021437	0	10951
CNDP1	-0.002144	0	10952
AP1S1	-0.0021447	0	10953
CHRFAM7A	-0.0021451	0	10954
CST1	-0.0021462	0	10955
GCHFR	-0.0021512	0	10956
MIR17HG	-0.0021514	0	10957
SLC3A1	-0.0021519	0	10958
SCARA3	-0.0021555	0	10959
MRPL9	-0.0021584	0	10960

NKD1	-0.0021593	0	10961
PLIN5	-0.0021603	0	10962
ARL2	-0.0021621	0	10963
ANKRD23	-0.0021622	0	10964
RFX5	-0.0021628	0	10965
KRT9	-0.0021635	0	10966
ECE2	-0.0021648	0	10967
BCKDHA	-0.0021654	0	10968
SLC38A3	-0.0021658	0	10969
RCVRN	-0.0021667	0	10970
TRPC3	-0.0021675	0	10971
FHA2	-0.002168	0	10972
BCL9	-0.0021685	0	10973
PLAGL2	-0.0021722	0	10974
GJA4	-0.0021758	0	10975
PNPLA4	-0.0021777	0	10976
LAMA4	-0.0021802	0	10977
CHPF	-0.0021846	0	10978
DCDC2	-0.0021868	0	10979
ABCB6	-0.0021877	0	10980
GPRC6A	-0.002189	0	10981
GRK6	-0.0021891	0	10982
MAGI1	-0.0021916	0	10983
SLFN11	-0.0021923	0	10984
SNORD59A	-0.002193	0	10985
MIR95	-0.0021985	0	10986
MIR331	-0.0021997	0	10987
SLC9C1	-0.0022007	0	10988
BRK1	-0.002203	0	10989
NUP43	-0.0022047	0	10990
DLX6	-0.0022062	0	10991
ASNS	-0.0022065	0	10992
SEC1P	-0.0022097	0	10993
CYP51A1	-0.0022124	0	10994
IL1F10	-0.0022146	0	10995
TMEM8C	-0.0022147	0	10996
NUB1	-0.0022153	0	10997
AQP11	-0.0022167	0	10998
CREB3L1	-0.0022174	0	10999
BTF3P11	-0.0022183	0	11000
ZNRD1	-0.0022205	0	11001
UBAP1	-0.0022212	0	11002
RBBP6	-0.0022225	0	11003

C1R	-0.0022226	0	11004
SLC19A1	-0.0022274	0	11005
TEN1	-0.0022301	0	11006
NAA10	-0.0022326	0	11007
SNRPA	-0.002234	0	11008
HCG27	-0.0022342	0	11009
IGKV2-36	-0.0022347	0	11010
SLC9A4	-0.0022371	0	11011
ZDHHC9	-0.0022393	0	11012
GNB3	-0.0022414	0	11013
SRSF4	-0.0022422	0	11014
PPP3CA	-0.0022427	0	11015
MSH4	-0.0022443	0	11016
LTC4S	-0.0022445	0	11017
MIR376B	-0.0022461	0	11018
TRIM52	-0.0022461	0	11019
TPP1	-0.0022474	0	11020
TAF1A	-0.0022483	0	11021
PBX2	-0.0022488	0	11022
MT1A	-0.0022491	0	11023
OSTF1	-0.0022528	0	11024
LDB3	-0.0022528	0	11025
DUSP19	-0.0022554	0	11026
PDE7A	-0.0022554	0	11027
INVS	-0.002259	0	11028
NUPL2	-0.0022613	0	11029
ESD	-0.0022624	0	11030
ORC4	-0.0022648	0	11031
RAD9B	-0.0022656	0	11032
USP22	-0.0022678	0	11033
RECQL5	-0.0022718	0	11034
ACACA	-0.0022732	0	11035
MTSS1	-0.0022735	0	11036
MSLN	-0.0022764	0	11037
NABP2	-0.0022771	0	11038
RTN4IP1	-0.0022795	0	11039
NR6A1	-0.0022833	0	11040
RASSF1	-0.0022841	0	11041
ITM2B	-0.0022845	0	11042
ACOT2	-0.002285	0	11043
OSGIN1	-0.0022933	0	11044
TAF9	-0.0022938	0	11045
MIR206	-0.0022949	0	11046

OTX1	-0.0022961	0	11047
NOSTRIN	-0.0023012	0	11048
CTC1	-0.0023076	0	11049
HSPA6	-0.0023099	0	11050
DDX24	-0.0023109	0	11051
MTAP	-0.0023127	0	11052
UIMC1	-0.0023153	0	11053
RAB3B	-0.0023158	0	11054
CXADR	-0.0023165	0	11055
RPL38	-0.0023176	0	11056
MIR31HG	-0.0023183	0	11057
INPP5E	-0.0023249	0	11058
TBL1X	-0.0023252	0	11059
FKBP2	-0.0023314	0	11060
SALL2	-0.0023316	0	11061
VENTXP1	-0.0023336	0	11062
SYCP3	-0.0023338	0	11063
KIAA1109	-0.0023381	0	11064
SCFD1	-0.0023383	0	11065
SLC7A5	-0.0023394	0	11066
STEAP3	-0.0023397	0	11067
CARD6	-0.0023424	0	11068
SLC9A9	-0.0023448	0	11069
FUCA1	-0.0023471	0	11070
FOXJ2	-0.0023489	0	11071
COLEC11	-0.0023494	0	11072
GCDH	-0.0023524	0	11073
PKP2	-0.0023535	0	11074
MIR570	-0.0023559	0	11075
IGKV2-29	-0.0023567	0	11076
CCNL1	-0.0023589	0	11077
DPP3	-0.002359	0	11078
PRMT3	-0.0023664	0	11079
ENTPD2	-0.0023682	0	11080
TMED2	-0.0023686	0	11081
STRA6	-0.0023693	0	11082
DPP6	-0.002372	0	11083
NEIL3	-0.002374	0	11084
SLC26A7	-0.0023746	0	11085
DPPA5	-0.0023764	0	11086
NR4A2	-0.0023801	0	11087
FFAR3	-0.0023807	0	11088
DNM3OS	-0.0023872	0	11089

CACNA1F	-0.0023875	0	11090
ERG	-0.0023876	0	11091
MIR744	-0.0023897	0	11092
BRD1	-0.0023898	0	11093
SLC30A10	-0.0023925	0	11094
CD151	-0.0023935	0	11095
ENDOV	-0.0023943	0	11096
SEC16A	-0.0023948	0	11097
IGSF5	-0.0023952	0	11098
TRAPPC4	-0.0023996	0	11099
TRHR	-0.0024012	0	11100
BCL6B	-0.0024022	0	11101
CDK5R2	-0.0024065	0	11102
CTNNA3	-0.0024081	0	11103
AFTPH	-0.0024082	0	11104
GDA	-0.0024085	0	11105
JPH2	-0.002409	0	11106
FDFT1	-0.0024094	0	11107
USP37	-0.0024115	0	11108
ADNP	-0.0024134	0	11109
SLC2A12	-0.0024141	0	11110
CALN1	-0.0024161	0	11111
ARMCX1	-0.0024173	0	11112
PSMC5	-0.0024181	0	11113
NUP107	-0.0024186	0	11114
CLN8	-0.0024207	0	11115
IDS	-0.0024215	0	11116
GNRH2	-0.0024228	0	11117
PHC2	-0.0024234	0	11118
PEPD	-0.0024332	0	11119
HGFAC	-0.0024339	0	11120
STMN3	-0.0024359	0	11121
AGPS	-0.0024364	0	11122
MIR499A	-0.0024386	0	11123
F5	-0.0024401	0	11124
LIN7C	-0.0024406	0	11125
MIR1207	-0.002441	0	11126
CNOT1	-0.0024416	0	11127
AMY1A	-0.0024426	0	11128
MED19	-0.0024449	0	11129
NPSR1	-0.002447	0	11130
HPGD	-0.002448	0	11131
CDKL5	-0.0024498	0	11132

CDX1	-0.0024509	0	11133
HIST1H2AM	-0.0024588	0	11134
TIMM50	-0.0024591	0	11135
ERAP2	-0.0024688	0	11136
SP9	-0.0024745	0	11137
MPLKIP	-0.0024747	0	11138
ST6GALNAC1	-0.0024759	0	11139
AHI1	-0.0024764	0	11140
ANXA10	-0.0024799	0	11141
SCIN	-0.0024804	0	11142
NPPB	-0.0024811	0	11143
OSBPL2	-0.002483	0	11144
ATP11A	-0.0024843	0	11145
CHRNA2	-0.0024845	0	11146
MAT1A	-0.0024888	0	11147
SOX4	-0.0024894	0	11148
INS-IGF2	-0.0024911	0	11149
CDK2AP2	-0.0024965	0	11150
PNLIPRP1	-0.0024989	0	11151
FXR2	-0.0025	0	11152
CHD5	-0.0025002	0	11153
TMED3	-0.0025073	0	11154
MYCNOS	-0.0025076	0	11155
ARMS2	-0.0025095	0	11156
SPTB	-0.0025098	0	11157
GSTA1	-0.0025113	0	11158
OMS	-0.0025144	0	11159
CLK2	-0.0025153	0	11160
TMC1	-0.0025168	0	11161
FGFBP1	-0.0025181	0	11162
MIR136	-0.0025193	0	11163
EPB41L3	-0.0025217	0	11164
ARX	-0.002522	0	11165
LCT	-0.002522	0	11166
RAB18	-0.0025272	0	11167
XYLT2	-0.0025284	0	11168
SUN1	-0.0025292	0	11169
BCO1	-0.0025308	0	11170
BRS3	-0.0025356	0	11171
RFC2	-0.0025393	0	11172
RFC4	-0.0025393	0	11173
BDNF-AS	-0.0025445	0	11174
CAPS2	-0.0025445	0	11175

WAGRO	-0.0025445	0	11176
KCNQ1OT1	-0.0025454	0	11177
TESC	-0.0025465	0	11178
LZTS2	-0.0025473	0	11179
TNFRSF19	-0.0025473	0	11180
PRKCE	-0.0025511	0	11181
COX1	-0.0025516	0	11182
SCUBE3	-0.0025551	0	11183
HSPB7	-0.002559	0	11184
PIWIL1	-0.002561	0	11185
HIVEP2	-0.0025624	0	11186
EIF4EBP3	-0.0025626	0	11187
F13B	-0.0025635	0	11188
CLDN6	-0.0025644	0	11189
CXCR6	-0.0025645	0	11190
FKBP10	-0.0025648	0	11191
TBC1D9	-0.0025669	0	11192
FGF20	-0.0025671	0	11193
CIDEB	-0.0025686	0	11194
SHE	-0.002569	0	11195
FAN1	-0.002571	0	11196
PNRC2	-0.0025719	0	11197
CHRAC1	-0.0025754	0	11198
MIR421	-0.0025758	0	11199
UMPS	-0.0025758	0	11200
SCD5	-0.0025762	0	11201
KCNJ5	-0.0025763	0	11202
PACSIN3	-0.0025785	0	11203
MORF4L1	-0.0025785	0	11204
NFX1	-0.0025793	0	11205
MIR410	-0.002582	0	11206
ZRANB2	-0.0025834	0	11207
NEUROD2	-0.0025865	0	11208
FTH1	-0.002587	0	11209
LMTK2	-0.0025881	0	11210
NINJ1	-0.0025894	0	11211
HNRNPAB	-0.0025897	0	11212
SEMA3E	-0.0025918	0	11213
MIR504	-0.0025921	0	11214
MYO1B	-0.0025927	0	11215
AXIN2	-0.0025938	0	11216
COL5A1	-0.002597	0	11217
SLC30A3	-0.0025977	0	11218

FBXO43	-0.0025998	0	11219
ISCU	-0.0026007	0	11220
SPX	-0.0026024	0	11221
DARC	-0.0026045	0	11222
PPP1R13B	-0.0026077	0	11223
ACTG1	-0.0026087	0	11224
DUX4	-0.0026131	0	11225
NPBWR1	-0.0026149	0	11226
ADH4	-0.0026178	0	11227
MND1	-0.0026195	0	11228
OTOF	-0.0026198	0	11229
SYNE2	-0.0026217	0	11230
CHRM4	-0.0026234	0	11231
SENP6	-0.0026235	0	11232
TNPO1	-0.0026248	0	11233
RNLS	-0.0026309	0	11234
MMADHC	-0.0026326	0	11235
TRIM29	-0.002635	0	11236
GP1BB	-0.0026377	0	11237
ANGPTL6	-0.0026402	0	11238
ACE	-0.0026423	0	11239
SH3BP4	-0.0026429	0	11240
SCPEP1	-0.0026436	0	11241
RYBP	-0.0026467	0	11242
MIR650	-0.0026516	0	11243
CIT	-0.0026549	0	11244
MBOAT4	-0.002658	0	11245
SIRT5	-0.0026587	0	11246
SCTR	-0.0026597	0	11247
ALKBH2	-0.0026641	0	11248
PNN	-0.0026642	0	11249
ACOT12	-0.0026657	0	11250
TRAPPC3	-0.0026657	0	11251
DKK3	-0.0026659	0	11252
DPPA2	-0.0026665	0	11253
USP16	-0.0026684	0	11254
MIR20B	-0.0026696	0	11255
NDUFB3	-0.0026711	0	11256
BP7	-0.0026713	0	11257
ICE1	-0.0026742	0	11258
CCDC85B	-0.0026744	0	11259
MMP24	-0.002675	0	11260
ACOT9	-0.002676	0	11261

F12	-0.0026771	0	11262
TOX	-0.0026775	0	11263
ZFY	-0.0026786	0	11264
RDH11	-0.002679	0	11265
BNIP3L	-0.0026796	0	11266
POU4F2	-0.0026839	0	11267
CBX2	-0.002689	0	11268
GABBR2	-0.0026892	0	11269
MIR1268A	-0.0026899	0	11270
PFKP	-0.0026901	0	11271
FBXO32	-0.0026915	0	11272
HMGCR	-0.0026923	0	11273
PLEKHF1	-0.0026939	0	11274
CHD1	-0.0026947	0	11275
PIFO	-0.0026955	0	11276
FMR1NB	-0.0026962	0	11277
TPMT	-0.0026969	0	11278
CREG1	-0.0027002	0	11279
SNORD48	-0.0027004	0	11280
DSCC1	-0.0027051	0	11281
PRM3	-0.0027063	0	11282
MTNR1A	-0.0027083	0	11283
CARD14	-0.002712	0	11284
SMYD2	-0.0027148	0	11285
SOX21	-0.0027171	0	11286
SUFU	-0.0027178	0	11287
ALAD	-0.0027184	0	11288
ECI1	-0.0027186	0	11289
ACSBG1	-0.0027193	0	11290
SHISA5	-0.0027197	0	11291
DLEC1	-0.0027202	0	11292
GSR	-0.0027225	0	11293
ZBED1	-0.0027226	0	11294
GAL3ST2	-0.0027244	0	11295
CLDN4	-0.002727	0	11296
CALCRL	-0.0027277	0	11297
BCL9L	-0.0027291	0	11298
TBXAS1	-0.0027316	0	11299
CHTF18	-0.0027316	0	11300
RHBDF2	-0.002736	0	11301
SLC6A11	-0.0027365	0	11302
SEPW1	-0.0027393	0	11303
EIF4A3	-0.0027455	0	11304

TXN2	-0.0027474	0	11305
JARID2	-0.0027475	0	11306
UNC5A	-0.002749	0	11307
LPIN3	-0.0027536	0	11308
SLC25A24	-0.0027564	0	11309
COL10A1	-0.0027568	0	11310
L1TD1	-0.002758	0	11311
RRM2	-0.0027617	0	11312
PPP2R1A	-0.0027637	0	11313
CYP11B1	-0.0027676	0	11314
RNY3	-0.0027679	0	11315
MYCBP2	-0.0027719	0	11316
GSTA4	-0.0027739	0	11317
KCNC3	-0.002774	0	11318
NLGN1	-0.0027778	0	11319
NT5C1A	-0.0027785	0	11320
NCAN	-0.0027797	0	11321
PPM1H	-0.0027805	0	11322
OLIG3	-0.0027816	0	11323
HNRNPD	-0.0027829	0	11324
SEMA3C	-0.0027832	0	11325
WIF1	-0.0027834	0	11326
FZD9	-0.0027859	0	11327
HULC	-0.0027882	0	11328
SNRPD2	-0.0027919	0	11329
PRH1	-0.0027929	0	11330
ABCC11	-0.0027936	0	11331
CCA1	-0.0027999	0	11332
NDUFS1	-0.0028019	0	11333
TBC1D7	-0.0028019	0	11334
TAOK3	-0.0028024	0	11335
PYCR1	-0.0028032	0	11336
CALCB	-0.0028043	0	11337
USE1	-0.0028101	0	11338
MIR190A	-0.0028102	0	11339
NFYA	-0.0028108	0	11340
FAM213A	-0.0028119	0	11341
MIR1-1	-0.002817	0	11342
CECR1	-0.0028207	0	11343
SYT7	-0.0028239	0	11344
MS4A3	-0.0028243	0	11345
ANKRD6	-0.0028244	0	11346
POLK	-0.0028251	0	11347

SLC27A2	-0.002828	0	11348
AMD1	-0.0028307	0	11349
OATP1	-0.0028312	0	11350
PAICS	-0.002834	0	11351
MIR124-1	-0.0028341	0	11352
SSD	-0.0028342	0	11353
GPR32	-0.0028354	0	11354
PDE7B	-0.0028354	0	11355
VCX	-0.0028354	0	11356
MIR485	-0.0028359	0	11357
SEMA3F	-0.0028371	0	11358
GTF2A1	-0.002839	0	11359
ERO1L	-0.0028418	0	11360
KCNS3	-0.0028443	0	11361
SKA2	-0.0028464	0	11362
C2	-0.002851	0	11363
NDUFV2	-0.0028516	0	11364
CCDC112	-0.0028534	0	11365
GRHL1	-0.0028609	0	11366
PADI4	-0.0028648	0	11367
CRCP	-0.0028656	0	11368
SLC39A8	-0.0028687	0	11369
NUP160	-0.0028699	0	11370
NAT1	-0.0028704	0	11371
NSA2	-0.002871	0	11372
PDE6B	-0.0028734	0	11373
NPFFR1	-0.0028756	0	11374
MAML2	-0.0028787	0	11375
MIR1224	-0.0028789	0	11376
SAT2	-0.0028837	0	11377
GPX4	-0.002885	0	11378
CITED2	-0.0028884	0	11379
GPC4	-0.0028905	0	11380
MIR30E	-0.0028911	0	11381
DPF2	-0.002893	0	11382
SHANK2	-0.0028967	0	11383
COL15A1	-0.0028974	0	11384
PDP2	-0.0029001	0	11385
SLU7	-0.0029001	0	11386
FILIP1L	-0.002905	0	11387
SLC47A2	-0.0029058	0	11388
FOXE3	-0.0029082	0	11389
TRIM44	-0.0029115	0	11390

SCLY	-0.0029137	0	11391
MIR130B	-0.0029219	0	11392
GLYCTK	-0.0029235	0	11393
TGIF2	-0.0029258	0	11394
ZMPSTE24	-0.0029272	0	11395
TRIML2	-0.0029299	0	11396
NRBF2	-0.0029333	0	11397
INO80B	-0.0029335	0	11398
LGALS2	-0.0029337	0	11399
SCN8A	-0.0029341	0	11400
ACD	-0.0029352	0	11401
MIR608	-0.0029356	0	11402
GRHL3	-0.002936	0	11403
KRT71	-0.0029374	0	11404
MAPK6	-0.0029381	0	11405
SYT4	-0.0029393	0	11406
APLF	-0.0029402	0	11407
STOM	-0.0029482	0	11408
APTX	-0.0029483	0	11409
MGST3	-0.0029514	0	11410
AKAP8L	-0.0029539	0	11411
PTPN14	-0.0029556	0	11412
DDIT4L	-0.0029567	0	11413
MIR4271	-0.0029579	0	11414
AAAS	-0.0029593	0	11415
ZNF236	-0.0029598	0	11416
MOGAT2	-0.0029614	0	11417
HMX1	-0.0029626	0	11418
TBX19	-0.0029656	0	11419
ST6GAL1	-0.0029677	0	11420
COPE	-0.0029716	0	11421
ANKRD26	-0.0029716	0	11422
FHIT	-0.0029724	0	11423
PDIA2	-0.0029735	0	11424
RASSF10	-0.0029759	0	11425
GPX3	-0.0029764	0	11426
UTP14C	-0.0029776	0	11427
HABP4	-0.0029778	0	11428
SNAP23	-0.0029821	0	11429
EBF2	-0.0029822	0	11430
WSB1	-0.0029833	0	11431
MYOF	-0.002988	0	11432
PF4V1	-0.0029949	0	11433

ENOSF1	-0.0029976	0	11434
TRNAR1	-0.0030013	0	11435
BET1	-0.0030016	0	11436
MIR33A	-0.0030044	0	11437
EFEMP2	-0.0030061	0	11438
RBP3	-0.0030064	0	11439
TXNL4B	-0.0030071	0	11440
HES7	-0.0030074	0	11441
EN2	-0.0030084	0	11442
SUPT6H	-0.003011	0	11443
CCBL1	-0.0030138	0	11444
GABRG2	-0.0030146	0	11445
BZX	-0.0030151	0	11446
GLI3	-0.0030152	0	11447
NPB	-0.0030158	0	11448
IGKV1-17	-0.0030162	0	11449
PRM2	-0.0030228	0	11450
BFSP2	-0.0030228	0	11451
SERPINB7	-0.0030238	0	11452
NSUN2	-0.0030254	0	11453
ENHO	-0.0030257	0	11454
ADAMTS3	-0.0030295	0	11455
IRX1	-0.0030339	0	11456
CAMKMT	-0.0030343	0	11457
GCFC2	-0.0030348	0	11458
LAMTOR5	-0.003036	0	11459
PTH2R	-0.0030427	0	11460
KDM5A	-0.0030437	0	11461
CREB5	-0.0030497	0	11462
VKORC1	-0.0030505	0	11463
CST6	-0.0030518	0	11464
DEFB114	-0.003053	0	11465
MIR181D	-0.0030532	0	11466
SLC4A9	-0.0030542	0	11467
TM7SF2	-0.0030552	0	11468
NAPEPLD	-0.0030554	0	11469
PZP	-0.0030579	0	11470
MAPRE3	-0.0030602	0	11471
ZBTB38	-0.0030612	0	11472
POU5F1B	-0.0030628	0	11473
ERC2	-0.0030637	0	11474
SMC3	-0.0030664	0	11475
ATMIN	-0.0030667	0	11476

UBL4A	-0.0030688	0	11477
APRT	-0.0030746	0	11478
SOHLH2	-0.0030747	0	11479
SCP2	-0.0030772	0	11480
NSFL1C	-0.0030785	0	11481
ZAR1	-0.0030804	0	11482
WDFY2	-0.0030833	0	11483
COX4I1	-0.0030856	0	11484
MIR664	-0.0030858	0	11485
ZGLP1	-0.0030866	0	11486
NPW	-0.0030875	0	11487
DNASE2	-0.0030883	0	11488
MIR328	-0.0030893	0	11489
UCN3	-0.0030918	0	11490
FZD7	-0.0030924	0	11491
TFAM	-0.0030926	0	11492
NPR1	-0.0030931	0	11493
PRND	-0.0030947	0	11494
SRSF9	-0.0030994	0	11495
GRK4	-0.0031064	0	11496
TRPV3	-0.0031106	0	11497
CDKN2C	-0.0031132	0	11498
CCRN4L	-0.0031176	0	11499
ARID1A	-0.0031202	0	11500
RCL	-0.0031252	0	11501
PRKACB	-0.0031271	0	11502
MGRN1	-0.0031314	0	11503
HNRNPL	-0.0031328	0	11504
IRX3	-0.0031329	0	11505
CMTM1	-0.0031358	0	11506
EIF3I	-0.0031363	0	11507
CLK1	-0.0031397	0	11508
DFFA	-0.0031423	0	11509
TFDP1	-0.0031427	0	11510
NAV3	-0.0031439	0	11511
ALDH5A1	-0.0031453	0	11512
GLA	-0.0031453	0	11513
ANGPTL3	-0.0031461	0	11514
CHEK2	-0.003148	0	11515
ARG2	-0.0031505	0	11516
MT1G	-0.0031507	0	11517
TFB1M	-0.0031524	0	11518
MBD1	-0.0031544	0	11519

C4ORF26	-0.0031552	0	11520
INMT	-0.0031552	0	11521
MTG1	-0.0031552	0	11522
SAP30L	-0.0031552	0	11523
SLC17A9	-0.0031552	0	11524
SPATA12	-0.0031552	0	11525
TMEM97	-0.0031552	0	11526
FKRP	-0.0031568	0	11527
LINC-ROR	-0.0031578	0	11528
SYT1	-0.003161	0	11529
POLQ	-0.0031654	0	11530
STYK1	-0.003168	0	11531
NEFM	-0.0031713	0	11532
DEFB104A	-0.0031713	0	11533
MXD1	-0.0031722	0	11534
NOS1AP	-0.0031726	0	11535
H2AFY	-0.0031767	0	11536
IRX2	-0.0031789	0	11537
NELL1	-0.0031798	0	11538
AMY2B	-0.0031817	0	11539
TAF1	-0.0031829	0	11540
RANBP9	-0.0031831	0	11541
SMCP	-0.0031902	0	11542
DPYSL3	-0.0031929	0	11543
CLDN7	-0.0031941	0	11544
EFHD1	-0.0031975	0	11545
GOLGA6L2	-0.0031978	0	11546
VLDLR	-0.003198	0	11547
LIN9	-0.0031996	0	11548
LARP6	-0.003203	0	11549
HEPH	-0.0032033	0	11550
SLC9A8	-0.0032043	0	11551
PDE3A	-0.0032062	0	11552
MTHFD1L	-0.0032066	0	11553
NUDT21	-0.0032072	0	11554
LMTK3	-0.0032092	0	11555
MEF2D	-0.0032108	0	11556
SAT1	-0.0032121	0	11557
MA	-0.0032131	0	11558
PRKG2	-0.003214	0	11559
ACTC1	-0.0032179	0	11560
HMGA1	-0.0032193	0	11561
SLFN12	-0.0032194	0	11562

SPATA18	-0.0032201	0	11563
TMPRSS15	-0.0032236	0	11564
POLR3F	-0.0032255	0	11565
POLR3K	-0.0032263	0	11566
NLGN4X	-0.0032267	0	11567
ATXN7	-0.0032277	0	11568
SEPHS1	-0.0032298	0	11569
DMPK	-0.0032308	0	11570
GPR68	-0.0032342	0	11571
NEK2	-0.0032419	0	11572
ADAM33	-0.0032432	0	11573
PAPD4	-0.0032438	0	11574
CDK16	-0.003244	0	11575
OVOL1	-0.0032477	0	11576
CCAT1	-0.0032481	0	11577
TSR2	-0.0032481	0	11578
GJA3	-0.0032487	0	11579
FCAMR	-0.0032491	0	11580
GABRA4	-0.0032491	0	11581
GPCPD1	-0.0032491	0	11582
MIR1183	-0.0032491	0	11583
MIR302C	-0.0032491	0	11584
MIR652	-0.0032491	0	11585
PHYHIPL	-0.0032491	0	11586
SERPINB11	-0.0032491	0	11587
SLC22A23	-0.0032491	0	11588
UTS2D	-0.0032491	0	11589
CES2	-0.0032504	0	11590
IQCB1	-0.0032542	0	11591
EEF1G	-0.0032579	0	11592
STAG2	-0.0032591	0	11593
RPS13	-0.0032616	0	11594
FOXK2	-0.0032619	0	11595
REV3L	-0.0032662	0	11596
PEX1	-0.003267	0	11597
PROKR1	-0.0032716	0	11598
CRABP2	-0.0032723	0	11599
SLC10A1	-0.0032724	0	11600
AVPR2	-0.0032789	0	11601
CDK14	-0.0032794	0	11602
SMN2	-0.0032812	0	11603
MIR1303	-0.0032816	0	11604
CCT2	-0.003287	0	11605

IL36RN	-0.0032887	0	11606
HOXA5	-0.0032908	0	11607
DYRK2	-0.0032912	0	11608
AK1	-0.003292	0	11609
SHISA9	-0.0032972	0	11610
TMEM35	-0.0032972	0	11611
CGB7	-0.0032996	0	11612
DHRS3	-0.0033017	0	11613
RAD21	-0.003305	0	11614
CBX7	-0.0033087	0	11615
EDNRB	-0.0033129	0	11616
BAIAP2L1	-0.003314	0	11617
MIR605	-0.0033148	0	11618
7-Sep	-0.0033148	0	11619
TMPRSS2	-0.0033162	0	11620
KCNJ3	-0.0033164	0	11621
UHRF1	-0.0033172	0	11622
FCHSD1	-0.0033184	0	11623
TNR1	-0.0033184	0	11624
GPSM2	-0.003319	0	11625
RBM12	-0.0033237	0	11626
CLIC1	-0.0033249	0	11627
LMBRD1	-0.0033255	0	11628
PRPF19	-0.0033286	0	11629
UPK3B	-0.0033327	0	11630
KCNH2	-0.0033335	0	11631
SSRP1	-0.0033377	0	11632
SCG3	-0.0033406	0	11633
AEN	-0.0033436	0	11634
C9ORF116	-0.0033436	0	11635
CHRNB1	-0.0033436	0	11636
CREBZF	-0.0033436	0	11637
DBH-AS1	-0.0033436	0	11638
ELL3	-0.0033436	0	11639
EPN3	-0.0033436	0	11640
FBXL20	-0.0033436	0	11641
FBXO42	-0.0033436	0	11642
FNBP4	-0.0033436	0	11643
GPR87	-0.0033436	0	11644
HYDIN	-0.0033436	0	11645
PALMD	-0.0033436	0	11646
PANK1	-0.0033436	0	11647
PPAP2C	-0.0033436	0	11648

PRG3	-0.0033436	0	11649
PRODH2	-0.0033436	0	11650
RAB6C	-0.0033436	0	11651
RASSF3	-0.0033436	0	11652
RPS27L	-0.0033436	0	11653
SIAH1P1	-0.0033436	0	11654
SMG6	-0.0033436	0	11655
SMPD4	-0.0033436	0	11656
TP53I11	-0.0033436	0	11657
TP53I13	-0.0033436	0	11658
TP53TG3	-0.0033436	0	11659
TRIM39	-0.0033436	0	11660
UBTD1	-0.0033436	0	11661
UBXN2A	-0.0033436	0	11662
YPEL3	-0.0033436	0	11663
KLLN	-0.0033464	0	11664
PRCP	-0.0033468	0	11665
NUTF2	-0.0033493	0	11666
RFC5	-0.0033516	0	11667
PTMA	-0.0033519	0	11668
SLX4	-0.0033527	0	11669
HEPACAM	-0.0033554	0	11670
COMMD1	-0.0033584	0	11671
LAYN	-0.0033615	0	11672
EPB41L2	-0.0033627	0	11673
NBPF1	-0.0033628	0	11674
RAD23B	-0.0033672	0	11675
UGT8	-0.0033715	0	11676
BLM	-0.003377	0	11677
KRT6B	-0.0033805	0	11678
SGK3	-0.0033806	0	11679
RMST	-0.0033831	0	11680
FGF14	-0.0033853	0	11681
TSGA10	-0.0033869	0	11682
HMGB3	-0.0033888	0	11683
PKP1	-0.0033921	0	11684
KIF2B	-0.0033958	0	11685
MLH3	-0.0033961	0	11686
GLYAT	-0.0033974	0	11687
GTSE1	-0.0033987	0	11688
MPG	-0.003401	0	11689
PEMT	-0.0034054	0	11690
KDM3A	-0.0034081	0	11691

TRPV4	-0.0034116	0	11692
WNT7A	-0.0034119	0	11693
TARBP2	-0.0034139	0	11694
SERPINI1	-0.0034142	0	11695
MIR660	-0.0034162	0	11696
REN	-0.0034207	0	11697
MIR195	-0.0034221	0	11698
EIF6	-0.0034234	0	11699
PAR5	-0.0034279	0	11700
CNTRL	-0.0034293	0	11701
NDOR1	-0.0034301	0	11702
MAFA	-0.0034309	0	11703
NCALD	-0.003431	0	11704
RTN1	-0.0034311	0	11705
CAGE1	-0.0034315	0	11706
CENPA	-0.003435	0	11707
GSTA3	-0.0034352	0	11708
TRIAP1	-0.003436	0	11709
MRBC	-0.0034403	0	11710
KLK11	-0.0034439	0	11711
IER2	-0.0034507	0	11712
RHCG	-0.003451	0	11713
TNFAIP1	-0.0034536	0	11714
TSHZ1	-0.0034542	0	11715
CLDN14	-0.0034559	0	11716
NELL2	-0.0034609	0	11717
RPL15	-0.003462	0	11718
PFN2	-0.0034629	0	11719
IFITM5	-0.0034636	0	11720
COPG2	-0.0034637	0	11721
MAGEA11	-0.0034661	0	11722
RBM14	-0.0034665	0	11723
UPP1	-0.0034691	0	11724
CUL9	-0.00347	0	11725
TNFSF14	-0.0034724	0	11726
TFF3	-0.0034733	0	11727
TMPRSS4	-0.0034751	0	11728
CHAC1	-0.0034789	0	11729
LPCAT2	-0.0034806	0	11730
CYP2A7	-0.0034824	0	11731
GRIN3A	-0.0034831	0	11732
RCHY1	-0.003484	0	11733
GLS	-0.0034841	0	11734

FANCE	-0.0034858	0	11735
NLGN2	-0.003489	0	11736
FMOD	-0.0034927	0	11737
HIP1	-0.003494	0	11738
HRH3	-0.0034945	0	11739
SLC12A9	-0.0034949	0	11740
B3GALT4	-0.0034977	0	11741
NUBP1	-0.0034981	0	11742
PDE1A	-0.0034986	0	11743
URGCP	-0.0034988	0	11744
GLIS3	-0.0034992	0	11745
NEIL2	-0.0035017	0	11746
ETV3	-0.0035043	0	11747
CCDC170	-0.0035084	0	11748
FADS1	-0.0035087	0	11749
TSPAN12	-0.0035087	0	11750
RTCA	-0.0035109	0	11751
SRD5A2	-0.0035111	0	11752
STRN4	-0.0035124	0	11753
RBPMS	-0.0035133	0	11754
GJB6	-0.0035139	0	11755
S100A11	-0.0035182	0	11756
VPS54	-0.0035188	0	11757
SPPL3	-0.0035196	0	11758
TMEM27	-0.0035203	0	11759
FAM5C	-0.003522	0	11760
PCDH9	-0.003526	0	11761
EGLN2	-0.0035316	0	11762
DICER1	-0.0035319	0	11763
YTHDF2	-0.0035376	0	11764
HOPX	-0.0035418	0	11765
H6PD	-0.0035424	0	11766
MIR202	-0.0035459	0	11767
THEG	-0.0035459	0	11768
ABR	-0.0035461	0	11769
DUOXA1	-0.0035501	0	11770
ABHD1	-0.0035512	0	11771
ART4	-0.0035512	0	11772
BZW1	-0.0035512	0	11773
GPM6B	-0.0035512	0	11774
GRIP2	-0.0035512	0	11775
IER5	-0.0035512	0	11776
IFRD2	-0.0035512	0	11777

LUZP4	-0.0035512	0	11778
MIR1202	-0.0035512	0	11779
NAA30	-0.0035512	0	11780
PDE8A	-0.0035512	0	11781
PIP4K2B	-0.0035512	0	11782
RFWD3	-0.0035512	0	11783
SAI1	-0.0035512	0	11784
SLC44A4	-0.0035512	0	11785
ALOX12	-0.003553	0	11786
RAD51C	-0.003555	0	11787
HNRNPA2B1	-0.0035601	0	11788
ADH1C	-0.0035615	0	11789
RAB7L1	-0.0035618	0	11790
MGARP	-0.0035632	0	11791
OSTN	-0.0035634	0	11792
MDGA1	-0.0035657	0	11793
MAP1B	-0.0035667	0	11794
CTSD	-0.0035723	0	11795
RTP3	-0.0035774	0	11796
LIN7A	-0.003582	0	11797
TAGLN2	-0.0035824	0	11798
GLIPR1L1	-0.003583	0	11799
GLIPR1L2	-0.003583	0	11800
THAP1	-0.0035836	0	11801
GLTSCR2	-0.0035841	0	11802
MIR338	-0.0035865	0	11803
RPH3A	-0.0035895	0	11804
STSP1	-0.0035914	0	11805
GOLGA1	-0.0035923	0	11806
FABP5	-0.0035964	0	11807
MMP19	-0.0035989	0	11808
USP10	-0.0036006	0	11809
MORC4	-0.0036008	0	11810
VSX2	-0.0036071	0	11811
STX2	-0.0036072	0	11812
MT-CO3	-0.0036099	0	11813
UNC5D	-0.00361	0	11814
AGA	-0.0036115	0	11815
FN1	-0.0036118	0	11816
HGH1	-0.0036164	0	11817
FECB	-0.0036193	0	11818
CTCFL	-0.0036203	0	11819
HSPB6	-0.0036218	0	11820

ABAT	-0.0036236	0	11821
HOXA4	-0.0036271	0	11822
BDKRB1	-0.00363	0	11823
ACSM3	-0.0036318	0	11824
ASNA1	-0.0036336	0	11825
MEST	-0.0036355	0	11826
CDC42SE2	-0.0036355	0	11827
KRT6A	-0.0036356	0	11828
AGR2	-0.0036401	0	11829
OBP2A	-0.0036438	0	11830
ADCY10	-0.0036474	0	11831
RPL24	-0.0036478	0	11832
P2RX1	-0.0036492	0	11833
CDK18	-0.0036506	0	11834
COL4A1	-0.0036529	0	11835
RPL14	-0.0036552	0	11836
PSME3	-0.0036573	0	11837
SNORD46	-0.0036623	0	11838
DCTN5	-0.0036636	0	11839
SPRY1	-0.0036642	0	11840
PHLPP2	-0.0036649	0	11841
GPKOW	-0.0036678	0	11842
TRIOBP	-0.0036684	0	11843
NHLRC1	-0.0036711	0	11844
APOPT1	-0.0036714	0	11845
DTNB	-0.0036715	0	11846
ABCB10	-0.0036716	0	11847
MIR892A	-0.0036732	0	11848
SYNM	-0.003678	0	11849
TAF10	-0.0036797	0	11850
SFTPB	-0.0036804	0	11851
LIPA	-0.0036811	0	11852
DBT	-0.0036817	0	11853
ATP11C	-0.0036845	0	11854
MFRP	-0.0036859	0	11855
DDC	-0.0036868	0	11856
DUSP10	-0.0036903	0	11857
SRP14	-0.0036946	0	11858
ERAP1	-0.0036988	0	11859
ADCY4	-0.0037049	0	11860
MGP	-0.0037055	0	11861
PTMS	-0.0037058	0	11862
AK6	-0.0037096	0	11863

ATAD5	-0.0037142	0	11864
MT-TR	-0.0037148	0	11865
DAO	-0.0037186	0	11866
NPY4R	-0.0037194	0	11867
TCF15	-0.00372	0	11868
ST3GAL1	-0.0037247	0	11869
ADRA2C	-0.0037265	0	11870
STX4	-0.0037272	0	11871
STK40	-0.0037281	0	11872
GRIK4	-0.0037306	0	11873
BNC2	-0.0037319	0	11874
BSX	-0.0037373	0	11875
STMN4	-0.0037406	0	11876
RSPO1	-0.0037413	0	11877
TRAP1	-0.0037448	0	11878
AKAP12	-0.0037453	0	11879
RLBP1	-0.0037478	0	11880
SSB	-0.0037481	0	11881
SGTA	-0.0037492	0	11882
SOAT1	-0.0037522	0	11883
PNP	-0.0037524	0	11884
PCTP	-0.0037524	0	11885
GPR83	-0.0037557	0	11886
NEK11	-0.0037567	0	11887
LACE1	-0.0037594	0	11888
HINFP	-0.0037595	0	11889
M6PR	-0.0037606	0	11890
MIR487A	-0.0037614	0	11891
PCSK1N	-0.0037623	0	11892
DBN1	-0.003764	0	11893
LCH	-0.0037701	0	11894
HYAL1	-0.0037766	0	11895
ZMAT3	-0.0037778	0	11896
INCENP	-0.0037817	0	11897
MIR508	-0.0037826	0	11898
MBNL1	-0.0037828	0	11899
GNS	-0.0037841	0	11900
DDX1	-0.0037844	0	11901
PKMYT1	-0.003787	0	11902
MIR23A	-0.0037908	0	11903
PRR4	-0.0037935	0	11904
PCDH10	-0.0037956	0	11905
APEH	-0.0037968	0	11906

SPON1	-0.0037969	0	11907
ASIP	-0.003798	0	11908
NOTCH3	-0.003802	0	11909
PRM1	-0.0038042	0	11910
STAR	-0.0038077	0	11911
FAM129A	-0.0038079	0	11912
F2RL3	-0.0038084	0	11913
C3ORF18	-0.0038113	0	11914
TWF1	-0.003813	0	11915
ADAM23	-0.0038139	0	11916
PDP1	-0.0038171	0	11917
NHEJ1	-0.0038181	0	11918
CREB3L2	-0.0038211	0	11919
COL14A1	-0.0038219	0	11920
FMO3	-0.0038257	0	11921
IHH	-0.0038264	0	11922
CASP6	-0.0038276	0	11923
MIR133B	-0.003833	0	11924
SLC39A14	-0.003834	0	11925
CHKB	-0.0038342	0	11926
MIR630	-0.0038353	0	11927
KCNE4	-0.0038365	0	11928
SLC14A1	-0.0038376	0	11929
GPR124	-0.0038383	0	11930
PSPC1	-0.0038383	0	11931
PON2	-0.0038397	0	11932
AKIRIN2	-0.0038424	0	11933
KIR2DL3	-0.0038432	0	11934
MED13	-0.003846	0	11935
MTMR11	-0.0038468	0	11936
OPRL1	-0.0038508	0	11937
MIR1254-1	-0.0038515	0	11938
KRT79	-0.003853	0	11939
YY2	-0.0038536	0	11940
ALAS1	-0.0038562	0	11941
SLC38A2	-0.003857	0	11942
LOXL1	-0.0038587	0	11943
TPSAB1	-0.003861	0	11944
GGNBP2	-0.003862	0	11945
EFHD2	-0.0038646	0	11946
KLF12	-0.0038646	0	11947
SERPINB10	-0.0038653	0	11948
MIR484	-0.0038701	0	11949

PGLS	-0.0038838	0	11950
CAD	-0.0038871	0	11951
AP1S2	-0.0038893	0	11952
EIF4ENIF1	-0.003892	0	11953
DCP2	-0.0038976	0	11954
DCLRE1C	-0.0038983	0	11955
KRT83	-0.0038983	0	11956
MIR199B	-0.0039011	0	11957
MIR373	-0.0039049	0	11958
TXNRD3	-0.0039077	0	11959
MASP1	-0.0039078	0	11960
RHNO1	-0.0039097	0	11961
MIR346	-0.0039128	0	11962
HOTAIR	-0.0039138	0	11963
STXBP5	-0.0039153	0	11964
NINL	-0.0039183	0	11965
NPTX2	-0.0039194	0	11966
BTBD10	-0.0039204	0	11967
ZW10	-0.0039251	0	11968
ARSH	-0.0039264	0	11969
CYP4F11	-0.0039264	0	11970
MLK4	-0.0039264	0	11971
SNN	-0.0039264	0	11972
WDR35	-0.0039264	0	11973
CDCA8	-0.0039275	0	11974
NTHL1	-0.0039295	0	11975
ACTN2	-0.0039342	0	11976
USP4	-0.0039349	0	11977
MEOX2	-0.0039358	0	11978
CTSH	-0.003936	0	11979
BP10	-0.0039364	0	11980
GRID2	-0.0039379	0	11981
HN1	-0.0039386	0	11982
SPG11	-0.003941	0	11983
KLF7	-0.00395	0	11984
ST8SIA2	-0.0039554	0	11985
RPL4	-0.003957	0	11986
GRIN3B	-0.0039576	0	11987
CIDEA	-0.003961	0	11988
MIR10A	-0.0039673	0	11989
RAD18	-0.0039682	0	11990
CYP8B1	-0.0039682	0	11991
REC114	-0.0039684	0	11992

MFGE8	-0.0039695	0	11993
ASIC5	-0.0039718	0	11994
AGO1	-0.0039795	0	11995
NUAK2	-0.0039798	0	11996
NTSR2	-0.0039829	0	11997
IH	-0.003983	0	11998
AHSG	-0.0039831	0	11999
MAMLD1	-0.0039872	0	12000
SOX30	-0.0039877	0	12001
BCKDK	-0.0039878	0	12002
GAST	-0.003989	0	12003
SNORD14B	-0.0039899	0	12004
DCN	-0.0039899	0	12005
TBC1D1	-0.0039928	0	12006
MIR301A	-0.0039972	0	12007
MYO10	-0.0040094	0	12008
METTL3	-0.0040121	0	12009
DACT2	-0.0040124	0	12010
BBS9	-0.0040125	0	12011
GABRA3	-0.0040127	0	12012
SELK	-0.0040128	0	12013
CST5	-0.0040165	0	12014
SFI1	-0.0040186	0	12015
GFRA3	-0.0040216	0	12016
UNC13A	-0.0040244	0	12017
ESCO1	-0.0040248	0	12018
SLC27A6	-0.0040269	0	12019
MFI2	-0.0040275	0	12020
PLK2	-0.0040307	0	12021
HTR7	-0.004032	0	12022
BDKRB2	-0.0040332	0	12023
APLP1	-0.0040334	0	12024
NDUFB9	-0.0040338	0	12025
RDH5	-0.0040371	0	12026
GPAM	-0.0040423	0	12027
MDM4	-0.0040447	0	12028
ATRN	-0.0040524	0	12029
HSPB2	-0.0040526	0	12030
SERPINC1	-0.0040532	0	12031
GPS2	-0.004054	0	12032
TXNDC5	-0.0040541	0	12033
RLN2	-0.0040556	0	12034
CUL4A	-0.0040561	0	12035

CABIN1	-0.0040628	0	12036
UCHL1	-0.0040673	0	12037
CKAP5	-0.0040697	0	12038
UFC1	-0.0040711	0	12039
COX17	-0.0040746	0	12040
MIR1228	-0.004075	0	12041
SCAI	-0.0040752	0	12042
HES6	-0.0040766	0	12043
MIR370	-0.0040786	0	12044
FERMT2	-0.0040824	0	12045
NEAT1	-0.0040915	0	12046
CCDC80	-0.0040923	0	12047
CTSL2	-0.0040979	0	12048
MTNR1B	-0.0041012	0	12049
ESYT1	-0.0041026	0	12050
USP28	-0.004105	0	12051
EIF5A2	-0.0041069	0	12052
ADC	-0.004118	0	12053
BCCIP	-0.0041253	0	12054
BEND3	-0.0041258	0	12055
HECW2	-0.0041263	0	12056
KRT90P	-0.0041265	0	12057
TFDP2	-0.0041315	0	12058
CXCL5	-0.0041323	0	12059
TELO2	-0.0041336	0	12060
CNGB3	-0.0041371	0	12061
TAS2R13	-0.0041411	0	12062
DNAJC5	-0.0041418	0	12063
TNRC6A	-0.0041419	0	12064
ASPH	-0.0041435	0	12065
FGF16	-0.0041444	0	12066
SLC27A1	-0.0041467	0	12067
STAG1	-0.0041508	0	12068
CDC14B	-0.0041531	0	12069
SLC28A2	-0.0041531	0	12070
CRYBB1	-0.0041553	0	12071
LPO	-0.004158	0	12072
INPP5B	-0.0041582	0	12073
MIR149	-0.0041598	0	12074
LNPEP	-0.0041613	0	12075
MC1R	-0.004164	0	12076
CYSLTR2	-0.0041648	0	12077
TAAR6	-0.0041658	0	12078

KBTBD11	-0.0041686	0	12079
SLC24A3	-0.0041749	0	12080
KLK1	-0.004175	0	12081
CEP250	-0.0041777	0	12082
FUBP1	-0.004181	0	12083
NCSTN	-0.0041814	0	12084
DFNA5	-0.0041839	0	12085
FOXL2	-0.0041909	0	12086
VV	-0.0041918	0	12087
TBRG1	-0.0041937	0	12088
PPP1R13L	-0.0041997	0	12089
IGKV2D-19	-0.0042007	0	12090
FECD3	-0.0042016	0	12091
ACADM	-0.0042022	0	12092
GCGR	-0.0042036	0	12093
CDH6	-0.0042062	0	12094
MT1F	-0.0042108	0	12095
ORC6	-0.0042112	0	12096
LHX1	-0.0042117	0	12097
PSMC3IP	-0.004212	0	12098
HOXB5	-0.0042135	0	12099
APLP2	-0.0042142	0	12100
CS	-0.0042174	0	12101
CACNA1G	-0.0042211	0	12102
PLAG1	-0.0042215	0	12103
KAP	-0.0042257	0	12104
LOX	-0.0042294	0	12105
CPLX2	-0.0042327	0	12106
PARL	-0.0042328	0	12107
CLDN1	-0.0042336	0	12108
CAMKK2	-0.0042355	0	12109
PGAM1	-0.0042394	0	12110
SNTB1	-0.0042414	0	12111
WDHD1	-0.0042482	0	12112
MED17	-0.0042485	0	12113
KAR	-0.0042544	0	12114
EIF4E3	-0.0042602	0	12115
ELOVL2	-0.0042604	0	12116
CTNNBIP1	-0.0042619	0	12117
TTR	-0.0042621	0	12118
MIR335	-0.0042724	0	12119
SRSF7	-0.004277	0	12120
RNF39	-0.0042776	0	12121

ATG4A	-0.0042807	0	12122
PIF1	-0.0042826	0	12123
KIRREL2	-0.0042843	0	12124
MIR541	-0.0042906	0	12125
MRGPRX1	-0.0042922	0	12126
CHR	-0.0042945	0	12127
COL9A1	-0.0042961	0	12128
CDCA7L	-0.0042973	0	12129
MMP25	-0.0043105	0	12130
C6ORF15	-0.0043108	0	12131
SLC22A3	-0.0043126	0	12132
RPGRIP1L	-0.0043127	0	12133
SPESP1	-0.0043137	0	12134
DTL	-0.0043157	0	12135
FDXR	-0.0043165	0	12136
RRN3	-0.0043177	0	12137
MIR1246	-0.0043212	0	12138
TGFBR3	-0.0043213	0	12139
KIF1B	-0.0043233	0	12140
ACTD	-0.0043313	0	12141
SFRP4	-0.004334	0	12142
LILRA5	-0.0043471	0	12143
DST	-0.0043471	0	12144
DIRAS3	-0.0043478	0	12145
GSTZ1	-0.0043536	0	12146
HRH2	-0.0043553	0	12147
NCS1	-0.0043573	0	12148
KCNJ4	-0.0043584	0	12149
MAP4K5	-0.0043646	0	12150
SLAIN1	-0.0043714	0	12151
KIF21B	-0.0043739	0	12152
TPI1	-0.0043751	0	12153
CRYL1	-0.0043787	0	12154
ULK2	-0.0043863	0	12155
MMP26	-0.0043881	0	12156
SIGMAR1	-0.0043894	0	12157
NPPA	-0.0043907	0	12158
PROK1	-0.0043917	0	12159
ACAA1	-0.0043929	0	12160
CACNA1A	-0.0043951	0	12161
C3AR1	-0.0043952	0	12162
STOML1	-0.0043958	0	12163
VSNL1	-0.0043986	0	12164

MCIDAS	-0.0043988	0	12165
MAOB	-0.0044014	0	12166
YY1AP1	-0.0044096	0	12167
MIR32	-0.0044124	0	12168
MIR613	-0.0044172	0	12169
MIR221	-0.0044241	0	12170
P2RX2	-0.0044258	0	12171
MIR9-2	-0.0044311	0	12172
CLDN8	-0.0044314	0	12173
CHST2	-0.004433	0	12174
UCN	-0.0044345	0	12175
LHX6	-0.0044398	0	12176
DDX4	-0.00444	0	12177
HADHA	-0.0044407	0	12178
ENTPD5	-0.004441	0	12179
SLC26A9	-0.0044448	0	12180
NSL1	-0.0044476	0	12181
XCL1	-0.004449	0	12182
CIZ1	-0.004452	0	12183
TRIM71	-0.0044561	0	12184
PVT1	-0.0044588	0	12185
PNPLA3	-0.0044595	0	12186
FOXC2	-0.0044617	0	12187
RAMP3	-0.0044678	0	12188
LGI3	-0.0044709	0	12189
AKAP5	-0.0044742	0	12190
FAM132A	-0.0044784	0	12191
BARD1	-0.0044887	0	12192
MIR214	-0.0044992	0	12193
DEFA8P	-0.0045004	0	12194
MIR208A	-0.0045005	0	12195
RAD54L	-0.0045008	0	12196
MUC3	-0.0045016	0	12197
PRPF6	-0.004502	0	12198
ADM2	-0.0045052	0	12199
TET3	-0.004506	0	12200
CILP	-0.0045087	0	12201
ABCA4	-0.0045088	0	12202
ANIB1	-0.0045122	0	12203
PTF1A	-0.0045135	0	12204
RNY5	-0.0045162	0	12205
LRPAP1	-0.0045183	0	12206
GRIK3	-0.0045217	0	12207

HIPK1	-0.0045246	0	12208
PRMT9	-0.0045324	0	12209
STX1B	-0.004534	0	12210
POLG	-0.0045361	0	12211
ALOX5AP	-0.0045389	0	12212
MOAP1	-0.0045429	0	12213
SLC2A5	-0.0045432	0	12214
CYP21A2	-0.004544	0	12215
VASH2	-0.0045509	0	12216
CEP76	-0.0045547	0	12217
POLH	-0.0045563	0	12218
ATP12A	-0.0045579	0	12219
MIR625	-0.0045601	0	12220
ACOT8	-0.0045612	0	12221
TP53AIP1	-0.0045636	0	12222
MIR494	-0.0045666	0	12223
CLCA2	-0.0045794	0	12224
CDK5RAP1	-0.0045799	0	12225
MPRIP	-0.0045806	0	12226
PTENP1	-0.0045848	0	12227
SENP2	-0.0045851	0	12228
RDH10	-0.0045856	0	12229
RPS26	-0.0045882	0	12230
DEFA4	-0.0045916	0	12231
DERL2	-0.0045947	0	12232
SCO2	-0.0046052	0	12233
CPT1C	-0.0046077	0	12234
DDAH2	-0.0046096	0	12235
MOB2	-0.0046125	0	12236
CCR3	-0.0046143	0	12237
NAP1L4	-0.0046243	0	12238
EBPL	-0.0046248	0	12239
LPSA	-0.0046283	0	12240
GPR142	-0.0046294	0	12241
PDRG1	-0.0046351	0	12242
MT1M	-0.0046377	0	12243
GRPR	-0.0046384	0	12244
SERPINB2	-0.004642	0	12245
KCNJ8	-0.0046471	0	12246
HRX	-0.0046497	0	12247
FBXO22	-0.0046561	0	12248
RPS29	-0.0046585	0	12249
JST	-0.0046622	0	12250

GEN1	-0.0046636	0	12251
EBM	-0.0046648	0	12252
SCGB2A1	-0.0046649	0	12253
KRT23	-0.0046739	0	12254
ACIN1	-0.0046753	0	12255
LAMTOR2	-0.0046757	0	12256
ANK2	-0.0046831	0	12257
CACNA1B	-0.0046849	0	12258
PEX19	-0.0046854	0	12259
ELOVL7	-0.0046868	0	12260
CIMT	-0.0046888	0	12261
AGPAT1	-0.0046897	0	12262
LARP4	-0.0046908	0	12263
ALPP	-0.0046944	0	12264
CBR3-AS1	-0.0046966	0	12265
SIK1	-0.0046992	0	12266
ZC3H12A	-0.0047017	0	12267
PVRL4	-0.004702	0	12268
SERPINB1	-0.0047021	0	12269
SLC16A10	-0.0047079	0	12270
THOP1	-0.0047088	0	12271
SMAR	-0.0047118	0	12272
NMUR2	-0.0047143	0	12273
NFKBIZ	-0.0047157	0	12274
NKX2-2	-0.0047172	0	12275
VSIG4	-0.0047182	0	12276
INHBB	-0.0047185	0	12277
RAD23A	-0.0047214	0	12278
CCNF	-0.0047221	0	12279
COPB2	-0.0047242	0	12280
FOXO6	-0.004727	0	12281
HCRTR1	-0.0047307	0	12282
DCTN6	-0.0047325	0	12283
CEP63	-0.0047333	0	12284
POU3F2	-0.0047451	0	12285
CXXC1	-0.0047596	0	12286
CTBP2	-0.0047599	0	12287
GPD1	-0.0047613	0	12288
MYLK3	-0.0047622	0	12289
NPY5R	-0.0047634	0	12290
NFIX	-0.0047701	0	12291
RNMT	-0.0047709	0	12292
ERP44	-0.0047714	0	12293

ASF1B	-0.0047733	0	12294
DAPK3	-0.0047747	0	12295
UACA	-0.0047823	0	12296
FGF18	-0.0047886	0	12297
VPS13C	-0.0047933	0	12298
CDKN2B-AS1	-0.0047938	0	12299
WWC1	-0.004794	0	12300
B3GNT3	-0.0047967	0	12301
MATN2	-0.0047981	0	12302
HOXA2	-0.0048004	0	12303
GALNT7	-0.0048088	0	12304
KCND3	-0.0048159	0	12305
LTBP3	-0.0048224	0	12306
UBIAD1	-0.0048227	0	12307
MTA2	-0.0048238	0	12308
GRIN2D	-0.0048338	0	12309
AHCY	-0.0048338	0	12310
NAGS	-0.0048358	0	12311
MAA	-0.0048372	0	12312
CDC14A	-0.0048453	0	12313
SLC25A18	-0.0048474	0	12314
MIR181C	-0.0048494	0	12315
PRSS50	-0.0048526	0	12316
NDRG4	-0.0048542	0	12317
MRGPRX2	-0.0048566	0	12318
KCNJ15	-0.0048588	0	12319
CABLES1	-0.0048597	0	12320
OFC1	-0.0048602	0	12321
TAF4B	-0.0048609	0	12322
ERLEC1	-0.0048633	0	12323
UCHL5	-0.0048642	0	12324
RPL11	-0.0048663	0	12325
TOR1A	-0.0048664	0	12326
APOA5	-0.0048696	0	12327
SULT1A1	-0.0048711	0	12328
SP5	-0.0048743	0	12329
RDH16	-0.0048791	0	12330
SYTL4	-0.0048882	0	12331
RAB5B	-0.0048886	0	12332
PPM1G	-0.0048926	0	12333
INPP5F	-0.0048935	0	12334
DKC1	-0.0048954	0	12335
HOTTIP	-0.0048959	0	12336

MAZ	-0.0049005	0	12337
CYCSP25	-0.0049014	0	12338
ACMSD	-0.0049065	0	12339
PPM1B	-0.0049078	0	12340
UHRF2	-0.0049105	0	12341
C1QTNF1	-0.0049108	0	12342
MIR422A	-0.0049134	0	12343
CUL4B	-0.0049138	0	12344
TINAG	-0.0049174	0	12345
REM2	-0.0049184	0	12346
MIR520G	-0.0049209	0	12347
ITPR3	-0.0049274	0	12348
PRRX2	-0.0049299	0	12349
WFDC2	-0.0049306	0	12350
TUSC5	-0.0049326	0	12351
CTSW	-0.0049431	0	12352
AGPAT6	-0.004947	0	12353
BW1	-0.004947	0	12354
CKS1BP7	-0.004947	0	12355
IGKV3D-11	-0.004947	0	12356
INGX	-0.004947	0	12357
LEPROT	-0.004947	0	12358
LINS	-0.004947	0	12359
METRNL	-0.004947	0	12360
PNPLA7	-0.004947	0	12361
SDR39U1	-0.004947	0	12362
SYNGR2	-0.004947	0	12363
TAR	-0.004947	0	12364
CLEC3B	-0.0049471	0	12365
RLIM	-0.004949	0	12366
RAB7A	-0.0049493	0	12367
CRY2	-0.0049496	0	12368
MDD2	-0.0049497	0	12369
TUSC2	-0.0049505	0	12370
CHD7	-0.0049528	0	12371
DISC1	-0.0049539	0	12372
SLC33A1	-0.0049541	0	12373
PNMA3	-0.0049545	0	12374
PRODH	-0.0049655	0	12375
SLC5A11	-0.0049656	0	12376
EXT2	-0.0049683	0	12377
GBA3	-0.0049698	0	12378
CLN5	-0.0049707	0	12379

GKN1	-0.0049744	0	12380
TUG1	-0.0049786	0	12381
RAB4B	-0.0049791	0	12382
HIRA	-0.0049821	0	12383
TRIM8	-0.0049886	0	12384
KLF15	-0.0049921	0	12385
NGDN	-0.0049936	0	12386
F11R	-0.0049941	0	12387
TM4SF1	-0.0049975	0	12388
GOT2	-0.0049998	0	12389
FAM83F	-0.0050002	0	12390
PMM2	-0.0050042	0	12391
HOXB13	-0.0050091	0	12392
SNTB2	-0.005013	0	12393
TMEM132A	-0.0050166	0	12394
MIR429	-0.0050169	0	12395
PTGER3	-0.0050173	0	12396
GLO1	-0.0050227	0	12397
TFPI2	-0.0050239	0	12398
FIGLA	-0.0050245	0	12399
NFIA	-0.0050259	0	12400
EDC4	-0.0050293	0	12401
SMARCAL1	-0.0050325	0	12402
SPOCK1	-0.0050357	0	12403
PIGT	-0.0050367	0	12404
IGFALS	-0.0050428	0	12405
MEP1A	-0.0050485	0	12406
D13S25	-0.0050516	0	12407
DYT10	-0.0050528	0	12408
HHAT	-0.0050541	0	12409
TP53RK	-0.0050568	0	12410
SYT3	-0.0050576	0	12411
VEZF1	-0.0050582	0	12412
CSNK2B	-0.0050608	0	12413
TDO2	-0.0050624	0	12414
SLC5A8	-0.0050706	0	12415
WNT9A	-0.0050762	0	12416
SCARNA14	-0.0050782	0	12417
FANCM	-0.0050789	0	12418
SLC25A33	-0.0050792	0	12419
NDUFV1	-0.0050795	0	12420
MIR194-1	-0.0050829	0	12421
SLC14A2	-0.0050843	0	12422

SAA2	-0.0050881	0	12423
FOXF1	-0.0050914	0	12424
KSR2	-0.0050915	0	12425
LSR	-0.0050974	0	12426
RAB23	-0.0050998	0	12427
RAMP2	-0.0051008	0	12428
MAD2L2	-0.0051008	0	12429
SIRT7	-0.0051077	0	12430
HIPK3	-0.0051122	0	12431
SLC26A3	-0.0051207	0	12432
LYRM1	-0.0051209	0	12433
HTRA1	-0.0051216	0	12434
MLXIPL	-0.0051217	0	12435
MIR211	-0.005122	0	12436
SORD	-0.0051293	0	12437
LAPTM4A	-0.0051425	0	12438
PLA2G16	-0.005149	0	12439
SSTR3	-0.0051529	0	12440
INSL5	-0.0051543	0	12441
REPIN1	-0.0051551	0	12442
CLMP	-0.0051557	0	12443
MIR151A	-0.0051592	0	12444
NEUROD1	-0.0051698	0	12445
CDC5L	-0.0051709	0	12446
GPT2	-0.0051714	0	12447
DDIT4	-0.0051726	0	12448
CKS1B	-0.0051738	0	12449
CBR1	-0.0051759	0	12450
CLASP2	-0.0051798	0	12451
SLC25A5	-0.0051818	0	12452
NEFL	-0.0051824	0	12453
TET1	-0.0051845	0	12454
SIL1	-0.0051882	0	12455
PLIN3	-0.0051886	0	12456
SLX1A	-0.0051937	0	12457
NDE1	-0.0051951	0	12458
SCGB3A1	-0.0052014	0	12459
LTBP4	-0.0052043	0	12460
DSE	-0.0052046	0	12461
HOOK1	-0.0052288	0	12462
PNPLA6	-0.005231	0	12463
NQO2	-0.0052326	0	12464
MC5R	-0.0052405	0	12465

ZFP36L1	-0.0052436	0	12466
DMAP1	-0.0052443	0	12467
WWOX	-0.0052483	0	12468
SHOX2	-0.005249	0	12469
MUC8	-0.005254	0	12470
TP53I3	-0.0052552	0	12471
IL20	-0.0052568	0	12472
LHCGR	-0.0052582	0	12473
VNN1	-0.0052626	0	12474
IGKV2D-38	-0.0052747	0	12475
RRM2B	-0.0052755	0	12476
COPA	-0.0052813	0	12477
FADS2	-0.0052869	0	12478
MIS18A	-0.0052869	0	12479
SPAST	-0.0052898	0	12480
CHRM3	-0.0052927	0	12481
SLC2A2	-0.0052956	0	12482
FRZB	-0.0053045	0	12483
STON1	-0.0053059	0	12484
ZBTB7A	-0.0053066	0	12485
APOM	-0.0053087	0	12486
LETMD1	-0.0053099	0	12487
MCM6	-0.0053203	0	12488
KCNE1	-0.0053225	0	12489
RBM45	-0.0053321	0	12490
WWTR1	-0.0053326	0	12491
SETMAR	-0.0053352	0	12492
SYN1	-0.0053359	0	12493
SCO	-0.0053399	0	12494
HSD3B2	-0.0053404	0	12495
ACACB	-0.0053424	0	12496
RNGTT	-0.0053442	0	12497
ZFR	-0.0053472	0	12498
PNLIP	-0.0053477	0	12499
CHKA	-0.0053478	0	12500
PARP2	-0.005351	0	12501
POLL	-0.0053548	0	12502
MIR27B	-0.0053582	0	12503
KCTD10	-0.0053683	0	12504
CPEB1	-0.0053691	0	12505
CLCNKB	-0.0053704	0	12506
SRRM4	-0.0053874	0	12507
MLL5	-0.0053912	0	12508

MATR3	-0.0053921	0	12509
SP4	-0.005397	0	12510
MIR148B	-0.0053985	0	12511
CYB561	-0.0053989	0	12512
MCM10	-0.0053997	0	12513
BAI1	-0.0054016	0	12514
LAMC2	-0.0054017	0	12515
TDH	-0.005404	0	12516
ZDHHC23	-0.0054048	0	12517
EEF2	-0.0054093	0	12518
ODF1	-0.0054131	0	12519
PSMD5	-0.0054163	0	12520
GZMK	-0.0054292	0	12521
ADRA1B	-0.0054309	0	12522
HCAR2	-0.0054314	0	12523
ADRA2B	-0.005435	0	12524
SNRK	-0.0054372	0	12525
SYT8	-0.0054441	0	12526
DUSP7	-0.0054459	0	12527
TBC1D8	-0.0054516	0	12528
SLCO1B3	-0.0054519	0	12529
GNL3L	-0.0054521	0	12530
TSPYL2	-0.0054526	0	12531
GPR17	-0.0054574	0	12532
ATG12	-0.0054602	0	12533
AZU1	-0.0054616	0	12534
TDP2	-0.0054679	0	12535
LZTS1	-0.0054714	0	12536
PDLIM2	-0.0054831	0	12537
MAP4	-0.0055071	0	12538
MIR377	-0.0055076	0	12539
MIR615	-0.0055086	0	12540
HEXB	-0.0055121	0	12541
ADAMTS7	-0.0055163	0	12542
MIR17	-0.0055176	0	12543
NRD1	-0.0055226	0	12544
CPEB4	-0.0055259	0	12545
SCNN1G	-0.0055285	0	12546
ANKK1	-0.0055398	0	12547
APOC1	-0.0055438	0	12548
SMARCD1	-0.0055447	0	12549
RXFP3	-0.0055459	0	12550
PPM1F	-0.005549	0	12551

MFN2	-0.0055522	0	12552
ALDH3A2	-0.0055561	0	12553
GLP2R	-0.0055565	0	12554
BHLHE41	-0.0055618	0	12555
CHIA	-0.0055643	0	12556
XRN1	-0.0055674	0	12557
RTN4	-0.0055699	0	12558
HMGN5	-0.0055736	0	12559
GJC1	-0.0055737	0	12560
UCK2	-0.0055934	0	12561
SMS	-0.0055969	0	12562
AVPR1A	-0.0056067	0	12563
CPT1A	-0.0056069	0	12564
RHCE	-0.0056105	0	12565
C1S	-0.0056204	0	12566
BSCL2	-0.0056293	0	12567
TRRAP	-0.0056328	0	12568
SERPINA10	-0.005634	0	12569
MAFF	-0.0056343	0	12570
OXR1	-0.0056397	0	12571
SPAG5	-0.0056398	0	12572
TOP2A	-0.0056412	0	12573
KERA	-0.0056441	0	12574
CNN1	-0.0056622	0	12575
NRN1	-0.0056641	0	12576
SERPINA3	-0.0056699	0	12577
SULT1A3	-0.0056704	0	12578
SLC41A1	-0.0056724	0	12579
ACTR1B	-0.005673	0	12580
ARHGAP22	-0.0056738	0	12581
XRCC3	-0.0056824	0	12582
ZC3H12D	-0.0056952	0	12583
DUT	-0.0056959	0	12584
PBK	-0.0056982	0	12585
SUB1	-0.0056994	0	12586
HLP	-0.0056997	0	12587
MIRLET7G	-0.0057046	0	12588
CTH	-0.005706	0	12589
C1QL1	-0.0057114	0	12590
DPYSL4	-0.0057124	0	12591
CELF2	-0.0057175	0	12592
KHSRP	-0.0057333	0	12593
BRSK2	-0.0057346	0	12594

COMP	-0.005737	0	12595
AMY2A	-0.0057391	0	12596
SLC16A2	-0.0057467	0	12597
HNRNPU	-0.0057471	0	12598
NPL	-0.0057525	0	12599
ITGA3	-0.0057581	0	12600
SLC39A1	-0.0057627	0	12601
S100A14	-0.0057667	0	12602
PRDM2	-0.0057674	0	12603
FFAR2	-0.0057775	0	12604
MDH2	-0.0057781	0	12605
AGTR2	-0.0057799	0	12606
ABCC9	-0.0057821	0	12607
GADD45B	-0.0057848	0	12608
CSRP1	-0.0057866	0	12609
EIF3M	-0.0057922	0	12610
BCAT2	-0.0057965	0	12611
KCNJ2	-0.0057974	0	12612
KDM4B	-0.0057993	0	12613
NUSAP1	-0.0058042	0	12614
DEDD	-0.0058067	0	12615
ASMT	-0.0058118	0	12616
KIF2C	-0.0058131	0	12617
EMR1	-0.0058145	0	12618
FTL	-0.0058207	0	12619
GPR180	-0.0058223	0	12620
ITGA9	-0.0058236	0	12621
PEG10	-0.0058238	0	12622
CPA3	-0.0058307	0	12623
HMGB2	-0.0058365	0	12624
EDC3	-0.0058377	0	12625
SPRR1B	-0.0058414	0	12626
RECQL	-0.0058509	0	12627
SLC6A1	-0.0058522	0	12628
ZFAND5	-0.0058614	0	12629
MAP4K3	-0.0058656	0	12630
ACF	-0.0058695	0	12631
HEYL	-0.0058723	0	12632
ABI3BP	-0.0058783	0	12633
ZFP42	-0.0058934	0	12634
NPR2	-0.005903	0	12635
PNKP	-0.0059054	0	12636
CAPRIN1	-0.0059098	0	12637

OBFC1	-0.0059146	0	12638
SRGN	-0.0059262	0	12639
CKMT1A	-0.0059266	0	12640
TRPM7	-0.0059333	0	12641
SCNN1A	-0.0059373	0	12642
SLC5A3	-0.0059388	0	12643
OXTR	-0.005943	0	12644
ASCL2	-0.005949	0	12645
TAF1C	-0.0059503	0	12646
ADAMTS9	-0.0059529	0	12647
MGEA5	-0.0059557	0	12648
MCM5	-0.0059655	0	12649
WNT5B	-0.0059669	0	12650
TIGAR	-0.0059799	0	12651
RFC1	-0.0059808	0	12652
RSF1	-0.0059954	0	12653
OAP	-0.006001	0	12654
IMP3	-0.0060038	0	12655
FBN2	-0.0060089	0	12656
HPT	-0.0060121	0	12657
CCKBR	-0.0060263	0	12658
PURA	-0.0060329	0	12659
SLC39A6	-0.0060329	0	12660
PGRMC1	-0.0060405	0	12661
MAF1	-0.0060428	0	12662
RGS5	-0.0060439	0	12663
SMTN	-0.006045	0	12664
DEFB1	-0.0060451	0	12665
LAMB1	-0.0060641	0	12666
TNFRSF10D	-0.0060809	0	12667
HSF2	-0.0060893	0	12668
AS3MT	-0.0060921	0	12669
NPY2R	-0.0060977	0	12670
RAD51B	-0.0060981	0	12671
CT49	-0.0061	0	12672
HESX1	-0.0061017	0	12673
PXDN	-0.0061057	0	12674
CYP2B7P1	-0.0061077	0	12675
GALR1	-0.0061083	0	12676
NLE1	-0.0061084	0	12677
EFEMP1	-0.0061194	0	12678
HIST1H2AH	-0.0061211	0	12679
KCTD1	-0.0061233	0	12680

SPARCL1	-0.0061392	0	12681
CAPG	-0.0061417	0	12682
PID1	-0.0061451	0	12683
ICAM5	-0.0061458	0	12684
PAGR1	-0.0061738	0	12685
CACNA1D	-0.0061774	0	12686
CPQ	-0.0061782	0	12687
S100A12	-0.0061799	0	12688
TRPM6	-0.0061891	0	12689
STRBP	-0.0061892	0	12690
SLC25A29	-0.00619	0	12691
ATP6V1H	-0.0061927	0	12692
FANCG	-0.006193	0	12693
FBF1	-0.006196	0	12694
FXYD1	-0.006196	0	12695
SLC29A2	-0.0061974	0	12696
AKR1C3	-0.0062138	0	12697
HTR3A	-0.006215	0	12698
SHBG	-0.0062185	0	12699
ELAVL4	-0.0062263	0	12700
MCPH1	-0.0062325	0	12701
SLC11A2	-0.0062334	0	12702
FBXO31	-0.0062342	0	12703
PAXIP1	-0.0062357	0	12704
GSK3A	-0.0062386	0	12705
DYNC1I2	-0.0062412	0	12706
PDCD10	-0.0062548	0	12707
PPM1L	-0.0062601	0	12708
CPA1	-0.0062604	0	12709
ND2	-0.0062659	0	12710
TNFAIP8	-0.0062665	0	12711
HSD11B2	-0.0062703	0	12712
XYLT1	-0.0062707	0	12713
TTK	-0.0062735	0	12714
CCNA1	-0.0062857	0	12715
ANKS1B	-0.0062884	0	12716
PPIF	-0.0063038	0	12717
RPS28	-0.0063046	0	12718
GYS1	-0.0063055	0	12719
ADAMTS4	-0.0063072	0	12720
MIR139	-0.0063114	0	12721
ARC	-0.0063148	0	12722
MSH5	-0.0063263	0	12723

TCHH	-0.0063272	0	12724
FBLN2	-0.0063364	0	12725
CRAC1	-0.0063628	0	12726
ADORA3	-0.0063714	0	12727
TLE4	-0.0063745	0	12728
MIR675	-0.006375	0	12729
UHMK1	-0.0063776	0	12730
SLC13A4	-0.006391	0	12731
UNG	-0.0063998	0	12732
AQP9	-0.006402	0	12733
FKBP5	-0.0064031	0	12734
ATP7A	-0.0064052	0	12735
SERPINB8	-0.0064137	0	12736
PRG4	-0.0064161	0	12737
EIF4EBP2	-0.0064162	0	12738
KLKB1	-0.0064311	0	12739
HCRTR2	-0.0064313	0	12740
MC2R	-0.0064316	0	12741
CDC27	-0.0064327	0	12742
SNED1	-0.006436	0	12743
LECT1	-0.0064361	0	12744
PIK3R3	-0.0064393	0	12745
RBM38	-0.0064447	0	12746
HPD	-0.0064478	0	12747
EXOSC6	-0.0064494	0	12748
MIR28	-0.0064526	0	12749
KLK6	-0.0064559	0	12750
DGAT2	-0.0064572	0	12751
RECQL4	-0.0064687	0	12752
RNU105A	-0.0064798	0	12753
SLC2A8	-0.0064871	0	12754
IGKV2-28	-0.0064902	0	12755
PPT1	-0.006491	0	12756
ACAT2	-0.0064913	0	12757
TRIM2	-0.0064965	0	12758
BACE2	-0.006497	0	12759
BP2	-0.0064971	0	12760
PLOD3	-0.0064992	0	12761
MACROD1	-0.0065031	0	12762
HTR6	-0.006504	0	12763
INPP4B	-0.0065583	0	12764
FZD1	-0.00656	0	12765
PMEPA1	-0.0065669	0	12766

VIPR1	-0.0065808	0	12767
STXBP4	-0.0065836	0	12768
ACOT1	-0.0065848	0	12769
PAF1	-0.0066238	0	12770
GAS5	-0.0066318	0	12771
POLR2B	-0.0066474	0	12772
JAM3	-0.0066538	0	12773
PCSK5	-0.0066567	0	12774
GUK1	-0.0066585	0	12775
IFRD1	-0.0066609	0	12776
CERS1	-0.0066625	0	12777
SLC12A6	-0.0066684	0	12778
IGF2BP2	-0.0066741	0	12779
EHF	-0.0066782	0	12780
TACR2	-0.0066912	0	12781
KCND2	-0.0067064	0	12782
BMP8B	-0.006709	0	12783
TFDP3	-0.0067095	0	12784
ATP6V1C1	-0.0067111	0	12785
C14ORF101	-0.0067111	0	12786
ADD3	-0.0067173	0	12787
PDSS1	-0.0067256	0	12788
MEPE	-0.0067264	0	12789
STATH	-0.0067282	0	12790
HSPA1B	-0.0067282	0	12791
9-Sep	-0.0067486	0	12792
NHLH2	-0.0067507	0	12793
ASIC3	-0.0067587	0	12794
BEST1	-0.0067609	0	12795
FOXQ1	-0.0067695	0	12796
SEPP1	-0.0067774	0	12797
TREH	-0.0067774	0	12798
PYY3	-0.0067779	0	12799
HJURP	-0.0067816	0	12800
MIR148A	-0.0067858	0	12801
PAFAH1B3	-0.0067975	0	12802
ABHD5	-0.0068021	0	12803
SMAD5-AS1	-0.0068088	0	12804
RENBP	-0.006811	0	12805
CAMK4	-0.0068112	0	12806
BUB1	-0.0068148	0	12807
GPHB5	-0.0068157	0	12808
C1QTNF3	-0.0068163	0	12809

MMP28	-0.006817	0	12810
ATP5G3	-0.0068224	0	12811
ARTN	-0.006823	0	12812
NOM1	-0.0068236	0	12813
CYP11B2	-0.0068319	0	12814
WTAP	-0.0068371	0	12815
TRPS1	-0.0068493	0	12816
URI1	-0.0068502	0	12817
PTK7	-0.0068524	0	12818
GAL3ST1	-0.0068587	0	12819
DSC2	-0.0068609	0	12820
APOA4	-0.0068666	0	12821
EPHX2	-0.0068692	0	12822
FTMT	-0.006873	0	12823
TRPA1	-0.006877	0	12824
SHD	-0.0068776	0	12825
SLC40A1	-0.006889	0	12826
PRG1	-0.0068997	0	12827
CPZ	-0.0069057	0	12828
VCAN	-0.0069079	0	12829
CDCA7	-0.0069117	0	12830
CHRNB2	-0.006922	0	12831
GLRX	-0.00693	0	12832
RIF1	-0.0069365	0	12833
IGKV1-13	-0.006949	0	12834
KLHL3	-0.0069575	0	12835
AWAT2	-0.0069642	0	12836
GPT	-0.0069664	0	12837
SIX1	-0.0069857	0	12838
TCEA1	-0.0069891	0	12839
SLC12A4	-0.0069906	0	12840
MMP15	-0.0069919	0	12841
RAD52	-0.0069949	0	12842
EPAS1	-0.0070131	0	12843
GGTLC1	-0.0070177	0	12844
AQP1	-0.0070179	0	12845
ABCC5	-0.0070225	0	12846
TNKS	-0.0070344	0	12847
FBLN5	-0.0070353	0	12848
ADAM9	-0.0070428	0	12849
TUB	-0.00705	0	12850
CCBP2	-0.0070554	0	12851
COX3	-0.007058	0	12852

HCN1	-0.0070642	0	12853
TPCN1	-0.0070874	0	12854
GNPDA2	-0.0070964	0	12855
FOXC1	-0.0071031	0	12856
BRD7	-0.0071048	0	12857
TRNAU1	-0.0071068	0	12858
CERS5	-0.0071094	0	12859
DLL3	-0.0071171	0	12860
FABP2	-0.0071244	0	12861
PFKFB3	-0.0071468	0	12862
ARID3A	-0.0071567	0	12863
KAT7	-0.0071584	0	12864
RYR1	-0.0071709	0	12865
AKAP4	-0.0071822	0	12866
TERC	-0.0071844	0	12867
ZRANB3	-0.0071904	0	12868
ATP2B2	-0.0071934	0	12869
EED	-0.0071974	0	12870
CXCL14	-0.007207	0	12871
CACNG2	-0.0072145	0	12872
MIR20A	-0.0072165	0	12873
TRAF4	-0.0072222	0	12874
MIR4443	-0.0072297	0	12875
MXI1	-0.0072472	0	12876
BW17	-0.0072637	0	12877
MIR498	-0.0072721	0	12878
MAPT	-0.0072736	0	12879
MIR100	-0.0072822	0	12880
ANG	-0.0072874	0	12881
GNMT	-0.0072923	0	12882
B9D1	-0.0072926	0	12883
QPCT	-0.0073152	0	12884
SLC10A5	-0.0073259	0	12885
MTFMT	-0.0073272	0	12886
FKTN	-0.0073283	0	12887
H19	-0.0073394	0	12888
SETDB1	-0.0073403	0	12889
STC2	-0.0073488	0	12890
CPNE1	-0.0073495	0	12891
DIXDC1	-0.0073539	0	12892
ESPL1	-0.0073705	0	12893
TAP2	-0.0073716	0	12894
EXOSC3	-0.0073733	0	12895

MIR210	-0.0073861	0	12896
LCAT	-0.0073907	0	12897
GPR39	-0.0073915	0	12898
WDR5	-0.0073995	0	12899
PRIMA1	-0.0074019	0	12900
BP5	-0.007409	0	12901
E2F7	-0.0074111	0	12902
NKD2	-0.0074161	0	12903
ATP5J	-0.0074178	0	12904
NANOS3	-0.0074186	0	12905
AQP5	-0.0074196	0	12906
CHEK1	-0.0074274	0	12907
NR2E1	-0.0074282	0	12908
CNPY2	-0.0074413	0	12909
CTSL	-0.0074521	0	12910
KCNN2	-0.0074521	0	12911
MTBP	-0.0074534	0	12912
MELK	-0.0074643	0	12913
MUL1	-0.0074681	0	12914
SPINK7	-0.007469	0	12915
GJA5	-0.0074701	0	12916
SLC43A2	-0.0074764	0	12917
PHOX2B	-0.0074768	0	12918
WNT4	-0.0074912	0	12919
RPS19BP1	-0.007499	0	12920
HCN2	-0.0075126	0	12921
EI24	-0.007528	0	12922
MCU	-0.0075345	0	12923
CFD	-0.0075385	0	12924
CDKN1C	-0.0075442	0	12925
LTBP2	-0.0075509	0	12926
RAPGEF5	-0.0075609	0	12927
MIR217	-0.0075686	0	12928
SLC5A2	-0.0075695	0	12929
SLC7A1	-0.0075875	0	12930
FSTL3	-0.0075918	0	12931
CDK8	-0.0075935	0	12932
PBXIP1	-0.0075945	0	12933
GPDS1	-0.0076043	0	12934
MDK	-0.0076149	0	12935
GTS	-0.0076237	0	12936
ICT1	-0.0076245	0	12937
IGF2R	-0.0076261	0	12938

FAM3B	-0.007635	0	12939
AQP7	-0.0076448	0	12940
MCHR1	-0.0076448	0	12941
ALPL	-0.007649	0	12942
SYP	-0.0076497	0	12943
SIK2	-0.0076509	0	12944
DRAM1	-0.0076544	0	12945
MIR140	-0.0076656	0	12946
KCNB1	-0.0076705	0	12947
POLE4	-0.0076729	0	12948
HSD11B1	-0.0076825	0	12949
CALM2	-0.0076858	0	12950
E4F1	-0.0076877	0	12951
MSMB	-0.0076998	0	12952
GRM4	-0.0077124	0	12953
PER2	-0.0077129	0	12954
SIRT4	-0.0077131	0	12955
DMC1	-0.0077314	0	12956
LPAR3	-0.0077441	0	12957
TRPM2	-0.0077566	0	12958
MTHFR	-0.0077715	0	12959
NKX3-1	-0.0077834	0	12960
SASH1	-0.007789	0	12961
RPS7	-0.0077975	0	12962
PPP1R2	-0.0077977	0	12963
MIR34B	-0.0077981	0	12964
CFHR1	-0.0077984	0	12965
FABP3	-0.0078121	0	12966
XK	-0.0078127	0	12967
RCBTB1	-0.0078148	0	12968
RBBP4	-0.007817	0	12969
MSH2	-0.0078264	0	12970
ADAMTS2	-0.0078413	0	12971
GOT1	-0.0078441	0	12972
SLC11A1	-0.0078443	0	12973
LITAF	-0.0078454	0	12974
A2M	-0.0078483	0	12975
KCNQ5	-0.0078558	0	12976
CPB2	-0.0078673	0	12977
PFDN4	-0.0078684	0	12978
AQP8	-0.0078805	0	12979
MAOA	-0.0078826	0	12980
CDK6	-0.0078883	0	12981

MIR23B	-0.0078911	0	12982
RNASE1	-0.0078925	0	12983
MIR519D	-0.0079106	0	12984
SERPINA12	-0.0079126	0	12985
MTDH	-0.007916	0	12986
AIS	-0.0079318	0	12987
COL7A1	-0.0079458	0	12988
IPP	-0.0079477	0	12989
DRD4	-0.0079578	0	12990
FUT8	-0.0079603	0	12991
HMGCS2	-0.0079604	0	12992
PSEN2	-0.0079666	0	12993
KIF20A	-0.0079703	0	12994
VIMP	-0.0079706	0	12995
AGTR1	-0.0079706	0	12996
PTGIS	-0.0079739	0	12997
SSX2	-0.0079751	0	12998
FBLN1	-0.0079765	0	12999
SULF2	-0.0079776	0	13000
MIR192	-0.0079782	0	13001
OMG	-0.0079785	0	13002
XPA	-0.0079903	0	13003
GOLGA6A	-0.0079976	0	13004
ODC1	-0.008	0	13005
HTR1F	-0.0080097	0	13006
SCAP	-0.0080212	0	13007
CCKAR	-0.0080373	0	13008
RPL5	-0.0080405	0	13009
NBL1	-0.0080415	0	13010
VGF	-0.0080613	0	13011
CIDEC	-0.0080669	0	13012
PHOX2A	-0.0081006	0	13013
CCNC	-0.0081017	0	13014
GRIA3	-0.0081033	0	13015
PDE4D	-0.0081045	0	13016
ENPP1	-0.0081048	0	13017
CNTF	-0.0081091	0	13018
ASL	-0.0081109	0	13019
LMBR1	-0.0081364	0	13020
CHN2	-0.0081406	0	13021
HDGF	-0.0081425	0	13022
MIR184	-0.0081438	0	13023
GJD2	-0.0081442	0	13024

CENPJ	-0.0081549	0	13025
CST3	-0.0081581	0	13026
HAS2	-0.0081593	0	13027
EIF4A2	-0.0081594	0	13028
BP3	-0.0081655	0	13029
IGKV1-22	-0.0081781	0	13030
OLFM1	-0.0081938	0	13031
GRM3	-0.0082009	0	13032
SARDH	-0.0082176	0	13033
HTR2B	-0.0082187	0	13034
CCL13	-0.0082602	0	13035
L1RE1	-0.0082697	0	13036
RARRES2	-0.0082721	0	13037
SHANK3	-0.0082813	0	13038
SEMA7A	-0.008285	0	13039
CKB	-0.0082912	0	13040
AATF	-0.0083041	0	13041
KAT8	-0.008306	0	13042
SLC12A7	-0.0083373	0	13043
HMBOX1	-0.0083435	0	13044
FBRS	-0.0083455	0	13045
HCFC1	-0.0083468	0	13046
PHLDB1	-0.0083652	0	13047
RAD54B	-0.0083664	0	13048
LMOD1	-0.0083748	0	13049
ZNF217	-0.0083873	0	13050
LAP3	-0.0084055	0	13051
DENR	-0.0084082	0	13052
SLC2A14	-0.0084179	0	13053
CERS6	-0.0084224	0	13054
L1CAM	-0.0084327	0	13055
MIR29C	-0.0084328	0	13056
STEAP4	-0.0084418	0	13057
ZNF148	-0.0084423	0	13058
GRP	-0.008485	0	13059
DMRT1	-0.0084876	0	13060
UCN2	-0.0085045	0	13061
PRB2	-0.0085131	0	13062
LDHA	-0.0085196	0	13063
TRPM8	-0.0085314	0	13064
PPP1R1B	-0.0085314	0	13065
TRPM5	-0.0085586	0	13066
CPP	-0.0085602	0	13067

SAA1	-0.0085697	0	13068
MMP8	-0.0085699	0	13069
TSPAN7	-0.008584	0	13070
NUAK1	-0.008592	0	13071
LIPG	-0.0085945	0	13072
CCDC3	-0.0086051	0	13073
MIR106B	-0.0086053	0	13074
LPIN1	-0.0086112	0	13075
MAT2A	-0.0086297	0	13076
ERCC2	-0.0086393	0	13077
PIK3C2A	-0.0086424	0	13078
KCNA4	-0.0086642	0	13079
CFL2	-0.0086692	0	13080
FOXE1	-0.0086725	0	13081
GLP1R	-0.0087009	0	13082
TRH	-0.0087119	0	13083
INSL3	-0.0087133	0	13084
ZNF2	-0.0087376	0	13085
CEP55	-0.0087431	0	13086
C20ORF85	-0.0087435	0	13087
CDC20	-0.008746	0	13088
MAS1	-0.0087483	0	13089
HTC2	-0.0087508	0	13090
P4HA1	-0.0087679	0	13091
CPS1	-0.0087886	0	13092
RBP2	-0.0088114	0	13093
MIR483	-0.0088321	0	13094
LTBP1	-0.0088653	0	13095
SLC6A2	-0.0088737	0	13096
AMTN	-0.008874	0	13097
FAM3A	-0.008906	0	13098
RPS27	-0.0089111	0	13099
GPR119	-0.0089133	0	13100
OVGP1	-0.0089149	0	13101
CHFR	-0.0089193	0	13102
LIG3	-0.0089296	0	13103
AMBP	-0.0089296	0	13104
MCTS1	-0.0089305	0	13105
TRNAE1	-0.0089553	0	13106
ATRX	-0.0089625	0	13107
VRK1	-0.0089664	0	13108
IGFBP2	-0.0089723	0	13109
FOXG1	-0.0089804	0	13110

FGF13	-0.0089827	0	13111
ALDOB	-0.0089867	0	13112
ARRB1	-0.0089909	0	13113
VTCN1	-0.0090055	0	13114
AGPAT9	-0.0090106	0	13115
MIR497	-0.0090107	0	13116
MIR152	-0.0090161	0	13117
UCP2	-0.0090166	0	13118
MIR141	-0.0090198	0	13119
STK39	-0.0090238	0	13120
RITA	-0.0090279	0	13121
SPAG8	-0.0090448	0	13122
PRPH	-0.0090615	0	13123
NUFIP2	-0.0090719	0	13124
SLC8A1	-0.009072	0	13125
ERCC1	-0.0090793	0	13126
CLCN7	-0.0090853	0	13127
ACAN	-0.0090905	0	13128
PRSS1	-0.0090985	0	13129
GAS1	-0.0091139	0	13130
SLC25A4	-0.009121	0	13131
POLD4	-0.0091231	0	13132
TCF21	-0.0091256	0	13133
MYBL2	-0.0091504	0	13134
MGLL	-0.0091536	0	13135
ORC2	-0.0092018	0	13136
RXFP2	-0.009213	0	13137
MSI1	-0.0092134	0	13138
GJB1	-0.0092149	0	13139
MIRLET7D	-0.0092398	0	13140
RMI1	-0.0092442	0	13141
SMC6	-0.0092467	0	13142
PRMT1	-0.0092506	0	13143
GFPT1	-0.009273	0	13144
ACAA2	-0.0092973	0	13145
SORT1	-0.0093066	0	13146
TP53INP2	-0.009316	0	13147
TAC1	-0.0093177	0	13148
GJB2	-0.0093243	0	13149
HAS3	-0.0093304	0	13150
PAX4	-0.009357	0	13151
MLST8	-0.0093578	0	13152
DIO3	-0.0093757	0	13153

PR@	-0.0093774	0	13154
PPM1K	-0.0094091	0	13155
DRD5	-0.0094109	0	13156
OCM	-0.0094208	0	13157
PDK4	-0.0094336	0	13158
HAND2	-0.0094409	0	13159
SERPINE2	-0.0094459	0	13160
NEU2	-0.0094623	0	13161
FOXF2	-0.0094715	0	13162
NIT2	-0.009473	0	13163
P2RY6	-0.0094918	0	13164
PIH	-0.0094979	0	13165
RGCC	-0.0095048	0	13166
SCN11A	-0.0095461	0	13167
MCM3	-0.0095783	0	13168
CERS4	-0.0095957	0	13169
INSIG1	-0.009613	0	13170
ABCC8	-0.0096274	0	13171
SIM1	-0.0096313	0	13172
CDK3	-0.0096387	0	13173
ANGPTL4	-0.0096611	0	13174
NKX6-1	-0.0096769	0	13175
CLCN3	-0.0097044	0	13176
CLDN11	-0.0097064	0	13177
ASAH2	-0.0097133	0	13178
ZBTB22	-0.0097342	0	13179
PIGF	-0.0097599	0	13180
GPR55	-0.0097622	0	13181
RGN	-0.0097673	0	13182
HRH1	-0.0097873	0	13183
PCK1	-0.0098196	0	13184
MIR19A	-0.0098216	0	13185
TJP3	-0.009828	0	13186
S100G	-0.0098307	0	13187
CKS2	-0.0098324	0	13188
CDH11	-0.0098346	0	13189
PDE4B	-0.0098427	0	13190
ARPP21	-0.0098558	0	13191
COL4A3	-0.0098689	0	13192
RECK	-0.0098843	0	13193
MTUS1	-0.009886	0	13194
ADK	-0.0098931	0	13195
DKK2	-0.0098934	0	13196

H2AFZ	-0.0098939	0	13197
NPR3	-0.0099019	0	13198
LATS2	-0.0099053	0	13199
NANOGP8	-0.0099285	0	13200
FTO	-0.0099703	0	13201
TERF2	-0.0099768	0	13202
NDP	-0.0100202	0	13203
REST	-0.0100458	0	13204
SLC4A2	-0.0100519	0	13205
SDS	-0.0100625	0	13206
ARID4B	-0.0100627	0	13207
SEMA4C	-0.0100945	0	13208
FANCA	-0.0101067	0	13209
FXR1	-0.0101138	0	13210
MMP17	-0.0101219	0	13211
GABBR1	-0.0101235	0	13212
FAM168B	-0.010127	0	13213
TPH2	-0.0101316	0	13214
SLC2A3	-0.0101426	0	13215
ALDH2	-0.0101591	0	13216
FNDC5	-0.0101749	0	13217
CDK7	-0.0101831	0	13218
KLK7	-0.0102007	0	13219
TBPL1	-0.0102057	0	13220
MYL4	-0.0102462	0	13221
REV1	-0.0102531	0	13222
KLF17	-0.0102544	0	13223
EDN3	-0.0102551	0	13224
BCAN	-0.0102636	0	13225
NMU	-0.0102669	0	13226
ZNF143	-0.0102766	0	13227
CCNB1	-0.010291	0	13228
HAS1	-0.0103064	0	13229
F13A1	-0.0103182	0	13230
NOS1	-0.0103207	0	13231
GLRA3	-0.0103376	0	13232
IDDM2	-0.0103426	0	13233
USP9X	-0.01036	0	13234
PITX3	-0.0103912	0	13235
ASRGL1	-0.0103916	0	13236
EDN2	-0.0104001	0	13237
MCF2L2	-0.0104243	0	13238
MIR107	-0.0104299	0	13239

SEMA3B	-0.010445	0	13240
HELLS	-0.0104491	0	13241
IDE	-0.0104757	0	13242
DRD3	-0.0104785	0	13243
MIR132	-0.010485	0	13244
FZR1	-0.0105175	0	13245
RXFP1	-0.0105354	0	13246
GRIA4	-0.0105407	0	13247
SLC6A3	-0.0105673	0	13248
DIO2	-0.0106124	0	13249
RPH3AL	-0.0106128	0	13250
FBP1	-0.0106214	0	13251
NPNT	-0.0106325	0	13252
MARCKS	-0.0106877	0	13253
AKT1S1	-0.0106944	0	13254
NPS	-0.0107036	0	13255
TRPV1	-0.0107055	0	13256
CPA6	-0.0107161	0	13257
WISP2	-0.0107208	0	13258
ECE1	-0.010725	0	13259
TNFRSF21	-0.0107251	0	13260
GALP	-0.0107328	0	13261
CDC25B	-0.0107385	0	13262
AANAT	-0.0107939	0	13263
PNOC	-0.0108035	0	13264
CAPN2	-0.0108036	0	13265
NPAS4	-0.0108156	0	13266
ADORA2B	-0.0108411	0	13267
CELA3B	-0.0108621	0	13268
PDCD4	-0.0108751	0	13269
SRSF3	-0.0108908	0	13270
PHEX	-0.0109063	0	13271
CKAP2	-0.0109153	0	13272
ACSL1	-0.0109195	0	13273
PMS1	-0.0109389	0	13274
E2F4	-0.0109565	0	13275
GRIK2	-0.0109643	0	13276
KLK4	-0.0109757	0	13277
PABPC1	-0.0109781	0	13278
LIG4	-0.0109983	0	13279
CPT2	-0.0110508	0	13280
MIR30D	-0.0110564	0	13281
LAMTOR1	-0.0110593	0	13282

PLOD2	-0.0110679	0	13283
ADCYAP1R1	-0.0111277	0	13284
GIPR	-0.0111356	0	13285
DGAT1	-0.0111515	0	13286
ERCC5	-0.0111659	0	13287
AMBN	-0.0111752	0	13288
FAIM2	-0.0111777	0	13289
E2F6	-0.0111999	0	13290
SESN2	-0.0112248	0	13291
PAH	-0.0112297	0	13292
CLCN5	-0.011231	0	13293
VAMP2	-0.0112362	0	13294
ASS1	-0.0112369	0	13295
ATP1B1	-0.0112548	0	13296
INSIG2	-0.0112576	0	13297
GLS2	-0.0112657	0	13298
BACH1	-0.0112722	0	13299
MIR26A1	-0.0112964	0	13300
NTSR1	-0.011306	0	13301
MIR10B	-0.0113114	0	13302
TNFAIP6	-0.0113196	0	13303
ACHE	-0.0113341	0	13304
SLC16A7	-0.0113836	0	13305
C1QTNF9	-0.0114259	0	13306
HBA1	-0.0114345	0	13307
SEC23A	-0.0114353	0	13308
GRM5	-0.011513	0	13309
FBXO8	-0.0115195	0	13310
MCM8	-0.0115837	0	13311
MNX1	-0.0115942	0	13312
ATRIP	-0.0115961	0	13313
PACAP	-0.0116522	0	13314
PER1	-0.0117327	0	13315
PREP	-0.0117576	0	13316
HK2	-0.01177	0	13317
MIR25	-0.011772	0	13318
NT3	-0.0118177	0	13319
EEF2K	-0.0118259	0	13320
SULT4A1	-0.0118262	0	13321
WRN	-0.0118276	0	13322
UCP3	-0.0118284	0	13323
MIRLET7B	-0.0118392	0	13324
LMX1A	-0.0118447	0	13325

TIMP3	-0.0118615	0	13326
BRIP1	-0.0118699	0	13327
MUTYH	-0.0118985	0	13328
HOMER2	-0.011946	0	13329
ERCC6	-0.0119647	0	13330
GFRA1	-0.0119745	0	13331
PC	-0.0119937	0	13332
RPLP1	-0.0120014	0	13333
O3FAR1	-0.0120029	0	13334
CGN	-0.0120156	0	13335
GRIN2A	-0.0120508	0	13336
SLC10A4	-0.012089	0	13337
FABP7	-0.0121293	0	13338
TDP1	-0.0121471	0	13339
GAD2	-0.0121582	0	13340
GRIK5	-0.0121667	0	13341
GRIN2C	-0.0122014	0	13342
NANOS2	-0.012205	0	13343
UTS2	-0.0122773	0	13344
KCNA1	-0.0123377	0	13345
GPR77	-0.0123588	0	13346
MYT1	-0.0123634	0	13347
TNR	-0.0124041	0	13348
SPO11	-0.0124133	0	13349
TPPP3	-0.012466	0	13350
DDB2	-0.012469	0	13351
GDF5	-0.0124736	0	13352
MSH3	-0.0125657	0	13353
AQP3	-0.0125827	0	13354
SSTR5	-0.0125992	0	13355
ADIPOR1	-0.0126443	0	13356
SMC1A	-0.0126675	0	13357
HOMER1	-0.0126915	0	13358
CDC25A	-0.0127057	0	13359
APOD	-0.012723	0	13360
EME1	-0.0127345	0	13361
ECT	-0.0127392	0	13362
GRIK1	-0.0127466	0	13363
CHGA	-0.0128816	0	13364
GMNN	-0.0128817	0	13365
EDNRA	-0.0128828	0	13366
CXCL6	-0.0128841	0	13367
TP53INP1	-0.0129135	0	13368

PNPLA2	-0.012964	0	13369
CRHBP	-0.0129805	0	13370
DBI	-0.0129824	0	13371
SLC17A8	-0.0129922	0	13372
TACR1	-0.013011	0	13373
HINT1	-0.0130197	0	13374
MECP2	-0.0130324	0	13375
CCNB2	-0.0130456	0	13376
P2RY1	-0.0130513	0	13377
CCL14	-0.0130533	0	13378
NMB	-0.0130547	0	13379
MIR200B	-0.0130683	0	13380
CLDN3	-0.0131469	0	13381
CCNE2	-0.0131522	0	13382
HSD3B1	-0.0131581	0	13383
1-Dec	-0.0132096	0	13384
THBS2	-0.0132281	0	13385
BCL2L2	-0.0132854	0	13386
NA	-0.0132995	0	13387
SULT2A1	-0.0133142	0	13388
LIN28A	-0.0133264	0	13389
MIR18A	-0.013341	0	13390
SLC32A1	-0.0133535	0	13391
CREM	-0.0133645	0	13392
PALB2	-0.0134208	0	13393
POLB	-0.0134318	0	13394
PARN	-0.0134345	0	13395
TICRR	-0.0134451	0	13396
CXCL3	-0.0134899	0	13397
CRB2	-0.0134914	0	13398
CCNG1	-0.0135124	0	13399
NTF4	-0.0135422	0	13400
FEN1	-0.0135503	0	13401
MIR205	-0.0135619	0	13402
SETD7	-0.0135831	0	13403
C10ORF10	-0.013629	0	13404
STX1A	-0.0136706	0	13405
XPC	-0.0137083	0	13406
COL3A1	-0.0137088	0	13407
MIR375	-0.0137885	0	13408
NTF3	-0.0137918	0	13409
CHST3	-0.0138202	0	13410
PCSK6	-0.0138446	0	13411

SHC2	-0.0138563	0	13412
APLNR	-0.0139473	0	13413
ADCY6	-0.0139518	0	13414
MIR200A	-0.0139745	0	13415
TIMP4	-0.0139826	0	13416
CDC7	-0.0140157	0	13417
NEFH	-0.0140431	0	13418
LOXL2	-0.0141137	0	13419
TCEAL1	-0.0141156	0	13420
E2F5	-0.0141374	0	13421
TFF2	-0.0141433	0	13422
CDS1	-0.0141718	0	13423
TIMP2	-0.0141877	0	13424
ESM1	-0.0142258	0	13425
IK	-0.0142334	0	13426
KISS1R	-0.0142722	0	13427
SLC10A2	-0.0143124	0	13428
GRIN2B	-0.0143481	0	13429
ORC1	-0.0143525	0	13430
PPYR1	-0.0143545	0	13431
CARTPT	-0.0143633	0	13432
GHRHR	-0.014374	0	13433
DBF4	-0.0144614	0	13434
SRD5A1	-0.0144668	0	13435
HTR4	-0.0145364	0	13436
SNAP25	-0.0145785	0	13437
ZFPM2	-0.014614	0	13438
SLC38A7	-0.0146221	0	13439
SLC18A3	-0.0146737	0	13440
AVPR1B	-0.0147633	0	13441
LDLRAP1	-0.0148439	0	13442
CCNA2	-0.0148726	0	13443
LIN28B	-0.0148773	0	13444
CC2D1A	-0.0148827	0	13445
PPM1D	-0.0148887	0	13446
ARSD	-0.0149179	0	13447
NUCB2	-0.0149723	0	13448
P2RX3	-0.0150234	0	13449
RAD17	-0.0151886	0	13450
AQP4	-0.0152492	0	13451
SLC12A5	-0.0152749	0	13452
UBE2C	-0.0152902	0	13453
LIG1	-0.0153052	0	13454

CRY1	-0.0153733	0	13455
MMP11	-0.0153999	0	13456
CNP	-0.0154013	0	13457
RRM1	-0.0154242	0	13458
SLC12A1	-0.0154754	0	13459
CBS	-0.015527	0	13460
TUBB3	-0.015588	0	13461
OR2AG1	-0.0155931	0	13462
RPA2	-0.0156513	0	13463
DCX	-0.0156755	0	13464
OLIG2	-0.015686	0	13465
MCM7	-0.0156955	0	13466
MRXS5	-0.015725	0	13467
RPA1	-0.0157542	0	13468
E2F2	-0.0157586	0	13469
MEG3	-0.015759	0	13470
NEIL1	-0.0157736	0	13471
FANCD2	-0.0158436	0	13472
RAD50	-0.0159473	0	13473
MRE11A	-0.0160509	0	13474
NPPC	-0.0161063	0	13475
HUS1	-0.0161466	0	13476
ERCC4	-0.0161566	0	13477
CDC45	-0.0162447	0	13478
IGFBP4	-0.0162558	0	13479
EHMT1	-0.0162602	0	13480
IAPP	-0.0162816	0	13481
SCG2	-0.0164267	0	13482
SST	-0.0165751	0	13483
IGKV1D-37	-0.016584	0	13484
SCG5	-0.0166539	0	13485
SLC18A2	-0.016715	0	13486
PCOS1	-0.0167299	0	13487
MMP16	-0.0168459	0	13488
AGRP	-0.0168709	0	13489
NOC2L	-0.0168994	0	13490
FAAH	-0.0169245	0	13491
ENO2	-0.0170255	0	13492
FFAR1	-0.0171577	0	13493
MC3R	-0.0172293	0	13494
B4GALT1	-0.0172512	0	13495
SERPINA6	-0.017303	0	13496
SIRT6	-0.0173822	0	13497

MIR122	-0.0173954	0	13498
PCSK2	-0.0175274	0	13499
OPRM1	-0.0175662	0	13500
ERVW-4	-0.0177688	0	13501
ADAMTS1	-0.0178534	0	13502
CGB	-0.0179257	0	13503
GHSR	-0.018051	0	13504
CPE	-0.0180969	0	13505
PCSK1	-0.018134	0	13506
ACE2	-0.0183418	0	13507
HTR1B	-0.0183799	0	13508
CDT1	-0.0184189	0	13509
PMS2	-0.0185314	0	13510
PAPPA	-0.0189144	0	13511
CBX4	-0.019	0	13512
SLC17A7	-0.0190168	0	13513
CRHR2	-0.0190934	0	13514
CDC6	-0.0191263	0	13515
GAD1	-0.019211	0	13516
RBFOX3	-0.0192668	0	13517
PTTG1	-0.0192951	0	13518
KISS1	-0.0193239	0	13519
SIRT3	-0.019596	0	13520
CLDN5	-0.019608	0	13521
HMGA2	-0.0197943	0	13522
EXO1	-0.019803	0	13523
DNA2	-0.0198913	0	13524
RAD9A	-0.0199606	0	13525
CORT	-0.0200943	0	13526
PNMT	-0.0201923	0	13527
POLE3	-0.0205636	0	13528
SLC6A4	-0.020646	0	13529
DRD2	-0.0207967	0	13530
RAD1	-0.0208213	0	13531
GHRL	-0.0208254	0	13532
IGKV2D-29	-0.0209388	0	13533
GNRH1	-0.0209658	0	13534
STC1	-0.0213064	0	13535
MC4R	-0.0213279	0	13536
MIR16-1	-0.0214032	0	13537
HTR2C	-0.0214956	0	13538
KCNJ11	-0.0214983	0	13539
SLC1A3	-0.0216719	0	13540

MIR93	-0.0217758	0	13541
APLN	-0.0218206	0	13542
SLC17A6	-0.0218889	0	13543
ADAMTS5	-0.0219156	0	13544
MCM2	-0.0219954	0	13545
MCM4	-0.0223784	0	13546
GRIA2	-0.0223948	0	13547
SRI	-0.0223951	0	13548
PDYN	-0.0225356	0	13549
RBBP8	-0.0227623	0	13550
SLC10A3	-0.0229124	0	13551
MALAT1	-0.0229674	0	13552
CA3	-0.0235364	0	13553
MMS	-0.0235934	0	13554
CGA	-0.0237938	0	13555
PENK	-0.0238125	0	13556
COMT	-0.0238282	0	13557
MUS81	-0.0241514	0	13558
TH	-0.024362	0	13559
ADORA2A	-0.0249429	0	13560
CLSPN	-0.0249765	0	13561
ADCYAP1	-0.0251178	0	13562
GRM2	-0.0251248	0	13563
CCNE1	-0.0252757	0	13564
SLC1A2	-0.0254036	0	13565
DBH	-0.0257521	0	13566
HTR2A	-0.0257861	0	13567
CA4	-0.0258551	0	13568
GIP	-0.0263414	0	13569
GRIA1	-0.0263837	0	13570
ZEB2	-0.0270196	0	13571
GAP43	-0.0271508	0	13572
WEE1	-0.0278639	0	13573
HTR1A	-0.0281038	0	13574
TOPBP1	-0.0288167	0	13575
FMR1	-0.0289999	0	13576
ADCY1	-0.029663	0	13577
VIP	-0.0299351	0	13578
CCK	-0.0300765	0	13579
SLC10A7	-0.0309227	0	13580
CRHR1	-0.0309628	0	13581
HCRT	-0.0313307	0	13582
PYY	-0.031721	0	13583

NTS	-0.0318099	0	13584
CALB2	-0.0321305	0	13585
PPY	-0.0321521	0	13586
GHRH	-0.036264	0	13587
CALCA	-0.0364455	0	13588
E2F3	-0.0372863	0	13589
DRD1	-0.0374334	0	13590
GRIN1	-0.039286	0	13591
CRH	-0.0399708	0	13592
PVALB	-0.0411117	0	13593
NPY	-0.0418681	0	13594
CALB1	-0.0420617	0	13595

BTK	ABCB1	28036258
BTK	ABL1	15939795
BTK	AKT1	10974038
BTK	BCL2	27157620
BTK	BCL2L1	12459462
BTK	BCR	9586640
BTK	BLNK	8855292
BTK	BTK	7522330
BTK	CALR	23596312
BTK	CARD11	FTMJ_elsvr-10-1016-j-bcmd-2015-06-016
BTK	CAV1	11751885
BTK	CBL	10427990
BTK	CD19	FTMJ_wiley_journals-10-1002-eji-201243081
BTK	CD34	18323795
BTK	CD38	8524855
BTK	CD40	26864273
BTK	CD44	24555985
BTK	CPAT1	FTMJ_wiley_journals-10-1111-bjh-13969
BTK	CREB1	14597636
BTK	CSF1R	18523268
BTK	CXCL12	FTMJ_elsvr-10-1016-j-hoc-2014-06-003
BTK	CXCR4	FTMJ_elsvr-10-1016-j-hoc-2014-06-003
BTK	DDX41	25704810
BTK	EGFR	FTMJ_elsvr-10-1016-j-ajpath-2016-01-019
BTK	EP300	28040583
BTK	EPHB2	24270740
BTK	ETS1	26209625
BTK	EXOSC10	FTMJ_wiley_journals-10-1002-eji-201646596
BTK	FAS	9751072
BTK	FCGR2B	FTMJ_wiley_journals-10-1002-eji-201040521
BTK	FGF2	14597636
BTK	FLT3	25605370
BTK	GTF2I	9012831
BTK	HAVCR2	25172495
BTK	HRASLS	19017959
BTK	IFNA1	22281426
BTK	IGKV2D-18	11788586
BTK	IGLL1	19302039
BTK	IKZF1	23977012
BTK	IL1	12724322
BTK	IL10	17207856
BTK	IL2	9556577
BTK	IL3	9642236

BTK	IL5	7538439
BTK	IL6	21113169
BTK	IL7	14634110
BTK	INPP5K	FTMJ_elsvr-10-1053-j-gastro-2010-12-005
BTK	INS	26034172
BTK	IRAK1	23836557
BTK	ITK	10556826
BTK	JAK1	FTMJ_elsvr-10-1016-j-cytogfr-2015-07-004
BTK	LYN	12533043
BTK	MAL	24840642
BTK	MAPK8	9642236
BTK	MET	21784852
BTK	MTOR	25083818
BTK	MYC	25605370
BTK	MYD88	21441935
BTK	NFATC1	18281276
BTK	NFKB1	10811866
BTK	NOTCH2	26034172
BTK	PHB2	11373296
BTK	PI3	10201980
BTK	PIK3AP1	11163197
BTK	PIKFYVE	14614849
BTK	PIP4K2C	14614849
BTK	PLCG1	22649724
BTK	PLCG2	10840066
BTK	PLEK	7972043
BTK	PRKCB	15237108
BTK	PTK2	FTMJ_elsvr-10-1016-j-pbiomolbio-2009-12-009
BTK	PTPN6	10327049
BTK	RAC1	11943162
BTK	RELA	18276597
BTK	SERPINA5	21422473
BTK	SH2D1A	15315965
BTK	SHH	FTMJ_wiley_journals-10-1002-mc-20604
BTK	SLC6A8	23596312
BTK	SMAD3	28065931
BTK	SMARCA1	15096481
BTK	SND1	17785824
BTK	STAP1	10518561
BTK	STAT1	24612681
BTK	SYK	12533043
BTK	TEC	9159207
BTK	TIRAP	FTMJ_elsvr-10-1016-j-exphem-2015-05-016

BTK	TLR2	25213142
BTK	TLR3	22454496
BTK	TLR4	15081522
BTK	TLR7	25634698
BTK	TLR8	FTMJ_elsvr-10-1016-j-immuni-2008-06-004
BTK	TLR9	24612681
BTK	TNFSF11	18281276
BTK	TOLLIP	FTMJ_wiley_journals-10-1111-bjh-12895
BTK	TP53	27630139
BTK	TRIM63	FTMJ_wiley_journals-10-1002-eji-201344030
BTK	UGCG	FTMJ_elsvr-10-1053-j-gastro-2010-12-005
BTK	VPREB1	26522721
BTK	WAS	19109164
BTK	WIPF1	25413351
BTK	XBP1	FTMJ_wiley_journals-10-1002-iid3-57
