# Supplementary material for: Identification of pharmacodynamic biomarker hypotheses through literature analysis with IBM Watson
Source: PLoS One. 2019 Apr 8;14(4):e0214619. doi: 10.1371/journal.pone.0214619 (PMC6453528; doi:10.1371/journal.pone.0214619)
Supplement: S1 Fig — Searched entities (inputs) are represented by white circles and connected gene entities by blue circles. Curvature of the connecting arrows indicates reciprocal (curved) or non-reciprocal (straight) relationships. Distance from the searched entity is associated with the number of documents supporting the connection: nearer circles are connected by relationships in more documents than farther circles. (PDF) [file pone.0214619.s003.pdf]

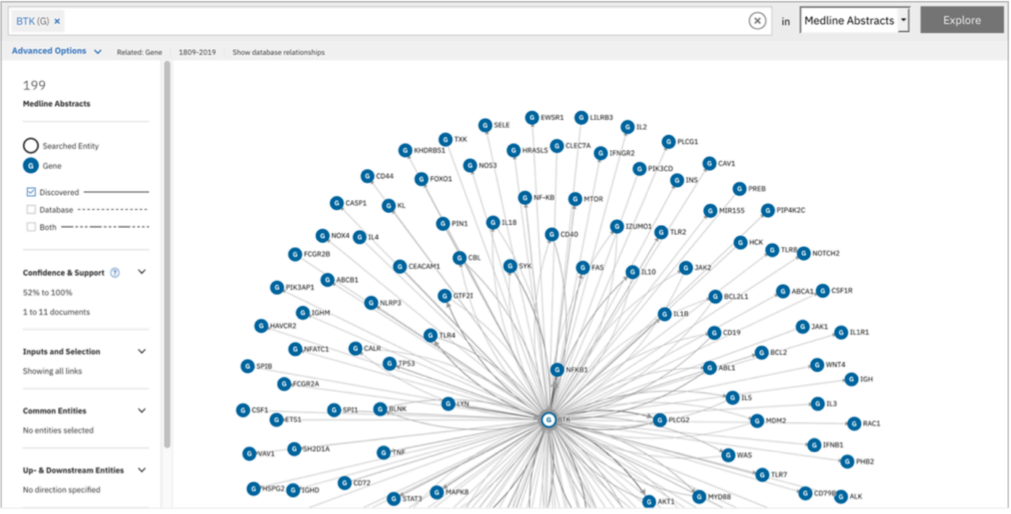
BTK (G) ×
in
Medline Abstracts
Explore
Advanced Options
Related: Gene
1809-2019
Show database relationships
199
Medline Abstracts
Searched Entity
Gene
Discovered
Database
Both
Confidence & Support
52% to 100%
1 to 11 documents
Inputs and Selection
Showing all links
Common Entities
No entities selected
Up- & Downstream Entities
No direction specified
SELE
EWSR1
LILRB3
IL2
TXK
KHDRBS1
HRASLS
CLEC7A
IFNGR2
PLCG1
NOS3
PIK3CD
CAV1
CD44
FOXO1
INS
NF-KB
MTOR
PREB
CASP1
KL
MIR155
PIP4K2C
PIN1
IL18
IZUMO1
TLR2
NOX4
IL4
CD40
HCK
FCGR2B
CBL
TLR8
NOTCH2
CEACAM1
SYK
FAS
IL10
JAK2
ABCB1
PIK3AP1
GTF2I
BCL2L1
ABCA1
CSF1R
NLRP3
IGHM
IL1B
HAVCR2
TLR4
CD19
JAK1
IL1R1
NFATC1
CALR
BCL2
TP53
WNT4
SPIB
NFKB1
ABL1
IGH
FCGR2A
IL5
LYN
IL3
CSF1
SPI1
BLNK
ETS1
BTK
PLCG2
MDM2
RAC1
IFNB1
VAV1
SH2D1A
TNF
WAS
PHB2
TLR7
HSPG2
IGHD
CD72
STAT3
MAPK8
CD79B
ALK
AKT1
MYD88
