# Supplementary material for: Identification of pharmacodynamic biomarker hypotheses through literature analysis with IBM Watson
Source: PLoS One. 2019 Apr 8;14(4):e0214619. doi: 10.1371/journal.pone.0214619 (PMC6453528; doi:10.1371/journal.pone.0214619)
Supplement: S2 Fig — On the left is a summary of gene-gene network connections which can be selected to show sentence-level evidence (on the right) for the relationship connection. (PDF) [file pone.0214619.s004.pdf]

Filters

Relationship

Select a relationship or relationship type to view the documents

All Documents

BTK → AKT1
6 documents (91% confidence)

BTK → PHB2
1 document (60% confidence)

BTK → BCR
5 documents (89% confidence)

BTK → SELE
1 document (69% confidence)

BTK → FOXO1
1 document (71% confidence)

BTK → IL10
3 documents (88% confidence)

[illegible]

Back to **Explore a Network**

199 Medline Abstracts found for **BTK**

Sort by: Most Recently Published

Authors: Xu,Xiaoqing; Xu,Jia; Wu,Jiacheng; Hu...
Nov 15, 2018
Medline_30318148

BTK elicited by extrinsic E-selectin engagement phosphorylates cytoplasmic IFN-γR2, facilitating EFhd2 binding and promoting IFN-γR2 trafficking from Golgi to cell membrane.

Authors: Franco,Andrea; Kraus,Zachary; Li,Hui...
Nov 14, 2018
Medline_30107486

We found that activating signaling molecules such as CD19, active PLCγ2 and BTK were rapidly recruited to FCRL5 upon engagement, suggesting a novel activating function for FCRL5.

Authors: Li,Tianfeng; Deng,Yi; Shi,Yu; Tian,Ruij...
Nov 1, 2018
Medline_30013190

Bruton's tyrosine kinase potentiates ALK signaling and serves as a potential therapeutic target of neuroblastoma.

Ibrutinib therapy releases leukemic surface IgM from antigen drive in chronic lymphocytic leukemia patients.

Clinical cancer research : an official journal of t...
Authors: Drennan,Samantha; Chiodin,Giorgia; ...
Oct 29, 2018
Medline_30373751

The sIgM was fully capable of mediating phosphorylation of SYK which lies upstream of BTK in the B-Cell Receptor pathway.

Stimulus strength determines the BTK-dependence of the SHIP1-deficient phenotype in IgE/antigen-triggered mast

Scientific reports
Authors: Zorn,Carolin N; Simonowski,Anne; Hu...
Oct 19, 2018
Medline_30341350

Moreover, they suggest that reduced activation or curtate expression of SHIP1 can be compensated by pharmacological inhibition of BTK and vice versa.

CXCR4- and BCR-triggered integrin activation in B-cell chronic lymphocytic leukemia cells depends on JAK2-activated

Oncotarget
Authors: Montresor,Alessio; Toffali,Lara; Rigo,...
Oct 12, 2018
Medline_30416684

A comparative analysis of 36 B-CLL patients demonstrates that JAK2-dependent BTK regulatory role on integrin activation by CXCL12 is fully conserved in CLL cells.
