# Supplementary material for: Identification of pharmacodynamic biomarker hypotheses through literature analysis with IBM Watson
Source: PLoS One. 2019 Apr 8;14(4):e0214619. doi: 10.1371/journal.pone.0214619 (PMC6453528; doi:10.1371/journal.pone.0214619)

Predict Genes for Entity of interest BTK (G) ×

Advanced Options ⌄ Ranked by Strength | Associated and Non-associated predictions | Top 100 rows

Heatmap | Rank rationale

Filter genes

Direct Evidence

Show: All Criteria ⓘ most similar to your input gene, with evidence to

Rank	Entity		Score	Relations
✔ 1	TNF	Ⓐ	0.53919	2856
✔ 2	IFNG		0.48556	1832
✔ 3	NF-KB	Ⓐ	0.47234	1955
✔ 4	AKT1	Ⓐ	0.45811	2730
✔ 5	IL6	Ⓐ	0.45547	1800
✔ 6	IL2		0.41024	843
✔ 7	IL4	Ⓐ	0.40827	1020
✔ 8	IL1B	Ⓐ	0.39976	1635
✔ 9	STAT3		0.39350	1429
✔ 10	CD28		0.38829	409
✔ 11	EGFR	Ⓐ	0.38597	1456

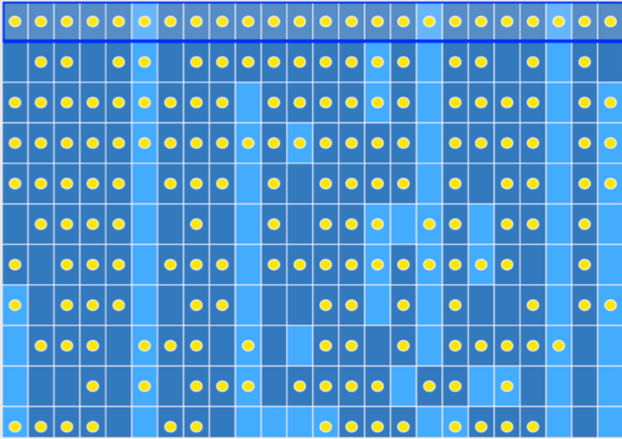

Supplement: S3 Fig — BTK is the input gene, and on the left-hand side we see the score for each gene in the matrix factorization result. On the right-hand side are selected columns from the original matrix with yellow dots indicating non-zero values in the original input matrix and darker shading indicating higher floating-point values in the resulting matrix after matrix factorization is applied. This gives the scientist both a value for likelihood of being downstream of BTK, as well as both direct and indirect evidence that generated that value. The direct evidence is sentences in publications that say BTK effects the gene. The indirect evidence is genes that are similar to BTK in their behavior, that show a downstream effect on the gene. (PDF) [file pone.0214619.s005.pdf]
